# Non‐Criminal Justice Interventions for Countering Cognitive and Behavioural Radicalisation Amongst Children and Adolescents: A Systematic Review of Effectiveness and Implementation: A Systematic Review

**DOI:** 10.1002/cl2.70079

**Published:** 2025-12-28

**Authors:** James Lewis, Sarah Marsden, James Hewitt, Chloe Squires, Anna Stefaniak

**Affiliations:** ^1^ Handa Centre for the Study of Terrorism and Political Violence (CSTPV), School of International Relations University of St Andrews St Andrews Scotland; ^2^ School of Psychology and Neuroscience University of St Andrews St Andrews Scotland

## Abstract

Growing numbers of young people are encountering the counter‐terrorism system. This international trend raises important questions about the effectiveness of efforts to prevent and counter childhood and adolescent radicalisation. This review examines the effectiveness and implementation of interventions designed to counter cognitive and behavioural radicalisation amongst children and adolescents up to 19 years of age. It examines secondary and tertiary interventions working with at‐risk and radicalised youth outside the criminal justice system. This focus reflects the growing recognition that diverting young people away from the criminal justice system helps mitigate the risks of securitisation, criminalisation, and stigmatisation. This review sought to answer three research questions: (1) Are secondary and tertiary interventions delivered outside of the criminal justice system effective at countering the cognitive and behavioural radicalisation of children and adolescents? (2) Are secondary and tertiary interventions delivered outside of the criminal justice system being implemented as intended? (3) What factors influence how interventions working with children and adolescents outside of the criminal justice system are implemented? Studies were identified through electronic searches of 20 academic and 50 grey literature sources; hand searches of academic journals and clinical trial repositories; forward and backward citation searches; and consultation with academic experts. Studies published after 2000 in English were eligible for inclusion. Searches of academic and grey literature sources were conducted in August and September 2024, respectively, with all remaining searches completed by December 2024. Studies had to report on secondary or tertiary interventions or prevention work outside of the criminal justice system (i.e., outside of correctional, custodial, or probation contexts) that sought to counter cognitive and behavioural radicalisation, often referred to as countering violent extremism (CVE) programming. Three types of interventions were included: (1) those designed for children and adolescents; (2) those working with children and adolescents as part of wider cohorts, provided a significant proportion of clients were aged 19 or under, or that provided insights specific to children and adolescents; or (3) those working with youth (or young people), broadly defined. Experimental or stronger quasi‐experimental research designs were eligible for inclusion in the assessment of effectiveness (Objective I). Inclusion criteria for the assessment of implementation (Objective II) and of implementation factors (Objective III) were broader, and included non‐experimental quantitative designs and qualitative research. The corpus of evidence was screened by trained reviewers, guided by screening tools, across a title/abstract and full‐text screening phase. Data were extracted using a piloted tool to capture key information on each included study. Risk of bias information was extracted using relevant existing tools. Effectiveness data from a single study was converted into a standardised effect size. Data relating to implementation was coded through a process of inductive and deductive coding. Twenty‐nine studies were included in the review. Only one quasi‐experimental study was eligible for inclusion in the analysis of effectiveness (Objective I). In this study, Kolbe (2019) reported that at‐risk young people participating in a group‐based intervention who also received individualised home‐based support were less likely to join a violent extremist group than young people who only participated in the group‐based intervention (Chi‐square: 5.295, *df* = 1, *p* = 0.021). This represents a large effect (Odds ratio: 0.181, 95% CI [0.0366, 0.8963]). However, the fact that this study was assessed as having a serious risk of bias, coupled with the lack of other eligible research designs, means there was insufficient evidence to draw conclusions about effectiveness. Evidence relating to implementation was drawn from qualitative and mixed‐methods studiesassessed as moderate to high quality. Seven studies examined how interventions were implemented (Objective II), including whether they were implemented as intended, or had been able to reach eligible clients. These studies reported positive results, but highlighted challenges that interventions might face in reaching those most in need of support (i.e., those most ‘at risk’), and the need for interventions to be flexible and adapt their approach when needed. Twenty‐eight studies examined implementation factors, and twenty‐seven analysed moderators (Objective III). Three types of implementation factors were identified: (1) Structural and systemic factors (e.g., knowledge and expertise, multi‐agency and partnership working); (2) Identifying and engaging eligible clients; and (3) Working with clients (e.g., relational processes; client‐centred approach). The moderators were the national and local context, client characteristics, and delivery contexts. The review found very little research that speaks to the effectiveness of interventions working with youth outside the criminal justice system. The absence of such research does not mean that current interventions should be considered ineffective, nor that a criminal justice response is likely to be more effective. It is also important to recognise that previous research has found that the evidence base underpinning these interventions is evolving, and that stronger non‐experimental research designs are being used to analyse effectiveness. However, in common with the wider evidence base on secondary and tertiary interventions – including those delivered through the criminal justice system – current knowledge remains limited. The field has developed a better understanding of the factors that facilitate or create barriers to implementation. Research on implementation offers a solid foundation from which to develop a more evidence‐based approach to policy and practice and provides several avenues for future research. The most obvious implication for policy and practice is to build evaluation into programme design. Notwithstanding the challenges to evaluating secondary and tertiary interventions, this effort should prioritise the use of stronger methodological designs that make it possible to demonstrate programme effectiveness. Further implications for policymakers and practitioners to consider include promoting a non‐stigmatising youth‐centred approach; applying a holistic and multidisciplinary social‐ecological approach; developing systems and structures that support practitioners and young people; recognising the importance of relational processes that enable systems and structures to work effectively; and active and ongoing engagement with families.

## 1 Plain Language Summary

*Non‐criminal justice interventions are potentially important ways of preventing and countering youth radicalisation; however, their effectiveness remains poorly understood.*

There is insufficient evidence to determine the effectiveness of current approaches, as only one eligible impact evaluation was identified. However, there is a growing body of evidence relating to implementation that provides important lessons for intervention design and delivery.

### 1.1 What Is This Review About?

Internationally, governments are increasingly concerned about children and adolescents being drawn towards violent extremism and terrorism. This review examines the effectiveness and implementation of interventions working to prevent cognitive and behavioural radicalisation of children and adolescents. It focuses on interventions that work with at‐risk and radicalised children and adolescents (up to 19 years old) outside the criminal justice system.

### 1.2 What Is the Aim of This Review?

This review summarises evidence from empirical studies that examine the effectiveness (*n* = 1), and the implementation (*n* = 29) of interventions designed to counter the cognitive and behavioural radicalisation of at‐risk and radicalised children and adolescents outside of criminal justice contexts.

### 1.3 What Are the Main Findings of This Review?

#### What Studies Are Included?

One study is included in the analysis of effectiveness: a quasi‐experimental evaluation of an intervention in an unnamed city in East Africa. Twenty‐nine studies are included in the analysis of implementation, including seven studies that examined whether interventions are implemented as intended.

The research is international in scope, and includes studies focused on 11 named countries, and 1 unnamed country in East Africa: the United Kingdom (*n* = 9); Australia (*n* = 5); Kenya (*n* = 4); Germany (*n* = 3); The Netherlands (*n* = 2); Canada (*n* = 2); USA (*n* = 2); Somalia (*n* = 1); Austria (*n* = 1); Uzbekistan (*n* = 1); Sweden (*n* = 1).

*1. Are secondary and tertiary interventions delivered outside of the criminal justice system effective at countering the cognitive and behavioural radicalisation of children and adolescents?*

There is insufficient evidence to draw a firm conclusion, as only one evaluation of effectiveness, assessed as having a serious risk of bias, was eligible for inclusion.

*2. Are secondary and tertiary interventions delivered outside of the criminal justice system being implemented as intended?*

Seven studies examined whether interventions were being implemented as intended, including the extent to which they had been able to identify and engage with at‐risk and/or radicalised young people. Although some interventions may face challenges in identifying those most in need of support/most at risk, these studies reported that the evaluated interventions were generally implemented as intended.

*3. What factors influence how interventions working with children and adolescents outside of the criminal justice system are implemented?*

Twenty‐eight studies identified factors that facilitated implementation. Structural features of interventions that supported implementation included the availability of relevant multidisciplinary knowledge and expertise; well‐functioning multi‐agency and partnership working; and appropriate resourcing. The importance of well‐defined and well‐functioning processes for identifying and assessing clients was also highlighted. Specific ways of working with young people and their families – including building strong relationships and adopting a client‐centred and contextualised approach – were also crucial in facilitating intervention work.

Twenty‐seven studies examined moderators – the specific features of the contexts in which interventions operated, or of the clients they worked with – that impacted intervention delivery, including features of local or national contexts; client characteristics; and delivery context.

### 1.4 What Do the Findings of This Review Mean?

The field still lacks a robust understanding of intervention effectiveness, and so further work examining whether and how these interventions work will be crucial. In the absence of robust evidence of effectiveness, the evidence base relating to implementation provides a number of important lessons that can be used to inform the design and delivery of interventions working with at‐risk and radicalised children and adolescents outside of the criminal justice system. However, more research is needed to examine whether and how the implementation factors identified in this review contribute to intervention success.

### 1.5 How Up‐to‐Date Is This Review?

Studies published after 2000 were eligible for inclusion. Electronic searches were completed between August and December 2024.

## 2 Background

### 2.1 The Problem, Condition, or Issue

This review examines interventions delivered outside of the criminal justice system that work to prevent and counter the radicalisation of children and adolescents, defined here as young people aged 19 years of age or younger (WHO undated).^1^ It speaks to the pressing problem of childhood and adolescent radicalisation that is becoming an increasing concern for governments around the world (Bronsard et al. 2022).

The concept of radicalisation remains contested and continues to be criticised by some authors for lacking conceptual clarity and a robust evidence base (Ford and Jackson 2023). However, efforts to counter radicalisation – more commonly known as countering violent extremism (CVE) programming – are now a central component of counter‐terrorism strategies around the world (Lewis, Marsden, Cherney, et al. 2024). Although recognising that there is no single definition of radicalisation (Neumann 2013), this review adopts the definition used by the United States Department of Homeland Security (DHS) as it reflects common understandings of radicalisation as having both cognitive and behavioural dimensions:

‘The process through which an individual changes from a non‐violent belief system to a belief system that includes the willingness to actively advocate, facilitate, or use unlawful violence as a method to effect societal or political change’.

(DHS 2017)

The process of radicalisation is therefore understood as resulting in individuals actively supporting or using ‘unlawful violence as a method to effect societal or political change’. In this view, radicalisation can be cognitive and/or behavioural (Wolfowicz et al. 2021). We use the work of Vergani et al. (2020) to distinguish between the two processes, whilst recognising that they are often related. *Cognitive* radicalisation results in individuals adopting views that are indicative of ‘support for violent extremist acts (e.g., terrorist attacks), people (e.g., Anders Breivik), and groups (e.g., Al Qaeda) that committed acts of violent extremism (e.g., terrorism)’, and behavioural radicalisation in an individual ‘committing an act of violent extremism (e.g., terrorism) or joining a violent extremist group (e.g., Islamic State of Iraq and the Levant or Al Qaeda)’ (Vergani et al. 2020, 859).

There are growing concerns about the cognitive and behavioural radicalisation of children and adolescents, and in particular the growing number of young people being arrested, charged and convicted for counter‐terrorism offences in different countries (Europol 2022; Rose and Vale 2023). This raises important questions as to the role that the criminal justice system should play in responding to childhood and adolescent radicalisation. Criminal justice responses remain an important part of any counter‐terrorism toolkit, and there will be cases where the severity of extremist offending by a young person may leave no viable alternative to arrest and imprisonment (Rose and Vale 2023; Wallner 2021). However, the importance of diverting at‐risk and even radicalised youth away from the criminal justice system when possible is widely recognised, owing to concerns that contact with the criminal justice system can be particularly distressing and stigmatising for children and adolescents (Global Counterterrorism Forum [GCTF] 2015; Kessels 2017; UNODC 2021).

There are important ethical and legal issues associated with tackling childhood and adolescent radicalisation through the criminal justice system. The United Nations Convention on the Rights of the Child states that the arrest, conviction, and imprisonment of children must be a last resort, even in the context of terrorism offenses (UNODC 2021). Interventions working to prevent young people from becoming radicalised to the point of committing a criminal offence, and/or that offer a viable alternative to criminal justice intervention for young offenders, therefore have increasing importance, particularly as young people make up an increasing proportion of the caseloads of CVE programmes delivered outside of the criminal justice context (Bronsard et al. 2022; Home Office 2023). Programmes delivered in community or other non‐criminal contexts are likely to attract less stigma and may also be perceived as more legitimate by youth than often mandated programming delivered in the criminal justice system (Ellis and Dalaine 2022; Global Counterterrorism Forum [GCTF] 2015; Kessels 2017).

Understanding the effectiveness of these programmes is therefore crucial, particularly as previous research has identified a lack of evidence relating to the effectiveness and implementation of CVE interventions working with youth (Cherney et al. 2022a).

### 2.2 The Intervention

#### 2.2.1 Interventions for Countering Cognitive and Behavioural Radicalisation

CVE interventions working with children and adolescents range from more universal *primary* interventions that work to build resilience to radicalisation amongst broader groups of youth, through to more targeted *secondary* and *tertiary* interventions (Elshimi 2020). This review specifically focuses on secondary and tertiary interventions and excludes primary interventions due to their broader focus on delivering outcomes that are often only indirectly related to countering cognitive and behavioural radicalisation, and because a comprehensive review of these interventions has already been completed (Wolfowicz et al. 2022). This review also specifically focuses on targeted interventions delivered outside the criminal justice system as defined in the next section.

Secondary interventions work to interrupt the radicalisation of those identified as being at risk of radicalisation or already on a radicalisation pathway based on indicators of vulnerability, or views and behaviours indicative of cognitive or behavioural radicalisation (Elshimi 2020). Youth‐focused secondary interventions vary in form and focus, ranging from fixed‐length and lighter‐touch educational programmes that work with pupils exhibiting problematic or extremist views (Cifuentes et al. 2013; Skiple 2020) through to more intensive case management (Lewis, Marsden, Cherney, et al. 2024) and mentorship approaches (e.g., Fisher et al. 2020) that use more individualised approaches to design and deliver tailored packages of multidisciplinary support. Tertiary interventions work to promote the deradicalisation and/or disengagement (Elshimi 2020) of individuals who have become radicalised, as evidenced by, for example, them actively supporting or engaging in violent extremist activities (Vergani et al. 2020). Owing to clients of tertiary interventions being at a later stage of radicalisation, these interventions commonly use more intensive case management or mentorship approaches (e.g., AEF 2018; Christensen 2015).

Importantly, the line between secondary and tertiary interventions is not absolute, and many programmes span both secondary and tertiary prevention by working with both at‐risk and radicalised clients (e.g., AEF 2018; Cherney 2024). Several secondary and tertiary interventions are specifically designed for youth cohorts (e.g., Sahgal and Kimaiyo 2020), whilst others work with children and adolescents as well as older clients (e.g., Bronsard et al. 2022; Home Office 2023), although, as noted above, younger age groups now make up an increasing proportion of these broader caseloads.

#### 2.2.2 Interventions Delivered Outside of the Criminal Justice System

High levels of cooperation between criminal justice agencies and other partners make it difficult to delineate the boundaries of the criminal justice system in different fields of practice (Levin 2023). The CVE field is no different, and high levels of multi‐agency collaboration between the police and other professionals (Mazerolle et al. 2021) complicates efforts to define what is (and is not) a criminal justice intervention. A particular challenge in the context of this review is that this type of multi‐agency partnership underpins many interventions that work with young people *before* they have committed any crime (e.g., Ellis et al. 2022; Ottis 2016). Although previous authors have defined criminal justice interventions as those interventions that partner with at least one criminal justice agency (e.g., Sydes et al. 2022), such a definition – whilst helpful – would have meant having to exclude interventions that primarily operate outside of the criminal justice system, but which have some police involvement. For example, the police are a key partner in the UK Channel programme, but the vast majority of Channel cases are individuals who have not committed (and who are not under investigation for) a relevant criminal offence (HM Government 2023). This review therefore restricts its definition of a criminal justice intervention as an intervention delivered *by* criminal justice agencies (e.g., the police, correctional, or probation staff) and/or *through* the criminal justice system to individuals prosecuted and convicted of relevant offences (e.g., in correctional, custodial, or probation contexts). This definition allows for the inclusion of a broader range of relevant interventions that work to divert young people away from the criminal justice system – including those that might partner with the police in some way.

Most interventions that operate outside of the criminal justice system are likely to represent some form of secondary prevention, as active support for or participation in violent extremist activities as defined above is likely to trigger a criminal justice response. However, some tertiary interventions are also delivered outside of the criminal justice system, for example, EXIT programmes in Europe are delivered by civil society organisations, and work to promote disengagement from extremist groups (e.g., Christensen 2015). Both secondary and tertiary interventions can therefore be defined as non‐criminal justice interventions, provided they work to prevent or divert young people away from arrest, prosecution, and imprisonment, or are offered to young people as an alternative to criminal proceedings by the authorities. Examples of relevant interventions might therefore include programmes working with at‐risk or radicalised young people *completely independently* of the criminal justice system; interventions engaging with youth at risk of radicalisation *before* they have committed or have been investigated for a violent extremist or terrorism offence; and interventions *diverting* individuals who have been investigated or arrested for an offence away from the criminal justice system.

### 2.3 How the Intervention Might Work

There is no single type of secondary or tertiary intervention, nor a single profile of an at‐risk or radicalised young person (Horgan 2008). However, these interventions commonly aim to counter cognitive and/or behavioural radicalisation by targeting risk factors and/or leveraging protective factors (Wolfowicz et al. 2022). Although adherence to specific programme logics is rarely made explicit (Lewis, Marsden, Cherney, et al. 2024), interventions may adopt a strengths‐based approach that is more focused on leveraging and building strengths and skills associated with desistance from, or resilience to, violent extremism (Marsden 2017); may be more risk‐oriented, and focus primarily on managing and mitigating risk factors (Lewis, Marsden, Cherney, et al. 2024); or may reflect and integrate aspects of both approaches. As noted above, programmes may vary as to whether they take a more individualised approach and target risk and protective factors specific to individual clients (Lewis, Marsden, Cherney, et al. 2024) or deliver a set curriculum or structure that explores issues seen to be relevant across different cases (Cifuentes et al. 2013; Skiple 2020). Depending on the programme, clients may be provided with single or multiple forms of support, including, for example, educational, psychological, ideological and vocational activities (Cherney and Belton 2021). Both one‐to‐one and group‐based models of delivery might also be used.

Most programmes work directly with the at‐risk or radicalised young person. However, other programmes seek to intervene indirectly by working with parents or other people in the ‘social environment’ to empower them to intervene in the radicalisation of an ‘index client’ (Uhlmann 2017), that is, an at‐risk or radicalised child or adolescent (Haugstvedt 2022). However, the distinction between these two approaches is not absolute, and both types of programmes will often work with both the young person and their families. This reflects the fact that many programmes use socio‐ecological models of prevention that seek to tackle risk factors and leverage protective factors at the individual, familial, social, and community levels (Ellis et al. 2022; Cherney et al. 2022a).

The fundamental logic of secondary and tertiary prevention work – that radicalisation is best tackled through activities that target relevant risk factors and build protective factors – underpins work with all intervention clients, regardless of age. This is reflected in the fact that a significant amount of CVE work with youth is delivered through programmes that are not youth‐specific, as noted above. However, the importance of practitioners and programmes working in age‐appropriate ways and tailoring their approach to take account of the specific needs of young cohorts is increasingly reflected in the literature (Barracosa and March 2022; Cherney et al. 2022a). Although there is no single process through which (young) people become involved in violent extremism (Horgan 2008), research points to important differences in processes of youth and adult radicalisation, for example in regard to the risk and protective factors that are more prevalent (Oppetit et al. 2019), as well as how developmental periods of childhood and adolescence can shape journeys towards extremism in ways that are relevant for intervention work (Beelmann 2021). Taking account of these developmental differences, including considering how engagement with violent extremist ideologies (Rousseau et al. 2024; Koehler 2020) and with CVE interventions might impact children and adolescents differently, is important.

### 2.4 Why It Is Important to Do This Review

Recent trends in radicalisation raise pressing questions relating to the efficacy of current approaches that work to prevent and counter the radicalisation of children and adolescents. As noted above, interventions that work to prevent young people from extremist offending and/or divert them away from the criminal justice system are important, both from a public safety standpoint and for protecting young people from the harms produced by radicalisation. This review, therefore, aims to support policymakers and other stakeholders by addressing key evidence gaps identified in previous, related reviews, most notably by Cherney et al. (2022a), who noted a lack of evidence relating to the implementation and effectiveness of youth‐focused CVE work.

This review aims to examine the effectiveness and implementation of relevant programmes to identify approaches that might be usefully transferred into other contexts, and to identify those implementation factors and moderators that should be considered when designing and delivering interventions. In doing so, it aims to build on previous reviews that have examined youth‐focused CVE interventions (Cherney et al. 2022a; Wallner 2021; White 2021). These important reviews have identified significant, relevant insights, but do not specifically focus on secondary and tertiary interventions delivered outside of the criminal justice system. Although extensive, they also do not fully conform to the methodological approach set out by the Campbell Collaboration, both in terms of the comprehensiveness of the search strategy and the inclusion criteria used.

Because these reviews specifically focus on youth‐focused CVE work, they were not designed to incorporate the full range of insights drawn from broader CVE interventions that are not age‐specific. They are also focused on ‘youth’ which, as we discuss in detail below, spans a greater age range than common understandings of childhood and adolescence, which are the specific focus of this review. This review also aims to build on systematic reviews that have examined relevant interventions (e.g., Lewis, Marsden, Cherney, et al. 2024; Hassan et al. 2021a, 2021b) but which do not specifically draw out findings relevant to youth. Although the broader findings presented in these reviews are likely to be relevant, it is important to determine whether studies included in previous reviews present evidence specifically relating to working with younger cohorts, as it cannot be assumed that insights relating to adults can be transferred to work with children and adolescents (Cherney et al. 2022a).

The analysis of implementation will make a particularly important contribution as only a small number of systematic reviews have examined whether interventions are being implemented in ways that are likely to produce positive outcomes, none of which are focused on work with children and adolescents (e.g., Lewis, Marsden, Cherney et al. 2024; Mazerolle et al. 2021). This dual focus on examining implementation and effectiveness also helps to overcome known weaknesses in the broader evidence base underpinning CVE interventions, and the noted absence of all but a small number of studies using stronger quasi‐experimental or experimental research designs needed to produce robust evidence of intervention effectiveness. This review faces similar challenges to our previous review of case management interventions, which was unable to find any eligible impact evaluations (Lewis, Marsden, Cherney, et al. 2024), making it particularly important to capture evidence that speaks to whether and how programmes are being implemented effectively. Although evidence relating to implementation cannot be used to determine whether an intervention is effective, it can provide relevant insights into whether a programme is *likely* to be effective. By elucidating such insights, we therefore aim to build on a growing – but still modest – body of evidence that has examined the processes and mechanisms through which interventions function to overcome some of the ongoing challenges associated with using quasi‐experimental or experimental designs in this space (Lewis, Marsden, Cherney, et al. 2024).

## 3 Objectives

This review aims to answer the three research questions and objectives listed in Table 1.

**Table 1:** Research questions and objectives.

RQ	Question	Objective
1	Are secondary and tertiary interventions delivered outside of the criminal justice system effective at countering the cognitive and behavioural radicalisation of children and adolescents?	Synthesise evidence relating to relevant primary and secondary outcomes of effectiveness.
2	Are secondary and tertiary interventions delivered outside of the criminal justice system being implemented as intended?	Synthesise evidence that captures how interventions are implemented, considering whether they are implemented as expected or in ways that align with their underlying logic.
3	What factors influence how interventions working with children and adolescents outside of the criminal justice system are implemented?	Identify those implementation factors (facilitators and barriers) and moderators that impact how interventions are delivered.

## 4 Methods

### 4.1 Criteria for Considering Studies For This Review

#### 4.1.1 Types of Studies

##### 4.1.1.1 Evaluating Effectiveness (Objective I)

Eligibility for inclusion in the analysis of effectiveness was limited to quantitative studies using experimental or stronger quasi‐experimental designs identified in previous reviews (Lewis, Marsden, Cherney, et al. 2024; Mazerolle et al. 2021). Only those designs that allowed for comparison between a treatment and a reasonable comparison group (e.g., matched control group, control group with face validity) were included. Adapting a list presented in Mazerolle et al. (2021), eligible designs therefore included:

– Randomised controlled trials.
– Cross‐over designs (randomised and non‐randomised).
– Propensity or statistically matched control group designs (with or without baseline).
– Unmatched control group designs without a baseline where the control group has face validity.
– Unmatched control group designs with pre‐post intervention measures allow for difference‐in‐difference analysis.
– Short interrupted time‐series designs with a control group (less than 25 pre‐and post‐intervention observations).
– Long interrupted time‐series designs with or without a control group (over 25 pre‐and post‐intervention observations).

No restrictions were set on eligible comparator conditions; therefore, studies using waitlist, treatment‐as‐usual, and alternative treatment conditions were eligible for inclusion. In the event that meta‐analysis had been possible, the comparator type would have been coded and analysed as a moderator. However, as discussed in more detail below, meta‐analysis was not possible as we only identified one eligible quasi‐experimental study.

##### 4.1.1.2 Evaluating Implementation (Objectives II and III)

Quantitative studies using experimental, quasi‐experimental and non‐experimental designs; qualitative studies; and studies using mixed methods designs were eligible for inclusion in the analysis of implementation. In deciding to include this broad range of research designs, we were guided by the approach used by Higginson et al. (2015), who included a similar variety of designs so as to ‘capture the broadest range of evidence that assesses the reasons for implementation success or failure’ (p. 22).

#### 4.1.2 Types of Participants

This review focuses on children and adolescents as opposed to the broader construct of ‘youth’ that is commonly understood as extending beyond adolescence.^2^ This reflects the distinctiveness of childhood and adolescence as key stages of development, and a growing recognition that interventions must be cognisant of developmental processes when working with younger cohorts (Beelmann 2021). Although there is debate as to the age at which adolescence ends (Sawyer 2018), this review was guided by the World Health Organisation definition of adolescence as ages 10–19 years (WHO n.d.), and therefore defined children and adolescents as those aged 19 years or under.

Studies reporting on interventions working to prevent or counter the radicalisation of at‐risk (secondary) or radicalised (tertiary) individuals aged 19 years and under were therefore eligible for inclusion in the review. This included interventions working directly with children and adolescents, as well as indirect forms of prevention that, as described above, primarily worked with the families or other people within the ‘social environment’ of an at‐risk or radicalised young person so as to intervene in their radicalisation. Eligible interventions, therefore, fell into one or more of the following categories:

– Those working with clients explicitly described as being at risk of being radicalised or already at an early stage of radicalisation and/or who have been assessed as such according to a specified set of criteria, for example, using a particular risk assessment tool, or based on their association with radicalised individuals.
– Those working with clients who have been cognitively or behaviourally radicalised, as evidenced by, for example, active support for a violent extremist organisation or through a conviction for a relevant extremist offence.
– Those working with family members or other contacts of at‐risk or radicalised young people aged 19 years or under (as defined above), when the focus is to prevent or counter the radicalisation of the young person in question.

There were no additional socio‐demographic exclusion criteria.

Although focused specifically on children and adolescents as defined above, studies reporting on interventions that worked with ‘youth’ (or ‘young people’ or similar) with no specification of participant age were also eligible for inclusion, following guidance from the Campbell Crime and Justice Steering Committee. Although, as noted above, youth is a broader construct that does not always neatly map onto our phenomena of interest, such studies are likely to have relevant insights owing to the fact that studies reporting on ‘youth’ interventions often work with relevant age groups (e.g., Kolbe 2019). Notable differences between interventions working with youth and those focused on children and adolescents are discussed.

Eligible interventions included those working exclusively with clients aged 19 years or under, and those working with this cohort as part of broader caseloads. This included youth‐focused programmes working with clients aged over 19 years of age (or that did not specify the age range of its clients); or interventions that were not age‐specific (i.e., that also worked with adults). Different eligibility criteria were used to assess whether broader studies should be included in the analysis of effectiveness and implementation:

– Effectiveness: Outcome data specific to children, adolescents, or youth (or young people) had to be presented in the study or available from the study author(s), with data specific to clients aged 19 years or under isolated wherever possible.
– Implementation: Studies had to meet at least one of two conditions: (1) at least half of the intervention caseload was aged 19 years or under; (2) findings specifically relating to children, adolescents, or youth (however defined) were presented.

#### 4.1.3 Types of Interventions

Studies examining any type of secondary or tertiary intervention working to prevent the cognitive and behavioural radicalisation of children and adolescents were eligible for inclusion. Both direct and indirect models of prevention were eligible for inclusion. Direct models refer to a broad range of interventions such as case management (Lewis, Marsden, Cherney, et al. 2024), educational (Skiple 2020), or mentoring programmes (Christensen 2015) that work directly with at‐risk and/or radicalised youth in one‐to‐one or group settings. Indirect models are interventions that work with families or other contacts of at‐risk or radicalised youth to help them intervene in a young person's radicalisation (Haugstvedt 2022). Although targeted interventions working to empower families or others to intervene in a person's radicalisation process were eligible for inclusion (i.e., an indirect approach), broader capacity‐building and training interventions were not included as they did not aim to prevent or counter the radicalisation of a *specific* radicalised or at‐risk young person.

Interventions that worked to counter cognitions and behaviours associated with any form of violent extremism, as defined by US government agencies, were eligible for inclusion in the review. This included both dedicated CVE interventions and interventions delivering CVE work alongside other functions. This included cognitions and behaviours associated with the different categories of domestic violent extremism used by DHS and the Federal Bureau of Investigation (FBI),^3^ as well as international forms of violent extremism, such as those associated with designated Foreign Terrorist Organisations.^4^ Eligibility against this criterion was determined by assessing whether the description of an intervention's aims and objectives and/or any outcomes used to evaluate it by study authors were relevant to countering these different types of violent extremist beliefs and behaviours.

To be considered a secondary intervention, the intervention had to be described as such and/or as working with individuals identified as being at risk of, or vulnerable to, radicalisation. When determining whether a programme worked with at‐risk clients, we were primarily guided by the descriptions of the interventions and interventions that clients provided by the study authors. However, as we discuss in Section 4.4, several programmes that purported to work with at‐risk youth were often not sufficiently targeted in the way described here and were therefore excluded.

When determining whether an intervention worked with radicalised individuals, we were again guided by the descriptions of interventions and clients provided by authors. To be considered a tertiary intervention, interventions had to be described as such (or as something similar, such as a deradicalisation or disengagement programme) or as specifically working with clients who, based on the description provided, could be considered cognitively or behaviourally radicalised (e.g., ‘violent extremists’).

Broader studies of CVE work with children and adolescents (as defined above) were also eligible for inclusion when it was possible to determine that the activities being examined were relevant to secondary and/or tertiary prevention. Studies that spanned all levels of CVE prevention (i.e., primary, secondary, and tertiary) were also eligible for inclusion when it was possible to isolate findings related to relevant secondary or tertiary activities. When this was not possible, the study was excluded.

As outlined in the protocol (Lewis et al. 2025), our definition of CVE interventions also extended to include larger Disarmament, Demobilisation and Reintegration (DDR) programmes that worked to promote the disengagement of ‘violent extremists’ (e.g., Khalil et al. 2019) or with former members of groups designated as terrorist organisations by the United States (Khalil et al. 2019; Richards 2017). However, no studies reporting on these programmes were ultimately eligible for inclusion in the review.

#### 4.1.4 Types of Outcome Measures

##### 4.1.4.1 Outcomes Relating to Effectiveness (Objective II)

Studies reporting on relevant primary and/or secondary outcomes were eligible for inclusion. In defining relevant outcomes, we were guided by a typology of radicalisation outcomes used by Wolfowicz et al. (2021) in their Campbell Collaboration systematic review that distinguished between radical attitudes, intentions, and behaviours.

Following the definition of radicalisation presented earlier, we adapted this typology to focus on violent extremist outcomes and identified three categories of eligible outcomes:

– Violent extremist attitudes (i.e., the impact of an intervention on clients' support/sympathy for a violent extremist or terrorist cause, movement, or organisation).
– Violent extremist intentions (i.e., the impact of an intervention on clients' willingness or stated intention to engage in violent extremist action, such as joining a violent extremist or proscribed terrorist organisation).
– Violent extremist behaviours: (i.e., the impact of an intervention in preventing engagement in violent extremism, as evidenced by, for example, a client's disengagement and desistence from an extremist or terrorist group).

Secondary outcomes were similarly defined as the risk and protective factors that Wolfowicz et al. (2021) identified as having a significant impact on one or more of these radicalisation outcomes. Studies were therefore eligible when they reported measuring an outcome relating to one or more of these factors, provided that the author(s) specifically identified these outcomes as relevant to preventing cognitive and/or behavioural radicalisation (Table 2).

**Table 2:** Example secondary outcomes (based on Wolfowicz et al. 2021).

Domain	Examples
Socio‐demographic/Background	*Risk factors* Unemployment, alcohol or substance abuse, relationship problems, etc. *Protective factors* Socio‐economic status, education, etc.
Attitudinal^a^	*Risk factors* Perceived in‐group superiority, perceived discrimination, perceived injustice, etc. *Protective factors* Law abidance, belief in legitimacy of law, trust in institutions, trust in others, etc.
Psychological/Personality	*Risk factors* Mental health issues, anger, negative affect, authoritarianism, etc. *Protective factors* Life satisfaction, higher self‐esteem, etc.
Experiential	*Risk factors* Family violence, personal strain, etc. *Protective factors* High perceptions of procedural justice.
Criminogenic	*Risk factors* Deviant/radical peers, low self‐control, thrill seeking, etc. *Protective factors* Parental involvement, school bonding, outgroup friends, etc.

^a^ This category is distinct from the primary outcome of ‘attitudes’ as it relates to attitudes that are linked to higher or lower risks of cognitive or behavioural radicalisation and not attitudes indicative of radicalisation.

No exclusion criteria were set on the methods or tools used to capture outcome data. Eligible data collection methods including primary research with practitioners, stakeholders, at‐risk and radicalised young people, family members, or peers (e.g., surveys, interviews, focus groups); secondary analysis of case data held by programmes (e.g., information held on case management systems, such as case note data); and data relating to (re)engagement in violent extremism (e.g., arrest data or evidence that a client had subsequently joined a violent extremist organisation after leaving an intervention).

##### 4.1.4.2 Outcomes Relating to Implementation (Objectives II and III)

The review did not set any exclusion criteria relating to implementation outcomes, following the approach used in previous Campbell Collaboration reviews (Lewis, Marsden, Cherney, et al. 2024; Mazerolle et al. 2021). However, all studies included in the analysis of implementation had to report on interventions that aimed to deliver primary or secondary outcomes relevant to cognitive and behavioural radicalisation as defined above.

Studies were included in the analysis for Objective II when they assessed whether an intervention was implemented as intended, including whether it was implemented in line with a theory of change or programme logic (Lewis, Marsden, Cherney, et al. 2024). Studies were included in the analysis of Objective III when they identified implementation factors and/or moderators that impacted how interventions were implemented. Following our previous review, we defined implementation factors as ‘actions or actors necessary to successfully install and maintain an intervention’ (Thornton et al. 2019, 267). This included implementation factors that were seen to facilitate the work of practitioners and programmes, and those that were a barrier to implementation (Cherney et al. 2020). Examples of relevant implementation factors identified in our previous review included partnership working, the availability of relevant knowledge and expertise in intervention teams, and tailoring services and delivery to individual clients (Lewis, Marsden, Cherney, et al. 2024).

Moderators were also defined in line with our previous review as the ‘contextual conditions’ or the ‘features of the people or places that are the target for intervention’ (Thornton et al. 2019, 267). Examples of moderators known to impact the delivery of CVE interventions based on our previous review of case management include local contexts and delivery contexts, including whether programmes are delivered in criminal justice or community settings, which is pertinent to this review (Lewis, Marsden, Cherney, et al. 2024).

##### 4.1.4.3 Duration of Follow‐Up

No restrictions were set on the length of the follow‐up period.

#### 4.1.5 Types of Settings

Interventions delivered in a broad range of settings, including community, clinical, educational, or social work settings, were eligible for inclusion if they fell into one of the following categories that were used to identify non‐criminal justice interventions:

– Worked with individuals before any criminal investigation or arrest.
– Provided as an alternative to arrest, charge, or imprisonment to individuals who were/had previously been subject to a criminal investigation or arrest.
– Worked with individuals who had been or were subject to a criminal investigation, arrest, prosecution, and/or sentence, but which operated independently from criminal justice agencies or the criminal justice system.

Interventions that were delivered to individuals detained in correctional or custodial contexts, or in other contexts that involved some apparent restriction on movement, deprivation of liberty, and/or military involvement (as described in Section 4.4), were excluded from the review. So too were any interventions delivered as part of post‐release conditions, for example, in probation contexts.

However, as discussed earlier, the involvement of criminal justice agencies in isolation was not used as a reason for exclusion because these agencies – and the police in particular – are often key partners of interventions that operate outside of the criminal justice system (Lewis, Marsden, Cherney, et al. 2024). Interventions with a police partner were therefore still eligible for inclusion when they fell into one of the three categories listed above. The only exception was interventions that involved a criminal justice agency working directly with clients. No other restrictions were placed on the type of delivery agents.

#### 4.1.6 Publication Types

Peer‐reviewed and non‐peer‐reviewed academic and grey literature were included. Although no such studies were identified, protocols and trial registries for relevant ongoing studies with no available outputs would have been included in a separate ‘References to ongoing studies’ section as described in the protocol (Lewis et al. 2025).

#### 4.1.7 Countries

There were no geographical exclusion criteria.

#### 4.1.8 Languages

Only research published in English was included.

#### 4.1.9 Date of Publication

Studies published after January 2000 were eligible for inclusion. Whilst some relevant interventions have a longer history, the policy framework of CVE only really emerged at the start of the 21st century (Neumann 2013).

### 4.2 Search Methods for Identification of Studies

A multi‐stage search methodology was used, comprising:

– Electronic searches of academic platforms (Table 3).
– Hand searches of key journals (Table 4), grey literature (Table 5), and clinical trial repositories (Table 6).
– Bibliographic searches of related synthesis papers (Table 7).
– Consulting with experts.
– Forward and backward citation searching of eligible studies.

**Table 3:** Academic databases and platforms.

Criminal Justice Abstracts	EBSCO
Cumulative Index to Nursing and Allied Health Literature (CINAHL)	EBSCO
International Policial Science Abstracts	EBSCO
Scopus	Elsevier
Epistemonikos	Epistemonikos
Australian Criminology Database (CINCH)	Informit
APA PyscINFO	OVID
MEDLINE	OVID
Joanna Briggs Institute EBP Database	OVID
PsycEXTRA	OVID
Applied Social Sciences Index & Abstracts (ASSIA)	ProQuest
Dissertations & Theses Global	ProQuest
Educational Resources Information Center (ERIC)	ProQuest
International Bibliography of the Social Sciences (IBSS)	ProQuest
Social Services Abstracts	ProQuest
Sociological Abstracts	ProQuest
Conference Proceedings Citation Index – Social Sciences & Humanities (CPCI‐SSH)	Web of Science
Emerging Sources Citation Index (ESCI)	Web of Science
Social Science Citation Index (SSCI)	Web of Science
Book Citation Index – Social Sciences & Humanities	Web of Science

**Table 4:** Key journals.

Journal name
*Terrorism and Political Violence*
*Studies in Conflict & Terrorism*
*Behavioral Sciences of Terrorism and Political Aggression*
*Critical Studies on Terrorism*
*Journal for Deradicalization*
*Perspectives on Terrorism*
*International Journal of Conflict & Violence*
*Dynamics of Asymmetric Conflict*
*Journal of Policing, Intelligence & Counter Terrorism*
*Journal of Threat Assessment and Management*
*Childhood*
*Children & Society*
*Journal of Child and Family Studies*
*Journal of Youth and Adolescence*
*Vulnerable Children and Youth Studies*
*Journal of Children's Services*
*Child & Family Social Work*

**Table 5:** Clinical trial registries.

Source
Clinical Trials Results
NIH RePORTER
Trials Register of Promoting Health Interventions (TRoPHI)
Unreported Trials Register
UK Clinical Research Network (UKCRN Study Portfolio)
WHO International Clinical Trials Registry Platform

**Table 6:** Grey literature sources.

Source	Link
Research centres and institutes	
Addressing Violent Extremism and Radicalisation to Terrorism Network (AVERT)	https://www.avert.net.au
Australian Institute of Criminology (AIS)	https://www.aic.gov.au
Centre for Research & Evidence on Security Threats (CREST)	https://crestresearch.ac.uk
Canadian Practitioners Network for the Prevention of Radicalization & Extremist Violence (CPN‐PREV)	https://cpnprev.ca
Danish Institute for International Studies (DIIS)	https://www.diis.dk/en
European Commission Radicalisation Awareness Network (RAN)^a^	https://home-affairs.ec.europa.eu/networks/radicalisation-awareness-network-ran_en
Global Center on Cooperative Security	https://globalcenter.org
Hedayah	https://hedayah.com
Institute for Strategic Dialogue (ISD)	https://www.isdglobal.org
International Centre for Counter‐Terrorism (ICCT)	https://www.icct.nl
International Centre for the Study of Radicalisation (ICSR)	https://icsr.info
International Center for the Study of Violent Extremism (ICSVE)	https://icsve.org
Polarization and Extremism Research and Innovation Lab (PERIL)	https://perilresearch.com
National Consortium for the Study of Terrorism & Responses to Terrorism (START)	https://www.start.umd.edu
PrEval	https://preval.hsfk.de/en/
The PREV‐IMPACT Canada Project	https://prev-impact.ca/en
RAND	https://www.rand.org
Resolve Network	https://www.resolvenet.org
Royal United Services Institute (RUSI)	https://rusi.org
VOX‐Pol	https://voxpol.eu
Non‐governmental organisations	
Save the Children	https://www.savethechildren.org.uk
Search for Common Ground	https://www.sfcg.org
Violence Prevention Network	https://violence-prevention-network.com
War Child	https://www.warchild.org.uk
Youth Endowment Fund (YEF)	https://youthendowmentfund.org.uk
Research repositories	
Blueprints for Healthy Youth Development	https://www.blueprintsprograms.org
Crime Reduction Toolkit	https://www.college.police.uk/research/crime-reduction-toolkit
CrimeSolutions	https://crimesolutions.ojp.gov
CVE Reference Guide for Local Organisations	https://www.cvereferenceguide.org/en
Radicalisation Research	https://radicalisationresearch.org
National and international government agencies	
Australian Department of Home Affairs	https://www.homeaffairs.gov.au
Ministry of Foreign Affairs of the Netherlands	https://www.government.nl/ministries/ministry-of-foreign-affairs
Netherlands National Coordinator for Security and Counterterrorism	https://english.nctv.nl
New Zealand Department of the Prime Minister & Cabinet	https://www.dpmc.govt.nz
Organisation for Security & Co‐Operation in Europe	https://www.osce.org
Public Safety Canada	https://www.publicsafety.gc.ca/index-en.aspx
Royal Canadian Mounted Police	https://www.rcmp-grc.gc.ca
The Swedish Center for Countering Violent Extremism	https://cve.se
United Nations Educational, Scientific & Cultural Organisation	https://www.unesco.org/en
United Nations Development Programme	https://www.undp.org
United Nations Office for Drugs & Crime	https://www.unodc.org
United Nations Office for Counter‐Terrorism	https://www.un.org/counterterrorism/
United Nations Counter‐Terrorism Executive Directorate (UNCTED)	https://www.un.org/securitycouncil/ctc/
United Nations International Children's Emergency Fund (UNICEF)	https://www.unicef.org
USAID	https://www.usaid.gov
US Department of Homeland Security	https://www.dhs.gov
US National Institute of Justice	https://nij.ojp.gov
US Office of Juvenile Justice and Delinquency Prevention	https://ojjdp.ojp.gov
UK Home Office	https://www.gov.uk/government/organisations/home-office
UK Ministry of Justice	https://www.gov.uk/government/organisations/ministry-of-justice

^a^ A synthesis paper reporting on various relevant projects was also reviewed: European Commission (2022) EU‐funded projects on preventing radicalisation: Synergies and insights.

**Table 7:** Review articles.

Source
Cherney, A., K. De Rooy, and R. Williams. 2022a. “An Evidence Review of Strategies Targeting Youth Who Have Radicalised to Violent Extremism.” *Journal for Deradicalization* 33: 40–69.
Hassan, G., S. Brouillette‐Alarie, S. Ousman, &QJ14;et al. 2021a. *A Systematic Review on the Outcomes of Primary and Secondary Prevention Programs in the Field of Violent Radicalization*. CPN‐PREV.
Hassan, G., S. Brouillette‐Alarie, S. Ousman, &QJ14;et al. 2021b. *A Systematic Review on the Outcomes of Tertiary Prevention Programs in the Field of Violent Radicalization*. CPN‐PREV.
Madriaza, P., D. Morin, G. Hassan, et al. 2022. *Evaluating Programs for Preventing Violent Extremism: A Systematic Methodological Review*.
Morrison, J. F., A. Silke, H. Maiberg, C. Slay, and R. Stewart. 2021. *A Systematic Review of Post‐2017 Research on Disengagement and Deradicalisation*. CREST.
Pistone, I., E. Eriksson, U. Beckman, C. Mattson, and &QJ0;M. Sager. 2019. “A Scoping Review of Interventions for Preventing and Countering Violent Extremism: Current Status and Implications for Future Research.” *Journal for Deradicalization* 19: 1–84.
Wallner, C. 2021. *The Contested Relationship Between Youth and Violent Extremism: Assessing the Evidence Base in Relation to P/CVE Interventions*. RUSI.
White, J. 2021. *Interventions Targeting Youth Engagement: A Systematic Literature Review of Effectiveness of Counter‐Terrorism and Preventing and Countering Violent Extremism Activities*. Ministry of Foreign Affairs of the Netherlands.
Wolfowicz, M., D. Weisburd, and B. Hasisi. 2022. *Counter‐Radicalization Interventions: A Review of the Evidence*. Institute for Futures Studies.

#### 4.2.1 Electronic Searches

The platforms and databases listed in Table 3 were searched using search strategies developed by an information retrieval specialist. The full search strategies are included in the appendices for reference. The search strategies were developed through an iterative process of testing as described in more detail in the protocol (Lewis et al. 2025).

#### 4.2.2 Searching Other Resources

Supplementary electronic sources were also searched: core journals relating to terrorism and CVE and to childhood and adolescence (Table 4); clinical trial repositories (Table 5); and relevant grey literature sources, including research repositories and institutions and organisations involved in researching or delivering relevant interventions (Table 6); the conventions for searching these resources are outlined in detail in the protocol (Lewis et al. 2025).

#### 4.2.3 Bibliographic Searches

The bibliographies of published, relevant Campbell Collaboration reviews funded through the Five Research and Development (5RD) Countering Violent Extremism programme were searched in August and September 2024 (Carthy et al. 2020; Lewis, Marsden, Cherney, et al. 2024; Mazerolle, Eggins, et al. 2020; Mazerolle et al. 2021; Sydes et al. 2022; Windisch et al. 2022). When reviewing our previous review (Lewis, Marsden, Cherney, et al. 2024), we also used keywords (e.g., ‘youth’) to search the database of studies that were excluded at the full‐text stage. The bibliographies of other (i.e., non‐Campbell) review articles reporting on related interventions were also reviewed, with a focus on those published in the 5 years before the date of the search (Table 7).

#### 4.2.4 Consulting With Experts

Eight researchers who have conducted research on relevant topics were contacted upon completion of the final searches to identify other relevant studies and/or to identify full texts of grey literature evaluations that we were unable to locate online.

#### 4.2.5 Forward and Backward Citation Searches

The bibliographies of all studies included after full‐text screening (see below) were reviewed to identify any additional, potentially eligible studies. Forward citation searches of all included studies were also conducted in Google Scholar. These forward and backward citation searches were completed until saturation was reached (i.e., when no new, eligible studies were identified).

### 4.3 Data Collection and Analysis

#### 4.3.1 Selection of Studies

Studies were screened using a staged process slightly adapted from the process outlined in the protocol, as discussed in more detail in Section 4.4. Search results were initially de‐duplicated in EndNote (Version 21) (The Endnote Team 2013) and uploaded into the Covidence online platform.^5^ A second stage of de‐duplication was completed within Covidence, whereby one of the lead reviewers (J. L.) ensured that only genuine duplicates had been excluded. The remaining records progressed to title and abstract screening.

Two reviewers were trained to use the title/abstract screening tool (see Appendix SII) before these two reviewers, and the lead author (J. L.) provisionally reviewed the first 50 records in Covidence (sorted on relevance). An online meeting was held to discuss any disagreements to ensure that the inclusion/exclusion criteria were fully understood before title and abstract screening. The reviewers then screened all titles and abstracts in duplicate using this same tool. All conflicts were reviewed by J. L., who made the final inclusion/exclusion decision, with input from S. M. when necessary. J. L. also reviewed a random selection of studies (5%) excluded by each reviewer to ensure that neither reviewer was returning a high level of false positives or negatives.

The same two reviewers then began full‐text screening, having been trained how to use the full‐text screening tool (see Appendix SII). Reviewers were asked to include studies when there was some ambiguity around their eligibility (e.g., relating to the boundaries of the criminal justice system), and to flag any clarifications for J. L. to review. To check that the screening tool was supporting consistent decision‐making, J. L. reviewed the first 50 studies for which consensus had been reached. As an additional check, J. L. also assessed the title and abstract of all excluded studies against the full text screening tool. All conflicts were resolved by J. L., with input from S. M. where necessary.

Potentially eligible studies identified by experts and through forward and backward searches were then de‐duplicated against the original corpus before being screened on title and abstract by J. L. All potentially eligible studies were then double‐screened on full text, with S. M. making the final decision on any conflicts.

#### 4.3.2 Data Extraction and Management

Data were extracted using the data extraction tool included in Appendix SIV. This tool was used to capture key information about the intervention focus, activities, and approach (e.g., secondary and/or tertiary intervention; description of client base; delivery context; types of practitioner, etc.); methodological information (e.g., research design; sample details, data collection tools included); data relating to effectiveness, implementation, and/or implementation factors and moderators; and other pertinent information relevant to the analysis. Quantitative data – including any data relevant to calculating effect sizes – were extracted using a Microsoft Excel spreadsheet (see Appendix SIV). Qualitative data relating to implementation were extracted in Microsoft Word. We had initially planned to extract all data in Excel; however, qualitative data could not be easily coded in a way suited for inputting into an Excel workbook.

#### 4.3.3 Assessment of Risk of Bias in Included Studies

Different tools were used when assessing studies relevant to different research objectives, reflecting the fact that different research designs were eligible for inclusion in the analysis of effectiveness (Objective I) and implementation (Objectives II and III).

##### 4.3.3.1 Objective I: Effectiveness

No eligible experimental studies were identified through the searches. Had they been identified, they would have been assessed using the RoBS‐2 tool as outlined in the protocol.

The single quasi‐experimental study eligible for inclusion in the analysis of effectiveness was assessed using the ROBINS‐I tool described in the appendix (Sterne et al. 2016). This tool was used to assess the risk of bias present pre‐intervention, at‐intervention, and post‐intervention. This informed an overall assessment of the study as having a low, moderate, serious, or critical risk of bias (Table 8).

**Table 8:** ROBINS‐I tool.

Assigned risk level	Description
Low risk of bias	Study assessed as having a low risk for all domains
Moderate risk of bias	Study assessed as having low or moderate risk for all domains
Serious risk of bias	Study assessed as having serious risk of bias in at least one domain, but not at critical risk of bias in any domain
Critical risk of bias	Study assessed as having critical risk of bias in at least one domain

##### 4.3.3.2 Objective II and III: Implementation

The other quasi‐experimental study reporting on implementation outcomes was again assessed using the ROBINS‐I tool described above. Non‐experimental quantitative studies were assessed using the Effective Public Health Practice Project (EPHPP) Quality Assessment Tool for Quantitative Studies, also outlined in the appendices.^6^ Studies were assessed as being either ‘strong’, ‘moderate’, or ‘weak’ based on an assessment of study design, analysis, withdrawals and dropouts, data collection, selection bias, intervention integrity, confounders, and blinding.

Qualitative studies were assessed using the Critical Appraisal Skills Programme (CASP) assessment tool presented in the appendices. This tool includes 10 ‘Yes’, ‘No’, or ‘Can't tell’ questions relating to different aspects of study design.^7^ Following the protocol, and replicating the approach of Lewis et al. (2023) and Mazerolle, Cherney, et al. (2020), studies were excluded when the answer to one or both of the following questions was ‘No’ or ‘Can't tell’:

1. Is the research design appropriate to answer the question?
2. Was the sampling strategy appropriate to the aims of the research?

Assessments of risk of bias/study quality are presented in summary tables when describing the included studies and summarised when reporting on findings relating to effectiveness, implementation, and individual implementation factors and moderators.

#### 4.3.4 Measures of Treatment Effect

Primary outcome data from the single study included in the analysis of effectiveness was converted into a standardised effect size, using the odds ratio from 2‐by‐2 contingency table formula as set out in the effect size calculator hosted on the Campbell Collaboration website (Lipsey and Wilson 2001; Wilson 2023):

$odds\;ratio\;=\;or\;=\;\frac{a.d}{b.c}$

#### 4.3.5 Unit of Analysis Issues

No unit of analysis issues were identified – again due to the small number of relevant studies that were identified – so the proposed steps for addressing such issues, as outlined in the protocol, were not required (Lewis et al. 2025). Should such issues arise in any updated review, we will use the processes set out in the protocol.

#### 4.3.6 Criteria for Determination of Independent Findings

No issues relating to dependent quantitative data were identified. The solutions outlined in the protocol (Lewis et al. 2025) were therefore not required. The single quantitative study eligible for the analysis of effectiveness only reported on one relevant outcome. No overlapping quantitative studies were included in the analysis of implementation, and none of the included studies reported on similar outcomes.

Several overlapping qualitative outputs were identified (i.e., multiple records reporting on the same research study). Following the protocol, data were extracted from all dependent records, but the individual study was only counted once.

#### 4.3.7 Dealing With Missing Data

The small number of quantitative studies included in the review did not have any issues relating to missing data. Even if such issues had presented, the fact that meta‐analysis was not possible meant that any missing data would have been unlikely to impact the findings of the review.

#### 4.3.8 Assessment of Heterogeneity

Meta‐analysis or other quantitative synthesis was not possible based on the studies included in the review. The proposed approach for assessing heterogeneity, as outlined in the protocol, was therefore not needed or possible (Lewis et al. 2025).

#### 4.3.9 Assessment of Reporting Biases

It was not possible to examine reporting biases in the way outlined in the protocol (Lewis et al. 2025) because meta‐analysis or other synthesis was not possible. However, the publication status of included studies is summarised in the next section of the review, and any relevant differences between the findings of published and unpublished studies are highlighted in the analysis that follows.

#### 4.3.10 Data Synthesis

Extracted data are presented in three forms in the analysis that follows:

1. Narrative summary of the evidence base;
2. Analysis of quantitative effectiveness data;
3. Synthesis of quantitative and qualitative data relating to implementation.

##### 4.3.10.1 Analysis 1: Narrative Summary of the Evidence Base

Information relating to the different types of interventions included in the review is presented in the form of a typology. This typology categorises interventions that are comparable in regard to their approach, focus, and scope, as reflected by similarities in, for example, their target population; activities; delivery context and/or agents; and intended outcomes. The presentation of each category in this typology is informed by the domains contained within the template for intervention description and replication (TIDieR) (Hoffmann et al. 2014). Information relating to each of the domains contained within this framework is presented wherever possible or relevant.

##### 4.3.10.2 Analysis 2: Analysis of Quantitative Effectiveness Data

Meta‐analysis or other synthesis was not possible, given that only one study was eligible for inclusion in the assessment of effectiveness. The analysis that follows, therefore, presents a standardised effect size for the relevant primary outcome reported in this study following the conventions outlined above. Should additional, eligible studies be identified in any updated review, we will follow the detailed conventions for meta‐analysis outlined in the protocol (Lewis et al. 2025).

##### 4.3.10.3 Analysis 3: Analysis of Implementation Data

Evidence relating to implementation was synthesised using a framework synthesis approach outlined in the protocol, following Booth and Carroll (2015). This involved a process of deductive and inductive coding to identify and categorise sub‐themes relating to Objective II (i.e., whether an intervention was implemented as intended; whether an intervention was able to reach relevant clients; whether an intervention was implemented in line with an underlying logic); and Objective III (i.e., specific implementation factors and moderators). This was an iterative process whereby data were initially coded by J. L., before the individual codes were assessed for internal homogeneity and external heterogeneity (Patton 1990) by J. L. and a second reviewer. Once codes had been finalised, the extracted data were re‐reviewed by J. L. to identify any additional data relevant to specific codes. Although we consider this to be a robust approach, it is important to acknowledge that we did not conduct any test for intra‐ or intercoder reliability.

The analysis also examines how the implementation factors relate to different domains of the RE‐AIM framework (Glasgow et al. 2019): *Reach* (i.e., ability to reach the target population, including clients' willingness to engage); perceived *Effectiveness* (as assessed by study participants); *Adoption* (by staff and organisations); *Implementation* fidelity; and *Maintenance* of over time (i.e., whether an intervention becomes ‘institutionalised’ or is sustained over time). Using the framework in this way helps to illustrate how different implementation factors are relevant.

Where appropriate, differences between studies reporting on different types of intervention – including interventions that work specifically with children, adolescents, or youth, and those that work with broader caseloads – are identified within the analysis.

#### 4.3.11 Subgroup Analysis and Investigation of Heterogeneity

Sub‐group analysis was not conducted because meta‐analysis was not possible. In the event that the review is updated, and other eligible studies are identified, we will follow the conventions set out in the protocol when conducting such analysis (Lewis et al. 2025).

#### 4.3.12 Sensitivity Analysis

Sensitivity analysis was not possible or needed due to only one quasi‐experimental study of effectiveness being included. In any future update, we will follow the processes outlined in the protocol, should additional studies be identified (Lewis et al. 2025).

#### 4.3.13 Treatment of Qualitative Research

As stated above, qualitative research is included in the analysis of implementation only.

#### 4.3.14 Summary of Findings and Assessment of the Certainty of the Evidence

As stated in the protocol (Lewis et al. 2025), the review does not present a summary of findings and assessment of the certainty of the evidence.

### 4.4 Deviations From the Protocol

The original eligibility criteria set out in the protocol were refined in three ways to reflect learning that emerged early in the full‐text screening process. First, although we had planned to rely on the descriptions of interventions and clients provided by study authors when identifying secondary interventions, we adapted our approach owing to the inconsistency with how ‘at‐risk’ individuals were identified and defined in different countries and contexts. This difference was particularly pronounced when reading across the Global North and South, as a number of interventions delivered in the latter defined risk at the community or collective level. This is different from dominant understandings of secondary prevention in the Global North, where risk is defined at the individual level. For consistency, we therefore only defined interventions as secondary prevention when they were sufficiently targeted at the individual level, as discussed in detail in Section 5.1.3.

A related challenge was the inconsistency with which ‘youth’ was defined in different contexts. Because it is important that youth work is oriented around ‘contextually and culturally relevant definitions of youth’ (Wallner 2021, 4), studies that specifically worked with ‘youth’ were eligible for inclusion in the review. However, one study evaluating an intervention that worked with youth beneficiaries aged 19–35 was excluded as it was evident that the study findings had limited relevance to children and adolescents as defined above (i.e., aged 19 or under) (Tropp et al. 2019).

A second, related deviation reflected similar challenges in reading across different contexts when delineating the boundaries of the criminal justice system. Several programmes ‐ particularly those operating in conflict‐affected contexts ‐ were technically delivered outside of the criminal justice system but were still delivered in settings where there appeared to be some restriction of movement, deprivation of liberty, and/or some military involvement. We considered these design features to be contra to our definition of a non‐criminal justice intervention and therefore excluded such interventions. For a full list of studies that were excluded using the refined criteria outlined here, see Appendix SV.

Third, the inclusion criteria specifying that at least half of a sample/caseload had to be aged 19 or under in studies of broader interventions (unless data specifically relevant to children and adolescents were presented) were loosened to include two studies in which the proportion of clients aged 19 or under was slightly below 50% (AEF 2018; Cherney 2024). Both studies specifically emphasised the high representation of children and adolescents within the intervention caseload and were therefore seen as relevant.

The practicalities of the screening process diverged from the protocol in two further ways. We did not obtain specific definitions of ‘youth’ from authors who did not specify the age ranges examined in their study. This was because these studies provided broader reflections on working with young people, rather than focusing on a specific age range. Finally, we were unable to share a preliminary list of studies with members of the 5RD CVE network as planned, due to time constraints. However, we included government websites hosting research produced or funded by the constituent governments within the search strategy to ensure that relevant outputs were identified.

## 5 Results

### 5.1 Description of Studies

#### 5.1.1 Results of the Search

The initial corpus of evidence consisted of 20,887 records: 19,477 records identified through the electronic searches of academic databases, and 1410 records identified through the hand searches. De‐duplication resulted in the removal of 6801 duplicate records, with the remaining 14,086 records progressing to title and abstract screening.

12,830 records were removed at the title and abstract screening stage, with 1256 progressing to full text screening. The full texts of 66 of these records were not accessible and so are currently categorised as awaiting classification (see Appendix SVI). The full texts of all remaining records were reviewed, resulting in the exclusion of 1163 records. Twenty‐seven records reporting on 27 unique studies were therefore eligible for inclusion.

An additional four eligible records were identified through forward and backward citation searches and by reaching out to expert researchers. This included two dependent records of studies already included, and two records reporting on new studies. In total, 31 records reporting on 29 unique studies were included in the review.

#### 5.1.2 Included Studies

Thirty‐one records reporting on 29 unique studies were included in the review. All of these studies examined implementation factors and/or moderators (Objective III), with seven also examining how programmes were implemented (Objective II). Only one study was eligible for inclusion in the assessment of effectiveness (Objective I) (Table 9).

**Table 9:** Details of included studies.

Study	Document type	Focus	Name of intervention/practice issues discussed	Location	Relevant dates
*Objective I (Effectiveness) and Objective III (Implementation factors and moderators)*					
1. Kolbe (2019)	Published – Journal Article	Intervention(s)	A youth development programme for young people identified as being at risk of joining Al‐Shabaab	Unspecified country in East Africa	Research conducted 2017–2018
*Objectives II and III (Implementation)*					
2. AEF (2018)	Grey – Research Institution Report	Intervention(s)	Forsa Exit Facility Family Support Centre	The Netherlands	Evaluation covers 2015–2018 (first 3 years of operation)
3. Badurdeen and Ndenyele (2024)	Published – Journal article	Practice	Examines implementation practices of various (international) P/CVE initiatives active in Kenya	Kenya	Study conducted August 2019–2020
4. Brett and Kahlmeyer (2017)	Grey – Research Institution Report	Intervention(s)	STRIVE – Mentorship	Kenya Somalia	Project ran from 2014‐2017, and evaluation was conducted in 2016
5. Cherney et al. (2022a) (a) Cherney et al. (2022a) (b) Cherney et al. (2022b)	(a) Published – Journal Article (b) Grey – Academic	Practice	Examines ‘best practices’ for intervening in youth radicalisation	Australia Austria Germany	Not stated
6. Cherney et al. (2018)	Published – Journal Article	Practice	Examines ‘community‐based services for youth at risk of violent extremism’ across three regions	Australia	Research conducted in 2016
7. Cherney (2024)	Published – Journal Article	Intervention(s)	Intervention 1	Australia	Established in 2014, evaluated in 2020
8. Chisholm and Coulter (2017)	Grey – Government	Practice	Examines social work responses to childhood radicalisation	UK (England)	Research conducted in 2016
9. Cifuentes et al. (2013)	Grey – Research Institution Report	Intervention(s)	The Think Project	UK (Wales)	Evaluation of pilot project (2011)
10. Davies (2023)	Grey – Thesis	Intervention(s)	Examines the range of ‘early interventions’ for youth spanning primary and secondary prevention	UK (Wales)	Research conducted 2020–2022
11. Fisher et al. (2020)	Grey – Research Institution Report	Intervention(s)	STRIVE II – Mentoring STRIVE II – Preventive Communication (Mentoring)	Kenya	Initially established to run from 2017 to 2020, with evaluation conducted in 2020
12. Foster (2010)	Grey – Government	Intervention(s)	Evaluation of Greater Manchester YOT/YOS's Preventing Violent Extremism Projects	UK (England)	Project operated 2009–2010 Interviews conducted between July and September 2009
13. Gielen (2020)	Grey – Thesis	Intervention(s)	Support groups for families of foreign fighters	The Netherlands	Most families received support 2014–2016
14. Glaser (2017)	Published – Book Chapter	Practice	Examines work of professionals working in projects for at risk young people or disengagement programmes for right‐wing extremists Particular focus on whether/how they work with female clients	Germany	Not stated
15. Grossman and Barolsky (2019)	Grey – Academic	Practice	Examines the role of community support in the re‐integration of children, women, and families returning from conflict zones	Australia	Research conducted in 2017
16. i‐Works Research Ltd. (2013)	Grey – Research Institution Report	Intervention(s)	The Think Project	UK (Wales)	Research covers period March 2012–September 2013
17. Joyce and Lynch (2017)	Published – Journal Article	Practice	Examines the role ex‐political prisoners play in programmes working with youth at risk of involvement in political violence	UK (Northern Ireland)	Research conducted 2013–2014
18. Langdon‐Shreeve and Nickson (2021)	Grey – Government	Practice	Examines how children's social care sector and partners manage cases relating to radicalisation	UK (England)	Research conducted 2020–2021
19. Oberg et al. (2023) (a) Oberg et al. (2023) (b) Adams et al. (2023)	Published – Journal Article	Practice	Both documents report findings from *Delivering Effective Services for CVE Intervention* project and examine work of CVE practitioners	Australia	Unspecified
20. Osorno Hernandez (2021)	Grey – Thesis	Practice	Explores experiences of grassroots P/CVE actors	UK (England and Wales)	Interviews conducted 2018–2018
21. Ottis (2016)	Grey – Thesis	Intervention(s)	ReDirect	Canada	ReDirect was established in 2015, research in 2016
22. Rousseau et al. (2024)	Published – Journal Article	Intervention(s)	Polarization	Canada	Established in 2016
23. Sahgal and Kimaiyo (2020)	Published – Journal Article	Intervention(s)	STRIVE II (Mentoring Component)	Kenya	Research covers period 2017–2019
24. Skiple (2020)	Grey – Thesis	Intervention(s)	The Tolerance Project	Sweden	Fieldwork conducted 2015–2016
25. Southern Poverty Law Center (SPLC) and Polarization and Extremism Research and Innovation Lab (PERIL) (2021)	Grey – Academic	Practice	Presents practitioner insights and feedback on a resource developed earlier: ‘Building Resilience & Confronting Risk in the COVID‐19 Era: A Parents and Caregivers Guide to Online Radicalization’	USA	Study conducted January–February 2021
26. Thomas et al. (2017)	Grey – Academic	Intervention(s)	Kirklees Prevent Young Peoples' Engagement Team	UK (England)	Research conducted 2017
27. Uhlmann (2017)	Grey – Government	Intervention(s)	Advice Centre on Radicalisation	Germany	Evaluation ran from April 2016 to August 2017
28. UNDP (2022)	Grey – Government	Intervention(s)	Youth for Social Harmony in the Fergana Valley	Uzbekistan	Experiments conducted in 2020–2021
29. Weine et al. (2018)^a^	Grey – Academic	Intervention(s)	Los Angeles County Department of Mental Health's ‘School Threat Assessment and Response Team’ (START)	USA	Formative evaluation conducted from December 2015 to November 2016

^a^ Weine et al. (2018) subsequently conducted a non‐experimental outcome evaluation of this programme that provided illustrative evidence of this programme producing positive outcomes – however, this evaluation did not meet our inclusion criteria, and so it is not discussed in this review. See Weine, S., Eisenman, D., Martinez, M., Boyd, L., and Brown, M. 2021. *Evaluation of a Targeted Violence Prevention Programme in Los Angeles County, California*. University of Illinois at Chicago.

Eighteen of the included studies analysed a specific intervention (or interventions), and 11 examined broader practice based on interviews with frontline practitioners working in fields such as youth work, social work, children's social care, other stakeholders, communities, and/or representatives of community organisations. Six studies discussed activities that did not meet our inclusion criteria because they also discussed primary‐level or broader capacity‐building interventions and/or activities conducted in a criminal justice setting.^8^ Data relating to these activities was not extracted or included in the analysis. However, a brief description of these activities is shown for relevant studies in Table 10.

**Table 10:** Methodological details of included studies.

Study	Research design	Research question eligibility	Overview of intervention(s)/practice issue(s) examined	Treatment of youth	Data
*Objective I (Effectiveness) and Objective III (Implementation factors and moderators)*					
1. Kolbe (2019)	Unmatched quasi‐experimental design with treatment‐as‐usual comparator: *Treatment as Usual:* At‐risk young people participating in a group‐based intervention. *Treatment:* At‐risk young people participating in the same group‐based intervention receiving supplementary home‐based support from social worker.	Effectiveness	The programme has two components, the second of which was added midway through implementation: (1) Group‐based activities (2) Home‐based social work	Intervention works with youth (aged 13–18 at intake) in a ‘large East African City’ identified by community leaders and CVE staff as at risk of joining Al‐Shabaab.	*Primary data:* Data at intake, programme completion (3 months), and 9‐month post‐completion Total *n* = 113 TAU (*n* = 53) Treatment (*n* = 60) Primary Outcome: Membership of a violent extremist group 9‐month post‐completion. Other Outcome: Graduation from the group intervention.
*Objectives II and III (Implementation)*					
2. AEF (2018)	Mixed methods evaluation of the **Forsa Exit Facility** and the **Family Support Centre**.	Implementation	**Forsa** Forsa provides case management support to adults and young people ‘who harbour extremist convictions’ or ‘who are/have been involved in extremist networks’. **Family Support Centre** Provides case management support to ‘family members of radicalised individuals’ to ‘combat radicalisation of other family members’ and ‘help prevent the perpetration of criminal acts’.	**Forsa** Evaluation reports that approximately 42% of Forsa clients between 2015 and 2018 (*n* = 26) were minors (not defined), and that ‘a large proportion’ were adolescents. **Family Support Centre** Case profile not stated, but insights relevant to working with families of radicalised youth.	*Primary data:* Interviews (*n* = unstated) with programme staff; national and local government stakeholders; programme managers (e.g., board members of foundation that runs programmes; programme coordinators). Interviews with clients (*n* = 6) of both programmes (no further details provided). *Secondary data:* Analysis of programme documentation.
3. Badurdeen and Ndenyele (2024)	Qualitative study: **ethnographic** study of youth who had histories of being involved with the Al‐Shabaab extremist movement and associated P/CVE interventions.	Implementation	Examines implementation of, and reception, to P/CVE interventions in two urban areas in Kenya described as ‘hotspots’ for radicalisation: Majengo (Mombasa) and Majengo (Nairobi).	Study focuses specifically on youth radicalisation and efforts to counter it. Although the majority of participants associated with Al‐Shabaab were adults at time of interview, almost half were aged 20 or under at the point of their radicalisation.	*Primary data:* Qualitative interviews (*n* = 67): individuals currently involved with P/CVE intervention (*n* = 27); family members (*n* = 23); civil society actors and community leaders (*n* = 17) 4 × focus group and 6 × observations of P/CVE workshops and training events.
4. Brett and Kahlmeyer (2017)	Mixed methods evaluation of various pilot programmes delivered through the EU‐funded **STRIVE** project including a mentoring programme for at‐risk youth.	Implementation	**STRIVE (Mentoring)** Mentoring strand of STRIVE is one of several pilot programmes for ‘at risk groups amongst youth’ developed and delivered through this project. Focuses on Majengo region of Nairobi that the authors describe as a ‘deprived area with a significant degree of urban youth marginalisation’ (p. 4). *Other pilot programmes were assessed as being out of scope due to their focus on building the capacity of, and cooperation between, security sector and law enforcement and civil society; strengthening the capacity of women's organisations; and understanding and addressing integration challenges faced by EU‐born youth in Somalia*.	Evaluation does not define ‘at‐risk’ or youth but reports that programme worked with 200+ mentees, ‘especially the 18–24 age group’, and that 20 were identified as ‘being on the path to radicalisation’ and five as ‘presenting serious risks’.	*Primary data:* Interviews and focus groups (*n*=unstated) with stakeholders, beneficiaries, and experts (no further details provided). *Secondary data:* Desk and document review.
5. Cherney et al. (2022a)	Small‐scale qualitative study interviewing subject matter experts about best practices for working with youth in the CVE context.	Implementation	Best practices for working with youth in the CVE context. Discussion spans all levels of prevention and both criminal and non‐criminal justice contexts.	Specifically examines best practices for working with youth – with specific interest in children and adolescents (aged 18 or under).	*Primary data:* Interviews with subject matter experts (*n* = 6): youth worker who managed youth centre programme that included work with radicalised youth; researcher and CVE practitioner; local CVE practitioners involved in implementing interventions.
6. Cherney et al. (2018)	Qualitative study examining community‐based services for youth at risk of radicalisation in specific parts of Melbourne, Brisbane, and Sydney selected by research teams.	Implementation	Study examines the ‘capabilities and needs’ of community‐based organisations in the context of countering youth radicalisation. Research in Sydney focused on area with diverse range of low‐ and middle‐income groups which was described as the ‘residential focus of Sydney Muslims’. Research in Melbourne focused on areas selected based on ‘socio‐economic disadvantage data’. Research in Brisbane focused on area ‘comprising a low socio‐economic demographic and home to a diverse range of cultures, more than 100 different service providers, and approximately 60% of Queensland Muslims’.	Youth‐specific focus (not defined).	*Primary data:* Interviews and focus groups in three regions: Sydney: 15 interviews and 3 focus groups with staff from 18 organisations Melbourne: 18 interviews with service providers Brisbane: 16 interviews with service providers (*n* = 13) and Muslim community leaders (*n* = 3).
7. Cherney (2024)	Mixed methods evaluation of Intervention 1, a case management programme operating in one state in Australia.	Implementation	**Intervention 1** Case management programme working with at risk individuals in community contexts as well as ‘convicted terrorists and radicalised offenders released from prison’.	Programme is not specifically targeted at youth, but 7 of the 15 cases examined in this study were aged 12–17.	*Primary data:* Interviews with programme staff (*n* = 3) and clients (*n* = 2). No further sample details provided. *Secondary data:* Quantitative analysis of case information/data held in case management system (*n* = 15). Clients vary in regard to life history (e.g., criminal history; history of violence; mental illness); familial context (i.e., whether parents are single/married/divorced; involved in extremism/radicalisation).
8. Chisholm and Coulter (2017)	Qualitative study drawing on case studies with 10 local authorities to examine responses to radicalisation and emerging practice.	Implementation	Study examines the role and use of social care interventions in countering radicalisation across 10 local authorities in England (mix of priority areas assessed as having higher level of local radicalisation risk and non‐priority areas).	Discussion is primarily focused on work with children, young people, and their families.	*Primary data:* Interviews/group discussion with strategic staff, frontline social care staff, and partners (e.g., schools, police, health services) across 10 local authorities (*n* = 94) Stakeholder workshop with 19 representatives of relevant national and regional bodies.
9. Cifuentes et al. (2013)	Mixed methods evaluation of a pilot of the Think Project delivered in Swansea, South Wales using pre/post‐test design.	Implementation	**The Think Project** Eight‐week educational intervention aiming to ‘address racism and far‐right extremism in young people’. All participants had been excluded from mainstream education and enroled in alternative provision.	Programme for young people aged 14–16.	*Primary data:* Interviews and survey of young people who attended the final session (*n* = 7).
10. Davies (2023)	Qualitative study examining work of early intervention providers in Wales.	Implementation	Study examines a range of ‘universal’ (i.e., primary) and ‘targeted’ (i.e., secondary) interventions working with youth in Wales. Only data related to interventions offering targeted support (i.e., to individuals referred into them by different actors) is examined in this review. *Universal interventions not examined in the review included lessons, workshops, and training sessions for young people about relevant topics and activities focused on building community cohesion. Projects delivering mix of targeted and universal prevention were included in the analysis*.	Focus is on interventions working with young people aged 11–18.	*Primary data:* Qualitative interviews (*n* = 28) with stakeholders and practitioners. Sample includes youth workers; education workers; local authority staff; teaching staff; police; support workers; and campaigners. Observations of five projects.
11. Fisher et al. (2020)	Mixed methods evaluation of programmes funded through STRIVE II, drawing on primary and secondary qualitative and quantitative data.	Implementation	**STRIVE II** Two of the four strands of STRIVE (Strengthening Resilience to Violent Extremism) II are relevant: 1. Mentorship: Mentoring/counselling for young people assessed as being at risk of radicalisation using specific criteria. Mentees drawn from ‘particularly disadvantaged neighbourhoods’ in Nairobi, Mombasa and Kwale which are considered to be radicalisation ‘hotspots’. 2. Preventive Communication: Included a ‘capacity‐building programme’ for mentors and mentees aiming to develop ‘their skills as communicators, spokespeople and storytellers’. *Strands focused on research and on training for law enforcement agencies not examined in review*.	Mentoring programme worked with youth aged 18–24 identified by mentors and community stakeholders (e.g., parents, teachers, community leaders, social workers) as at risk and meeting specific eligibility criteria set by programme.	*Primary Data:* Interviews with mentors (*n* = 26); observation of mentor training sessions (*n* = 2); two focus groups with beneficiaries (*n* = 18); telephone interviews with programme stakeholders (*n* = unstated). *Secondary data:* Desk review of project‐level documentation (e.g., management reports; policy documents; guideline documents; raw and analysed data; theory of change; and logical framework), including secondary analysis of baseline, midline, end‐line survey data mentors, mentees, stakeholders.
12. Foster (2010)	Qualitative descriptive evaluation of activities delivered across different regions of Greater Manchester.	Implementation	Process evaluation of programming delivered through the Greater Manchester YOT/YOS's Preventing Violent Extremism Projects. Projects commonly involve activities working with individuals inside (e.g., those managed by youth offending teams) and outside of the criminal justice system. *Some regions also deliver primary interventions such as community cohesion and engagement work and (non‐targeted) educational work in schools. Data relating to these activities not examined here*.	Core objective was engaging young people at risk of radicalisation. No specific definition used, but age of interviewees ranged from 12 to 20 years. All identified as Muslim, six were male and five were female.	*Primary data:* Interviews with young people involved in projects (*n* = 11); Observation of groupwork; Interviews with internal staff and with external partners (*n* = not stated). *Secondary data:* Analysis of project activities and materials.
13. Gielen (2020)	Realist evaluation of support groups for families of foreign fighters, drawing on qualitative data.	Implementation	Support groups work with families of those who travelled or who had been stopped from travelling with a view to supporting the families and preventing any potential risk of familial radicalisation.	Work is not restricted only to families of children and adolescents, but sample of interviews included the families of a minor female foreign fighter who had been stopped (*n* = 3); the family of a deceased minor foreign fighter (*n* = 1); and the family of a deceased foreign fighter who had travelled with his wife and children (*n* = 1).	*Primary data:* Interviews (*n* = 9) and focus groups (*n* = 4) with families; interviews (*n* = 5) with five different organisations delivering relevant support; focus group with local municipality representatives (*n* = 6).
14. Glaser (2017)	Qualitative study examining experiences of individuals involved in projects working with ‘right‐wing extremist oriented young people or with young people at risk of becoming right‐wing extremist’ or ‘aimed at initiating and supporting disengagement and deradicalization processes among this target group’.	Implementation	Sample is drawn from practitioners working from programmes working with at‐risk young people and from deradicalisation programmes for right wing extremists in Germany.	Interventions working with young people (not defined) specific focus of this study project.	*Primary data:* Interviews with representatives of 22 projects involved in outreach, street work, or deradicalisation/disengagement.
15. Grossman and Barolsky (2019)	Qualitative examination of how community organisations and practitioners in Victoria can support the reintegration of women and children from conflict zones.	Implementation	Examines potential approaches for supporting the reintegration of women and children and develops a proposed model of case management for supporting them.	Focus is on designing holistic community support model of reintegration for both women and children.	*Primary data:* Interviews with community participants (*n* = 16); government officials (*n* = 5); practitioners (*n* = 2) based in Victoria.
16. i‐Works Research Ltd. (2013)	Mixed methods interim evaluation of the Think Project.	Implementation	**The Think Project** Separate evaluation of same programme examined in Cifuentes et al. (2013) delivered to young people in Wales.	Age range of clients not defined, but specific focus is on ‘young people who are vulnerable and at higher risk’ Participants recruited from educational institutions.	*Primary data:* Pre‐post survey and interviews with young people; interviews with partners (*n*=unstated). Supplemented by data from young people, tutors, and education providers (e.g., records and evaluations completed by tutors at the end of each session). *Secondary data:* Desk review of programme documents (e.g., management information; monitoring data), supplemented by review of relevant policy and literature and population‐level data.
17. Joyce and Lynch (2017)	Qualitative study examining experiences of ex‐political prisoners in working with youth at risk of involvement in political violence in Northern Ireland.	Implementation	Examined interventions include restorative justice programmes, peace‐building initiatives and ex‐prisoner support services.	Work specifically focuses on youth at risk of becoming involved in political violence.	*Primary data:* Interviews with self‐identified Republican (*n* = 25) and Loyalist (*n* = 27) ex‐prisoners involved in restorative justice and peace‐building work, as well as ex‐prisoner support services.
18. Langdon‐Shreeve and Nickson (2021)	Qualitative study drawing on case studies in 11 local authorities to examine the delivery of Prevent work in the children's social services sector.	Implementation	Study examines how the children's social care sector engages in work to prevent youth radicalisation. Follow‐up study to Chisholm and Coulter (2017) that examines how practice and cases related to youth radicalisation have changed.	Focus is on work with youth aged 18 years and under.	*Primary data:* Interviews (*n* = 68) with children's social care staff (*n* = 42); Prevent and other local authority staff (*n* = 13) across 11 local authorities (six Prevent Priority areas); and national and regional stakeholders (*n* = 13). including Counter Terrorism Policing (*n* = 5); Director of Children's Services (*n* = 2); Social workers/principal social workers (*n* = 3); voluntary/community organisations (*n* = 2); Social Work England (*n* = 1).
19. Oberg et al. (2023) (a) Oberg et al. (2023) (b) Adams et al. (2023)	Dependent records of study examining the role of (a) trauma; and (b) gender in work of CVE practitioners in Australia.	Implementation	The *Delivering Effective Services for CVE Intervention* project explores the experience of CVE practitioners in Australia. Documents examine the relevance of (a) trauma and (b) ideology.	Report on findings specific to working with ‘adolescents’ (a); or ‘young men’ (b).	*Primary data:* Interviews with CVE practitioners (*n* = 12) from varying fields, including police, custodial settings, counsellors, teachers, occupational therapists, psychologists, case workers.
20. Osorno Hernandez (2021)	Qualitative study focusing on role of ‘grassroots’ youth workers in P/CVE.	Implementation	Examines ‘grassroots’ P/CVE youth work spanning primary and secondary prevention. Relevant secondary interventions include targeted educational interventions and youth work and safeguarding interventions. *Primary interventions included theatre projects; projects focused on integration and community cohesion; and education work*.	The focus is on youth workers and therefore relates to the people that come into contact with or receive support from youth workers.	*Primary data:* Interviews (*n* = 26) with youth workers, government practitioners, project managers and directors of grassroots organisations working across different parts of the United Kingdom.
21. Ottis (2016)	Qualitative study of Calgary Police Service's ReDirect programme.	Implementation	**ReDirect**, run by Calgary Police Service, is a municipal‐level, community policing, radicalization prevention programme in Canada.	‘Youth’ is not defined, but the thesis comments that ReDirect focuses on youth (p. 12) and that many of the clients are underage (p. 8).	*Primary data:* Interviews (*n* = 14) of people with in‐depth knowledge of ReDirect and/or knowledge of the Canadian CVE space more broadly.
22. Rousseau et al. (2024)	A mixed‐methods study of the Polarization team in Montreal, Canada, focusing on the children of extremist parents.	Implementation	**The Polarization team** is a clinical team providing case management and other services to extremists and their significant others.	This study focuses on minors (aged up to 17 years).	*Primary data:* Qualitative data: interviews with clinical service providers and supervisors (*n* = 5). Focus group discussion with team clinicians (*n* = 7). *Secondary data:* Retrospective review of intervention case file information (total *n* = 160).
23. Sahgal and Kimaiyo (2020)	Mixed methods evaluation of the effect of the STRIVE II mentoring programme focusing on youth resilience.	Implementation	Evaluation of the **STRIVE II** mentoring pilot for at‐risk youth examined in Fisher et al. (2020).	Programme is designed for at‐risk youth aged between 14 and 35 years. The majority of clients are aged 14–24 years. Mentees drawn from parts of Nairobi and Mombasa. Mentees varied in terms of age, employment status, and educational level.	*Primary data:* Focus groups with mentees (*n* = 72); Longitudinal survey examining differences from baseline at midline (*n* = 254) and endline (*n* = 93).
24. Skiple (2020)	An ethnographic (qualitative) study of the implementation of the Tolerance Project in a municipality in Sweden.	Implementation	**The Tolerance Project** is an educational project delivered to 14‐ and 15‐year‐olds in Swedish schools. The project brings intolerant youths identified by schools together with participants with more pro‐social attitudes.	The project is advertised within participating schools to those aged 14 and 15 years.	*Primary data:* Interviews with delivery team (*n* = 5), regional crime prevention office employees (*n* = 4), municipality employees (*n* = 2), security coordinator (*n* = 1) and a police officer (*n* = 1). Participatory observations (*n* = 7 meetings).
25. Southern Poverty Law Center (SPLC) and Polarization and Extremism Research and Innovation Lab (PERIL) (2021)	Qualitative study capturing insights from professionals working with youths in the United States.	Implementation	The study captures practitioner feedback on a resource titled ‘Building Resilience & Confronting Risk in the COVID‐19 Era: A Parents and Caregivers Guide to Online Radicalization’.	Captures insights from professionals who work directly with youth, such as teachers and social workers.	*Primary data:* Focus groups conducted with youth‐focused professionals divided into three clusters: teachers and educators; school counsellors and social workers; and coaches, mentors, and youth group leaders (total *n* = 43).
26. Thomas et al. (2017)	A qualitative study into the creation and operationalisation of Kirklees Prevent Young People's Engagement Team.	Implementation	The **Kirklees Prevent Young Peoples' Engagement Team** delivers various activities, including direct prevention work with youths. *PYPET also engages in broader community engagement and capacity‐building/training activities that are not examined here*.	The Prevent Young Peoples' Engagement Team focuses on youth interventions and youth work, although ‘youth’ is not defined in the study.	*Primary data:* Interviews (total *n* = 11): Council officers with Prevent‐related responsibilities (*n* = 4), members of the PYPET (*n* = 4), Police Prevent staff that work with the PYPET (*n* = 3).
27. Uhlmann (2017)	A mixed methods study, primarily based on interviews and focus groups, evaluating the Advice Centre on Radicalisation (BAMF Advice Centre) in Germany.	Implementation	The **Advice Centre on Radicalisation** (BAMF Advice Centre) provides counselling services to people at risk of radicalisation and those in the social environment of individuals who have been radicalised.	The Advice Centre is not youth‐specific, but the descriptions of local partners suggest that a significant proportion of their work involves youth. Additionally, the report implies that young people are the subject of a lot of the referrals.	*Primary data:* Interviews and focus groups with advisors from BAMF Advice Centre (*n* = unstated). Plus, interviews with government security agencies and state coordinating agencies, interviews with local partners, and interviews with experts (*n* = unstated). Focus groups with counsellors of local partners and evaluation workshops with local partners (*n* = unstated). *Secondary data:* Analysis of counselling logs.
28. UNDP (2022)	Quasi‐experimental evaluation of behavioural intervention aiming to improve levels of (sustained) engagement in a youth‐focused programme.	Implementation	**‘Youth for Social Harmony in the Fergana Valley’** aims to enhance community resilience, particularly focusing on youth empowerment, including equipping youth – and in particular young women – with opportunities and skills that will aid them in the job market. Fergana Valley has low average income compared to national average, and lowest growth in nominal wages; high rates of juvenile criminality, and specific challenges with violent extremism.	Experiment examined whether youth ambassadors were able to increase levels of participation amongst ‘marginalised youth aged 15+’ who were identified as being at an elevated risk of radicalisation.	*Secondary data:* Quasi‐experimental analysis of intervention attendance records for two cohorts of young people: control *n* = 67; treatment *n* = 75.
29. Weine et al. (2018)	Formative evaluation of a CVE framework in Los Angeles.	Implementation	The formative evaluation reports on the formation of the Los Angeles Framework for Countering Violent Extremism. It sets out how the ‘School Threat Assessment Response Team’ (START) will be built upon for secondary CVE interventions.	‘Youth’ is not defined, and the CVE framework is not limited to a youth focus, but the START programme is focused on school threats and receives referrals from educational institutions and parents (among other sources) about individuals of concern.	*Primary data:* Interviews, consultation and tabletop exercises (*n* = unstated) with local stakeholders (e.g., law enforcement; local government agencies; faith‐based organisations; service organisations). *Secondary data:* Selective analysis of qualitative data from a separate study of the Los Angeles Police Department and the county's Muslim‐American community (interviews *n* = 100).

Twenty‐eight studies reported on primary data. These included 1 quasi‐experimental quantitative study, 18 qualitative studies, and 9 mixed methods studies that complemented qualitative data collection with a survey (*n* = 3) or secondary analysis of programme data (*n* = 6). The most common method of primary data collection was interviews (including focus groups; *n* = 26). Ten studies interviewed intervention beneficiaries/participants who had been identified and/or referred into programmes by different actors/institutions, most commonly by community stakeholders, educators (and educational institutions), social workers, youth workers, and police. Twenty‐three studies interviewed practitioners, staff or professional stakeholders from a range of different sectors and institutions – including civil society and community organisations; the police; local government practitioners; educators; social workers; youth workers; psychologists and other mental health professionals – either directly or indirectly involved in intervention delivery. Four studies interviewed families, and 14 interviewed other stakeholders (*n* = 14), such as local or national government staff and programme funders.

Five studies collected data through observation of working practices, workshops or training events. Four studies conducted surveys or other quantitative interviews with young people (*n* = 4), practitioners (*n* = 1) or families (*n* = 1). When stated, interview sample sizes ranged from 7 to 72, and survey sample sizes ranged from 7 to 347. The remaining study used a quasi‐experimental design to examine attendance data amongst two different cohorts of intervention beneficiaries (*n* = 142).

Eleven studies provided insights relevant to working with children and adolescents specifically (i.e., those aged 19 or under). This included nine studies reporting on interventions or practices that were specific to this age group; one study that examined issues relating to children in the context of a broader intervention (Rousseau et al. 2024); and one study interviewing practitioners who worked in the ‘adolescent CVE space’ (Oberg et al. 2023). Twelve other studies focused on broader youth cohorts. Eight of these did not define youth. The remaining four used varying definitions: aged 18–24 (*n* = 2); 14–24 (*n* = 1); and 15+ (*n* = 1). Six studies did not report on youth‐specific interventions but were included because children and adolescents (*n* = 2) or youth more broadly (*n* = 4) were identified as a significant area of focus. These assessments were based on either their caseload data or the intervention description provided by the author. Socio‐demographic information was rarely reported. However, the few studies that provided this information highlighted how caseloads varied in regard to age and level of education. Studies in Australia and Canada also pointed to variation in life and family history and mental health.

Following the protocol, only studies published in English were included. However, studies reported on data collected from a total of 11 countries, plus 1 unspecified country in East Africa. This total includes nine studies that collected data from samples in the United Kingdom, disaggregated to four in England, three in Wales, one covering England and Wales, and one referring to CVE work in Northern Ireland. Data from Australia featured five times. Four studies focused on interventions or practices in Kenya. Other countries examined include Germany (*n* = 3), the Netherlands (*n* = 2), Canada (*n* = 2), the United States (*n* = 2), Somalia (*n* = 1), Austria (*n* = 1), Uzbekistan (*n* = 1), and Sweden (*n* = 1). The majority of studies, therefore, examined interventions or practices in the Global North.

Twenty‐eight studies focused on a single country (*n* = 27) or examined an eligible intervention that was delivered in a single country as part of a broader evaluation (*n* = 1). Most (*n* = 18) of these studies focused either on a single area/region within a country (*n* = 13), or on interventions targeted at a small number of areas (*n* = 5). This included studies reporting on interventions in Africa (*n* = 5) that were specifically targeted at cities (or specific parts of cities) that had experienced high rates of youth radicalisation and deprivation. Four of these studies focused on disadvantaged areas in Nairobi and/or Mombasa, including two reporting on the STRIVE II mentoring programme in both cities (Fisher et al. 2020; Sahgal and Kimaiyo 2020). Similarly, the single study examining an intervention in Uzbekistan was implemented in a disadvantaged region of the country with a specific history of youth radicalisation (UNDP 2022). The remaining studies examined interventions that operated nationally (*n* = 2); looked across a diverse range of regions within a country (*n* = 2); or examined practices within a country more broadly (*n* = 7).

The evidence in this review is drawn from 11 published documents and 20 unpublished documents. All but one of the published documents were journal articles (*n* = 10), the other was a chapter in an edited volume (*n* = 1). Grey literature sources were evenly split between government reports (including those published by independent researchers for government) (*n* = 5); unpublished academic outputs (*n* = 5); academic theses (*n* = 5); and research institution reports (*n* = 5). All documents were published between 2010 and 2024, with most published from 2020 onwards (*n* = 14). Although inclusion criteria permitted publications from 2000 onwards, we found no studies that met the full inclusion criteria published between 2000 and 2009.

#### 5.1.3 Summary of Interventions Examined in Included Studies

A range of different interventions working with children and adolescents (and youth more broadly) was identified in the literature. Most studies examined more direct forms of prevention in which practitioners worked directly with at‐risk or radicalised young people. Different models of direct prevention were used, including more tailored *case management* and *mentoring* approaches, and less individualised *educational* approaches. Indirect models of prevention were less widely discussed, but included *case management* for families and *family support groups*. These different categories are examined below, alongside other interventions that could not easily be categorised – such as those captured in studies examining broader prevention work, or multiple programmes.

##### 5.1.3.1 Case Management Programmes

Case management interventions are the most widely examined form of intervention in this review. The searches identified a significant number of studies that examined the implementation of a case management intervention or presented evidence on the implementation of case management in this context (AEF 2018; Cherney et al. 2018; Cherney et al. 2022; Cherney 2024; Chisholm and Coulter 2017; Langdon‐Shreeve and Nickson 2021; Osorno Hernandez 2021; Ottis 2016; Rousseau et al. 2024; Thomas et al. 2017; Uhlmann 2017; Weine et al. 2018).^9^ Two further studies also explored the potential for developing new case‐managed CVE structures (Grossman and Barolsky 2019; Weine et al. 2018).

This literature includes studies reporting on specific case‐managed interventions, such as Forsa and the Family Support Centre in the Netherlands (AEF 2018) and Intervention 1 in Australia (Cherney 2024); studies examining how specific agencies performed a counter‐radicalisation function in the context of wider, multi‐agency case management processes (e.g., Chisholm and Coulter 2017; Langdon‐Shreeve and Nickson 2021); and studies reporting on CVE work with youth more broadly that provided insights on the utility and implementation of case management in this context (e.g., Cherney et al. 2018; Cherney et al. 2022a; Grossman and Barolsky 2019; Osorno Hernandez 2021). The use of case management spanned secondary and tertiary prevention, and interventions operated across contexts, including clinical, community, educational, and home settings.

Three types of case management programmes are examined in this review:

1. *Case management programme for at‐risk or radicalised clients:* In most programmes, the at‐risk or radicalised (young) person is the primary client who is provided with a tailored package of multidisciplinary support, often including some work with families, such as family therapy (e.g., AEF 2018; Cherney 2024).
2. *Case management programmes for family members and social contacts of at‐risk or radicalised individuals*: Other programmes seek to prevent or counter the radicalisation of at‐risk or radicalised youth indirectly by working with family members and other social contacts (e.g., AEF 2018; Uhlmann 2017). The family member or other contact is therefore the primary client, but the support provided to them is focused on countering the radicalisation of an ‘index client’ and/or other family members (AEF 2018; Uhlmann 2017). A young person may therefore be considered at risk in part due to the confirmed or suspected radicalisation of a family member, and/or may be deemed to require some form of clinical intervention for this reason (Rousseau et al. 2024). This form of case management is discussed in more detail in the section on indirect prevention.
3. *Case management programmes for young people impacted by parental radicalisation*. Grossman and Barolsky (2019) explore the utility of using case management to deliver whole‐of‐family support to female and child returnees from conflict zones to Australia, and Rousseau et al. (2024) interviewed clinicians about case management work with children of extremist parents. Although these approaches were primarily oriented towards supporting reintegration (Grossman and Barolsky 2019) and supporting children overcome issues such as stigma and distress linked to parental extremism (Rousseau et al. 2024), both studies provide relevant insights as they examine work with young people exposed to extremism.

Case management interventions are commonly multi‐agency in nature and rely on partnership working between actors working in different sectors. In most cases, the police and other criminal justice agencies are important partners within these structures, although their role varies. For example, whilst programmes like ReDirect in Canada are police‐led (Ottis 2016), the police play a more supportive or restricted role within other local case management structures (e.g., Chisholm and Coulter 2017; Thomas et al. 2017).^10^

Most programmes examined in the review are government‐funded. However, several studies examine ‘hybrid’ models: programmes delivered by civil society organisations that receive some level of government funding, coordination, or oversight (Grossman and Barolsky 2019; Osorno Hernandez 2021; Uhlmann 2017). Programmes also vary as to whether they have a local or national focus. Some operate in specific regions or cities (e.g., Ottis 2016; Cherney 2024), whereas others operate across regions or nationwide, although local delivery was often found to vary across geographies.

Although there is no single model of case management, the interventions included in this review commonly use some variation of the staged framework outlined in a previous Campbell Collaboration review by Lewis, Marsden, Cherney, et al. (2024). Potential clients are first identified and referred into programmes, at which point their eligibility is assessed before a more detailed assessment is undertaken – often supported by specific risk and needs (RNA) assessment tools – to identify risk and protective factors and needs that are specific to the individual case. This assessment informs the design and development of what is often a multidisciplinary intervention plan that outlines a tailored and bespoke package of support targeted at the factors and needs relevant in an individual case and delivered by different intervention providers. These might include, for example, social workers, mental health practitioners, and ideological experts. The implementation and impact of this plan are monitored, and the plan is revised if needed to respond to emerging and changing needs, with the client exiting the programme when sufficient progress has been made.^11^

None of the studies included in this review report on established case management programmes that were specifically designed for younger cohorts – although the proposed case management approaches discussed by Weine et al. (2018) and Grossman and Barolsky (2019) had a more specific youth focus. The included interventions, therefore, worked with children and adolescents as part of broader caseloads.^12^ Whilst most of these studies did not provide a demographic breakdown of intervention caseloads, those that did highlighted that young people often make up a high proportion of intervention clients. For example, AEF (2018) reported that 42% of cases supported by Forsa from 2015 to 2018 were ‘minors’, and that ‘a large proportion’ were adolescents.

##### 5.1.3.2 Mentoring Interventions

Mentoring is another form of tailored approach that is commonly used in this context. These programmes work by pairing one or more mentors with a client and are tasked with exploring and addressing that person's needs – predominantly through one‐to‐one engagement, but at times also involving group work. Three studies evaluated secondary mentoring interventions delivered through the STRIVE Horn of Africa and STRIVE II projects that, whilst not solely focused on children and adolescents, had a particular focus on youth aged up to age 24 (Brett and Kahlmeyer 2017; Fisher et al. 2020; Sahgal and Kimaiyo 2020). Other studies also discussed how programmes might offer mentorship to young people as part of broader packages of support consisting of different, multidisciplinary, components (Chisholm and Coulter 2017; Rousseau et al. 2024).^13^

There is no single model of mentoring. However, several programmes reflect elements of a case management approach – even when not formally defined as case management programmes – in that they follow a staged process similar to that outlined in the previous discussion. For example, the STRIVE II mentoring programme only works with individuals who meet certain eligibility criteria that are used to identify those who are at risk of becoming violent extremists (Fisher et al. 2020; Sahgal and Kimaiyo 2020).^14^ Much of the support provided through mentoring programmes is provided by the mentor assigned to a case, and the match between the mentor and the mentee is fundamental to the success of these interventions (Fisher et al. 2020). However, programmes may also connect clients to other forms of support when required, whether delivered through in‐house intervention providers or by external partners (Fisher et al. 2020; Rousseau et al. 2024).

Although not strictly a mentoring intervention, the study by Kolbe (2019) discusses an intervention that worked in a similar way to those mentoring interventions described above. The intervention worked with youth aged 13–18 identified as being at risk of joining violent extremist groups and consisted of two components: a group‐based intervention delivered through a local Mosque, and a home‐based social work component that was only offered to a subset of clients. This social work component worked in a similar way to mentoring approaches, as it involved a dedicated social worker engaging with young people regularly so as to address any issues and facilitate their ongoing participation.

##### 5.1.3.3 Educational Interventions

Although most educational work in the CVE context is oriented towards primary prevention, several studies examined the implementation of targeted educational interventions working with young people identified as being at risk of radicalisation (Cifuentes et al. 2013; i‐Works Research Ltd. 2013; Davies 2023; Osorno Hernandez 2021; Skiple 2020).^15^ These interventions commonly deliver group sessions to participants selected on the basis of cognitions and behaviours indicating heightened risk of extremist violence.

Examples of relevant projects included the Tolerance Project in Sweden (Skiple 2020), and the Think Project in Wales (Cifuentes et al. 2013; i‐Works Research Ltd. 2013), which has since been renamed (Davies 2023).^16^ Both projects conduct group‐based work with youth aged under 16 who might be considered as being at heightened risk of right‐wing radicalisation (broadly speaking), but they work in different ways. The Think Project worked exclusively with young people aged 14–16 identified as being ‘higher risk’ or ‘vulnerable’ – based on exhibiting racist views and behaviours – through a series of workshops over a 6‐ to 8‐week period. Each workshop explores a different theme, such as ‘identity and culture, diversity, migration and asylum, and understanding extremism’ (i‐Works Research Ltd. 2013, 7). The Tolerance Project is similarly organised around a course made up of approximately 10 sessions delivered over the course of the school year and was again ‘developed to target young people at risk of being drawn to racist or extreme organizations’ (Skiple 2020, 424). These sessions bring ‘intolerant’ youth together with more tolerant peers with the view to encouraging dialogue and debate, and a constructive challenging of views.^17^

These programmes are underpinned by an inclusive logic, as young people are given space to share their views with providers and/or their peers, with a view to ‘promoting dialogue between different perspectives’ (Skiple 2020, 424). The assumption is that ‘safe spaces’ (Osorno Hernandez 2021) that allow young people to share their views are useful settings for promoting constructive dialogue and debate, which is likely to be more effective in challenging prejudicial views than simply excluding these young people. The success of this approach rests on the skills and confidence of the intervention provider and requires a skilled and experienced facilitator, as many educators likely lack the confidence or knowledge to perform this function (Skiple 2020; Cifuentes et al. 2013).

##### 5.1.3.4 Indirect Intervention

Several studies reported on interventions that worked with families or other people known to at‐risk or radicalised young people with the stated aim of preventing the (further) radicalisation of the young person in question, or the potential radicalisation of family members, such as siblings (AEF 2018; Grossman and Barolsky 2019; Gielen 2020; Uhlmann 2017). These interventions fell into two categories: case management programmes that provide tailored support to family members or other individuals within the ‘social environment’ of a young person, as outlined above (AEF 2018; Grossman and Barolsky 2019; Uhlmann 2017); and support groups for family members of radicalised individuals delivered in the Netherlands (Gielen 2020). These programmes were not always specifically designed to prevent youth radicalisation, but concerns related to the radicalisation of children and adolescents made up a large proportion of caseloads.

Case management programmes work through families to counter the (further) radicalisation of at‐risk or radicalised young people and/or prevent further familial radicalisation.^18^ For example, the Family Support Centre in the Netherlands ‘strives to combat radicalisation of other family members, for example, brothers or sisters, and to help prevent the perpetration of criminal acts’ (AEF 2018, 4), whilst Germany's BAMF Advice Centre aims to ‘stabilise the social environment of radicalised individuals and individuals at risk of radicalisation’ and to ‘influence radicalised individuals through their social environment to initiate or further advance a process of deradicalisation’ (Uhlmann 2017, 5). Support is primarily delivered face‐to‐face, although the BAMF Advice Centre also provides telephone counselling before connecting clients to local providers. As discussed above, case management programmes for family members and others affected by radicalisation also often work with the young person as part of a ‘whole‐of‐family’ approach (AEF 2018; Grossman and Barolsky 2019; Uhlmann 2017). This approach is used by the Family Support Centre in the Netherlands (AEF 2018) and the BAMF Advice Centre in Germany (Uhlmann 2017) and was identified as an important element of the case management model discussed by Grossman and Barolsky (2019).

An evaluation of the BAMF Advice Centre encapsulates the logic of this approach: ‘the assumption that the (emotional) proximity of family members and the social environment would make them best able to counteract an existing or worsening radicalisation’ (Uhlmann 2017, 22). These programmes are therefore rooted in a ‘socio‐ecological’ approach whereby effective counter‐radicalisation work is seen as resting on addressing potential issues and leveraging strengths that exist in the young person's environment (Ellis et al. 2022). This type of approach is explored in detail in the main analysis.

The second category of programming is reflected in support groups delivered to family members affected by radicalisation in the Netherlands (Gielen 2020). This is a group‐based form of support that brings family members affected by jihadist radicalisation together with a view to both providing psycho‐social support and, in some cases, countering potential familial radicalisation, including the radicalisation of young people.

The prevention of youth radicalisation was not always the sole or even primary focus of family support programmes, including some case‐managed programmes. Often, these programmes focused on helping families cope with the effects of familial radicalisation, for example, the psychological impact of a family member having travelled to and/or died in a conflict zone might be the primary focus (AEF 2018; Gielen 2020). However, every programme of this kind included in this review identified the prevention of youth radicalisation as an important focus of their work when relevant to a specific case.^19^

##### 5.1.3.5 Other

A number of interventions could not easily be categorised using the typology above. This reflected the diversity of activities discussed in studies that offered broader reflections on working with at‐risk and radicalised young people (Cherney et al. 2018; Cherney et al. 2022; Glaser 2017; Joyce and Lynch 2017; Oberg et al. 2023; Southern Poverty Law Center (SPLC) and Polarization and Extremism Research and Innovation Lab (PERIL) 2021; UNDP 2022); studies examining projects that contained multiple and/or overlapping components that could not be easily categorised (Badurdeen and Ndenyele 2024; Cherney et al. 2018; Foster 2010; and studies examining multiple interventions (Davies 2023; Osorno Hernandez 2021). Examples of each type of study are discussed below.

Several studies examined a wide variety of interventions delivered in a range of different settings. For example, Davies (2023) and Osorno Hernandez (2021) both examined the delivery of various projects funded or associated with the Prevent strategy in England and Wales, in addition to some projects operating independently of Prevent or the government. These studies observed various P/CVE activities, including a 4‐week ‘Project for Boys’ exploring radicalisation, gangs and drugs, delivered by a specialist youth worker, targeting ‘small groups of boys aged 13–16 who had been referred by teachers and/or support workers’ (Davies 2023, 60–61) and multiple initiatives delivered by ‘grassroots’ youth workers (Osorno Hernandez 2021). These studies capture valuable insights from practitioners working across a range of projects, and the lessons that can be taken from their professional experience, including challenges commonly faced when conducting CVE work and contextual issues that can impact their work.

These lessons complement older studies that capture youth‐focused practitioner insights, such as Foster's (2010) evaluation of Greater Manchester's various PVE projects delivered by the region's Youth Offending Team (YOT) and their project partners, including music and film‐focused initiatives. A more recent practice‐focused example is a study conducted by Cherney et al. (2022a) that explores best practices in youth rehabilitation of violent extremists, drawing upon interviews with Subject Matter Experts in Australia, Austria and Germany. Various lessons relating to programme design and how to tailor interventions to youths are among the takeaways from this study.

Together, these studies capture perspectives, concerns and best practices from those tasked with delivering various activities and provide valuable insights that complement the studies included elsewhere in the typology that focus on specific forms of intervention.

#### 5.1.4 Excluded Studies

**Figure 1:**
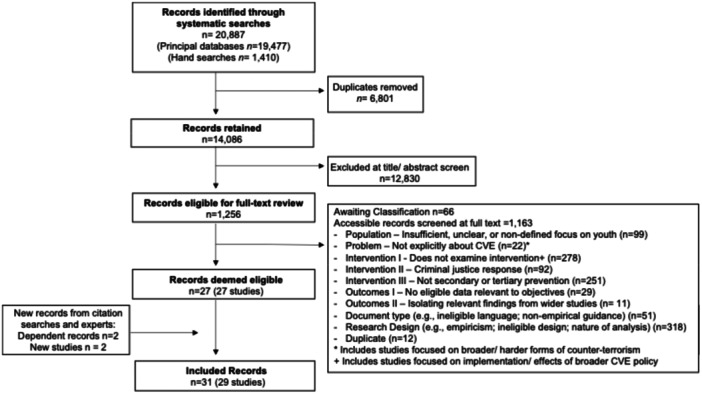
PRISMA diagram.

A total of 1163 records were excluded at the full‐text screening stage. Records were most commonly screened based on their research design (*n* = 318), for example, because they did not present empirical data, used an ineligible research design, or were review articles; because they did not examine an intervention (*n* = 278), for example, studies examining processes of radicalisation, broader perspectives of CVE programmes, or the implementation of CVE policies and strategies more broadly; or because the intervention being examined was not a form of secondary or tertiary prevention (*n* = 251), including studies examining primary interventions and capacity‐building programmes. Other reasons for exclusion are shown in the PRISMA diagram presented in Figure 1 above. For a full list of studies excluded at full‐text screen, see Appendix SVII.

##### 5.1.4.1 Boundaries of Primary and Secondary Prevention

There was not always a clear boundary between primary and secondary prevention. Two related challenges complicated the screening process. First, as already noted, several programmes working with at‐risk populations (including youth) identified risk at the collective or community level (e.g., Swedberg and Smith 2011). Second, programmes often used broad criteria when identifying risk, and we note disagreements between previous reviews when categorising interventions such as the Introducing New Vocational Education and Skills Training (INVEST) programme in Afghanistan as either primary (Hassan et al. 2021a) or secondary (Madriaza et al. 2022). Researchers have also previously noted how programmes identifying risk at the collective level or using broad criteria have struggled to identify at‐risk youth (Ipp et al. 2014; Management Systems International 2017). Similar criticisms have been made of programmes that are explicitly defined as secondary interventions (e.g., Pettinger 2020), and the ability to identify and reach at‐risk clients is an important implementation factor. Therefore, an inability to reach those at risk does not necessarily mean that an intervention is not a form of secondary prevention. However, we concluded that insights drawn from interventions that aim to focus on at‐risk or vulnerable youth were less relevant to this review when:

– Risk was defined at the collective or community level (or specific sub‐groups thereof) rather than at the individual level.
– The criteria used to identify risk at the individual level were so broad that the majority of those meeting them were unlikely to be at specific risk of radicalisation.

Whilst we accept other research teams might make different choices about the eligibility of such studies, we did so to maintain fidelity with the research questions and the concern of this review with individuals already radicalised, or at direct risk of radicalisation.

##### 5.1.4.2 Isolating Relevant Findings From Broader Studies

Many studies examining CVE programming for youth did not solely or explicitly focus on secondary or tertiary prevention, for example, when studies analysed the work of a range of interventions or evaluated programmes that had multiple components. These studies were only included when evidence meeting our inclusion criteria could be isolated and extracted (e.g., Davies 2023; Fisher et al. 2020; Osorno Hernandez 2021). When this was not possible, the study was excluded. Two qualitative records excluded for this reason reported on a study of youth work (Aiello et al. 2018; Puigvert et al. 2020). A sensitivity check was completed, which involved comparing the findings from these two studies against our findings once the analysis had been completed to ensure that including these studies would not have impacted the conclusions drawn in this review.

##### 5.1.4.3 Criminal Justice Context

The boundaries of the criminal justice system were not always clear when studies reported on multiple activities that spanned criminal justice and non‐criminal justice contexts. In such cases, studies were excluded when it was not possible to identify and extract empirical evidence relating to activities that were in scope (e.g., Hirschfield et al. 2012; Meringolo 2020). A sensitivity check was undertaken, and the findings from such studies were compared against the results of our analysis to ensure their inclusion would not have impacted our conclusions. A related question was whether programmes that worked with clients in both criminal justice and non‐criminal justice contexts were in scope. Studies reporting on such interventions were included when they clearly specified that they worked with relevant clients in both contexts (e.g., AEF 2018; Cherney 2024).

##### 5.1.4.4 Relevance to Children and Adolescents

A significant proportion of empirical research examining secondary and tertiary prevention is likely to have some relevance to children and adolescents, given that young people make up a significant and growing proportion of caseloads. However, only studies that explicitly examined work with youth, and/or that presented robust findings relating to youth cohorts, were eligible for inclusion. This meant that studies presenting only anecdotal data relating to youth or that did not clearly define or specify whether and how an intervention worked with youth, or if the findings presented were specific to work with young people, were excluded (e.g., Jackson et al. 2019; Spalek et al. 2010).

#### 5.1.5 Risk of Bias in Included Studies

Table 11 shows the risk of bias assessments for the two quasi‐experimental research designs included in this review, including the single study that was included in the analysis of effectiveness (Kolbe 2019). Both studies were assessed as having a serious risk of bias – based on the risk of bias for the Confounding domain being scored as serious for both studies due to neither study controlling for important confounding areas. Caution is therefore needed when interpreting the results of both studies.^20^

**Table 11:** ROBINS I assessments for quasi‐experimental studies (*n* = 2).

Study	Confounding	Participant selection	Intervention classification	Departure from intended interventions	Missing data	Measurement of outcomes	Reported result	Overall
Objective I (Effectiveness) and Objective III (Implementation factors and moderators)								
Kolbe (2019)	Serious	Low	Serious	Low	Low	Moderate	Moderate	Serious
Objective III (Implementation factors and moderators)								
UNDP (2022)	Serious	Low	Low	Low	Low	Moderate	Moderate	Serious

Assessments of studies using qualitative methods using the CASP tool are shown in Table 12. The primary purpose of this assessment was to identify any studies that should be excluded based on having a critical weakness in regard to research design or sampling. Although the CASP tool is not designed to produce cumulative scores, all included studies scored positively on at least 7 of the 10 domains, which gave us greater confidence in the methodology based on the information available. In the analysis that follows, we distinguish between studies that scored positively on 7 or 8 domains (moderate quality) and those that scored positively on 9 or 10 (high quality) to indicate the strength of the qualitative evidence. Notably, one‐third (*n* = 9) of all qualitative designs evaluated with the CASP tool scored positively on all 10 domains. The overall quality of the evidence was therefore considered high.

**Table 12:** CASP assessment of qualitative and mixed methods studies (*n* = 27) included in analysis of Objectives II and III.

Study	Clear aims	Methods appropriate	Design appropriate	Recruitment appropriate	Data collection appropriate	Adequate consideration researcher–participant relationship	Ethical issues considered	Data analysis rigorous	Clear statement of findings	Research valuable
AEF (2018)	Yes	Yes	Yes	Yes	Yes	Can't tell	Can't tell	Can't tell	Yes	Yes
Badurdeen and Ndenyele (2024)	Yes	Yes	Yes	Yes	Yes	Can't tell	Can't tell	Can't tell	Yes	Yes
Brett and Kahlmeyer (2017)	Yes	Yes	Yes	Yes	Yes	Can't tell	Can't tell	Yes	Yes	Yes
Cherney et al. (2022a)	Yes	Yes	Yes	Yes	Yes	Yes	Yes	Can't tell	Yes	Yes
Cherney et al. (2018)	Yes	Yes	Yes	Yes	Yes	Can't tell	Yes	Yes	Yes	Yes
Cherney (2024)	Yes	Yes	Yes	Yes	Yes	Can't tell	Yes	Yes	Yes	Yes
Chisholm and Coulter (2017)	Yes	Yes	Yes	Yes	Yes	Can't tell	Can't tell	Yes	Yes	Yes
Cifuentes et al. (2013)	Yes	Yes	Yes	Yes	Yes	Can't tell	Can't tell	Can't tell	Yes	Yes
Davies (2023)	Yes	Yes	Yes	Yes	Yes	Yes	Yes	Yes	Yes	Yes
Fisher et al. (2020)	Yes	Yes	Yes	Yes	Yes	Yes	Can't tell	Yes	Yes	Yes
Foster (2010)	Yes	Yes	Yes	Yes	Yes	Can't tell	Can't tell	Yes	Yes	Yes
Gielen (2020)	Yes	Yes	Yes	Yes	Yes	Yes	Can't tell	Yes	Yes	Yes
Glaser (2017)	Yes	Yes	Yes	Yes	Yes	Can't tell	Can't tell	Can't tell	Yes	Yes
Grossman and Barolsky (2019)	Yes	Yes	Yes	Yes	Yes	Yes	Yes	Yes	Yes	Yes
i‐Works Research Ltd. (2013)	Yes	Yes	Yes	Yes	Yes	Can't tell	Can't tell	Yes	Yes	Yes
Joyce and Lynch (2017)	Yes	Yes	Yes	Yes	Yes	Can't tell	Can't tell	Yes	Yes	Yes
Langdon‐Shreeve and Nickson (2021)	Yes	Yes	Yes	Yes	Yes	Can't tell	Can't tell	Yes	Yes	Yes
Oberg et al. (2023)	Yes	Yes	Yes	Yes	Yes	Yes	Yes	Yes	Yes	Yes
Osorno‐Hernandez (2021)	Yes	Yes	Yes	Yes	Yes	Yes	Yes	Yes	Yes	Yes
Ottis (2016)	Yes	Yes	Yes	Yes	Yes	Yes	Yes	Yes	Yes	Yes
Rousseau et al. (2024)	Yes	Yes	Yes	Yes	Yes	Yes	Yes	Yes	Yes	Yes
Sahgal and Kimaiyo (2020)	Yes	Yes	Yes	Yes	Yes	Yes	Yes	Yes	Yes	Yes
Skiple (2020)	Yes	Yes	Yes	Yes	Yes	Yes	Yes	Yes	Yes	Yes
Southern Poverty Law Center (SPLC) and Polarization and Extremism Research and Innovation Lab (PERIL) (2021)	Yes	Yes	Yes	Yes	Yes	Can't tell	Yes	Can't tell	Yes	Yes
Thomas et al. (2017)	Yes	Yes	Yes	Yes	Yes	Yes	Can't tell	Can't tell	Yes	Yes
Uhlmann (2017)	Yes	Yes	Yes	Yes	Yes	Yes	Yes	Yes	Yes	Yes
Weine et al. (2018)	Yes	Yes	Yes	Yes	Yes	Can't tell	Can't tell	Can't tell	Yes	Yes

All 27 studies assessed as having clear aims and findings, and as producing valuable research based on the noted limitations of the evidence base as discussed earlier. The fact that each study examined topics relating to implementation meant that their use of qualitative research and specific research designs was considered appropriate. The recruitment strategies and data collection approaches used were also considered appropriate for eliciting data relevant to the aims of each individual study, with the types of participants selected well‐placed to produce relevant insights (e.g., practitioners or intervention staff were interviewed when examining facilitators and barriers of implementation). The fact that several of these studies did not specify an exact sample size introduces a potential source of bias. However, their recruitment approach was still considered appropriate based on the type and range of participants being sampled.

There were, however, some common limitations in the evidence base. First, few studies specifically discussed the relationship between researcher(s) and participants or ethical considerations. Although studies would have been excluded had they clearly failed to recognise a specific relationship that might have biased the results (e.g., a clear power differential) or had been assessed as operating in an unethical way, the reviewers did not consider such issues to be present. Similarly, studies did not always outline how data were analysed (i.e., by describing a specific analytical approach), which is not uncommon for qualitative research. Whilst studies that did not present evidence in support of their conclusions were excluded, the failure to outline a specific analytical framework was not, in isolation, used as a basis for excluding studies.

The methodologies used to collect and analyse primary quantitative data in three mixed methods studies were assessed as being of moderate (*n* = 1) or poor quality (*n* = 2) using the EPHPP tool. Of these, the study by Sahgal and Kimaiyo (2020) was assessed as being of borderline Strong/Moderate quality but ultimately received a global rating of Moderate due to a lack of Blinding. However, it is considered highly robust based on it being assessed as strong against almost every other domain of the EPHPP tool: Selection Bias (based on the study population being very likely representative of the target population); Confounders (based on the study controlling for different confounders during the analysis); Data Collection (based on validity and reliability of the tools used); and Withdrawals and Dropouts. There was also some rationale for rating the Research Design as strong, but the lack of a control group meant that it was assessed as Moderate quality. In contrast, the studies by Cifuentes et al. (2013) and i‐Works Research Ltd. (2013) were assessed as Weak against most of the domains of the tool, and thus as Weak overall. However, it is worth noting that most of the relevant data from these studies came from the qualitative strand of their methodologies, which were assessed as robust as stated above.

It was not possible to examine publication bias in the way outlined in the protocol owing to the small number of relevant, eligible studies identified. However, the evidence relating to different elements of implementation – including implementation factors and moderators – was consistent across both published and unpublished studies.

### 5.2 Synthesis of Results

#### 5.2.1 Analysis of Effectiveness (Objective I)

Only one identified study used an eligible research design to evaluate effectiveness. In this study, Kolbe (2019) used a non‐matched quasi‐experimental design with a treatment‐as‐usual condition to compare four cohorts of at‐risk youth (*n* = 113) aged 13–18 participating in a group‐based ‘after school enrichment’ programme in an unnamed East African City. This programme involved group activities that took place 5–6 days per week, including ‘field trips, organized recreational activities, voluntary community service, classes in religion and cultural issues, programming to increase participation in civil society and awareness of children's rights, health and hygiene training, job skills workshops, and a psycho‐educational support group’ (Kolbe 2019, 2). To graduate from this programme, youth had to participate in 50 sessions over a 3‐month period.

- Treatment as Usual: Group‐based programme only (*n* = 53, three lost at follow up).
- Alternative Treatment: Group‐based programme, complemented by weekly visits from a social worker to identify and address any barriers that young people might face in participating in the programme, facilitate access to relevant services, and, if necessary, mediate conflicts within the family (*n* = 60).

This study made use of a naturally occurring experiment: funding for the social work provision was only made available after the first cohort of youth had begun to engage with the programme, before being removed before a fourth cohort enroled. All participants in Cohorts 1 and 4, therefore, received the Treatment as Usual Condition, and all participants in Cohorts 2 and 3 received the alternative treatment. To examine the impact of the alternative treatment, Kolbe (2019) therefore compared the two groups on the following:

1. The number in each condition who had joined an armed group 9‐month post‐intervention (12 months after intake).
2. The number in each condition who graduated from the programme (based on attending a minimum of 50 sessions).

Those in the alternative treatment group were less likely to have joined an armed group at follow‐up (*n* = 2, 3.3%) than those in the treatment‐as‐usual group (*n* = 8, 16%). The authors report a Chi‐square test result of 5.295, *df* = 1, *p* = 0.021, which can be converted to an odds ratio of 0.181, 95% CI [0.0366, 0.8963], indicating a large effect.

Those in the alternative treatment group were also more likely to graduate from the programme, with Kolbe (2019) identifying a medium positive correlation between graduation and receipt of social work services: *r*(113) = 0.307, *p* < 0.001, 95% CI [0.13, 0.47].

Although the author did not identify specific mechanisms of change, they concluded that ‘the work of social workers to facilitate reintegration in education and work was well‐received and contributed to the success of the program overall’ (Kolbe 2019, 4).

However, caution is needed when interpreting the results of this study due to its serious risk of bias. Use of a treatment‐as‐usual comparator condition also means that the study does not provide robust evidence relating to the effectiveness of the intervention overall (i.e., compared to no treatment), only that a variation in treatment was associated with better outcomes than treatment‐as‐usual. This means there is insufficient evidence with which to conclude whether this intervention, or any other non‐criminal justice intervention, is effective in countering childhood and adolescent radicalisation.

Several limitations were also noted by the author, most notably that the intervention was ‘geographically and temporally limited to a particular circumstance, culture, political conflict, and population’ (Kolbe 2019, 5). The homogeneity of the group, in terms of language, religion, and nationality, was also seen as helping social workers in ‘addressing common problems without the distraction of constantly adapting to meet the diverse cultural needs of the individual participants’ (Kolbe 2019, 5). Taken together, this study provides some evidence of the positive impact of social work services, but the limitations stated here and above should be considered when reading the results (Table 13).

**Table 13:** EPHPP Assessment for non‐experimental primary research studies (*n* = 3) included in analysis of Objectives II and III.

Study	Selection bias	Study design	Confounders	Blinding	Data collection	Withdrawals and dropouts	Overall
Cifuentes et al. (2013)	Moderate	Weak	Weak	Weak	Weak	Strong	Weak
i‐Works Research Ltd. (2013)	Weak	Weak	Weak	Weak	Weak	Weak	Weak
Sahgal and Kimaiyo (2020)	Strong	Moderate	Strong	Weak	Moderate	Strong	Moderate

#### 5.2.2 Analysis of Implementation (Objective II)

Seven studies presented evidence relevant to Objective II (AEF 2018; Brett and Kahlmeyer 2017; Fisher et al. 2020; Foster 2010; i‐Works Research Ltd. 2013; Skiple 2020; Uhlmann 2017). These studies spoke to two important elements of implementation: whether programmes were implemented as intended (*n* = 6) and worked with relevant clients (*n* = 4). Relevant findings relating to these two themes are summarised in Table 14.

**Table 14:** Analysis of implementation – Key themes.

Theme	Key findings
Implemented as intended (*n* = 6)	Evaluations find intervention implementation generally aligns with the underlying theory of change or objectives, or with relevant standards or assumed good practices identified in the literature. Flexibility is important for ensuring that the intervention can adapt to specific or emerging needs.
Working with eligible clients (*n* = 4)	The ability of interventions to identify and engage with at‐risk or radicalised youth varies. Interventions may face challenges in identifying those most at risk or in need of support.

All relevant evidence was qualitative, drawn from qualitative (*n* = 2) and mixed methods (*n* = 5) studies. The overall strength of this evidence was considered of medium‐to‐high quality based on the quality assessments described above, with 4 studies scoring positively on 7 or 8 of the CASP domains, and 3 studies on 9 or all 10.

##### 5.2.2.1 Examining Whether Programmes Were Implemented as Intended

Six studies examined whether programmes were implemented as intended and/or in ways that aligned with an implicit or explicit theory of change or underlying programme logic (AEF 2018; Brett and Kahlmeyer 2017; Fisher et al. 2020; Foster 2010; i‐Works Research Ltd. 2013; Uhlmann 2017). These studies reported generally positive results overall, reporting that interventions were being implemented as intended, or in line with an underlying logic.

Two of these evaluations examined whether mentorship activities delivered through the STRIVE I and STRIVE II projects in Kenya were being implemented in ways that aligned with the theory of change that underpinned both projects: that identifying and mentoring (young) people who are at heightened risk of radicalisation will help to build their resilience to violent extremist narratives and recruitment (Brett and Kahlmeyer 2017; Fisher et al. 2020).^21^ Although neither evaluation produces robust evidence of effectiveness owing to the research designs they use, they find that mentorship activities were being delivered in ways that aligned with this underlying logic. First, Brett and Kahlmeyer (2017) concluded that the delivery of different STRIVE I activities had been ‘relevant to the aims, purpose and objectives of the project as set out in the terms of reference’ (p. 10), highlighting the mentorship strand as ‘closely targeting distinct groups that are at risk’ (p. 10), including youth. Similarly, Fisher et al. (2020) found ‘significant evidence to demonstrate that the [STRIVE II] programme is successful in supporting those at risk to become more resilient to violent extremism in ways that align well with the theory of change’, and that the mentoring component should be seen as ‘a tried and tested model’ that had ‘produced remarkable, positive effects on the lives of youth at risk of violent extremism’ (p. 7). These studies highlighted how a clearly defined and evidence‐informed theory of change can support implementation and evaluation. Indeed, Brett and Kahlmeyer (2017) concluded that ‘spelling out theories of change in the idea, design and baseline stage of each [project] will strengthen the scope for learning and results’ (p. 13).

Two evaluations similarly examined whether programmes were operating in ways that aligned with relevant evidence (AEF 2018) or professional standards (Uhlmann 2017) and drew positive conclusions. Both studies also highlighted the benefits of flexibility and of adjusting delivery in response to client needs. In the Netherlands, AEF (2018) concluded that the methodology underpinning Forsa and the Family Support Centre was solid – albeit, in need of further validation – and that intake processes ‘operate in accordance with a self‐developed methodology’ (p. 27). The programmes were also positively evaluated for adapting to the needs of their clients and establishing an in‐house mental health treatment team. Although ‘not specified in the original plans for the facility’, establishing this team had ultimately ‘proved necessary to provide further assistance with psychological and psychiatric issues and basic knowledge for the purposes of diagnosing mental health issues and slight mental impairments’ (AEF 2018, 51).

The evaluation of the BAMF Advice Centre in Germany was similarly positive about counselling work delivered by the Centre and its local partners, whilst also pointing to the importance of flexibility. Uhlmann (2017) notes there had been ‘a conscious desire not to restrict the range of options too much, so as to leave room for various approaches to develop’ when establishing the centre, which had left space to ‘try out various approach options’ whilst also preventing ‘the model project from being narrowed down to a particular approach prematurely’ (p. 47). Counselling procedures ‘have become more structured’ over time, and the evaluation was positive that procedures were being followed. That is, that the key steps in an ‘idealised’ case management process were ‘generally practiced in some form across the board at the counselling centres and among counsellors’ (Uhlmann 2017, 39). However, the evaluation also emphasised that the individualised nature of case work requires a flexible approach, and that case management work with every client will not and does not always need to follow every step in this process.

##### 5.2.2.2 Examining Whether Programmes Worked With Eligible Clients

Four evaluations assessed whether a programme identified and worked with relevant clients (AEF 2018; Brett and Kahlmeyer 2017; Fisher et al. 2020; Skiple 2020). Two of these studies reported highly positive findings in regard to the programme's reach:

– Fisher et al. (2020) concluded that the STRIVE II mentoring programme in Kenya had successfully identified and worked with at‐risk youth – defined using specific criteria set by the programme (Fisher et al. 2020).^22^
– Brett and Kahlmeyer's (2017) positive evaluation of the STRIVE I mentoring programme – also in Kenya – was based on the project having worked with around 100 youth, 10% of whom were ‘highly vulnerable’ or in the process of being radicalised – although the specific criteria used to assess individuals as being vulnerable or already in process of being radicalised is not stated.

Although the other two studies reported positive findings relating to reach, both AEF (2018) and Skiple (2020) identified challenges. In the Netherlands, AEF (2018) reported that the National Support Centre for Extremism (LSE) believed it was aware of a ‘large proportion’ of cases nationally that would be eligible for support through Forsa, whilst also noting that there was a ‘hard core of individuals’ that LSE felt that ‘they cannot address’ due to their unwillingness to participate voluntarily (p. 46). It also suggested that cases supported through the associated Family Support Centre might only be the ‘tip of the iceberg’, owing to what they perceived as a ‘cautious attitude towards active profiling’, and the possibility that ‘municipalities may wish to initiate their own programme’, rather than refer into an LSE programme (p. 46).^23^ This study, therefore, identifies challenges in identifying and recruiting cases that are explored in more detail in Section 5.2.4 below.

Skiple's (2020) evaluation of the Tolerance Project in Sweden noted that recruiting ‘the most “at‐risk” youth’ had proved challenging. This study also discussed the twin risks of ‘over and under identification’ that might have resulted from asking teachers and social workers to identify eligible youth. In this context, over identification relates to concerns that disproportionately high number of young people will be wrong identified as being at risk given that ‘it is not clear at an early age which youths will end up in extreme environments and which not’, and under identification to concerns that some proportion of young people who are at risk will not be identified due to ‘members of extreme groups … becoming less visible’ as far‐right milieus have evolved over time (Skiple 2020, 59). Issues of under‐ and over‐identification were commonly discussed in the wider corpus of studies and are discussed in the examination of implementation factors that follows below.

#### 5.2.3 Analysis of Implementation Factors (Objective III)

Twenty‐eight studies examined implementation factors that affected how different types of interventions functioned. Three types of implementation factors were identified. The first were structural factors relating to programme design and resourcing as described in Table 15. The second set of factors was related to methods for identifying and engaging with youth and/or their families as outlined in Table 16. The third set of factors related to specific practices that facilitated practitioners working with young people and their families (Table 17). Evidence relating to these different types of factors, including whether and how they impacted the domains of the RE‐AIM framework, is discussed below.

**Table 15:** Structural or systemic implementation factors.

Implementation factor	Sub‐theme
Knowledge and expertise (*n* = 21)	– Subject matter expertise (*n* = 11) – Lived experience of extremism (*n* = 3) – Practice‐based knowledge (*n* = 10) – Mental health and neurodiversity (*n* = 12) – Multidisciplinary approach (*n* = 12) – Training and learning (*n* = 15) – (Perceived) Lack of knowledge (*n* = 4)
Community working (*n* = 15)	N/A
Multi‐agency and partnership working (*n* = 19)	– Efficiency (*n* = 5) – Facilitating access to support (*n* = 10) – Relational processes (*n* = 9) – Effective coordination (*n* = 5) – Shared objectives and understanding (*n* = 9) – Engaging partners (*n* = 6) – Information‐sharing (*n* = 8)
Police involvement (*n* = 13)	N/A
Staff supervision and support (*n* = 7)	– Institutional support (*n* = 5) – Supervision and quality assurance (*n* = 4)
Resourcing programmes (*n* = 15)	– Time (*n* = 6) – Funding and resources (*n* = 13) – Staffing (*n* = 6)

**Table 16:** Implementation factors relating to identifying and engaging clients.

Implementation factor	Sub‐theme
Identifying eligible clients (*n* = 16)	– Working with partners (*n* = 12) – Accessibility of programmes (*n* = 9) – Clearly defined target audience (*n* = 10)
Engaging eligible clients (*n* = 13)	– Accurate and consistent decision‐making (*n* = 9) – Intake process and procedures (*n* = 8) – Accessibility of services (*n* = 4) – Enroling clients (*n* = 10)

**Table 17:** Implementation factors relating to working with clients.

Implementation factor	Sub‐theme
Relational processes (*n* = 20)	– Establishing relationships and trust (*n* = 16) – Matching practitioners and clients (*n* = 10) – Practitioner commitment (*n* = 6)
Client‐centred approach (*n* = 18)	– Individualised approach (*n* = 16) – Developmentally appropriate (*n* = 7) – Cultural sensitivity (*n* = 7)
Ways of working (*n* = 14)	– Dialogue and discussion (*n* = 9) – Empowerment and agency (*n* = 9)
Contextualised approach (*n* = 17)	– Working with families (*n* = 14) – Socio‐ecological approach (*n* = 10)

##### 5.2.3.1 Structural and Systemic Factors

This section examines structural or systemic implementation factors that impacted the overall functioning of interventions as outlined in Table 15 above. Facilitators and barriers associated with each type of structural or systemic factor are shown in Table 18 below.

**Table 18:** Implementation factors relating to structural features.

Factor	Facilitators	Barriers
*Knowledge and expertise*		
RE‐AIM factors: Implementation; Effectiveness; Adoption; Maintenance		
Subject matter expertise	1. Availability of specialist staff 2. Prior experience working with radicalisation cases 3. Ideological expertise in relevant cases 4. Nuanced, tailored approach to assessing the relevance of ideology	
Lived experience of extremism	1. Those with lived experience can have greater credibility	1. Concerns that some of those with lived experience may retain extremist views
Practice‐based knowledge	1. Previous experience in adjacent fields 2. Cultural, linguistic and age‐related understanding	
Mental health and neurodiversity	1. Availability of specialist mental health and psychological assessment and support, including trauma‐informed approaches	
Multidisciplinary approach	1. Structure for bringing multidisciplinary teams together, either through in‐house teams or partnership working 2. Increased access to different services able to meet clients' needs 3. Ability of the team to perform a range of functions within an intervention	
Training and development	1. Supports frontline practitioners in building relationships with clients 2. Trauma‐informed training 3. Ongoing training to ensure able to respond to emerging issues 4. Building knowledge and confidence, especially for those inexperienced in CVE 5. Training on: radicalisation indicators; working with radicalisation cases; broaching radicalisation with young people/families 6. Knowledge sharing through work shadowing; knowledge exchange within and across organisations; sitting in on one another's meetings 7. Experiential learning, for example, through multi‐agency case reviews 8. Multi‐professional training 9. Practical training tools such as case studies, face‐to‐face delivery, video‐based scenarios, role‐playing scripts, and decision trees 10. Train the trainer models enabling information to be cascaded to staff	1. Lack of practical guidance 2. Training that provokes fear or concern over profiling communities 3. Low case numbers limit experiential learning 4. Uneven access to training across regions 5. Limited time and resources
Perceived lack of knowledge		1. Lack of knowledge and confidence is particularly challenging for those with little experience of CVE 2. Anxiety caused by a perceived lack of knowledge 3. Difficulties engaging with partners due to a lack of knowledge
*Community working*		
RE‐AIM: Implementation		
Community working	1. Partnership working across sectors 2. Equitable relationships between government and partners 3. Enables trust and community buy‐in 4. Involving community leaders and members 5. Community consultation and ongoing community engagement enhance the ability to meet local needs 6. Acts as an alternative means of support if a young person is reluctant to work with government‐led programmes	1. Excessive monitoring by governments and funders may lead to stigmatisation 2. Reputational risks for community organisations due to working with government agencies
*Multi‐agency partnership*		
RE‐AIM: Reach; Implementation; Effectiveness; Maintenance		
Efficiency	1. Multi‐agency structures help mobilise and build awareness of local resources 2. Reduces duplication of effort 3. Enables individual partners to overcome resource issues	
Facilitating access to support	1. Reduces the need for individuals to navigate multiple systems 2. Increases the likelihood of individuals being connected to the right people	
Relational processes	1. Trust, equity and stability characterise positive multi‐agency working 2. Histories of collaboration enable constructive relationships 3. Interpersonal relationship support partnership working 4. Institutionalising informal relationships can mitigate the risks of temporary interpersonal relationships	1. Staffing shortages can undermine the stability and sustainability of partnerships
Effective coordination	1. Supported by organisations that interface between a range of government and civil society actors 2. Clearly defined roles for different institutions with a shared understanding of where responsibility for managing cases lies	1. Disagreements over which agency is best placed to lead on cases involving young people
Shared objectives and understanding	1. Mutual understanding of different partners' priorities, roles and responsibilities	1. Disagreements over which youth should be the focus of programmes 2. Competing priorities across organisations, for example, between those focused on rehabilitation and those principally concerned with public safety
Engaging partners	1. Ensuring partners understand their role within CVE structures 2. Enabling partners to understand the value they are bringing to the programme	1. Lack of clarity over a partner's role 2. Lack of buy‐in and belief in the need for a programme
Information sharing	1. Effective and efficient information‐sharing protocols 2. Memoranda of understanding to agree on information‐sharing processes 3. Trusting interpersonal relationships supported information sharing	1. Discomfort or unwillingness to share information, particularly with the police 2. Difficulties in gaining information from the police due to the need for security clearances and sensitivity over sharing information 3. Privacy legislation can impede information sharing
*Police involvement*		
RE‐AIM: Reach; Implementation		
Police involvement	1. Involving police in joint screening and risk assessment processes 2. Clear criteria and processes for contacting law enforcement when risk thresholds are reached 3. Transparency with clients and communities around what would trigger a report enables trust and confidence 4. Limiting the availability of information about a case within police systems 5. Contextualised assessment of the most appropriate and proportionate role for the police	1. Reporting to the police when a risk threshold is reached can undermine relationships of trust with clients and potentially stigmatise youth 2. Anxiety caused by police involvement, especially in voluntary programmes
*Staff supervision and support*		
RE‐AIM: Implementation		
Institutional support	1. Discussing cases with colleagues can provide alternative perspectives and support decision‐making 2. Partnering new staff with senior colleagues 3. Protocols setting out de‐escalation techniques 4. Regular group reflective practice meetings help build confidence and capability 5. Risk assessment to identify and mitigate risks to staff and clients	
Supervision and quality assurance	1. Peer supervision and discussion 2. Reflective group discussions 3. Case review processes, including independent reviews to support quality assurance practices 4. Process supervisors or institutional leads maintain consistency and quality of delivery 5. Establishing quality standards alongside adequate training	
*Resourcing programmes*		
RE‐AIM: Implementation; Maintenance		
Time	1. Sufficient time available for practitioners to work with clients 2. The time made available to work with a client should be proportionate to the need	1. Time‐limited programmes cause constraints 2. Large caseloads can reduce the time available to support clients 3. Practitioner burnout caused by time constraints 4. The open‐ended nature of some tailored approaches can see support maintained unnecessarily 5. Risk of programmes being overwhelmed if clients become too dependent 6. Short‐term funding horizons create time constraints and may mean the necessary structures and partnerships are not in place when needed
Funding	1. Sustainable, long‐term funding 2. Planning exit strategies to limit the impact of projects coming to an end, for example, formalising structures and partnerships able to outlast the project 3. Identifying routes to financial sustainability, including receiving support from multiple sources 4. National or local government supervision and coordination of local work helps improve quality and efficiency 5. Bottom‐up approaches that seek to empower organisations	1. Short‐term, unstable funding structures negatively impact programme capacity and sustainability 2. Sustainable change is harder to deliver when time‐limited funding arrangements mean interventions end prematurely 3. Short‐term approaches can be unable to address the structural issues facing youth 4. Over‐reliance on government funding
Staffing	1. Professionalisation of the field and professional standards 2. Sufficient funding to support recruitment and remuneration of skilled professionals	1. Recruiting and retaining staff 2. High workloads, low pay and short‐term contracts informed by time‐limited funding 3. Lack of staff retention inhibits the development of best practice and leads to loss of expertise and capacity 4. Quality and motivation of staff can vary

###### 5.2.3.1.1 Knowledge and Expertise

Twenty‐one studies examined implementation factors relating to knowledge and expertise (AEF 2018; Cherney 2024; Cherney et al. 2018; Cherney et al. 2022a; Chisholm and Coulter 2017; Davies 2023; Fisher et al. 2020; Gielen 2020; Grossman and Barolsky 2019; i‐Works Research Ltd. 2023; Joyce and Lynch 2017; Kolbe 2019; Langdon‐Shreeve and Nickson 2021; Oberg et al. 2023; Ottis 2016; Rousseau et al. 2024; Sahgal and Kimaiyo 2020; SPLC and PERIL 2021; Thomas et al. 2017; Uhlmann 2017; Weine et al. 2018).

Evidence pointing to the impact of knowledge and expertise was primarily drawn from qualitative analysis presented in qualitative (*n* = 13) or mixed‐methods (*n* = 7) studies. The qualitative methodology used in these studies was assessed as being high quality using the CASP tool, with over half (*n* = 12) scoring positively on at least 9 of the 10 domains. The strength of evidence relating to this factor was therefore particularly robust based on the large number of high‐quality studies that examined how different forms of knowledge and expertise – and associated knowledge‐building activities – facilitated implementation.

This evidence, in turn, highlights how the availability (or lack thereof) of knowledge, expertise, and experience impacted multiple domains of the RE‐AIM framework. Different forms of practice‐based and subject matter expertise were needed to support interventions in performing crucial functions and providing relevant forms of support (*Implementation)*, and in turn, whether participants perceived programmes as likely to be effective (*Effectiveness*). Training and knowledge‐building activities were crucial in building relevant knowledge and expertise, as well as the confidence and awareness needed to support less‐experienced practitioners in delivering counter‐radicalisation work (*Adoption*). Finally, establishing knowledgeable, experienced, and well‐functioning teams was seen as crucial to programme sustainability (*Maintenance*).

####### Subject Matter Expertise

Eleven studies highlighted the importance of interventions employing or working with frontline practitioners with expertise or experience relevant to (countering) radicalisation (AEF 2018; Cherney et al. 2018; Cherney et al. 2022a; Chisholm and Coulter 2017; Gielen 2020; Langdon‐Shreeve and Nickson 2021; Oberg et al. 2023; Ottis 2016; Thomas et al. 2017; Uhlmann 2017; Weine et al. 2018). Although delivery commonly relied on partnership working with non‐specialists (see below), the importance of employing or working with ‘specialist staff’ was emphasised (AEF 2018; Cherney et al. 2018; Chisholm and Coulter 2017; Langdon‐Shreeve and Nickson 2021). Importantly, such practitioners were not necessarily expected to be subject matter experts, and some previous experience of working with radicalisation cases or in a counter‐radicalisation specific role was itself seen as a useful form of knowledge (AEF 2018; Chisholm and Coulter 2017; Langdon‐Shreeve and Nickson 2021). Some forms of subject matter expertise – particularly in relation to ideology and religion – were, however, identified as being particularly important (AEF 2018; Chisholm and Coulter 2017). Although the danger of overstating the relevance of ideology was also noted. Practitioners stressed the importance of taking a nuanced approach when considering the impact of ideology on different cases, and of tailoring any discussion relating to ideology accordingly (Adams et al. 2023). This observation speaks to the importance of taking a client‐centric approach – as discussed later in this section.

####### Lived Experience of Extremism

Three studies discussed programmes using former extremists or mentors with ‘experience of radicalisation themselves’ (Chisholm and Coulter 2017, 31) when working with at‐risk or radicalised youth (Davies 2023; Joyce and Lynch 2017). Although evidence relating to this implementation was inconclusive, opportunities and challenges associated with employing such individuals were noted.^24^ Although the credibility and insider knowledge held by those with lived experience were identified as potentially useful, some practitioners expressed ‘nervousness’ around engaging with former extremists owing to concerns that they might continue to hold residual extremist views (Davies 2023).

####### Practice‐Based Knowledge

Ten studies highlighted the importance of practice‐based knowledge and experience not specific to CVE (AEF 2018; Cherney et al. 2018; Cherney et al. 2022b; Gielen 2020; Grossman and Barolsky 2019; Langdon‐Shreeve and Nickson 2021; Oberg et al. 2023; Ottis 2016; Thomas et al. 2017; Weine et al. 2018). Because practitioners interviewed in these studies often pointed to a perceived overlap between counter‐radicalisation work with young people and other fields of practice, these studies emphasised the utility of practitioners drawing on their previous professional background or experience when engaging in this space, including experience gained in fields including ‘care provision’ (AEF 2018; Gielen 2020); social work and safeguarding (Ottis 2016; Langdon‐Shreeve and Nickson 2021); and youth work (Langdon‐Shreeve and Nickson 2021; Thomas et al. 2017). The importance of skills and knowledge including ‘cultural and language skills’ (Cherney et al. 2018); an understanding of ‘youth culture’ and being able ‘to share the same language and ideas with a client’ (Cherney et al. 2022b 40) was also cited, particularly in relation to building a trusted relationship with a young person – a point that is explored in more detail in a later section of the analysis.

####### Mental Health and Neurodiversity

Twelve studies highlighted the importance of knowledge relating to mental health and neurodiversity (AEF 2018; Cherney et al. 2022a; Cherney 2024; Davies 2023; Fisher et al. 2020; Gielen 2020; Grossman and Barolsky 2019; Langdon‐Shreeve and Nickson 2021; Oberg et al. 2023; Rousseau et al. 2024; Uhlmann 2017; Weine et al. 2018). Several noted a recent rise in complex cases presenting with mental health needs or needs relating to neurodivergence (Langdon‐Shreeve and Nickson 2021; Rousseau et al. 2024; Uhlmann 2017). It was therefore unsurprising that studies cited the importance of clients being able to access mental health or psychological support (AEF 2018; Cherney 2024; Fisher et al. 2020; Grossman and Barolsky 2019; Langdon‐Shreeve and Nickson 2021; Uhlmann 2017; Weine et al. 2018) or support from autism specialists (Davies 2023) when needed. Studies also discussed the topic of trauma, and/or the need for trauma‐informed approaches (Cherney et al. 2022a; Gielen 2020; Rousseau et al. 2024).

####### Multidisciplinary Approach

The above themes speak to the importance of programmes being underpinned by a multidisciplinary approach that brings together different kinds of knowledge to support clients. This was highlighted in 12 studies (AEF 2018; Cherney et al. 2022a; Cherney 2024; Fisher et al. 2020; Gielen 2020; Grossman and Barolsky 2019; Kolbe 2019; Langdon‐Shreeve and Nickson 2021; Ottis 2016; Thomas et al. 2017; Uhlmann 2017; Weine et al. 2018). Models of multidisciplinary working included embedding specialist functions within programmes, most notably in‐house or attached mental health providers (AEF 2018; Fisher et al. 2020); and partnerships with other external service providers – such as educational professionals, social workers, youth workers, law enforcement professionals, and community or non‐governmental organisations, often as part of multi‐agency working structures (see section on multi‐agency working below). Regardless of the model used, multidisciplinary approaches were seen as crucial in ensuring that clients can access different services related to their specific needs, particularly in the context of case management, mentoring, and other tailored approaches (AEF 2018; Cherney et al. 2022a; Cherney 2024; Fisher et al. 2020; Grossman and Barolsky 2019; Kolbe 2019; Langdon‐Shreeve and Nickson 2021; Ottis 2016; Uhlmann 2017; Weine et al. 2018).

Studies also highlighted that different skillsets and kinds of expertise are needed to perform different functions within interventions, ranging from supervisory or case manager functions through to the delivery of particular forms of support (Grossman and Barolsky 2019; Gielen 2020). For example, Gielen's (2020) analysis of family support groups in the Netherlands identifies a number of roles that can help family support groups function effectively: a process supervisor; trauma/grief counsellors; and external experts and professional care providers (pp. 138–193). The sustainability of programmes can also be enhanced through the ‘maintenance and expansion of expertise’ possessed by individuals and teams (Uhlmann 2017, 46).

####### Training and Learning

Fifteen studies discussed the implementation and/or importance of training and learning in supporting implementation (AEF 2018; Cherney et al. 2022a; Cherney et al. 2018; Chisholm and Coulter 2017; Fisher et al. 2020; Gielen 2020; Grossman and Barolsky 2019; i‐Works Research Ltd. 2013; Langdon‐Shreeve and Nickson 2021; Oberg et al. 2023; Sahgal and Kimaiyo 2020; SPLC and PERIL 2021; Thomas et al. 2017; Uhlmann 2017; Weine et al. 2018).

The importance of training specialist CVE practitioners, particularly those working directly with at‐risk and radicalised youth, such as counsellors and mentors, was noted in several studies (AEF 2018; Fisher et al. 2020; Oberg et al. 2023; Thomas et al. 2017; Uhlmann 2017). Practitioner feedback was generally positive about the impact of such training. However, in one study that reported more negative feedback overall, a perceived lack of practical guidance was seen as a particular issue in the training (Chisholm and Coulter 2017).

Several studies emphasised that training is an ongoing process, highlighting the importance of staff regularly updating their knowledge in response to new or emerging threats or changes in programme caseload (AEF 2018; Uhlmann 2017). For example, Uhlmann (2017) commended counsellors working for the BAMF Advice Centre in Germany for continuing to pursue training and qualifications on relevant topics.

Training and awareness‐building activities were seen as particularly important in building the knowledge and confidence of practitioners who might be asked to perform a CVE function outside of their usual day‐to‐day role, for example, youth workers, social workers, or mental health professionals (Cherney et al. 2022a; Cherney et al. 2018; Chisholm and Coulter 2017; Grossman and Barolsky 2019; Langdon‐Shreeve and Nickson 2021; SPLC and PERIL 2021; Weine et al. 2018). Practical training on topics such as indicators of radicalisation; working with radicalisation cases; and approaching the topic of radicalisation with young people and/or families was cited as being particularly useful (Chisholm and Coulter 2017; Langdon‐Shreeve and Nickson 2021; SPLC and PERIL 2021).

However, the content or tone of training benefits from not inadvertently making staff fearful or contributing to the profiling of communities (Cherney et al. 2018). Providing practical and subject matter training for community actors and organisations in contexts where they are asked to perform a CVE function is also important (Grossman and Barolsky 2019). Practitioners in Australia also caution against a ‘top‐down’ approach to training and support, stressing the importance of training being ‘bottom up in orientation and focused on empowering organisations and understanding their unique needs and capacity to respond to youth radicalisation and violent extremism’ (Cherney et al. 2018, 13).

The sharing of knowledge between colleagues and institutions was seen as useful. Relevant practices included newly qualified staff shadowing more experienced colleagues (Langdon‐Shreeve and Nickson 2021); knowledge sharing and transfer within and across different institutions (AEF 2018; Gielen 2020; i‐Works Research Ltd. 2013); and working practices that enable partners to learn from (and about) one another – for example, sitting in on each other's meetings; or conducting regular multi‐agency case review meetings to discuss previous cases (Langdon‐Shreeve and Nickson 2021). The last example speaks to the potential role of ‘experiential learning’ in helping to build knowledge and confidence in working in this space. However, this same study also noted that opportunities for experiential learning are limited in contexts where there are low numbers of radicalisation cases, and for those practitioners who have limited involvement in such cases.

Practitioners interviewed in several studies identified a number of approaches to training they saw as beneficial. For example, Langdon‐Shreeve and Nickson (2021) noted a preference for multi‐professional training amongst social workers in the United Kingdom; the use of real‐life examples; and face‐to‐face delivery, whilst SPLC and PERIL (2021) noted that participants in their United States‐based programme recommended ‘formats such as video‐based scenarios, role playing scripts, conversation models, and decision trees’ (p. 6). Langdon‐Shreeve and Nickson (2021) also identified challenges, including that access to relevant training was inconsistent across regions and the limited time and resources that institutions might have for training. This study, therefore, highlighted the benefits of senior staff relaying lessons from any in‐depth training that may have less day‐to‐day relevance to more junior staff, but which might still contain useful information.

####### Challenges Associated With a (Perceived) Lack of Knowledge

A perceived lack of knowledge and confidence was identified as a barrier to implementation in four studies, further emphasising the need for training (AEF 2018; Cherney et al. 2018; Chisholm and Coulter 2017; Langdon‐Shreeve and Nickson 2021). This challenge was seen as acute when seeking to engage non‐CVE specialists in CVE work, particularly those with little to no direct experience of working with radicalisation cases (AEF 2018; Cherney et al. 2018; Chisholm and Coulter 2017; Langdon‐Shreeve and Nickson 2021). This lack of experience (and in turn confidence) was seen as potentially contributing to a sense of anxiety and as hampering efforts to engage partners such as social workers and schools in this work (AEF 2018; Langdon‐Shreeve and Nickson 2021).

###### 5.2.3.1.2 Community Working

Fifteen studies pointed to the importance of working and engaging with communities, and community and civil society partners, when working with young people and/or families in a CVE space (Badurdeen and Ndenyele 2024; Brett and Kahlmeyer 2017; Cherney et al. 2018; Davies 2023; Fisher et al. 2020; Gielen 2020; Grossman and Barolsky 2019; i‐Works Research Ltd. 2013; Langdon‐Shreeve and Nickson 2021; Osorno Hernandez 2021; Ottis 2016; SPLC and PERIL 2021; Thomas et al. 2017; Uhlmann 2017; Weine et al. 2018).

All data pointing to the importance of community engagement and partnerships were drawn from qualitative studies (*n* = 11) or the qualitative component of mixed methods studies (*n* = 4). The qualitative methodologies of these studies were assessed as medium to high quality, with almost all scoring positively against 8 (*n* = 5), 9 (*n* = 3), or 10 (*n* = 5) of the CASP domains. This evidence was therefore considered robust and spoke most directly to the *Implementation* domain of the RE‐AIM framework, given that partnering with communities and community actors was often crucial to delivery.

Several interventions specifically relied on such partnerships, whereby governments or other funding agencies needed community or civil society actors to implement programmes or deliver support to young people and families (e.g., Brett and Kahlmeyer 2017; Fisher et al. 2020; i‐Works Research Ltd. 2013; Langdon‐Shreeve and Nickson 2021; Uhlmann 2017). Leveraging community actors and organisations was therefore seen as an appropriate approach (Brett and Kahlmeyer 2017; Weine et al. 2018). Governments and other funders often play an important coordination role within such partnerships (Brett and Kahlmeyer 2017; Gielen 2020; Grossman and Barolsky 2019). For example, Grossman and Barolsky (2019) found a high level of support for an ‘integrated government‐support model’ for female and child returnees amongst stakeholders in Australia. This would combine ‘informal community‐based social support’ and ‘localised agency‐led social services and educational support’ with ‘government‐based risk monitoring and management’ (p. 102). Participants emphasised the need for equity in any government‐community partnership – a point also stressed by Brett and Kahlmeyer (2017) – calling for a ‘two‐way partnership in which communities and government have equal standing and are willing both to listen to each other and engage in mutual recognition’ (Grossman and Barolsky 2019, 87).

However, some stakeholders interviewed for this study made the case for this work to be more ‘community‐led’ so that it would ‘maintain rather than jeopardise its credibility’ (Grossman and Barolsky 2019, 88). This speaks to a broader challenge, discussed in more detail in the section on funding and financing, whereby some community actors prefer to remain independent from government owing to potential reputational concerns linked to accepting government funding (Davies 2023; Osorno Hernandez 2021). A related challenge was that excessive monitoring of community organisations might also be seen as potentially contributing to stigmatisation (Badurdeen and Ndenyele 2024).

Partnering with communities and community actors was believed to help establish trust and community buy‐in (Badurdeen and Ndenyele 2024; Brett and Kahlmeyer 2017; Thomas et al. 2017), and so involving individuals from local communities in leadership and delivery functions was considered helpful (Badurdeen and Ndenyele 2024; Brett and Kahlmeyer 2017; Fisher et al. 2020). Consultation with local communities early in the design and implementation of programmes, and ongoing community engagement, were also cited as helping to ensure that programming meets local needs and addresses local concerns (Ottis 2016; SPLC and PERIL 2021; Thomas et al. 2017; Weine et al. 2018).

Community‐led programming was also considered useful in cases where a young person or family was reluctant to engage with government‐led programmes, as it provided an alternative mechanism for supporting them (Davies 2023; Langdon‐Shreeve and Nickson 2021). Finally, studies emphasised the importance of leveraging local community resources when working with youth and their families: for example, by drawing on approaches that ‘were community located or drew on positive peer influences’ (Cherney et al. 2018, 9) or empowering local communities to support the longer‐term reintegration of young people and their families (Grossman and Barolsky 2019). Such approaches are discussed in a later section examining socio‐ecological models.

###### 5.2.3.1.3 Multi‐Agency and Partnership Working

The functioning of different forms of multi‐agency and partnership working was examined in nineteen studies (AEF 2018; Brett and Kahlmeyer 2017; Cherney et al. 2022a; Cherney 2024; Chisholm and Coulter 2017; Cifuentes et al. 2013; Davies 2023; Foster 2010; Gielen 2020; Grossman and Barolsky 2019; i‐Works Research Ltd. 2013; Kolbe 2019; Langdon‐Shreeve and Nickson 2021; Oberg et al. 2023; Osorno Hernandez 2021; Ottis 2016; Rousseau et al. 2024; Uhlmann 2017; Weine et al. 2018). Partnerships varied in regard to:

– Geographical Focus: Although partnerships were localised, the delivery of services delivered by some programmes, including Forsa in the Netherlands (AEF 2018) and the BAMF Advice Centre in Germany (Uhlmann 2017), relied on collaboration across regions.
– Complexity: Some studies examined relationships between small numbers of partners, for example, between schools and a specific intervention provider (Davies 2023; i‐Works Research Ltd. 2013), whereas other networks were larger. For example, case management programmes such as Channel in the United Kingdom (Chisholm and Coulter 2017; Langdon‐Shreeve and Nickson 2021) were supported by complex multi‐agency partnerships between different sectors. These included the police, education, social work, education, and healthcare, which support different processes including client identification, assessment, and the delivery of intervention plans.
– Actors: The actors involved in partnerships differed. For example, whilst some studies examined multi‐agency collaboration between public agencies (e.g., Chisholm and Coulter 2017; Langdon‐Shreeve and Nickson 2021), others examined the functioning of government partnerships with civil society and community actors as described above (e.g., Grossman and Barolsky 2019).

Data examining the impact of multi‐agency and partnership working was primarily drawn from qualitative studies (*n* = 11) or the qualitative component of mixed methods studies (*n* = 7). The quality of these qualitative research designs was high, with half (*n* = 10) of the studies assessed using the CASP framework scoring positively on 9 or more of the 10 domains. This qualitative data were supplemented by quantitative data drawn from survey data (*n* = 2), secondary analysis of programme data (*n* = 5); and a quasi‐experimental analysis of programme completion rates (*n* = 1). Although the quantitative research designs were assessed as being weak (*n* = 2) or as having a serious risk of bias (*n* = 1), when considered as a whole, the evidence relating to partnership working was strong.

Effective and efficient partnership working was seen as positively impacting most domains of the RE‐AIM framework: helping programmes to identify and engage eligible clients *(Reach*); facilitate the performance of key intervention functions (*Implementation*), including the delivery of relevant forms of (multidisciplinary) support seen as crucial to producing positive intervention outcomes (*Effectiveness*); and, help to ensure the sustainability of programmes and best practice over longer time periods (*Maintenance*).

####### Efficiency

Five studies highlighted how partnership working was believed to improve the efficiency of interventions (Gielen 2020; Grossman and Barolsky 2019; Osorno Hernandez 2021; Uhlmann 2017; Weine et al. 2018). These studies discussed how multi‐agency partnerships provided an efficient structure for mobilising and building awareness of existing local resources (Grossman and Barolsky 2019; Weine et al. 2018); helped to avoid duplication of effort (Osorno Hernandez 2021) across different partners; and might help individual partners overcome resource issues (Gielen 2020). In this way, a multi‐agency network can be seen as ‘more than the sum of its actors’ (Uhlmann 2017, 21).

####### Facilitating Access to Support

Ten studies discussed how multi‐agency and partnership working was perceived to improve the efficacy of interventions, most commonly by facilitating clients' access to different types of (multidisciplinary) support (AEF 2018; Cherney 2024; Cifuentes et al. 2013; Davies 2023; Gielen 2020; Grossman and Barolsky 2019; Kolbe 2019; Langdon‐Shreeve and Nickson 2021; Ottis 2016; Uhlmann 2017). Other, more practical benefits of intervention work were noted in these studies. For example, Grossman and Barolsky (2019) noted that having access to a broad range of services through a single programme would benefit female and child returnees in Australia who might otherwise have to ‘navigate multiple, often complex delivery systems’ (Grossman and Barolsky 2019, 66), whilst Langdon‐Shreeve and Nickson's (2021) analysis of local Prevent work with young people and their families in the United Kingdom suggested that having ‘[a] wide pool of professionals who are confident and skilled in addressing radicalisation means a higher chance of finding someone who is best placed to engage’ a young person or their family (p. 67).

####### Relational Processes

Relational processes between institutions and individuals were identified as crucial facilitators of multi‐agency and partnership working in nine studies (AEF 2018; Brett and Kahlmeyer 2017; Chisholm and Coulter 2017; Grossman and Barolsky 2019; i‐Works Research Ltd. 2013; Langdon‐Shreeve and Nickson 2021; Osorno Hernandez 2021; Thomas et al. 2017; Uhlmann 2017). Positive *institutional relationships* were marked by trust, equity, and stability (Brett and Kahlmeyer 2017; Grossman and Barolsky 2019; Osorno Hernandez 2021; Thomas et al. 2017; Uhlmann 2017). Several studies emphasised that a history of collaboration helped to establish such relationships (AEF 2018; i‐Works Research Ltd. 2013; Osorno Hernandez 2021; Uhlmann 2017). For example, AEF (2018) notes that LSE had come to be seen as an institution that ‘keeps its promises’ and was now highly valued.

Strong *inter‐personal* relationships were also important to partnership working across interventions (Chisholm and Coulter 2017; Langdon‐Shreeve and Nickson 2021; Osorno Hernandez 2021). However, such relationships were vulnerable to staffing changes or funding cuts, which could in turn undermine the stability and sustainability of partnerships (Osorno Hernandez 2021). One response recommended by Langdon‐Shreeve and Nickson (2021) in an analysis of Prevent work in the United Kingdom involved institutionalising ‘informal, individual relationships’ (p. 43) by creating specialist roles within institutions that would ‘act as points of contact and conduits for joint working’ (p. 64). This, they argued, has the potential to overcome a reliance on informal relationships, which had led to inconsistencies in the nature and scope of collaboration within different local structures.

####### Effective Coordination

The importance of effective coordination at the strategic and/or operational level was noted by five studies (AEF 2018; Grossman and Barolsky 2019; Chisholm and Coulter 2017; Langdon‐Shreeve and Nickson 2021; Uhlmann 2017). For example, Uhlmann (2017) emphasised the crucial function that the national BAMF Advice Centre played in coordinating the work of local partners, noting that it was ‘optimally positioned’ for such a role, thanks to its position at 'the interface between the relevant governmental and civil‐society actors at both the national and regional level’ (Uhlmann 2017, 23).

Clearly defining the roles of specific institutions was seen as important, particularly clarifying which partner has overall responsibility for leading local work, or managing cases (AEF 2018; Grossman and Barolsky 2019; Langdon‐Shreeve and Nickson 2021). However, Langdon‐Shreeve and Nickson (2021) highlighted that UK partners might disagree on which agency is best placed to lead on cases involving children and adolescents. Although several organisations involved in Prevent work felt that children's social care is best positioned to lead on such cases, children's social workers argued that in some cases, ‘social care involvement might not be necessary or appropriate’ (p. 63).

####### Shared Objectives and Understanding

A related point discussed in nine studies was the importance of partners having shared objectives and understanding (AEF 2018; Cherney et al. 2022; Chisholm and Coulter 2017; Davies 2023; Grossman and Barolsky 2019; Langdon‐Shreeve and Nickson 2021; Oberg et al. 2023; Rousseau et al. 2024; Uhlmann 2017). However, partners might disagree over, for example, which youth should be the focus of a programme (Davies 2023) or might have competing priorities that reflect the nature of their day‐to‐day work. Most commonly, the research drew a distinction between professionals whose focus is on rehabilitation and care, and those focused on public safety (Oberg et al. 2023; Langdon‐Shreeve and Nickson 2021; Uhlmann 2017). However, such challenges were not restricted only to this particular type of partnership. For example, clinicians in Canada interviewed by Rousseau et al. (2024) emphasised ‘the difficulties of bringing together and establishing a consensus’ within and across agencies, including health care providers, youth protection workers, and schools (p. 694). Although it would be expected for partners to have competing priorities or perspectives, studies emphasised the importance of partners developing a ‘mutual understanding’ (Uhlmann 2017) and better understanding each other's roles, responsibilities and objectives, or the services that they provide (AEF 2018; Chisholm and Coulter 2017; Grossman and Barolsky 2019; Langdon‐Shreeve and Nickson 2021); for example, through multi‐agency training or knowledge exchange.

####### Engaging Partners

Ensuring that different partners understand the importance of their own role within multi‐agency CVE structures was also cited as important when trying to establish local partnerships. Six studies identified potential challenges when trying to engage local partners (Cherney et al. 2022a; Cifuentes et al. 2013; Davies 2023; Foster 2010; i‐Works Research Ltd. 2013; Langdon‐Shreeve and Nickson 2021). These studies highlighted that efforts to partner with local institutions can be challenging when they are ‘unsure about the purpose of their input and the value they could add to the process’ (Cifuentes et al. 2013, 322; Langdon‐Shreeve and Nickson 2021; Cherney et al. 2022a) or when unconvinced about the need for a specific programme (Davies 2023; Foster 2010). The importance of partners adding value to these partnerships was also noted. For example, the i‐Works Research Ltd. (2013) evaluation of the Think Project – an educational intervention that was delivered to young people in Wales – stressed the importance of ‘getting all of the needed agencies engaged’ and of ‘moving those agencies which are “observers” into agencies that are taking real action to move the Project on to a sustainable footing’ (p. 9). This highlights the importance of not just sustaining, but improving the depth of collaboration over time – a point also noted by Langdon‐Shreeve and Nickson (2021) in calling for a shift away from a ‘reactive’ multi‐agency approach towards one that was more ‘proactive’ in the United Kingdom.

####### Information Sharing

The importance of effective and efficient information sharing between partners was noted in eight studies (AEF 2018; Cherney et al. 2022a; Chisholm and Coulter 2017; Foster 2010; Grossman and Barolsky 2019; Langdon‐Shreeve and Nickson 2021; Ottis 2016; Uhlmann 2017). This was seen as helping to reduce delays (Langdon‐Shreeve and Nickson 2021); improve responsivity to emerging risks and threats (Grossman and Barolsky 2019); facilitate the delivery of specific functions, including client assessment and screening and case planning (Langdon‐Shreeve and Nickson 2021; Cherney et al. 2022; Ottis 2016); and enhance the overall effectiveness and efficiency of interventions (Uhlmann 2017).

The development of clear procedures and processes was seen to facilitate information sharing by ensuring that different partners knew when and how to share information and were confident in how this information would be used (AEF 2018; Cherney et al. 2022a; Chisholm and Coulter 2017; Foster 2010; Grossman and Barolsky 2019; Ottis 2016). Relevant examples cited as having improved information sharing included the signing of ‘memoranda of understanding and information sharing agreements’ (Ottis 2016) or naming specific points of contact within partner institutions (Chisholm and Coulter 2017; Langdon‐Shreeve and Nickson 2021). Inter‐personal relationships founded on trust were cited as crucial facilitators of information sharing (Langdon‐Shreeve and Nickson 2021).

Studies also identified barriers to information‐sharing, most commonly related to work with criminal justice agencies. These barriers included practitioners being uncomfortable or unwilling to share information *with* the police (see next section), and the difficulty of obtaining relevant information *from* the police owing to rules around the sharing of security‐sensitive information and security clearances (Chisholm and Coulter 2017; Langdon‐Shreeve and Nickson 2021). In the United Kingdom, Chisholm and Coulter (2017) noted that police sometimes had to ‘bend the rules’ to share information. Further restrictions on information sharing might also be created by privacy legislation (Grossman and Barolsky 2019).

###### 5.2.3.1.4 Police Involvement

Thirteen studies examined different ways of working with criminal justice agencies (Cherney et al. 2018; Cherney et al. 2022b; Chisholm and Coulter 2017; Davies 2023; Gielen 2020; Grossman and Barolsky 2019; Langdon‐Shreeve and Nickson 2021; Oberg et al. 2023; Ottis 2016; Rousseau et al. 2024; Thomas et al. 2017; Uhlmann 2017; Weine et al. 2018). Although participants in some studies emphasised the importance of programmes being independent from the police and the security services (Cherney et al. 2022b; Gielen 2020), the police were often seen as important partners in multi‐agency structures, most commonly in relation to making referrals and jointly assessing risk and eligibility with partner agencies (Langdon‐Shreeve and Nickson 2021; Ottis 2016; Weine et al. 2018).

All data relating to this implementation factor were again qualitative, drawn from qualitative studies (*n* = 11) or interviews conducted as part of mixed methods studies (*n* = 2). The quality of these qualitative designs was high, with three‐quarters scoring positively on 9 (*n* = 3) or all 10 (*n* = 6) of the 10 CASP domains. The evidence is therefore considered robust and highlights how police involvement with interventions created both opportunities and barriers around identifying and engaging clients (*Reach*) and created specific challenges for practitioners when working with clients (*Implementation*).

The positive contribution of police partners to multi‐agency working structures was noted by practitioners interviewed in several studies, for example, when helping to ‘jointly screen and assess risk’ (Langdon‐Shreeve and Nickson 2021, 10). However, challenges associated with police involvement were more commonly identified. A key issue was the impact of (potentially) having to report young people to the police if they exceeded a certain threshold of risk. Although some practitioners noted that there would be cases when doing so was important for meeting a ‘duty of care’ (Langdon‐Shreeve and Nickson 2021), this was seen as having ‘fundamental implications for the important relationships of trust that services needed to form with clients’ (Cherney et al. 2018, 15; Cherney et al. 2022b; Ottis 2016), as well as potentially serving to stigmatise youth (Davies 2023).

Although some practitioners spoke of having their own thresholds or ‘red flags’ (Davies 2023; Grossman and Barolsky 2019), interventions benefitted from having clearly defined criteria and processes for contacting law enforcement (Grossman and Barolsky 2019; Uhlmann 2017; Weine et al. 2018). Being transparent with clients and communities around whether, and under what circumstances, information might be shared with law enforcement can help build confidence that programmes are not ‘intelligence‐gathering’ exercises (Ottis 2016), and build trust with intervention clients (Cherney et al. 2022b; Uhlmann 2017).^25^ If practitioners fail to do so and then report an incident to the authorities at a later date, ‘this can impact on the level of trust between a client and staff member and influence the disclosure of sensitive information’ (Cherney et al. ,2022b 37).

Police involvement was noted as a potential source of ‘anxiety’ that might inhibit efforts to engage young people and their families, particularly in the context of voluntary programming (Langdon‐Shreeve and Nickson 2021). Ottis (2016) noted that Calgary Police had structured the Re‐Direct programme in a way that sought to ‘minimize’ such concerns. Whilst the programme was police‐led, and information relating to clients was held on police systems, this information was not accessible to staff outside the ReDirect team.

Two studies noted that police were ‘gradually withdrawing’ from (Thomas et al. 2017) or would be willing to take a ‘back seat’ (Ottis 2016) within local CVE structures. Both studies highlighted the importance of the police taking a more limited role. For example, Ottis (2016) notes that Calgary Police Service input to ReDirect case planning meetings ‘is limited to presenting the background information that ReDirect has collected in its initial assessment’ (p. 39). This study concluded that ‘while the police has a role to play in radicalization prevention programming, it should not be a central one’, arguing that the police are ‘usually the wrong actor to take a leading role in community‐level radicalization prevention’ (Ottis 2016, 77), owing to the risk of securitisation and criminalisation.

###### 5.2.3.1.5 Staff Supervision and Support

Seven studies examined implementation factors relating to how programmes supported and supervised their staff (AEF 2018; Brett and Kahlmeyer 2017; Cherney et al. 2022; Gielen 2020; Langdon‐Shreeve and Nickson 2021; Skiple 2020; Uhlmann 2017). Evidence relating to staff supervision and support was drawn from qualitative research designs assessed as being high‐to‐medium quality using the CASP tool: 4 scored positively on at least 9 of the 10 domains, and 2 scored positively on all 10. Although the evidence base relating to the impact of staff supervision and support was limited to only seven studies, the fact that six of these studies were assessed as being high quality means that there was still a solid body of evidence highlighting how effective supervision and support of intervention staff was supported efficient and consistent delivery and therefore related to the *Implementation* domain of the RE‐AIM framework.

####### Institutional Support

Five studies highlighted how formal and informal types of institutional support enable practitioners to perform CVE roles (AEF 2018; Brett and Kahlmeyer 2017; Langdon‐Shreeve and Nickson 2021; Skiple 2020; Uhlmann 2017). Being able to discuss cases with colleagues and managers produced a number of tangible benefits: providing alternative perspectives on cases (Langdon‐Shreeve and Nickson 2021); and assisting with decision‐making around, for example, whether to break confidentiality (AEF 2018).

More formalised processes, for example, partnering new staff with more senior colleagues when performing certain functions (Uhlmann 2017); developing protocols ‘that outline de‐escalation techniques designed to provide the adoption of extremist beliefs, behaviours, and values’ (Skiple 2020, 5); and regular group reflective practice meetings to discuss cases (Langdon‐Shreeve and Nickson 2021) help build confidence and capability. In some contexts, programmes may also benefit from conducting risk assessments and identifying mitigating strategies for reducing risks to staff and to clients (Brett and Kahlmeyer 2017).

####### Supervision and Quality Assurance

The importance of effective supervision and quality assurance was also noted in four studies (Cherney et al. 2022a; Gielen 2020; Langdon‐Shreeve and Nickson 2021; Uhlmann 2017). Many of the support processes discussed above also performed an important quality control function. For example, peer discussion and supervision (Uhlmann 2017), and reflective group sessions that provided practitioners with space to reflect on their own practice and on cases (Langdon‐Shreeve and Nickson 2021; Uhlmann 2017). Similarly, introducing ‘procedures for case review that involve independent scrutiny’ can help to quality assure practice (Cherney et al. 2022a, 55). Employing ‘process supervisors’ (Gielen 2020) or institutional leads (Langdon‐Shreeve and Nickson 2021) was seen as helping to improve the consistency and quality of delivery. For example, the BAMF Advice Centre uses ‘outside professional supervisors’ to ensure that ‘[w]orking processes are supported and kept on a professional basis’ (Uhlmann 2017, 29).

Two studies discussed the importance of governments and/or funding organisations setting quality standards (Gielen 2020; Uhlmann 2017). This was most clearly reflected in the evaluation of the BAMF Advice Centre in Germany, which assessed the performance of the Centre and its local partners against specific standards as discussed above (Uhlmann 2017). Within such standards, the importance of practitioners receiving an adequate level of relevant training was again identified as important for ensuring the consistency and quality of delivery (Langdon‐Shreeve and Nickson 2021; Uhlmann 2017).

###### 5.2.3.1.6 Resourcing programmes

Fifteen studies discussed the importance of programmes being adequately resourced and having the requisite capacity to perform effectively (AEF 2018; Badurdeen and Ndenyele 2024; Brett and Kahlmeyer 2017; Cherney et al. 2018; Cifuentes et al. 2013; Fisher et al. 2020; Foster 2010; Gielen 2020; Grossman and Barolsky 2019; Langdon‐Shreeve and Nickson 2021; Oberg et al. 2023; Osorno Hernandez 2021; Thomas et al. 2017; Uhlmann 2017; Weine et al. 2018).

The analysis of resourcing and capacity is primarily based on qualitative data extracted from qualitative (*n* = 10) and mixed methods (*n* = 5) studies assessed as being of medium‐to‐high quality based on scoring positively on 7 (*n* = 4), 8 (*n* = 4), 9 (*n* = 3), or all 10 (*n* = 4) CASP domains. The evidence base pointing to the crucial role of resourcing in facilitating implementation was therefore considered robust based on the number and quality of studies examining this aspect of intervention delivery. This evidence highlighted how the funding and resourcing of programmes affects multiple domains of the RE‐AIM framework, facilitating programme design and delivery in the short term (*Implementation*), as well as impacting the sustainability of programmes over the longer term (*Maintenance*).

####### Time

Six studies discussed how time constraints can limit the amount of time that practitioners have to engage with young people (AEF 2018; Cherney et al. 2018; Cifuentes et al. 2013; Gielen 2020; Oberg et al. 2023; Uhlmann 2017). These constraints were sometimes a feature of interventions working with youth for set periods of time, for example, when delivering a set curriculum. Cifuentes et al. (2013) argued that the length of delivery of the Think Project pilot was ‘constraining’ in this regard, as the course was designed around eight weekly half‐day sessions. However, time constraints were often identified as being particularly pronounced for more tailored – and therefore resource‐intensive – programmes that did not set limits on treatment length, such as case management, mentoring, and counselling programmes.

The effective delivery of such programmes was seen to rest on practitioners having sufficient capacity to devote significant time and effort to clients (AEF 2018; Uhlmann 2017). However, resource and time constraints may require practitioners to compromise ‘between ideal practice and pragmatics’ when ‘the time, knowledge and resources needed to conduct complex work engaging with clients in sufficiently nuanced detail’ is not available (Oberg et al. 2023, 665). The intensity and complexity of this work, coupled with staff shortages, and increasing caseloads, can also reduce the time and resources available to work with individual clients, or respond to requests (Uhlmann 2017).

Such intensity can contribute to ‘burnout’ (Cherney et al. 2018) or be ‘psychologically exhausting’ (Uhlmann 2017). It can also create organisational challenges for institutions and funders, who must ensure that the application of resources in an individual case is ‘proportional’ to the expected benefit (AEF 2018). An additional challenge is the open‐ended nature of some tailored approaches, whereby support continues until no longer needed. In these circumstances, programmes risk becoming ‘overwhelmed’ if clients ‘become dependent on them by staying in overly long programs’ (Gielen 2020, 138).

Short‐term funding horizons were seen to produce different time constraints. Work with youth may need to end prematurely if funding runs out or if a project ends, in ways that might inhibit ‘sustainable, long‐term stabilisation processes’ (Uhlmann 2017, 48). Short set‐up times can mean that relevant structures and partnerships are not established before programmes commence (Cifuentes et al. 2013). Broader challenges relating to short‐term or time‐limited funding are discussed in more detail in the next section.

####### Funding and Resources

Thirteen studies discussed funding and resources (AEF 2018; Badurdeen and Ndenyele 2024; Brett and Kahlmeyer 2017; Cherney et al. 2018; Fisher et al. 2020; Foster 2010; Gielen 2020; Grossman and Barolsky 2019; Langdon‐Shreeve and Nickson 2021; Osorno Hernandez 2021; Thomas et al. 2017; Uhlmann 2017; Weine et al. 2018). These studies pointed to challenges related to ‘limited financial and human resources’ (Brett and Kahlmeyer 2017), and the importance of funding from government (Cherney et al. 2018; Gielen 2020; Thomas et al. 2017; Uhlmann 2017; Weine et al. 2018), as well as more hybrid or independent funding models (AEF 2018; Osorno Hernandez 2021).

The importance and limitations of government funding were emphasised in several studies (Cherney et al. 2018; Gielen 2020). In Australia, for example, the government's growing reliance on multi‐agency partners, or on partnerships with community and non‐governmental organisations as noted above, means that the provision of such funding is necessary to avoid partners being ‘set up for failure’ (Cherney et al. 2018). However, the short‐term and unstable nature of government funding arrangements was commonly identified as a challenge to programme capacity and sustainability in different countries, and as potentially inhibiting a culture of best practice from emerging (AEF 2018; Cherney et al. 2018; Foster 2010; Osorno Hernandez 2021; Uhlmann 2017). As noted above, time‐limited funding arrangements can also limit a programme's ability to deliver sustainable change, for example, when work with a young person has to end prematurely (Uhlmann 2017). One study examining work in Kenya also suggested that short‐term approaches that do not tackle more systemic or structural issues may also simply be a ‘band aid’ for wider problems facing at‐risk youth (Badurdeen and Ndenyele 2024).

Providing more long‐term funding was therefore seen as crucial for ensuring the sustainability of programmes (AEF 2018; Brett and Kahlmeyer 2017; Uhlmann 2017). However, because funding is often time‐limited, it is also important to plan exit strategies to limit the impact of projects ending (Brett and Kahlmeyer 2017). These can include formalising structures and partnerships, for example, between local providers and employers, so that they outlast the project (Fisher et al. 2020).

In countries such as the United Kingdom, the availability of national government funding can also vary across regions (Langdon‐Shreeve and Nickson 2021; Osorno Hernandez 2021). Local governments may therefore need to fund specific activities or positions that are financed by the national government in other regions (Langdon‐Shreeve and Nickson 2021). However, local authorities face their own financial pressures that may limit the sustainability of the funding they provide (Langdon‐Shreeve and Nickson 2021; Thomas et al. 2017). An over‐reliance on national (and local) government funding can therefore bring risks. This makes it important for programmes to work towards being sustainable, so they are less reliant on government funding (AEF 2018) by finding alternative funding sources (Osorno Hernandez 2021). This is particularly important for those interventions that might prefer to remain independent from government funding owing to the perceived reputational risks of accepting support from state actors (Osorno Hernandez 2021). More hybrid models, where programmes receive funding from multiple sources, were identified as one potential way of ensuring sustainability (AEF 2018; Osorno Hernandez 2021).

####### Staffing

Opportunities and challenges relating to staffing and recruitment were discussed in six studies (AEF 2018; Cherney et al. 2018; Gielen 2020; Langdon‐Shreeve and Nickson 2021; Osorno Hernandez 2021; Uhlmann 2017). Recruiting (and retaining) staff was a particular challenge (AEF 2018; Cherney et al. 2018; Langdon‐Shreeve and Nickson 2021; Uhlmann 2017), and it was noted in one study that ‘[t]he supply of qualified personnel who are well trained, able and willing to work in this very complex field is small’ (Uhlmann 2017, 46). High workloads, low pay, and a lack of certainty or permanency owing to short‐term funding or employment terms were identified as barriers to recruitment and retention (AEF 2018; Cherney et al. 2018; Uhlmann 2017). A lack of staff retention was also seen as inhibiting the development of best practice or resulting in important knowledge and experience being lost when individuals leave their post (Cherney et al. 2018; Langdon‐Shreeve and Nickson 2021; Osorno Hernandez 2021).

Studies from Europe emphasised the importance of professionalisation and professional standards when recruiting and training staff, and delivering services (Gielen 2020; Uhlmann 2017). Making funding available to support recruitment or remunerate skilled professionals tasked with delivering a CVE function outside of their usual role (Cherney et al. 2018; Gielen 2020; Langdon‐Shreeve and Nickson 2021; Osorno Hernandez 2021), was also seen as important in Europe and Australia, although not always possible.

##### 5.2.3.2 Identifying and Engaging Youth and Families

This section examines implementation factors that impact how programmes identified and adopted youth and families as clients, as outlined in Table 16 and summarised in Table 19 below. The analysis below examines interventions that worked directly with at‐risk and radicalised youth, as well as those that aimed to prevent and counter radicalisation indirectly through services delivered to families or others in the ‘social environment’ (Uhlmann 2017) of young people. This analysis is combined because there was a large overlap in the factors that impacted both types of intervention. Insights that are specifically relevant to working with youth and with families are identified where relevant.

**Table 19:** Implementation factors relating to identifying and engaging clients.

Factor	Facilitators	Barriers
*Identifying and engaging eligible clients*		
RE‐AIM: Reach		
Working with partners	1. Partnerships with institutions or actors able to identify and refer at‐risk or radicalised youth 2. Building relationships with organisations that regularly engage with young people 3. Working with organisations with credibility and visibility in local communities	1. Reluctance from community partners to refer, for example, if they believed they were better able to handle cases and reduce stigma
Accessibility of programmes	1. Publicising services means programmes are more visible to local partners and those seeking support 2. Identifying and communicating dedicated points of contact 3. Well‐defined, clearly understood and ideally standardised referral mechanisms and processes	1. Differing thresholds for risk and referrals across regions 2. Lack of confidence or experience in referring to programmes
Clearly defined target audience	1. Shared understanding of the target audience and eligibility criteria or thresholds for referring a case of concern 2. Input from expert practitioners or teams able to offer advice on cases 3. Community safeguarding panel independent of government referral processes able to act as a gatekeeper linking communities to formal government programmes	1. Lack of understanding and/or subjectivity in referral processes 2. Weak understanding or lack of clear guidance as to the client group
Accurate and consistent decisions	1. Partnership working with internal or external actors can support decision‐making over case adoption 2. Codified eligibility criteria and threshold guidance 3. Specialist risk assessment tools	1. Inconsistent adoption thresholds across institutions and individuals 2. Checklist approach to using risk assessment tools that does not allow for a sufficiently nuanced assessment of a case
Intake process and procedures	1. Effective assessment of risks and needs 2. Reducing waiting times between referral/adoption and accessing services 3. Minimising bureaucratic or administrative barriers 4. Directing cases to relevant staff/partners at intake 5. Effective information sharing between partners involved in referring, screening and handling cases at intake	
Accessibility of services	1. Reducing the threshold for clients to access services by not charging, or making services available in different languages, and potentially making services available online	
Enroling clients	1. Voluntary enrolment in programmes to reduce perceptions of securitisation	1. Difficulties gaining consent from clients in voluntary programmes 2. Parents or clients feel they have little choice but to consent and/or take part in programmes 3. Overt pressure, for example, from police to take part in programmes can be counterproductive

###### 5.2.3.2.1 Identifying Eligible Clients

Sixteen studies examined how programmes defined and/or identified eligible clients, and associated implementation factors (AEF 2018; Brett and Kahlmeyer 2017; Cherney et al. 2022a; Chisholm and Coulter 2017; Davies 2023; Fisher et al. 2020; Foster 2010; Gielen 2020; Grossman and Barolsky 2019; i‐Works Research Ltd. 2013; Langdon‐Shreeve and Nickson 2021; Osorno Hernandez 2021; Ottis 2016; Skiple 2020; Uhlmann 2017; Weine et al. 2018).

Evidence was again primarily qualitative, drawn from qualitative (*n* = 11) and mixed methods (*n* = 5) studies assessed as being high‐to‐medium quality based on over half (*n* = 9) scoring positively on nine or more domains of the CASP framework. The number and quality of these studies therefore provided a robust body of evidence pointing to the impact of different approaches on the *Reach* domain of the RE‐AIM framework. Effective and efficient identification of clients was, in turn, identified as a crucial facilitator of implementation, as to reach target clients, interventions first had to identify them.

####### 5.2.3.2.1.1 Working With Partners

Most programmes included in the review relied on referrals from community actors, front‐line professionals (e.g., teachers and social workers), and/or multi‐agency partners to identify at‐risk or radicalised children and adolescents. This was particularly true of case management programmes in this review, but as already noted, programmes such as the Tolerance Project also rely in part on referrals (Skiple 2020).

Twelve studies identified partnerships with institutions or actors who are well‐placed to identify and refer at‐risk or radicalised youth as crucial (AEF 2018; Brett and Kahlmeyer 2017; Chisholm and Coulter 2017; Davies 2023; Fisher et al. 2020; Foster 2010; Gielen 2020; i‐Works Research Ltd. 2013; Langdon‐Shreeve and Nickson 2021; Osorno Hernandez 2021; Ottis 2016; Weine et al. 2018). Building partnerships with institutions that had day‐to‐day contact with younger cohorts was therefore seen as important. This included schools (Davies 2023; i‐Works Research Ltd. 2013) and children's social care providers (Chisholm and Coulter 2017; Langdon‐Shreeve and Nickson 2021). It was also important that partners were embedded in and had credibility with local communities (Fisher et al. 2020) and/or had visibility of local cases that might warrant referral into national programmes (AEF 2018).

Such partnerships were seen to help improve practitioners' access to at‐risk and radicalised youth. However, accessibility was sometimes a challenge. For example, Davies (2023) reported that ‘gaining access to the desired groups was not straightforward’ for one youth programme in Wales due to schools' reluctance to engage with it (p. 176). This study also noted that ‘Prevent authorities were denied access (often unknowingly)’ from cases that ‘clearly fall within the scope of Prevent as it is described in strategy documents’ because some community intervention providers felt they were better placed to handle these cases in a non‐stigmatising way (Davies 2023, 192–193).

####### 5.2.3.2.1.2 Accessibility of Programmes

The importance of programmes being accessible to local actors was noted in nine studies (AEF 2018; Cherney et al. 2022a; Chisholm and Coulter 2017; Grossman and Barolsky 2019; Langdon‐Shreeve and Nickson 2021; Osorno Hernandez 2021; Ottis, 2016; Uhlmann 2017; Weine et al. 2018). Two sub‐themes related to accessibility. The first was that *programmes need to be visible to local partners* (AEF 2018) and to individuals seeking support (Grossman and Barolsky 2019). Uhlmann (2017) identified the ‘publication of services’ as a basic standard for counselling work, highlighting the importance of public relations and media work. Identifying dedicated points of contact was also found to help local actors connect with programmes (Chisholm and Coulter 2017; Langdon‐Shreeve and Nickson 2021).

The second was the importance of *referral mechanisms and processes being well‐defined and clearly understood* (Cherney et al. 2022a; Chisholm and Coulter 2017; Langdon‐Shreeve and Nickson 2021; Osorno Hernandez 2021; Uhlmann 2017; Weine et al. 2018).^26^ A key challenge identified in studies on Prevent in the United Kingdom (Chisholm and Coulter 2017; Davies 2023; Langdon‐Shreeve and Nickson 2021) is that different individuals and institutions had different thresholds for risk and for making referrals into the government's Channel programme. Different actors – particularly those with limited experience of radicalisation cases – were less confident in deciding when a referral is needed. Chisholm and Coulter (2017) therefore point to the importance of providing ‘clear guidance’ around thresholds for referrals and for case adoption, noting this improved the confidence of social workers.

Some level of standardisation in referral processes was recommended in several studies (Chisholm and Coulter 2017; Langdon‐Shreeve and Nickson 2021; Uhlmann 2017). For example, Chisholm and Coulter (2017) concluded that local authorities should ‘define a single referral process’ to give staff ‘greater clarity and confidence about their role’ (p. 34) when deciding how to handle radicalisation cases. A follow‐up study conducted by Langdon‐Shreeve and Nickson (2021) drew a different conclusion, identifying two distinct referral pathways for radicalisation cases across different local authorities, noting that stakeholders ‘did not identify a need for a single referral pathway; [and that] there was generally a consensus that both these pathways can work well in a single local authority area’ (Langdon‐Shreeve and Nickson 2021, 33). However, this study still highlighted the importance of practitioners clearly understanding when and how to make referrals.

####### 5.2.3.2.1.3 Clearly Defined Target Audience

Ten studies examined implementation factors relating to how interventions define their target audience (Cherney et al. 2022a; Chisholm and Coulter 2017; Davies 2023; Foster 2010; Fisher et al. 2020; Langdon‐Shreeve and Nickson 2021; Osorno Hernandez 2021; Ottis 2016; Skiple 2020; Uhlmann 2017). These studies highlighted the importance of different partners having a shared understanding of the target audience and any associated eligibility criteria so as to make relevant referrals.

A key theme across these studies was the importance of programmes having *clearly defined eligibility criteria and/or thresholds* for referring cases of concern and communicating this to relevant audiences. Although the importance of programmes having a clearly defined target age group was noted (Cherney et al. 2022a), this discussion primarily focused on the importance of identifying appropriate criteria for defining ‘at‐risk’ youth and on setting appropriate thresholds of risk. For example, Fisher et al. (2020) cited the use of 10 eligibility criteria as a key strength of the STRIVE II mentoring programme that had helped to ensure the programme identified and supported at‐risk youth.

The problems of ‘under and over identification’ discussed by Skiple (2020) above were also seen when target audiences and associated eligibility criteria were not well‐defined or understood. For example, Fisher et al. (2020) reported that only 53% of potential mentees referred into the STRIVE II mentoring programme in Kenya were accepted. Although positive about the programme's ability to reach its target audience, they still concluded ‘there is further work that could be done in improving stakeholder knowledge of the selection criteria’, noting the subjective nature of some of the criteria used (Fisher et al. 2020, 27). Similar challenges were also noted by Chisholm and Coulter (2017) and Langdon‐Shreeve and Nickson (2021), who pointed to the large number of referrals received by local Prevent teams in the United Kingdom that fell below the threshold for Channel adoption, as well as variations across individuals and institutions as to their threshold for referral.

Under‐identification was less widely discussed, although Foster (2010) noted how practitioners in Manchester in the North‐West of England ‘were unsure of who their client group was or should be, what the need was and how it could be met’ (p. 20) when trying to implement local Prevent programming for ‘vulnerable’ youth owing to a lack of clear guidance.

One UK practitioner interviewed by Davies (2023) argued that a willingness to hold risk above standard reporting thresholds was a positive aspect of their practice. They felt that it was sometimes better not to refer onwards into government programmes, as their organisation was better placed to support youth, and to avoid potential criminalisation, arguing that ‘our model was safer for young people than Prevent’ (Davies 2023, 196). Although they had ‘very clear criteria and “red flags” which would qualify their cases for referrals onto Prevent’ they also noted they were not ‘necessarily engaging along those lines’ used by Prevent officials when assessing risk or, whether a referral is needed (p. 194).

Programmes and practitioners used several tools and methods to improve the quality of referrals, and in turn, how referrals were handled. This included using specific radicalisation referral forms that were perceived to help ‘focus the referrer's mind on the reason and evidence for the referral, which can support the person screening the referral to pick up on the risks’ (Langdon‐Shreeve and Nickson 2021, 34; also, Chisholm and Coulter 2017; Ottis 2016). Clear guidance about thresholds for reporting was important (Chisholm and Coulter 2017) as was input from expert practitioners or teams offering advice on specific cases (Chisholm and Coulter 2017; Davies 2023). A distinctive approach discussed by Davies (2023) was the development of a ‘community safeguarding panel’ that operated in parallel to the local authority Channel panel in a specific region of England.^27^ This community‐led panel was seen as ‘creating the infrastructure for P/CVE referrals’ (Davies 2023, 119) and served as a gatekeeper linking communities to formal government interventions. Community members would refer cases to the community‐led panel, which would, in turn, collaborate with Channel when this was deemed necessary.

###### 5.2.3.2.2 Engaging Eligible Clients

Thirteen studies examined implementation factors relating to how programmes adopted and enroled at‐risk and radicalised clients once they are identified (AEF 2018; Badurdeen and Ndenyele 2024; Cherney et al. 2022a; Chisholm and Coulter 2017; Davies 2023; Fisher et al. 2020; Gielen 2020; Langdon‐Shreeve and Nickson 2021; Ottis 2016; Rousseau et al. 2024; Uhlmann 2017; UNDP 2022; Weine et al. 2018).

Relevant evidence was again primarily qualitative, drawn from qualitative studies (*n* = 8) or qualitative components of mixed‐methods (*n* = 4) studies. These studies were assessed as being high quality based on half scoring positively on nine (*n* = 3) or all 10 (*n* = 4) of the CASP domains. Evidence relating to the process of enroling youth once they have been identified was also extracted from one quantitative study using a quasi‐experimental design that, despite having a serious risk of bias, provided relevant insights. Taken together, these studies provided a solid body of evidence highlighting how the effective and efficient adoption of clients was again seen as impacting the *Reach* domain of the RE‐AIM framework, as it impacted whether eligible clients were ultimately supported.

####### 5.2.3.2.2.1 Accurate and Consistent Decision‐Making

Nine studies pointed to the importance of effective and efficient case adoption decisions (AEF 2018; Cherney et al. 2022a; Chisholm and Coulter 2017; Davies 2023; Fisher et al. 2020; Langdon‐Shreeve and Nickson 2021; Ottis 2016; Uhlmann 2017; Weine et al. 2018). This reflected the large number of programmes that relied on referrals from different actors (as noted above) that required some form of screening before adoption.

Partnership working within institutions was a crucial facilitator of decision‐making. In the Netherlands, for example, Forsa staff engaged with an in‐house mental health team to support the ‘interpretation of cases during the intake process’ (AEF 2018, 50). External partnerships were also important. Examples included several interventions assessing cases in multidisciplinary teams at intake (e.g., Weine et al. 2018); children's social care workers in England working in partnership with Counter‐Terrorism Police to ‘jointly screen and assess risk’ (Langdon‐Shreeve and Nickson 2021); and practitioners in the Netherlands seeking guidance from the National Support Centre for Extremism (LSE), which AEF (2018) found helped to facilitate accurate and timely case adoption decisions.

However, several studies pointed to inconsistencies in adoption thresholds across different institutions and different individuals (Chisholm and Coulter 2017; Davies 2023; Langdon‐Shreeve and Nickson 2021). Such differences were seen to complicate multi‐agency collaboration. Codifying eligibility criteria (Fisher et al. 2020) and threshold guidance (Chisholm and Coulter 2017; Langdon‐Shreeve and Nickson 2021) were again seen as helping to overcome this challenge. For example, Fisher et al. (2020) noted in their evaluation of the STRIVE II mentoring pilot in Kenya that the use of codified eligibility criteria ‘takes some of the subjective decision‐making out of recruitment process’ (p. 24). However, as noted above, subjective criteria may remain ‘open to interpretation’ (Fisher et al. 2020, 24). Studies also highlighted the utility of using risk assessment tools to aid in this process (Cherney et al. 2022a; Langdon‐Shreeve and Nickson 2021 Ottis 2016), although practitioners have cautioned against using a ‘checklist’ approach when using such tools (Ottis 2016).

####### 5.2.3.2.2.2 Intake Process and Procedures

The importance of an efficient intake process was noted in eight studies (AEF 2018; Cherney et al. 2022a; Chisholm and Coulter 2017; Gielen 2020; Langdon‐Shreeve and Nickson 2021; Ottis 2016; Uhlmann 2017; Weine et al. 2018). The effective assessment of risk (and needs) was often cited as a crucial part of this process (Cherney et al. 2022a; Weine et al. 2018). Other features of a well‐functioning intake process included limited waiting times between referral/adoption and accessing services (AEF 2018; Uhlmann 2017; Langdon‐Shreeve and Nickson 2021); minimising bureaucratic or administrative barriers (Gielen 2020); directing cases to relevant staff or partners at intake (Chisholm and Coulter 2017; Langdon‐Shreeve and Nickson 2021; Uhlmann 2017); and effective information sharing between partners involved in referring, screening, and handling a case at intake (Langdon‐Shreeve and Nickson 2021; Uhlmann 2017). For example, Uhlmann (2017) notes that initial analysis and ‘case preparation’ by call handlers and counsellors working at the BAMF Advice Centre in Germany helped inform the assessments and work of local providers to whom cases were referred.

####### 5.2.3.2.2.3 Accessibility of Services

A related factor examined by four studies was the importance of services being accessible to those seeking support (AEF 2018; Gielen 2020; Langdon‐Shreeve and Nickson 2021; Uhlmann 2017). Two of these studies emphasised the importance of clients facing a ‘low threshold’ for accessing services (Gielen 2020; Uhlmann 2017). Strategies for reducing this threshold included delivering services in accessible locations (e.g., close to clients, or in their homes); not charging for services; and making services available in different languages. Gielen (2020) suggested that online delivery might also reduce barriers to entry, although stakeholders interviewed by Langdon‐Shreeve and Nickson (2021) identified challenges linked to services being moved online in the wake of the COVID‐19 pandemic, reporting ‘people are less likely to engage when it is not face‐to‐face’ (p. 66).

####### 5.2.3.2.2.4 Enroling Clients

Client enrolment was examined in 10 studies (AEF 2018; Badurdeen and Ndenyele 2024; Cherney et al. 2022a; Davies 2023; Langdon‐Shreeve and Nickson 2021; Oberg et al. 2023; Ottis 2016; Rousseau et al. 2024; Uhlmann 2017; UNDP 2022). Several of these studies stressed the importance of programmes being voluntary. For example, Ottis (2016) notes that local stakeholders in Calgary made ReDirect a voluntary programme to counter fears of securitisation, whilst Uhlmann (2017) identified ‘no compulsion to receive counselling’ as an important macro‐standard for counter‐radicalisation work in Germany. However, challenges in obtaining consent – including consent later being withdrawn, or clients going ‘AWOL’ (Davies 2023) – were also noted (AEF 2018; Davies 2023; Langdon‐Shreeve and Nickson 2021; UNDP 2022). And, in Canada, Rousseau et al. (2024) note that some parents only consented to children being assessed ‘because they had no choice’ (p. 693), for example, when compelled by youth protective services.^28^

Langdon‐Shreeve and Nickson (2021) identify a number of approaches that practitioners in the United Kingdom used to overcome challenges associated with obtaining consent. For example, children's social care staff reported that conducting joint visits to families with the police or local Channel coordinator had ‘led to improved uptake of voluntary Channel support’, potentially because it helped to ‘mitigate some of the anxiety families may have about engaging with police’ (p. 67). Such an approach was seen as particularly useful when engaging families who already have an allocated social worked ‘as families are more likely to engage with professionals with whom they already have a relationship’ (p. 67).

The UNDP (2022) evaluation of the *Youth for Social Harmony in the Fergana Valley* programme in Uzbekistan was particularly noteworthy as it used a quasi‐experimental design to examine whether employing dedicated youth ambassadors to encourage youth to enrol and persist with the programme had impacted levels of engagement. Contrary to expectations, it concluded that this ‘did not bring about positive changes in attendance’ (p. 21), identifying the short 2‐week duration of the intervention and the potential lack of motivation of some ambassadors as potential explanations for this result. Although this study was assessed as having a serious risk of bias, the results are still illustrative.

Individuals might face, or perceive, pressure to cooperate, even when programmes are voluntary (Badurdeen and Ndenyele 2024; Oberg et al. 2023; Rousseau et al. 2024). This type of pressure can produce negative effects. For example, Oberg et al. (2023) discuss a case in which the home of an individual who had refused to participate in an intervention was raided by the police for suspected drug offences 'not only to gain evidence about a criminal act, but also to get some leverage on the individual in order to convince them to cooperate' (p. 1756). The authors saw this as contributing to an increased sense of ‘othering’ whilst a practitioner interviewed for this study argued that it was likely traumatising those present at the raid. Other studies focus on more implicit pressure or perceptions of pressure. For example, Badurdeen and Ndenyele (2024) spoke of pressure being placed on young people and their families to engage with P/CVE programmes ‘to avoid being seen as at risk or a threat and monitored’ (p. 16).^29^

##### 5.2.3.3 Working With Youth and Families

This section examines implementation factors that facilitated practitioners and interventions in their work with youth and families, as outlined in Table 17 above. As in the previous section, the analysis below examines both direct and indirect forms of prevention owing to the high degree of overlap in relevant factors. Facilitators and barriers associated with relevant implementation factors are summarised in Table 20 below.

**Table 20:** Implementation factors relating to working with clients.

Factor	Facilitators	Barriers
*Relational processes*		
RE‐AIM: Reach; Implementation; Effectiveness		
Establishing relationships and trust	1. Emphasis on building trust from the earliest stages of the intervention 2. Diverse teams with a range of characteristics and skills support effective matching able to engender trusting relationships 3. Skilled case workers who have experience and confidence in building trust with clients 4. Efforts to build trust with parents 5. Ongoing commitment to sustaining trusting relationships throughout the intervention 6. Case workers reflecting respect, transparency, empathy and reliability to build trust 7. Equitable and horizontal relationships between case workers and clients	
Matching practitioners and clients	1. Individualised approach to matching clients with case workers 2. Assessing whether matching according to gender, ethnicity, or religious background is appropriate	
Practitioner commitment	1. Staff reflecting commitment to the client and being responsive to their needs 2. Staff being available to clients in case of need	1. High caseloads and staff shortages can limit practitioners' capacity to respond to or engage with clients 2. High levels of commitment and care can lead to stress 3. Perceived lack of commitment from other agencies
*Client‐centred approach*		
RE‐AIM: Effectiveness		
Individualised approach	1. Tailored support able to address the individualised nature of radicalisation processes 2. Case management approaches that enable a tailored approach 3. Person‐centred, flexible, responsive and needs‐based approach 4. Provision of practical support, for example, with housing or managing day‐to‐day living 5. Combining formal interventions with informal types of support	1. Lack of clear guidance as to how to handle radicalisation cases caused by need for flexibility
Developmentally appropriate	1. Interventions should be age and/or developmentally appropriate 2. Recognition that ruptures and setbacks are part of adolescence 3. Trauma‐informed approaches that recognise the developmental effects of trauma on how young people present themselves and respond to interventions	
Cultural sensitivity	1. Training to help develop appropriate levels of cultural sensitivity 2. Practitioners with appropriate language and cultural skills	
*Ways of working*		
RE‐AIM: Effectiveness		
Dialogue and discussion	1. Neutral, non‐biased and non‐judgmental approach, particularly when engaging with questions of ideology 2. Opportunities for reflective practice to draw out biases or preconceptions 3. Training on the role of trauma in youth radicalisation 4. Group work that brings peers with different perspectives together supports dialogue and debate 5. Safe spaces and open forums which afford young people the opportunity to share their views and have them challenged	
Empowerment and agency	1. Enhancing opportunities for young people to experience a sense of agency and control over their intervention 2. Focusing on developing self‐esteem and self‐confidence 3. Identifying and activating resources, protections and strengths in the young people's environment 4. Finding opportunities to pursue positive alternatives to violent extremism	
*Contextualised approach*		
RE‐AIM: Reach; Effectiveness		
Working with families	1. Collaborating with families can help facilitate the delivery of services and enhance the support available to young people 2. Working with families to enhance practitioners' understanding of the client 3. Enabling families to play a role in motivating the client 4. Direct engagement with families can help the client reconnect with their family, mediate family tensions, and mitigate issues that may impact their engagement with a programme 5. Whole‐of‐family approaches provide support for families and help to meet their needs and enhance their capacity to support their family member	1. Distrust or unwillingness to engage with programmes or consent to their child's participation
Socio‐ecological approach	1. Awareness of contextual factors (e.g., negative peer groups) that influence an individual's vulnerability to radicalisation 2. Recognising the impact of social stigma and structural barriers, such as discrimination that young people might face 3. Identifying and maximising the strengths and protections in the client's social environment 4. Providing opportunities to socialise young people into pro‐social networks and contexts 5. Capacity building within communities so they are better able to support young people at risk of radicalisation	1. Communities or contexts that lack the resources to support young people

###### 5.2.3.3.1 Relational Processes

Twenty studies highlighted the importance of relational processes between clients and practitioners (AEF 2018; Brett and Kahlmeyer 2017; Cherney et al. 2022; Cherney et al. 2018; Cherney 2024; Chisholm and Coulter 2017; Cifuentes et al. 2013; Davies 2023; Fisher et al. 2020; Gielen 2020; Grossman and Barolsky 2019; i‐Works Research Ltd. 2013; Joyce and Lynch 2017; Langdon‐Shreeve and Nickson 2021; Oberg et al. 2023; Osorno Hernandez 2021; Rousseau et al. 2024; Sahgal and Kimaiyo 2020; Skiple 2020; Uhlmann 2017).

This evidence was drawn from high‐quality qualitative research: over half of the studies (*n* = 13) scored positively on 9 or all 10 domains of the CASP framework. The evidence base pointing to the importance of relational processes in facilitating implementation is particularly robust given the large number of high‐quality studies that provided evidence to this effect. This evidence highlighted how strong and trusted relationships between clients and practitioners and the commitment of practitioners to clients positively impacted multiple domains of the RE‐AIM framework: helping to motivate clients to engage (and remain engaged) with programmes (*Reach*); to identify and address any emerging issues (*Implementation*); and to motivate positive change amongst clients (*Effectiveness*).

####### Establishing Relationships and Trust

A trusted relationship between practitioners and youth and/or families was identified as crucial in motivating engagement and change in sixteen studies (AEF 2018; Brett and Kahlmeyer 2017; Cherney et al. 2022a ; Cherney et al. 2018; Cherney 2024; Davies 2023; Fisher et al. 2020; Grossman and Barolsky 2019; i‐Works Research Ltd. 2013; Langdon‐Shreeve and Nickson 2021; Oberg et al. 2023; Osorno Hernandez 2021; Rousseau et al. ;^2024^; Sahgal and Kimaiyo 2020; Skiple 2020; Uhlmann 2017).

A number of studies noted that establishing trust can be difficult and/or emphasised that building trust is a time‐consuming process (AEF 2018; Brett and Kahlmeyer 2017; Cherney 2024; Rousseau et al. 2024). Authors therefore suggested that the establishment of trust between practitioners and clients might itself be an important outcome (Cherney et al. 2022a) or ‘milestone’ (Cherney 2024), particularly in cases when clients are initially reluctant to engage with programmes. An emphasis on building trust early in relationships was seen to help practitioners ‘get a foot in the door’ (Cherney et al. 2018, 15). Uhlmann (2017) argued that ‘establishing a foundation of trust between the counsellor and the advice seeker […] begins with the very first contact’ (p. 40) and should therefore be ‘Step 1’ in the counselling process. Employing a diverse team with different characteristics and skills was also cited as increasing the chances of finding the right match between a practitioner and a young person or family (Chisholm and Coulter 2017; Fisher et al. 2020).

Although challenging, building trust with parents was also seen as important in obtaining consent when first seeking to engage young people (Rousseau et al. 2024).^30^ This was also a way of ensuring that any issues in the family home that might affect a young person's engagement in a programme were known to practitioners (Cherney et al. 2022a).

Establishing trust in the short to medium term was important in itself; however, a sustained trusted relationship was also considered the ‘foundation’ for longer‐term change (AEF 2018; Uhlmann 2017). Uhlmann (2017) therefore argued that trust must be ‘expanded further and cultivated’ once it has been established, and identified principles such as respect, empathy, authenticity, forthrightness, transparency, courtesy, and reliability as counselling standards that ‘are fundamental in connection with gaining and maintaining trust (and thus relevant to the entire counselling process)’ (p. 40). Eliciting ‘the individual's intrinsic motivation to cooperate’ (AEF 2018, 45), whilst also sustaining motivation in the event of any setbacks (Cherney et al. 2022a) was therefore seen as important.

Several studies similarly argued that sustaining trust and motivation over the long‐term helped facilitate positive change. For example, municipalities interviewed by AEF (2018) were positive that the lasting relationships that practitioners built with clients ‘enables clients to truly change’ (p. 38). Youth who felt they had benefited from programmes also cite a relationship or ‘click’ (AEF 2018) with a mentor or counsellor, or the approach taken by such individuals, as crucial to their positive experience (AEF 2018; Fisher et al. 2020; i‐Works Research Ltd. 2013; Sahgal and Kimaiyo 2020). For example, STRIVE II mentors in Kenya were seen as ‘people who genuinely and selflessly care’ by their mentees (Fisher et al. 2020, 25).

The skills, personality, and disposition of practitioners were therefore identified as crucial to effective implementation. Several studies highlight that practitioners must be ‘skilled in relationship building’ (Cherney et al. 2022a, 59); be able to ‘convince and entice’ (AEF 2018, 35); and have ‘the right personality and skills set’ (Brett and Kahlmeyer 2017, 19). The importance of practitioners being seen as a ‘role model’ to and by young people was also noted in several studies (Brett and Kahlmeyer 2017; Grossman and Barolsky 2019; Skiple 2020), with mentees interviewed by Fisher et al. (2020) in Kenya describing mentors as ‘inspirational’. The importance of relationships with young people being equitable and horizontal was also emphasised by Davies (2023) and Osorno Hernández (2021) in analyses of intervention work with young people in different parts of the United Kingdom.

####### Matching Practitioners to Clients

Ten studies discussed whether and how programmes might deliberately match practitioners to clients based on specific characteristics (AEF 2018; Brett and Kahlmeyer 2017; Cherney et al. 2018; Cherney et al. 2022b; Chisholm and Coulter 2017; Cifuentes et al. 2013; Fisher et al. 2020; Joyce and Lynch 2017; Oberg et al. 2023; Sahgal and Kimaiyo 2020). Although in general, study authors suggested that such an approach improved the fit between practitioners and clients, there was some debate on this point.

Partnering with youth and practitioners based on a shared background or experience was a common practice. Programmes might match youth with practitioners based on, for example, a shared ‘ethnic and religious background’ (Cherney et al. 2018, 12), gender (Sahgal and Kimaiyo 2020), or a shared history of engagement in a specific extremist milieu (Chisholm and Coulter 2017; Joyce and Lynch 2017), or recruit providers who come from the same communities as their clients (Brett and Kahlmeyer 2017; Fisher et al. 2020). This approach was informed by a belief that practitioners who hold relevant knowledge and experience are able to build motivating relationships with their clients. For example, the evaluation of STRIVE II in Kenya noted that mentors recruited from the same communities as mentees were ‘relatable’ and individuals that young people could ‘realistically aspire to be like’ (Fisher et al. 2020, 26–28). Practitioners interviewed by Cherney et al. (2022b) also highlighted that programmes need to be aware of the unfortunate situation when ‘ideological factors may influence the level to which a radicalised youth engages and reacts to staff (e.g., staff who are female or of certain racial and ethnic backgrounds)’ (p. 55) when considering which practitioners are best placed to work with a particular young person or family.

However, matching practitioners and clients based on a shared background ‘may not necessarily guarantee success in relation to client engagement and the effectiveness of service provision’ (Cherney et al. 2018, 13). In some cases, it may even be more beneficial to take a different approach. For example, both Cifuentes et al. (2013) in Wales and Adams et al. (2023) in Australia highlight how youth at risk of being drawn into right‐wing extremism likely benefited from having staff from Muslim backgrounds lead or attend sessions with them. For example, Think Project workshops were jointly delivered by a ‘youth worker from a Muslim background’ and a ‘White Welsh’ youth worker, which ‘promotes a message of diversity in action’ whilst also providing an opportunity for youth to ‘form a bond’ and engage in dialogue with somebody from a Muslim background, which ‘fundamentally and experientially challenges and goes against the negative stereotypes and myths’ perpetuated by far‐right organisations (Cifuentes et al. 2013, 321).

####### Practitioner Commitment

Six studies discussed the importance of practitioners being committed to clients, and of clients perceiving them as such (AEF 2018; Cherney 2024; Davies 2023; Fisher et al. 2020; Gielen 2020; Uhlmann 2017). This reflected the importance of staff being responsive to client requests, including outside of normal working hours and at weekends (AEF 2018; Gielen 2020; Uhlmann 2017). This level of commitment meant emerging issues could be addressed rapidly, although those high caseloads and staff shortages identified earlier can limit practitioners' capacity to respond to requests immediately, or to engage with clients as often as they would like (Uhlmann 2017). Such commitment can also facilitate relational processes, as being perceived as genuine and caring can help build trust (Fisher et al. 2020, 25) and in motivating change (Cherney 2024).

However, the intensity of this work, and practitioners' commitment to their clients can place practitioners under a great deal of psychological and mental pressure (AEF 2018; Uhlmann 2017; Cherney 2024). Particularly as the flexible nature of CVE work means it is not always clear to practitioners when they have ‘done enough’ (AEF 2018, 36). Tension may also arise if practitioners do not feel that partners in other agencies are similarly committed to a young person (Davies 2023). Although such pressure was common across different fields, the particular sensitivity of CVE work may create specific pressure (Uhlmann 2017).

###### 5.2.3.3.2 Client‐Centred Approach

Eighteen studies examined implementation factors that together highlighted the importance of programmes using client‐focused approaches (AEF 2018; Brett and Kahlmeyer 2017; Cherney 2024; Cherney et al. 2018; Cherney et al. 2022; Chisholm and Coulter 2017; Davies 2023; Gielen 2020; Grossman and Barolsky 2019; Kolbe 2019; Langdon‐Shreeve and Nickson 2021; Oberg et al. 2023; Osorno Hernandez 2021; Ottis 2016; Rousseau et al. 2024; SPLC and PERIL 2021; Uhlmann 2017; Weine et al. 2018).

Evidence relating to these factors was primarily qualitative, drawn from qualitative (*n* = 12) and mixed methods studies (*n* = 5) assessed as being high quality based on two‐thirds scoring positively on 9 (*n* = 4) or all 10 (*n* = 7) of the CASP domains. This data was supplemented with Kolbe's (2019) quasi‐experimental study that highlighted how a client‐focused approach might help to improve programme completion rates. Although this study had a serious risk of bias, it provided evidence of how working to address client‐specific needs might facilitate engagement with interventions and ultimately prevent young people from becoming engaged with violent groups. This study, coupled with the large number of higher‐quality qualitative and mixed‐methods studies, represents a robust evidence base with which to assess the importance of adopting a client‐centred approach.

Overall, this evidence highlights that a client‐centred approach that was individualised, developmentally appropriate, and culturally sensitive was crucial for delivering positive outcomes, and therefore spoke to the *effectiveness* domain of the RE‐AIM framework.

####### Individualised Approach

The importance of individualised approaches was discussed in sixteen studies (AEF 2018; Brett and Kahlmeyer 2017; Cherney et al. 2022a; Cherney 2024; Chisholm and Coulter 2017; Davies 2023; Gielen 2020; Grossman and Barolsky 2019; Kolbe 2019; Langdon‐Shreeve and Nickson 2021; Oberg et al. 2023; Osorno Hernandez 2021; Ottis 2016; SPLC and PERIL 2021; Uhlmann 2017; Weine et al. 2018).

Tailoring support to individual clients was considered important because it reflected both the ‘uniqueness of individuals’ (Davies 2023, 133) and the highly individualised nature of radicalisation processes (Brett and Kahlmeyer 2017). This emphasis on ‘individualisation’ (Cherney et al. 2022a) was reflected in the widespread use of case management and mentoring approaches that were specifically tailored to the needs of each young person or each family. Studies examining such programmes consistently emphasised the importance of taking a ‘person‐centred’ (Davies 2023) or ‘needs‐based’ (Brett and Kahlmeyer 2017) approach. For example, Uhlmann (2017) concluded that ‘an essential feature of good counselling is an individualised, case‐based, flexible approach’ in their analysis of the BAMF Advice Centre in Germany (p. 39). Weine et al. (2018) and Grossman and Barolsky (2019) advocate for the use of case‐managed CVE approaches when examining potential models for delivering secondary prevention work in the United States and support for returnees in Australia, respectively, for the same reason.

Some individuals may need support in meeting more basic needs or may require more intensive forms of support when such needs are not currently being met. Examples might include providing support relating to housing (AEF 2018; Langdon‐Shreeve and Nickson 2021); providing practical support, such as ‘transporting clients to appointments’ (Cherney 2024, 57); and ‘helping youth to organise daily routines and manage their daily living, which will have a bearing on their capacity to engage in an intervention’ (Cherney et al. 2022a, 54). Complementing more formal intervention with informal forms of support – particularly when working with clients who ‘are very high need’ – was also seen as helping establish the ‘conditions necessary for ongoing and intense engagement, helping to generate client change in the long term’ (Cherney 2024, 57).

Studies pointed to the importance of flexibility (Uhlmann 2017) and creativity (AEF 2018; Cherney et al. 2022a) when working with individuals. The extent to which creative and flexible responses were possible was seen as both a function of interventions leaving space for creativity and of practitioners having the confidence and skills to respond creatively. At the same time, a lack of clear direction was seen as potentially constraining creativity, with Chisholm and Coulter (2017) noting how a ‘lack of clear guidance’ around how to handle radicalisation cases ‘had a knock‐on effect on the ability of frontline staff to respond to a case flexibly and take “risks” or unorthodox approaches’ (p. 30).

####### Developmentally Appropriate Approaches

Seven studies discussed the importance of being responsive to developmental considerations (Cherney et al. 2022a; Davies 2023; Gielen 2020; Grossman and Barolsky 2019; Oberg et al. 2023; Osorno Hernandez 2021; Rousseau et al. 2024).

These studies primarily discussed the importance of programmes being age‐ and/or developmentally appropriate (Grossman and Barolsky 2019; Cherney et al. 2022; Osorno Hernandez 2021; Rousseau et al. 2024).^31^ For example, experts interviewed by Cherney et al. (2022a) argued that practitioners working with youth need to be adept at managing the ‘various ruptures/setbacks that youth may experience and that are a part of adolescence’ (p. 58). This study also stressed the importance of practitioners recognising that ‘youth are at an important stage in their lives where they are trying to assert control over their identity’ (Cherney et al. 2022a, 53). One UK practitioner interviewed by Osorno Hernández (2021) adopted a similar perspective when interpreting the disruptive behaviours of pupils who ‘have been moved across foster homes and schools’, which they saw as an attempt ‘assert control’ in the only space where they felt that this was possible – the classroom (p. 176). This perspective meant that this practitioner advocated for ways of working that helped young people regain a sense of control in their lives.

Adopting a trauma‐informed perspective, including recognising and responding to the developmental effects of trauma, was also seen as important when working with youth (Cherney et al. 2022a; Oberg et al. 2023; Rousseau et al. 2024). The importance of being responsive to the needs of neurodivergent youth (Davies 2023; Rousseau et al. 2024) was also noted. Although studies primarily discussed how developmental factors might impact work with young people, Gielen (2020) also stressed the importance of family support groups being designed with similar considerations in mind.

####### Cultural Sensitivity

Seven studies highlighted the importance of cultural sensitivity (AEF 2018; Cherney et al. 2018; Gielen 2020; Grossman and Barolsky 2019; Langdon‐Shreeve and Nickson 2021; Uhlmann 2017; Weine et al. 2018). Ensuring that teams possess ‘necessary cultural and language skills’ (Cherney et al. 2018, 9) and ‘a high level of cultural sensitivity’ (AEF 2018, 50) and deliver services such as psychological support in culturally sensitive ways are all important (Grossman and Barolsky 2019; Langdon‐Shreeve and Nickson 2021). Training and guidance around culturally sensitive issues was therefore important, particularly for staff lacking confidence around such topics (Cherney et al. 2018; Langdon‐Shreeve and Nickson 2021). These points speak to the broader importance of client‐practitioner compatibility, another important implementation factor examined below.

###### 5.2.3.3.3 Ways of Working

Fourteen studies examined specific ways of working that were seen as important when working with young people (AEF 2018; Brett and Kahlmeyer 2017; Cherney et al. 2022; Chisholm and Coulter 2017; Cifuentes et al. 2013; Davies 2023; Gielen 2020; Grossman and Barolsky 2019; Langdon‐Shreeve and Nickson 2021; Oberg et al. 2023; Osorno Hernandez 2021; Rousseau et al. 2024; Skiple 2020; Uhlmann 2017).

All data relating to ways of working were qualitative, drawn from qualitative (*n* = 9) and mixed methods (*n* = 5) studies, two‐thirds of which scored positively on 9 (*n* = 2) or all 10 (*n* = 7) CASP domains. These data were therefore considered robust overall and served to highlight how practitioners and study authors saw empowering and dialogical approaches as helping to motivate young people and/or their families to engage with interventions in ways that were more likely to produce positive outcomes. These ways of working, therefore, spoke directly to the *effectiveness* domain of the RE‐AIM framework.

####### Dialogue and Discussion

Nine studies discussed how practitioners worked to encourage dialogue and discussion (Adams et al. 2023; Chisholm and Coulter 2017; Cifuentes et al. 2013; Davies 2023; Gielen 2020; Osorno Hernandez 2021; Rousseau et al. 2024; Skiple 2020; Uhlmann 2017). These studies emphasised the importance of practitioners adopting a ‘neutral’ (Gielen 2020), ‘non‐biased’ (Uhlmann 2017), or ‘non‐judgemental’ (Davies 2023) approach when engaging with young people and/or their families. Such approaches were considered particularly important when engaging in ideological discussions and determining whether and how to challenge youth who express problematic views (Davies 2023; Osorno Hernandez 2021; Adams et al. 2023). Whilst exploring ideology and politics was likely to be important when working with some clients, the focus of such discussions was more commonly on understanding the individual's mindset, rather than directly challenging or attempting to change their views (Adams et al. 2023). However, practitioners also stressed that challenging views are sometimes appropriate and needed.

Engaging in reflective practice was seen to help practitioners to adopt this approach. Four studies noted how reflecting on biases or preconceptions they might hold towards people who are drawn towards extremism was important (Cherney et al. 2022; Rousseau et al. 2024). Recognising and receiving training on the relevance of trauma and trauma‐informed practice is noted as particularly important (Rousseau et al. 2024). Practitioners interviewed by Oberg et al. (2023) also noted how their mindset towards radicalised individuals had changed towards ‘wanting to understand’ and ‘to help clients who were at risk of acting in a criminal or violent way’ after receiving training on trauma (p. 1755).

Four studies discussed how group work – particularly group work that brought peers with differing opinions together – was a useful forum for dialogue and debate (Chisholm and Coulter 2017; Cifuentes et al. 2013; Osorno Hernandez 2021; Skiple 2020). These studies discussed programmes establishing ‘safe spaces’ (Osorno Hernandez 2021) or ‘open forums’ (Cifuentes et al. 2013) so that youth were both given assurance that they were free to share their views (within appropriate limits) but were also fully aware that their views would likely be challenged by providers or peers who might hold alternative positions. A notable and commonly cited example of this type of approach is the Tolerance Project in Sweden, which facilitates dialogue between at‐risk youth and more tolerant peers through a programme of group‐based work over several weeks (Skiple 2020).

####### Empowerment and Agency

An emphasis on empowering youth and families was noted in nine studies (AEF 2018; Brett and Kahlmeyer 2017; Cherney et al. 2022a; Chisholm and Coulter 2017; Davies 2023; Grossman and Barolsky 2019; Langdon‐Shreeve and Nickson 2021; Osorno Hernandez 2021; Uhlmann 2017). These studies discussed the importance of giving youth a sense of control and of building their capacity to pursue positive alternatives to extremism.

Studies emphasised the importance of giving young people a sense of agency in both a theoretical and a literal sense. In regard to the former, AEF (2018) stressed the importance of intervention goals being client‐led, and Cherney et al. (2022a) argued that ‘[a]ssistance needs to give youth a sense of mastery over their identity formation and a sense of purpose in life and not dictate the process or direction’ (p. 54). More practically, Davies (2023) interviewed one practitioner who was deliberately ‘more passive’ in early interactions with young people, including in some cases ‘letting them lead during initial sessions’ (p. 131). This was seen as giving young people a sense of agency, whilst also providing an opportunity for the practitioner to identify any potential ‘underlying issues’.

The empowerment of youth was often a stated objective of programmes, with several studies identifying constructs such as ‘self‐esteem’ (Langdon‐Shreeve and Nickson 2021) or ‘resilience’ (Osorno Hernandez 2021) as crucial outcomes that were seen as useful in countering radicalisation. Empowerment was also often identified as a mechanism through which these outcomes were achieved. For example, Uhlmann (2017) identified empowerment or ‘helping people help themselves’ (p. 38) as an important standard when counselling families and peers. This was one of several studies that spoke of leveraging or activating ‘resources’ (Uhlmann 2017); ‘pro‐social strengths and resilience assets’ (Grossman and Barolsky 2019); or ‘supportive factors found within the extended family’ (Chisholm and Coulter 2017) when working with at‐risk and radicalised young people and/or their families. This emphasis on leveraging existing strengths pointed to the use and relevance of more strengths‐based approaches in this context (see Marsden 2017).

Helping youth to identify and pursue ‘positive alternatives’ (Brett and Kahlmeyer 2017) to violent extremism was a key feature of these approaches – for example, by activating those psychological mechanisms identified above, or providing more material or practical support (Langdon‐Shreeve and Nickson 2021). This ‘positive focus’ (Langdon‐Shreeve and Nickson 2021, 50) or emphasis on ‘human development’ (Osorno Hernandez 2021) was seen as distinct from risk‐oriented approaches that have traditionally dominated CVE practice. However, even studies advocating for such an approach stressed a continuing need for ongoing risk assessment and management (Grossman and Barolsky 2019).

###### 5.2.3.3.4 Contextualised Approach

Seventeen studies discussed how interventions and practitioners adopted a contextualised approach when working with young people and their families (AEF 2018; Cherney et al. 2022a; Cherney 2024; Cherney et al. 2018; Chisholm and Coulter 2017; Davies 2023; Foster 2010; Grossman and Barolsky 2019; Kolbe 2019; Langdon‐Shreeve and Nickson 2021; Osorno Hernandez 2021; Ottis 2016; Rousseau et al. 2024; Sahgal and Kimaiyo 2020; Skiple 2020; SPLC and PERIL 2021; Uhlmann 2017).

Relevant evidence was primarily drawn from high‐quality qualitative (*n* = 11) and mixed‐methods (*n* = 5) studies, with 11 of the 16 studies assessed using the CASP tool scoring positively on nine (*n* = 3) or all 10 of the domains (*n* = 8). This was complemented by Kolbe's (2019) quasi‐experimental study that had a serious risk of bias, but provided relevant insights into how a contextualised approach, and working with families in particular, can support implementation. Taken together, there was robust data to illustrate how working with families and across different levels of social context impacted different domains of the RE‐AIM framework: supporting programmes to engage with young people (*Reach*) and to intervene in their radicalisation directly and indirectly (*Effectiveness*).

####### Working With Families

Fourteen studies stressed the importance of engaging with families (AEF 2018; Cherney et al. 2022a; Cherney 2024; Chisholm and Coulter 2017; Davies 2023; Foster 2010; Grossman and Barolsky 2019; Kolbe 2019; Langdon‐Shreeve and Nickson 2021; Ottis 2016; Rousseau et al. 2024; Skiple 2020; SPLC and PERIL 2021; Uhlmann 2017). Although most clearly reflected in programmes that intervene indirectly in youth radicalisation by adopting families as intervention clients (AEF 2018; Uhlmann 2017), family engagement was also seen as ‘critical to the success of youth CVE interventions’ that worked directly with young people (Cherney et al. 2022, 54).

Supportive families were seen as helping to facilitate the delivery of services and support to young people (Cherney et al. 2022a; Cherney 2024; Chisholm and Coulter 2017; Ottis 2016; Skiple 2020). Key roles that families were seen as potentially performing included providing and confirming important information about the young person; helping to monitor their progress; assisting them in attending appointments or enroling in courses; and motivating the young person to remain engaged with a programme (Cherney et al. 2022; Cherney 2024). They were also seen as providing a ‘countervailing pressure’ that might serve to supplement the effects of services provided to youth (Ottis 2016).

Direct work with families was also often an aspect of holistic intervention plans for at‐risk or radicalised youth. Interventions may help young people to reconnect with their families; mediate family conflicts; or facilitate family therapy or counselling to address specific needs that young people might have, and/or to mitigate potential issues that might prevent them from engaging with a programme (Chisholm and Coulter 2017; Kolbe 2019).

It was noted that families will often require support if they are to intervene in a young person's radicalisation or support the efforts of interventions working with their child (Cherney et al. 2022a; Cherney 2024; Grossman and Barolsky 2019; Uhlmann 2017). The importance of such support was most clearly reflected in those interventions designed specifically for families (AEF 2018; Uhlmann 2017), but the utility of a more ‘whole‐of‐family approach’ (Grossman and Barolsky 2019) was also noted in studies examining programmes working directly with youth. For example, Cherney et al. (2022a) note how parents may ‘have specific needs (e.g., health and financial) influencing the degree to which they could support their child’ and may themselves require support. In such cases, providing ‘separate counselling processes [for parents and for their children] that strategically align with each other’ may therefore be useful (Cherney et al. 2022a, 56).

However, challenges relating to familial engagement were also noted. For example, parents may be distrustful and unwilling to engage with programmes or consent to their child's participation in them – particularly programmes that are government funded and/or involve some police engagement (Cherney et al. 2022a; Chisholm and Coulter 2017; Langdon‐Shreeve and Nickson 2021; Rousseau et al. 2024). And, because interventions such as Channel in the United Kingdom must obtain parental consent before they work with a young person aged under 18, the opportunities for intervening in some cases can be limited when this consent is not obtained or is withdrawn – although, as noted in the section on enroling clients above, specific approaches such as joint visits to families have been cited as having ‘improved uptake’ of Channel support (Langdon‐Shreeve and Nickson 2021). Depending on the context, interventions may be able to refer youth to alternative forms of support, such as that provided by community‐based providers who may be viewed more favourably than statutory agencies or police (Langdon‐Shreeve and Nickson 2021).

Challenges related to parental consent may be particularly acute in cases where families are themselves a potential source of radicalisation or hold racist or extremist views – a point that is discussed in detail below (Cherney et al. 2022; Chisholm and Coulter 2017; Davies 2023; Langdon‐Shreeve and Nickson 2021; Rousseau et al. 2024; Skiple 2020; SPLC and PERIL 2021). In such cases, engaging with families to tackle such views is likely to be particularly important (Foster 2010), but will not always be possible.

####### Socio‐Ecological Approaches

The importance of working with families speaks to the broader utility of socio‐ecological approaches, and of recognising how the social context(s) might contribute to risk or resilience (Ellis et al. 2022). Ten studies examined the use of such approaches (Cherney et al. 2018; Grossman and Barolsky 2019; Langdon‐Shreeve and Nickson 2021; Osorno Hernandez 2021; Ottis 2016; Rousseau et al. 2024; Sahgal and Kimaiyo 2020; Skiple 2020; SPLC and PERIL 2021; Uhlmann 2017). A good example is the ‘contextual safeguarding’ approach used by practitioners in the United Kingdom, which considers broader, familial, and peer influences when working with youth (Langdon‐Shreeve and Nickson 2021).

These studies highlighted how programmes should be sensitive to and address more contextual sources of vulnerability, such as those reflected in specific community and peer group contexts (SPLC and PERIL 2021). Experiences of social stigmatisation and other more systemic or structural barriers that youth might face in specific contexts were also important issues for interventions to work through (Osorno Hernandez 2021). At the same time, the broader social environment can also be an important source of strength and resilience, as explicitly emphasised in those studies reporting on programmes that directly engage families (Uhlmann 2017); as well as those that aim to leverage positive peer group influence (Cherney et al. 2018; Skiple 2020).

The importance of context was also noted when diverting people away from extremism, with studies discussing the importance of socialising youth into pro‐social networks (Grossman and Barolsky 2019; Ottis 2016; Rousseau et al. 2024; Sahgal and Kimaiyo 2020). Building support systems across different contexts, including within families, peer groups, communities, and schools, was therefore seen as important for preventing and countering youth radicalisation (Rousseau et al. 2024; SPLC and PERIL 2021). This is not without its challenges, particularly when trying to leverage communities who may lack the resources to support young people who are exiting violent extremist contexts and may be unwilling to do so (Grossman and Barolsky 2019). Consultation and engagement with communities may therefore be important to help build their capacity and their willingness.

#### 5.2.4 Analysis of Moderators (Objective III)

Twenty‐seven of the studies included in the review examined moderators that impacted implementation. These moderators are shown in Table 21.

**Table 21:** Moderators.

Category	Moderator
National and local context (*n* = 21)	– Local structures and histories (*n* = 13) – Localised practices (*n* = 10) – Political, economic, and cultural context (*n* = 8) – Public and political discourse (*n* = 8) – Broader policy context (*n* = 6)
Client characteristics (*n* = 19)	– Changing caseloads (*n* = 5) – Gender (*n* = 6) – Client history with criminal justice (*n* = 3) – External challenges (*n* = 3) – Familial radicalisation (*n* = 10)
Delivery context (*n* = 4)	– Delivery context (*n* = 4)

##### 5.2.4.1 National and Local Context

Twenty‐one studies examined how features of national and local context might impact programme delivery (AEF 2018; Brett and Kahlmeyer 2017; Cherney et al. 2018; Chisholm and Coulter 2017; Davies 2023; Fisher et al. 2020; Foster 2010; Gielen 2020; Grossman and Barolsky 2019; Joyce and Lynch 2017; Kolbe 2019; Langdon‐Shreeve and Nickson 2021; Osorno Hernandez 2021; Ottis 2016; Sahgal and Kimaiyo 2020; Skiple 2020; SPLC and PERIL 2021; Thomas et al. 2017; Uhlmann 2017; UNDP 2022; Weine et al. 2018).

The role of national and local context was primarily evidenced by data drawn from qualitative studies (*n* = 14) and qualitative research conducted as part of mixed‐methods studies (*n* = 5). This evidence was assessed as being of moderate quality based on half (*n* = 10) of these studies scoring positively on 9 or 10 of the CASP domains. Overall, the strength of evidence was therefore assessed as robust. This evidence highlighted how specific features of local and national contexts affected how interventions function.

###### 5.2.4.1.1 Local Structures and Histories

Thirteen studies discussed how national and local histories, structures, and resources might shape delivery in different ways (AEF 2018; Brett and Kahlmeyer 2017; Cherney et al. 2018; Chisholm and Coulter 2017; Davies 2023; Gielen 2020; Grossman and Barolsky 2019; Langdon‐Shreeve and Nickson 2021; Osorno Hernandez 2021; Ottis 2016; Thomas et al. 2017; Uhlmann 2017; Weine et al. 2018).

Some countries have national institutions such as the BAMF Advice Centre (Uhlmann 2017) and Forsa and the Family Support Centre (AEF 2018) that facilitated CVE work.^32^ These institutions were found to have supported local practitioners in Germany and the Netherlands, respectively, by providing information and guidance, as well as providing help‐seekers with important information and access to services. Gielen (2020) suggested that family support groups in the Netherlands be organised using a model similar to the BAMF Advice Centre in Germany, whereby a national organisation would facilitate and supervise support delivered by local organisations. However, national bodies may face challenges when working across regional boundaries, as the working practices of different local partners ‘places different demands on each collaboration’ (AEF 2018, 37). Regardless, these examples demonstrate that structures and resources supporting CVE work can vary markedly across country contexts in ways that can affect delivery.

The resources available for CVE work can also vary across regions within countries. This makes it important that efforts to establish local programming are underpinned by a good understanding of the capacity of local providers, and any gaps in provision (Grossman and Barolsky 2019; Cherney et al. 2018). Pre‐existing relationships between local multi‐agency partners and with local communities were seen to support the design and implementation of CVE work (Langdon‐Shreeve and Nickson 2021; Osorno Hernandez 2021; Ottis 2016; Weine et al. 2018). Programmes do not need to ‘start from scratch’ (Ottis 2016) when they can adapt and ‘build on existing frameworks and programming’ (Ottis 2016, 75–76). Adapting existing programmes, when possible, may therefore be more efficient than introducing entirely new CVE interventions (Weine et al. 2018).

However, the maturity and formalisation of such networks can vary, as can the level of local experience and knowledge relating to radicalisation (Cherney et al. 2018; Chisholm and Coulter 2017; Langdon‐Shreeve and Nickson 2021), and the range of potential local partners that are available (Brett and Kahlmeyer 2017). Local actors *may* therefore need to ‘start from scratch when relevant relationships, frameworks, and structures are not established’, which can ‘cause a lag in addressing immediate issues’ (Ottis 2016, 66). There may also be regional variations in the type of funding available, as noted above (Chisholm and Coulter 2017; Langdon‐Shreeve and Nickson 2021; Osorno Hernandez 2021; Thomas et al. 2017). This can result in the local delivery and availability of relevant programmes varying markedly within countries (Chisholm and Coulter 2017; Langdon‐Shreeve and Nickson 2021; Ottis 2016). In some cases, specific local actors might also be fundamental to the success of a programme in a given context (Brett and Kahlmeyer 2017).

Levels of community trust in CVE work can differ markedly in ways that can both facilitate and inhibit implementation. Whilst levels of trust may be high in some communities owing to a particular history of engagement (Ottis 2016), especially those where extensive outreach has already been undertaken to address potential concerns (Chisholm and Coulter 2017; Thomas et al. 2017), local police activity has been shown to create challenges when trying to recruit young people into CVE programmes (Davies 2023).

###### 5.2.4.1.2 Localised Practices

The importance of programmes being tailored to local contexts was highlighted in 10 studies (Brett and Kahlmeyer 2017; Fisher et al. 2020; Foster 2010; Gielen 2020; Grossman and Barolsky 2019; Langdon‐Shreeve and Nickson 2021; Ottis 2016; Skiple 2020; SPLC and PERIL 2021; Uhlmann 2017). Important features of a ‘context‐tailored response’ (Grossman and Barolsky 2019) included: partnering with or employing credible practitioners with strong levels of local knowledge (Brett and Kahlmeyer 2017; Fisher et al. 2020); tailoring programmes according to the needs and challenges facing local communities (Foster 2010; Ottis 2016; SPLC and PERIL 2021) whilst taking care to avoid the risk of ‘aggravating sensitivities in the local environment’ that can emerge when communities feel targeted (Foster 2010, 39); and adapting training that is delivered alongside programmes in accordance to local needs, whilst raising awareness of local challenges and risks (Langdon‐Shreeve and Nickson 2021; Skiple 2020). Giving local stakeholders space to tailor programmes was therefore seen as important because they are often ‘best placed … to decide how things work best within their particular communities and within their structures’ (practitioner quoted in Ottis 2016, 74). The Geographical features of the region may also be relevant when designing and delivering interventions, for example, limiting distances between interventions/providers and clients (Gielen 2020).

###### 5.2.4.1.3 Political, Economic, and Cultural Contexts

The impact of political, economic and cultural factors was noted in eight studies (Brett and Kahlmeyer 2017; Grossman and Barolsky 2019; Joyce and Lynch 2017; Kolbe 2019; Langdon‐Shreeve and Nickson 2021; Sahgal and Kimaiyo 2020; Skiple 2020; UNDP 2022). The wider political context can create challenges and opportunities. For example, studies examining the implementation of STRIVE I and II in the Horn of Africa discuss how working in contexts with elevated rates of political violence can create practical challenges, including creating potential risks for programmes and their staff (Brett and Kahlmeyer 2017), as well as potentially contributing to increased violent sentiment amongst local youth (Sahgal and Kimaiyo 2020). Political developments such as contested elections and the rise of specific political parties or increasing political focus on issues that might be leveraged by extremist groups (e.g., migration) might also contribute to a rise in intolerant or extremist views amongst youth in both the Global South (Sahgal and Kimaiyo 2020) and Global North (Skiple 2020). In contrast, an absence of violence can be a useful tool in contexts that were previously marked by high rates of political violence, with practitioners working in Northern Ireland noting how this was helpful in convincing at‐risk youth that ‘the time is not right’ for acts of political violence (Joyce and Lynch 2017), for example. This links to a broader point made by Kolbe (2019) noted above, whereby the effectiveness of an intervention might be ‘geographically and temporally limited’ to a specific political and cultural context that facilitates its implementation.

Programmes benefit from being delivered in ways that reflect the ‘geocultural’ (Grossman and Barolsky 2019) or economic context in which they are working, considering both the opportunities and barriers that these contexts can produce. Broader international events can also impact delivery. Most notably, two studies discussed the impact of the COVID‐19 pandemic (Langdon‐Shreeve and Nickson 2021; UNDP 2022), for example, by necessitating a shift towards online delivery (Langdon‐Shreeve and Nickson 2021).

###### 5.2.4.1.4 Political and Public Discourse Relating to CVE

Eight studies examined how broader public and political discourses around radicalisation and CVE can potentially impact practitioners and communities (AEF 2018; Cherney et al. 2018; Chisholm and Coulter 2017; Grossman and Barolsky 2019; Langdon‐Shreeve and Nickson 2021; Osorno Hernandez 2021; Ottis 2016; Uhlmann 2017).

Working in the ‘highly sensitive, highly politicised field’ of CVE places practitioners under a great deal of scrutiny and pressure (Uhlmann 2017, 46; also, AEF 2018; Grossman and Barolsky 2019). Practitioners in Australia discussing the sensitive topic of reintegration work for returnees from conflict zones noted how ‘inaccurate or sensationalised media coverage could undermine efforts to reintegrate returnees’ by making communities wary of such efforts (Grossman and Barolsky 2019, 94). Speaking to broader concerns about how problematic media discourses around radicalisation and CVE were negatively impacting Muslim communities in the Global North (Cherney et al. 2018), this study also identified concerns about communities being targeted by the media should a family be repatriated (Grossman and Barolsky 2019).

These broader discourses might also make some communities and organisations distrustful of CVE programming, and therefore reluctant to engage with or deliver CVE work, particularly government‐funded activities (Cherney et al. 2018; Chisholm and Coulter 2017; Langdon‐Shreeve and Nickson 2021; Osorno Hernandez 2021). However, effective community engagement and outreach was able to mitigate some of these concerns (Langdon‐Shreeve and Nickson 2021). Although negative reporting about CVE programming might not always reflect the perceptions or experiences of local communities (Ottis 2016). This points to the importance of programmes having an accurate understanding of local community perceptions towards relevant issues, with one study discussing the importance of conducting research on community sentiment towards the potential reintegration of returnees (Grossman and Barolsky 2019), for example.

###### 5.2.4.1.5 Broader Policy Context

Six studies discussed the intersection between CVE and other policy areas, and associated opportunities and challenges (Cherney et al. 2022a; Foster 2010; Grossman and Barolsky 2019; Langdon‐Shreeve and Nickson 2021; Osorno Hernandez 2021; Weine et al. 2018). The actual or perceived overlap between CVE and other policy areas can be a challenge, as it can create confusion as to the distinctiveness of, and need for, CVE work amongst local stakeholders (Foster 2010), and potentially contribute to a perception that CVE is ‘an unwarranted addition’ (Cherney et al. 2022a). CVE activities therefore benefit from trying to ‘compliment services and add value, given youth who are vulnerable to radicalisation may already be receiving assistance’ (Cherney et al. 2022a, 55). In some contexts, broader policies or legislation might create implementation challenges, for example, around information sharing (Grossman and Barolsky 2019).

The overlap between radicalisation and other social issues, or between CVE and other types of programming, can be beneficial. This can provide the foundation for establishing more integrated approaches that consider radicalisation alongside a broad range of other harms (Osorno Hernandez 2021), or for leveraging existing experience and structures as noted above (Weine et al. 2018). When CVE and other related processes operate in a complementary way, they can be mutually reinforcing. For example, processes that ensured that young people referred into local Prevent teams were also assessed for other safeguarding issues (and vice versa) were seen well‐placed to identify issues that might not otherwise be picked up (Langdon‐Shreeve and Nickson 2021). This again highlights the importance of effective multi‐agency working arrangements and information sharing (Langdon‐Shreeve and Nickson 2021).

##### 5.2.4.2 Client Characteristics

Nineteen studies examined how the specific, often changing, features of caseloads affected how programmes worked (Adams et al. 2023; AEF 2018; Cherney et al. 2022; Cherney 2024; Chisholm and Coulter 2017; Cifuentes et al. 2013; Davies 2023; Foster 2010; Gielen 2020; Glaser 2017; Grossman and Barolsky 2019; Kolbe 2019; Langdon‐Shreeve and Nickson 2021; Rousseau et al. 2024; Sahgal and Kimaiyo 2020; Skiple 2020; SPLC and PERIL 2021; Thomas et al. 2017; Uhlmann 2017). Although one of these studies specifically noted how clients sharing a common language, religion, and nationality mitigated the need for adaptation in a way that was helpful (Kolbe 2019), the remaining studies emphasised the importance of interventions being responsive to differences between clients. This section focuses on these 18 studies, which were assessed as being of moderate to high quality based on the CASP quality assessment, with over half scoring positively 9 (*n* = 3), or all 10 (*n* = 7) CASP domains, and which therefore provide strong evidence of how different client characteristics impact delivery. However, none of the included studies examined whether and how implementation varied across different age groups (e.g., between children and adolescents) in detail, which represents a key limitation of the evidence base that we return to in the conclusions.

###### 5.2.4.2.1 Changing Nature of Caseloads

Five studies discussed how the changing nature of programme caseloads impacted how programmes worked (Adams et al. 2023; AEF 2018; Davies 2023; Langdon‐Shreeve and Nickson 2021; Uhlmann 2017). These studies highlighted different ways in which caseloads had changed over time, emphasising the importance of programmes being able to adapt to what is an ‘extremely dynamic’ field (Uhlmann 2017, 6).

Being able to respond to ‘the changing landscape of radicalisation’ (Langdon‐Shreeve and Nickson 2021, 79) was seen as important when working with clients and delivering training. This included being alert to shifts in the ideologies young people are being drawn into; the rise of new and ‘emerging threats’ (Davies 2023); and the increase in ‘complex case configurations’ (Uhlmann 2017, 5) linked to, for example, mental health issues or neurodiversity (Langdon‐Shreeve and Nickson 2021; Uhlmann 2017; Davies 2023) that require practitioners to be adaptive in their responses (Davies 2023). Such complexity also means that CVE programmes benefit from assessing or considering whether other safeguarding issues are present when working with young people and connecting young people to other service providers when needed (Langdon‐Shreeve and Nickson 2021). Studies were positive about how programmes had adapted to changing caseloads. However, an increasing prevalence of nebulous or unstable ideologies in caseloads was seen as a particular challenge (Adams et al. 2023; Langdon‐Shreeve and Nickson 2021).

###### 5.2.4.2.2 Gender

Six studies discussed considerations relating to gender (Davies 2023; Grossman and Barolsky 2019; Glaser 2017; Sahgal and Kimaiyo 2020; Thomas et al. 2017; Uhlmann 2017). Several of these studies emphasised the importance of recruiting female providers and/or ensuring a gender balance amongst programme staff to better support different clients (Grossman and Barolsky 2019; Sahgal and Kimaiyo 2020; Thomas et al. 2017; Uhlmann 2017). For example, the STRIVE II mentoring programme in Kenya made efforts to recruit female mentors ‘to ensure that female mentees felt comfortable sharing information about their lives and circumstances’ (Sahgal and Kimaiyo 2020, 127).^33^ Some practitioners were found to hold gendered assumptions about female roles in violent extremism or to perceive women as lacking agency in ways that were unhelpful to delivering relevant programming (Davies 2023; Glaser 2017). Although it is important to avoid flawed assumptions, programmes must be responsive to gendered differences between clients, as recruitment methods and programmes developed for males may be less effective in engaging and impacting females (Glaser 2017; Davies 2023). This speaks to a wider challenge whereby women may face cultural barriers that might inhibit their ability to fully engage with programmes in some contexts (Sahgal and Kimaiyo 2020).

###### 5.2.4.2.3 History With Criminal Justice System

Three studies highlighted how police activity or young people having some history of past contact with the criminal justice system can create challenges for programmes seeking to engage youth (Cherney et al. 2022; Davies 2023; Oberg et al. 2023). Past personal experience of the criminal justice system was seen as potentially making young people reluctant to engage with programmes (Cherney et al. 2022; Oberg et al. 2023). Davies (2023) discusses how a past history of youth being stopped and searched on their way to a gym that was delivering a relevant intervention had deterred youth from attending, until the intervention provider contacted the police and was able to mediate the issue.

###### 5.2.4.2.4 External Challenges

The fact that clients may face external challenges outside the control of intervention providers was noted in three studies (Cherney et al. 2022a; Cherney 2024; Cifuentes et al. 2013). This included psychological challenges such as ‘fluctuations in mental health and general motivation’ (Cherney 2024, 57), as well as broader challenges in an individual's personal, social, or family life (Cherney et al. 2022a; Cherney 2024; Cifuentes et al. 2013). Because such issues can negatively impact a young person's ability and desire to engage with programmes, practitioners need to be cognisant of emerging and external challenges when seeking to interpret any stalled progress or regression (Cherney et al. 2022a). Effective communication between partners when such challenges emerge improves the chance that they are ‘dealt with effectively’ (Cifuentes et al. 2013).

###### 5.2.4.2.5 Familial Radicalisation

Issues linked to familial radicalisation and/or potential radicalising or extremist influences within families were discussed in 10 studies (Cherney et al. 2022a; Chisholm and Coulter 2017; Davies 2023; Foster 2010; Gielen 2020; Grossman and Barolsky 2019; Langdon‐Shreeve and Nickson 2021; Rousseau et al. 2024; Skiple 2020; SPLC and PERIL 2021).

The radicalising influence of parents and other family members was noted in several studies. In some cases, the radicalisation of a family member may be the reason why a young person is considered at risk and/or in need of intervention (Gielen 2020; Rousseau et al. 2024). In the most severe cases, youth who are ‘socialised into extremism early in their life by immediate and extended family members and friends’ (Cherney et al. 2022a, 53‐54; Rousseau et al. 2024), including those returning from territory previously controlled by the Islamic State (Grossman and Barolsky 2019), may present with developmental or psychological issues that should be addressed through intervention (Rousseau et al. 2024). The ongoing presence of radicalising influences within the family can also create challenges, making it harder to ‘limit their exposure to such social environments’ and to identify alternative, positive social influences to support their rehabilitation (Cherney et al. 2022a, 54) and placing youth in ‘a position of considerable emotional and moral ambiguity’ (Grossman and Barolsky 2019, 56). Practitioners have also raised concerns about young people being ‘coached’ by parents so as to avoid suspicion (Chisholm and Coulter 2017).

In cases of parental radicalisation, practitioners argued that programmes should ‘leverage and reinforce supportive factors found within the extended family’ (Langdon‐Shreeve and Nickson 2021, 24). Providing ‘sustained support for at‐risk youth outside of the home’ (SPLC and PERIL 2021, 5), including delivering support in less stigmatising settings such as schools (Rousseau et al. 2024) was also seen as important in such cases, as was being cognisant of – and working in ways that minimise the risk of re‐enforcing – the distress and stigma that can result from parental radicalisation (Rousseau et al. 2024).

##### 5.2.4.3 Delivery Context

Four studies highlighted how specific settings helped to facilitate intervention work both directly and indirectly (Grossman and Barolsky 2019; Langdon‐Shreeve and Nickson 2021; Kolbe 2019; Rousseau et al. 2024). The impact of different delivery settings was evidenced by both qualitative and quantitative data drawn from a mix of qualitative (*n* = 2), mixed methods (*n* = 1) and quantitative (*n* = 1) studies. The small numbers of studies that reported on delivery context means that the strength of evidence is limited.

Schools were seen as useful settings for engaging with children and adolescents, and as ‘a second home’ that provides an important space for positive socialisation (Grossman and Barolsky 2019). Educational settings were also suggesting as helping to ‘provide a route through which other interventions could be delivered’ (Langdon‐Shreeve and Nickson 2021, 50). It was also noted that some parents may also be more willing for their children to receive therapy in school on the basis that schools are ‘perceived as a less stigmatizing setting’ than clinical spaces (Rousseau et al. 2024, 693).^34^

Home‐based services can also be impactful. This is most clearly evidenced by Kolbe (2019), who, as noted above, highlighted how participating in home‐based social services in an unnamed East African city alongside group‐based work was associated with a decreased likelihood of joining a violent extremist group. However, there are opportunities and challenges associated with home‐based work. For example, whilst practitioners in Canada interviewed by Rousseau et al. (2024) reported that (extremist) parents may see the opportunity to host them at their home ‘as a way of establishing a more balanced relation through the rituals of hospitality’, they also suggested that other parents may see this as a ‘further intrusion of their lives’ (Rousseau et al. 2024, 693). In such instances, these practitioners suggested that schools can be a useful ‘middle ground’ for youth work.

### 5.3 Summary of Main Results

This review set out to examine the effectiveness (Objective I) and implementation (Objective II) of secondary and tertiary interventions for children and adolescents that operated outside of the criminal justice system and to identify relevant implementation factors and moderators (Objective III). Only one evaluation of effectiveness using an eligible research design was identified and included in the review, reflecting wider limitations in the evidence base underpinning the field of counter‐radicalisation more broadly (Lewis, Marsden, Cherney, et al. 2024). However, a more expansive body of work (*n* = 29) examining implementation and associated barriers, facilitators, and moderators was identified.

#### 5.3.1 Objective I: Effectiveness

It is not possible to draw robust conclusions about the effectiveness of non‐criminal justice interventions from one eligible, quasi‐experimental evaluation – particularly as the study author concluded that the results of this evaluation were likely to be specific to the particular context of the unnamed East African city in which the intervention operated (Kolbe 2019). This evaluation found that complementing a group‐based intervention for at‐risk young people (aged 13–18) with home‐based social work support had a positive effect on intervention completion rates and on relevant primary outcomes (i.e., whether a young person subsequently joined a violent group), However, caution is needed when interpreting the results of this study as it was assessed as having a serious risk of bias.

#### 5.3.2 Objective II: Implementation

The evidence relating to implementation is stronger than that on effectiveness but remains limited. Seven studies examined whether specific interventions were being implemented as intended (*n* = 6) and/or whether they had successfully identified and worked with their target audience (*n* = 4). These studies identified a number of important lessons that are likely relevant to broader efforts to counter youth radicalisation, including the importance of a clearly defined and well‐evidenced theory of change in supporting implementation and evaluation; the utility of evaluating and validating interventions against existing research evidence and against professional standards; and the importance of practitioners adapting their approach to meet evolving/emerging needs.

These evaluations also highlighted how an intervention's likely impact on countering cognitive and behavioural radicalisation rests on its ability to first identify and engage at‐risk or radicalised individuals. When stated, evaluations were generally positive on this point. However, challenges associated with identifying those most in need of support (i.e., those most at risk) were also seen as potentially limiting an intervention's impact.

#### 5.3.3 Objective III: Implementation Factors

A wide variety of implementation factors were identified in this review. Three broader categories of implementation factors were examined: structural features; processes of identifying and engaging clients; and ways of working with young people and families.

##### 5.3.3.1 Structural Features of Interventions

###### 5.3.3.1.1 Knowledge and Expertise

The importance of different forms of knowledge, expertise, and/or experience – and/or associated knowledge‐building activities – was examined in 21 studies. Relevant forms of knowledge and expertise included *specialist knowledge* relating to (counter‐) radicalisation (*n* = 11), including ideological knowledge and, potentially, a personal history of radicalisation; *practice‐based knowledge* (*n* = 10) from fields such as social work or youth work; and expertise relating to *mental health, neurodiversity, and/or trauma* (*n* = 12), reflecting a recent rise in such issues in intervention caseloads (see Lewis, Marsden, Hewitt, et al. 2024).

Interventions able to draw on *multidisciplinary forms of knowledge* were considered most likely to be effective (*n* = 12). The availability of multidisciplinary expertise was seen as benefiting clients (by ensuring they are able to access different types of services) and practitioners (by providing access to guidance or advice from multidisciplinary partners). Studies also discussed how different skills and knowledge were needed to perform different intervention functions, emphasising the benefits of a multidisciplinary team.

*Training and learning* were therefore seen as crucial (*n* = 15). Specialist CVE practitioners were seen to benefit from ongoing training and from updating their knowledge in response to emerging trends. Training for non‐specialists was also seen as important for building the knowledge and confidence needed to deliver a CVE function, particularly given studies highlighted how a lack of knowledge, experience, or confidence might inhibit practitioners from performing a CVE function outside of their normal role. Knowledge‐sharing activities between individuals and institutions were also seen as beneficial.

###### 5.3.3.1.2 Community Working

Fifteen studies highlighted how partnerships with community and civil society actors were often crucial, particularly when such actors worked directly with clients. It was considered important for programme funders – particularly government – to build equitable partnerships with credible and established community and civil society actors knowledgeable about local issues. Community partnership and engagement were also seen as crucial in building trust and buy‐in, and for ensuring that programming meets local needs. Community support was also cited as important in supporting the long‐term rehabilitation of young people. In some contexts, community‐led programming may also be used to support those reluctant to engage with government intervention.

Government agencies were seen to perform an important coordination function within such partnerships. However, risks associated with excessive control and monitoring were noted, and some local actors may wish to remain independent from the government.

###### 5.3.3.1.3 Multi‐Agency and Partnership Working

Nineteen studies examined how different models of multi‐agency and partnership working functioned. Multi‐agency networks were cited as improving *efficiency* (*n* = 5), providing structures for mobilising local resources, avoiding duplication of effort, and helping individual institutions overcome resource issues. These structures also provided a platform through which young people could access *different forms of support* (*n* = 10). Partnership working was facilitated by *relational processes* between institutions and individuals (*n* = 9); partnerships having *clearly defined roles* and high levels of coordination (*n* = 5); partners working towards *shared objectives* and having a mutual understanding of each other's priorities (*n* = 9); and effective and efficient *information sharing* (*n* = 8) facilitated by clear processes and procedures and strong levels of trust. However, *engaging partners* who lack confidence or understanding of their potential role within such structures was found to be challenging (*n* = 6). Issues relating to *information sharing* were also identified (*n* = 8), commonly relating to the sharing of information between police and partners.

###### 5.3.3.1.4 Police Involvement

Thirteen studies discussed partnership working with criminal justice agencies. The police were often crucial partners in multi‐agency structures, performing functions such as making referrals and contributing to risk assessments. However, police involvement also had the potential to make some people more reluctant to engage with interventions. Some practitioners also worried about how potentially having to contact the police when there was a more immediate risk to or from a client might undermine efforts to build trust. Putting relevant safeguards in place, for example, restricting access to information only to police staff with a relevant role within a multi‐agency counter‐radicalisation structure, and being transparent about the nature of police involvement can help to address some of these concerns. Clearly defined criteria and processes for when law enforcement should be contacted can also support practitioners in making what can be difficult decisions.

###### 5.3.3.1.5 Staff Supervision and Support

Seven studies discussed how *institutional support* and different forms of *supervision* and quality assurance can facilitate implementation. Different types of informal and formal support were seen to facilitate delivery, and as potentially useful mechanisms of quality assurance (*n* = 5). Informal discussions about cases with colleagues were cited as helping to support decision‐making. More formalised processes, such as partnering new staff with senior colleagues, developing protocols and other guidelines, and regular, scheduled meetings to discuss cases, helped to improve delivery. Other methods of quality assurance and supervision identified by studies (*n* = 4) included independent case review processes, employing process supervisors or similar, and establishing quality standards.

###### 5.3.3.1.6 Resourcing Programmes

The importance of interventions being adequately resourced was discussed in 15 studies. Different types of *time constraints* (*n* = 6) – whether caused by short‐term funding horizons, the particular intensity of CVE intervention work, high caseloads, or staffing shortages – were seen as potentially limiting the duration, frequency, and intensity of practitioners' contact with their clients in ways that might be unhelpful. *Funding and resourcing* issues (*n* = 13) were also seen as creating potential challenges. Although the government was identified as an important source of funding, several challenges relating to government funding were identified. These included its short‐term and unstable nature; regional variations in its availability (in some countries); and some institutions being unwilling or unable to access government funding owing to reputational concerns. Avoiding an over‐reliance on government funding by accessing alternative funding sources instead of or in addition to government funding was therefore seen as useful.

Finally, opportunities and challenges associated with *staffing and recruitment* were also identified (*n* = 6), particularly in relation to recruiting individuals with specialist expertise. High workloads, low salaries, and a lack of permanency were all seen to make recruitment and retention harder. High staff turnover and short‐term contracts also made it harder to retain knowledge. Issues relating to staffing quality and motivation were also noted, and the importance of professionalisation and professional standards was, in turn, identified as important. Funding was again seen as crucial in supporting efforts to recruit and retain staff, but owing to the challenges described above, such funding was not always available.

##### 5.3.3.2 Identifying and Engaging Eligible Clients

###### 5.3.3.2.1 Identifying Eligible Clients

Sixteen studies examined processes that enabled interventions to identify and reach at‐risk and radicalised young people and their families. *Partnership working* (*n* = 12) with institutions such as schools and social care providers and/or with credible local actors embedded in local communities was seen as useful in this regard. Although interventions can struggle to engage with local institutions that do not see the value in or need for a CVE intervention. Studies also stressed the importance of *interventions being accessible* to local actors (*n* = 9). Relevant factors include programmes being visible and known to individuals who might seek help from them, and referral mechanisms and processes being well‐defined and understood. Ten studies also emphasised the need for *target audiences and associated eligibility criteria to be clearly defined*, which was considered particularly important given the subjectivity inherent in identifying radicalisation risk, and associated concerns about ‘under and over identification’ (Skiple 2020), Different tools and approaches for improving the accuracy of referrals were therefore used in different contexts including developing detailed referral forms; providing clear guidance around when (and when not) to make a referral; and seeking advice from colleagues or partners.

###### 5.3.3.2.2 Engaging Eligible Clients

Thirteen studies examined how interventions adopted and engaged eligible young people and/or their families. Research examining the *accuracy and consistency of case adoption decisions* (*n* = 9) identified different methods considered useful in supporting case adoption decisions, including joint screening with external partners or specialist in‐house teams, and using specific risk assessment tools or eligibility criteria. However, some level of subjectivity was again noted in research examining practitioners' adoption thresholds.

An *efficient intake procedure* was identified as important (*n* = 8) and was characterised by features including short waiting times, accurate and efficient risk and needs assessments, limited bureaucratic or administrative barriers, and effective transfer of information and cases between partners. *The accessibility of services* was also seen as important (*n* = 4), with relevant considerations including selecting settings that are accessible to clients or providing services in different languages. Online delivery may potentially help to improve the accessibility of some services, but challenges with online engagement were noted.

Ten studies discussed the process of *enroling and engaging clients*. Although there was insufficient evidence from which to draw a definitive conclusion, several studies stressed the importance of interventions being voluntary. However, there was some concern that individuals might face implicit, and sometimes, explicit, pressure to participate in voluntary programmes. Challenges in obtaining consent were also identified.

##### 5.3.3.3 Working With Young People and Families

###### 5.3.3.3.1 Relational Processes

Relational processes between practitioners and young people and families were considered crucial facilitators (*n* = 20). *Trusting and equitable relationships* were seen as vital (*n* = 16) in motivating and sustaining engagement with interventions over time, and ultimately in motivating positive, long‐term change. Studies emphasised the importance of practitioners working to build trust from the first interaction with a client, whilst also noting that this can be challenging and time‐consuming. Building trust with families was important in obtaining parental consent and for ensuring that emerging issues were identified quickly. Practitioners must therefore possess the requisite skills and personalities needed to build relationships with and motivate young people.

*Selecting practitioners who are best‐suited to working with specific individuals* was one potential way of facilitating these processes (*n* = 10). Most commonly, this involved matching young people with practitioners with a similar background or life history, although it was noted that this type of matching does not ‘guarantee success’ (Cherney et al. 2018). In some cases, interventions also brought youth at risk from right‐wing extremism into contact with practitioners from communities stereotyped and misrepresented by right‐wing organisations (e.g., Muslim communities) to challenge such stereotypes, although this needs to be managed carefully and on a case‐by‐case basis.

The *commitment of practitioners* was fundamental in building trust and motivation (*n* = 6). Studies stressed the importance of practitioners being responsive to client requests, although this was not always possible due to time constraints and high workloads. Such responsivity helped address emerging issues whilst demonstrating the care and commitment of practitioners to clients. However, such commitment can place practitioners under psychological and mental pressure, particularly given the sensitivity of counter‐radicalisation work. Tension might also arise when practitioners feel that colleagues or partners are not similarly committed to the welfare and care of a young person.

###### 5.3.3.3.2 Client‐Centred Approach

Eighteen studies discussed the importance of practitioners adopting a client‐centred approach that is *individualised* (*n* = 16), *developmentally appropriate* (*n* = 7), and *culturally sensitive* (*n* = 7). Individualisation was a common feature of many interventions, particularly those using case management and mentoring approaches. Relevant practices included delivering packages of support that targeted different needs; providing more intensive support and addressing basic needs for clients who are in greater need; and providing less formal support when required. The importance of practitioners being flexible in how they work with individual clients was also noted, although the scope for flexible or creative responses was at times constrained by a lack of confidence or guidance.

A *developmentally appropriate* approach was considered to include, for example, being sensitive to the search for identity that is often a feature of adolescence; responsive to the needs of neurodivergent youth; and cognisant of the developmental effects of trauma. Working in *culturally sensitive ways* – and receiving relevant training – was also considered important, including when delivering specific services such as psychological support.

###### 5.3.3.3.3 Ways of Working

Fourteen studies examined specific ways of working that were seen as important for facilitating positive change, including facilitating constructive *dialogue and discussion* (*n* = 9), particularly when engaging with topics relating to ideology; and focusing on *empowerment and agency* (*n* = 9): both as important outcomes within interventions that focused on building strengths and skills, and as important mechanisms for delivering positive change. Approaches that were seen as helping to facilitate dialogue included adopting neutral or non‐confrontational positions and establishing ‘safe spaces’ (Osorno Hernandez 2021) for a constructive exchange of (often competing) views. Engaging in reflective practice and training on the impact of trauma helped practitioners adopt the type of non‐confrontational position needed. Approaches that foregrounded empowerment and agency included practitioners collaboratively setting goals with clients or letting young people lead sessions to give them a sense of agency over their own journey.

###### 5.3.3.3.4 Contextualised Approach

Seventeen studies discussed the utility of adopting a contextualised approach, and of considering strengths and vulnerabilities that might exist in the broader familial or social environment. *Family engagement* was seen as a crucial element of such an approach (*n* = 14). Although particularly relevant to more indirect forms of prevention that worked through families to interrupt radicalisation, family engagement was also seen as crucial when working directly with young people – particularly when parental consent was required. Families were seen as important partners who could provide relevant information, monitor progress, help to keep young people engaged and motivated, and compound the effects of direct services through their interactions with young people at home. Providing support for families to help them perform these functions was therefore seen as important. Family therapy and counselling were also sometimes an important component of holistic intervention plans developed for young people. However, engaging with families was not always easy, for example, when they are distrustful of interventions, or when they themselves hold racist or extremist views. Work with families is likely to be particularly important in such cases, but simultaneously harder to initiate and to sustain.

The utility of *socio‐ecological approaches* that work across different familial, community, and environmental contexts was also noted (*n* = 10). Such approaches offered a foundation for addressing contextual sources of vulnerability, including more systemic or structural issues that young people might face. They also provide a framework for leveraging strengths and resources that exist in young people's contexts, such as the positive influence of families and peers. Engaging communities and other relevant actors and institutions was, therefore, often an important element of working with a young person.

#### 5.3.4 Objective III: Moderators

##### 5.3.4.1 National and Local Context

Twenty‐one studies identified different ways in which the specific features of local and national contexts might impact implementation. CVE work can be facilitated by local/national *structures and histories* (*n* = 13). For example, some countries have specialist national institutions that provide support to local practitioners and help‐seekers – although such institutions must also navigate regional differences in working practices, which can be challenging. Specific local histories of multi‐agency and community relationships can also facilitate the development and delivery of CVE interventions. However, the maturity of local networks, levels of radicalisation expertise, and range of potential partners vary across and within countries. Alongside funding disparities, these issues can impact programme availability across regions. Local actors and community trust can support programme implementation. However, levels of local trust and engagement are shaped by historical experience with government agencies and the police, which has the potential to create challenges when trying to engage young people in CVE programmes.

Ten studies discussed the importance of *localised practice*, including employing and partnering with local practitioners; adapting programmes to community needs while avoiding stigmatisation; and customising training to address local challenges. Involving local stakeholders in programme design and delivery can help to ensure that interventions reflect the needs of local communities. So too can providing space for local stakeholders to adapt how they deliver CVE work according to their experience of ‘what works’ when working with local partners and communities. Taking account of geographical considerations, for example, by minimising travel distances for clients, is also important.

*Political, economic, and cultural factors* can also positively and negatively impact delivery (*n* = 8). Political instability, violence, and contentious elections can create the context for violent extremism and inhibit programme delivery, whilst peaceable contexts might afford more positive conditions for interventions. Cultural barriers, including gender biases, have the potential to hinder engagement in interventions in different ways. Whilst global events, notably the COVID‐19 pandemic, can impact how programmes are delivered.

*Public and political discourse*s also impact delivery (*n* = 8). Practitioners face significant scrutiny, including pressure from politicised narratives and sensationalised media coverage, which has the potential to undermine reintegration efforts and fuel community distrust. These discourses can also generate scepticism towards programmes, particularly when funded by the government, which can hinder efforts to engage young people. Effective outreach can mitigate this. However, negative reporting does not always reflect local perceptions, and an accurate understanding of local sentiment is important.

Six studies explored the *broader policy context* and the intersection of CVE with other policy areas. Broader policy and legislative barriers, like information sharing restrictions, can hinder implementation, whilst perceived overlap with different policy areas can create confusion about the distinctiveness of, and need for, CVE. At the same time, programmes benefit from trying to complement existing services, and recognising the overlap between radicalisation and other social issues can facilitate integrated approaches, addressing radicalisation alongside other harms, and leveraging existing structures.

##### 5.3.4.2 Client Characteristics

Nineteen studies examined how different client characteristics impacted programme delivery, further underlining the need for interventions to employ client‐centric approaches that reflect the particular characteristics and needs of different individuals. This included five studies which examined the *changing nature of programme caseloads*, emphasising the need for programmes to adapt to evolving and emerging ideologies and threats, and growing case complexity. Programmes also benefit from assessing broader safeguarding issues and connecting individuals to relevant services.

Research examining topics relating to *gender* (*n* = 6) emphasised the importance of programmes being responsive to the needs and preferences of female clients, including by ensuring that they are able to work with female providers when preferred, whilst avoiding gendered assumptions that undermine their agency. Recognising that cultural barriers can impede female participation also underlines the need for tailored approaches.

Specific *external challenges* (*n* = 3) related to mental health difficulties and personal and social issues, and a past history of (negative) *engagement with the police or the criminal justice system* (*n* = 3) were identified as potentially inhibiting engagement with programmes. Practitioners must take account of the former when interpreting progress and consider how such challenges might stall or inhibit participation and change.

Ten studies discussed the issue of *familial radicalisation*. Although families were often identified as key partners in efforts to counter radicalisation, in some cases, parental and family influence have the potential to increase radicalisation risk. Early exposure to extremism within families can cause developmental and psychological issues that need to be addressed through appropriate support. Negative family influences can also hinder interventions, limiting positive influence and creating emotional ambiguity for the client. Programmes can leverage extended family members and provide sustained out‐of‐home support, including school‐based interventions, to counteract negative familial influences.

##### 5.3.4.3 Delivery Context

Four studies emphasised the impact of delivering interventions in specific settings, including schools and homes. Schools provide a familiar and potentially less stigmatising environment for youth engagement and support, facilitating positive socialisation and access to interventions. Parents may also prefer school‐based therapy over clinical settings. There is some evidence that home‐based services can reduce the likelihood of radicalisation. However, while some parents may welcome practitioners, others may perceive it as intrusive. Schools can serve as a valuable ‘middle ground’ (Rousseau et al. 2024) in such cases.

#### 5.3.5 Overall Completeness and Applicability of Evidence

It is not possible to draw firm conclusions as to the effectiveness of the interventions and practices examined in this review. Evidence of effectiveness was drawn from only one study, the author of which explicitly noted that the reported findings were likely to be context specific (Kolbe 2019). Relevant insights relating to effectiveness were identified in several other studies included in this review, most notably the two studies that were able to draw links between programme activities and self‐reported outcomes by examining an intervention's underlying theory of change (Brett and Kahlmeyer 2017; Fisher et al. 2020). Whilst these studies provide insights into how programmes work, they do not provide robust evidence of impact. Their findings are also likely to be context‐specific.

The evidence base relating to implementation is far stronger, albeit with some limitations. Seven studies were identified that examined the fidelity of an intervention, that is, whether it was implemented as intended, and/or worked with relevant clients. These studies formed part of a larger evidence base comprising 29 studies that examined implementation factors and moderators that impacted how interventions were delivered. These studies were international in nature, spanning a diverse range of countries and contexts, as well as different types of intervention. Although some of the findings are likely to be highly context‐specific – particularly given that both local context and delivery context were identified as important moderators – the higher‐order themes identified in the review appear to have relevance across contexts. This is evidenced by the fact that 8 of the 12 categories of implementation factors examined in this review were identified in 50% or more of the studies included in the analysis of implementation factors.

There are, however, three notable gaps in the evidence presented in this review. The first is the lack of research that has examined whether and how counter‐radicalisation work differs between different age groups (i.e., between children and adolescents) in any depth. The second is the lack of research that specifically examines whether and how interventions work differently with youth compared to adults. Although several of the interventions examined in this review worked with children, adolescents, and adults, there was often limited comparison of youth and adult cohorts. This means that some of the evidence examined in this review – particularly when drawn from studies that were included based on a significant proportion of the intervention caseload being aged 19 or under – will not be specific to younger cohorts and will instead reflect broader practice. Most notably, whilst there is a significant body of research that examines whether and how interventions might tailor their work to young people (see section on client‐centred approaches), there has been limited empirical research examining whether and how practitioners take account of developmental considerations when assessing risk (and needs) and using associated tools in this context, which is likely to be crucial component of any effort to deliver developmentally appropriate interventions as described above.

The third key evidence gap is the lack of empirical research that has specifically examined opportunities and constraints associated with delivering relevant interventions outside of the criminal justice system instead of through the criminal justice system. This type of evidence would have provided further insights into the assumed benefits of responding to youth radicalisation outside of the criminal justice system outlined earlier.

#### 5.3.6 Quality of the Evidence

This review confirmed that efforts to understand the effectiveness of secondary and tertiary CVE interventions continue to be inhibited by a lack of experimental and quasi‐experimental research. The searches only identified two robust quasi‐experimental studies – only one of which reported on outcomes related to effectiveness – and no relevant experimental studies. This review, therefore, primarily relies on qualitative and non‐experimental quantitative research. There are clear limitations with both types of research design that should be considered when interpreting the results of this review.

The quality of evidence relating to effectiveness is low, given the absence of experimental research and the fact that only one eligible quasi‐experimental study with a serious risk of bias was identified. This reflects broader weaknesses in the evidence relating to secondary and tertiary CVE prevention identified in previous reviews (Lewis, Marsden, Cherney, et al.^2024^), and the methodological and ethical challenges that have restricted the use of quasi‐experimental designs in all but a small number of cases (e.g., Webber et al. 2018).

A much larger body of evidence relating to implementation was identified, comprising 29 studies. This evidence was primarily drawn from qualitative research. Although we recognise that quality assessments of qualitative research are prone to bias and subjectivity (Soilemezi and Linceviciute 2018), all qualitative research included in this review was assessed as being of medium‐to‐high quality based on scoring positively on between 7 and 10 of the CASP domains. All qualitative methodologies were assessed by at least two reviewers to minimise the risk of one assessment biasing the inclusion/exclusion decision. Although the strength of quantitative evidence was weaker, it provided important, complementary evidence to the qualitative data. There was a high degree of consistency across the studies included in the analysis of implementation, as most implementation factors and moderators were examined in at least half of the studies.

#### 5.3.7 Potential Biases in the Review Process

Two potential sources of bias have already been discussed in the review. First, we accept that other research teams may have reached different conclusions when reviewing the various sources of ambiguity we identified above, particularly when delineating between primary and secondary prevention. Interventions delivered in the Global South that identified risk at the collective and community level or used overly broad criteria were excluded, even when they were described as working with ‘at‐risk’ youth. This broad approach to identifying risk does not align with dominant understandings of secondary prevention used in the Global North, whilst the risks of defining vulnerability at the collective level or using broad criteria are well‐established in research examining secondary prevention in different contexts (Wallner 2021). Whilst we do not want to downplay the importance of research conducted in the Global South and include several studies of interventions that met our own definition of secondary prevention delivered outside the Global North, evidence from studies that took a broader approach to identifying risk had less relevance to this review, or to policy, and were therefore excluded.

Second, the inclusion of qualitative research introduced a degree of bias into the screening process, as discussed in the previous section. However, as noted above, all qualitative methodologies were reviewed by at least two reviewers and were only included when consensus was reached. Excluding studies with a critical weakness, as defined above, helped ensure the quality of the included qualitative research. The fact that all studies scored positively on at least 7 of the 10 domains of the CASP tool also increased our confidence in the quality of the methodologies underpinning these studies.

#### 5.3.8 Agreements and Disagreements With Other Studies or Reviews

The findings align with previous reviews in several ways. Most obviously, reviews examining secondary and/or tertiary prevention work with youth have also pointed to a lack of evidence of effectiveness (Cherney et al. 2022; Wallner 2021; White 2021). Notably, Wallner (2021) drew this conclusion despite also including insights drawn from a broader range of interventions – including those working with ‘at risk’ youth as defined at the collective level described above – owing to a lack of clarity as to how the outcomes from such interventions specifically related to countering radicalisation. This reflects a broader weakness in the evidence base underpinning the wider CVE field, with previous reviews of secondary and tertiary programming also identifying the same evidence gap around effectiveness (e.g., Lewis, Marsden, Cherney, et al. 2024; Hassan et al. 2021a, 2021b).

The analysis of implementation also aligns with previous reviews of youth‐focused CVE work and related reviews of case management and mentoring interventions (DuBois and Alem 2017; Lewis, Marsden, Cherney, et al. 2024; Winterbotham 2020) in terms of those factors that can facilitate implementation. One of the most consistent themes reflected in these reviews is the importance of interventions being adequately and efficiently targeted (Cherney et al. 2022; Wallner 2021; Winterbotham 2020). For example, Winterbotham (2020) notes that ‘[w]here mentees are poorly selected, the impact on [violent extremism] will likely be reduced’ (p. 30). Wallner (2021) similarly concludes that ‘[b]etter targeting strategies are needed to allocate resources efficiently and avoid the marginalisation of already vulnerable groups’ (p. 32). This reflects one of the key implementation factors identified in this review, as well as some of the broader challenges that we have already discussed around adequately defining ‘at‐risk’ segments of the population, as well as the limitations of defining risk at the collective level or based on overly broad criteria.

Another consistent theme that overlaps with our review is the importance of relational processes, and the relationship between practitioners and young people (Wallner 2021; DuBois and Alem 2017; Lewis, Marsden, Cherney, et al. 2024; Winterbotham 2020). The ways of working that were identified as important for effectively working with youth in this review also overlapped with methods and approaches discussed in previous reviews of youth CVE work (Cherney et al. 2022; Wallner 2021) and of mentoring work in this space (DuBois and Alem 2017; Winterbotham 2020). The importance of engaging with families was also specifically reflected by Cherney et al. (2022a), and in a review of family‐oriented interventions by Haugstvedt (2022), which examined indirect forms of prevention.

Other reviews make more definitive claims about whether such approaches are likely to be effective, which this review cannot do based on the evidence available. However, such analyses identify important considerations. For example, Wallner (2021) emphasises the limitations (and potential risks) of interventions focused on empowerment and raises related concerns about some interventions taking an overly simplistic approach by focusing on singular factors or being guided by simple explanations for radicalisation. Although concerns about an emphasis on empowerment are a potential point of divergence with our review, our observation that empowerment is often a mechanism of change rather than an outcome aligns with Wallner's argument. Our review similarly agrees with Wallner and others in stressing the importance of interventions reflecting the complexity and heterogeneity of radicalisation processes and individuals. This is most clearly seen in the emphasis on taking an individualised, multidisciplinary approach.

## 6 Authors' Conclusions

The review found very little research that has assessed the effectiveness of interventions working with youth outside the criminal justice system. One study met our inclusion criteria (Kolbe 2019), which means that there is insufficient evidence of effectiveness to draw definitive conclusions. Importantly, an absence of such evidence is not evidence of ineffectiveness. We note the growing use of more robust non‐experimental designs when evaluating youth‐focused interventions that have been able to produce evidence of effectiveness, even if such studies do not meet the threshold for inclusion in our own analysis of effectiveness (e.g., Sahgal and Kimaiyo 2020). Nor does this absence of evidence suggest that responding to childhood and adolescent radicalisation through the criminal justice system is likely to be more effective. Indeed, because previous reviews have noted a similar lack of evidence for interventions delivered through the criminal justice system (e.g., Lewis, Marsden, Cherney, et al. 2024; Cherney et al. 2022a), it is more accurate to say that the field currently lacks robust evidence relating to efforts to counter (youth) radicalisation more broadly.

The field has developed a better understanding of the factors that facilitate or create barriers to the implementation of interventions. This body of work covers a range of areas, including structural, organisational, relational, and individual client‐level factors. It offers a solid foundation from which to develop a more evidence‐based approach to policy and practice in this area and provides a number of avenues for future research. And crucially, whilst many of the implementation factors identified in this review are also likely to be relevant to interventions delivered through the criminal justice system, previous research has highlighted how criminal justice responses and associated interventions may face barriers relating to, for example, developing the relational and motivational processes that this review identified as being crucial in motivating and sustaining engagement with programmes (Lewis, Marsden, Cherney, et al.^2024^). Although empirical research examining the relative strengths and weaknesses of different approaches is needed, this suggests that non‐criminal justice interventions will be crucial to efforts to counter youth radicalisation.

### 6.1 Implications for Policy and Practice

The most obvious implication for policy and practice is to build evaluation into programme design. Notwithstanding the challenges to evaluating secondary and tertiary interventions, this effort should prioritise the use of stronger methodological designs that make it possible to demonstrate programme effectiveness.

The evidence based on implementation is stronger than for effectiveness, and points to a number of implications for policymakers and practitioners. These implications are likely to be relevant to the interventions reflected in the typology presented in Section 5.1.3 earlier, but are particularly pertinent to the use of case management and mentoring programmes that work with at‐risk and radicalised youth owing to the prevalence of such interventions within this review. The findings are therefore also likely to be relevant to interventions that use similar approaches to prevent targeted violence more broadly, such as Behavioural Threat Assessment and Management (BTAM) teams in the United States.

Although it is not possible to conclusively determine whether they are effective, there is a solid body of evidence highlighting how ways of working can help to facilitate implementation and practitioners' efforts to engage and motivate young people, which in turn have been suggested as (potentially) contributing to positive intervention outcomes. These include taking a youth‐centred approach that avoids securitising and stigmatising young people; a holistic, social‐ecological approach that foregrounds community engagement; multidisciplinary working that brings together expertise from a range of sectors; developing systems and structures that support all those involved in the intervention process; recognising the importance of relational processes that enable systems and structures to work effectively; alongside active and ongoing engagement with families. And, although there is again insufficient evidence to draw a firm conclusion on this point, non‐criminal justice interventions appear particularly well‐suited to this type of approach, given the potential barriers – particularly relating to securitisation and stigmatisation – associated with criminal justice responses that were identified above.

Interventions benefit from taking a *youth‐centred approach* that is tailored to the individual's needs and goals, and which takes account of the particular characteristics and evolving nature of this population. In practice, this translates to individualised intervention plans, developmentally appropriate and culturally sensitive interventions, and providing flexible, accessible, safe spaces for young people to gain the support they need. Although interventions that are specifically designed for youth cohorts are, by definition, likely to take a youth‐centred approach, interventions that work with youth as part of broader caseloads will also need to be cognisant of the need for such an approach given that several of the programmes examined in this review were not specifically designed for youth, but worked with a large proportion of clients aged 19 or under. Because interventions benefit from taking account of developmental considerations when working with young people, it also follows that such considerations should be reflected in the risk and needs assessment practices and tools used by interventions, but research examining the implementation of these tools and practices is limited at this time (Lewis, Marsden, Cherney, et al. 2024).

It is important that work with young people resists becoming over‐securitised and actively works to reduce the potential stigma young clients might experience. This process needs to recognise the changing nature of radicalisation dynamics, including shifting and sometimes unstable ideologies, and the increasing complexity of cases involving mental health and neurodiversity. Considering how the needs and preferences of male and female clients might differ is also important, for example, by partnering female clients with female practitioners when preferred, but so too is avoiding assumptions that risk overstating gendered differences and overlooking the agency of female youth. Recognising that negative experiences with the criminal justice system can hinder youth engagement underlines the need for practitioners to adopt a non‐judgemental and non‐biased perspective and to prioritise trust‐building with their clients.

The research points to the benefits of dialogue, creating safe spaces for discussion, and practitioner training on trauma‐informed practices. Finding ways of working that empower young people and recognise the importance of nurturing their agency in moving towards more positive futures is important. Interventions can do this by focusing on building self‐esteem, developing skills, and collaboratively setting goals. Strengths‐based approaches, which leverage existing protective factors and resources that might be available in their local context, help with this process.

The advantages of adopting a *socio‐ecological approach* were identified in the review. This recognises the interconnectedness of familial, community, and environmental factors and the role they can play, both as contextual sources of vulnerability and as spaces that provide strengths, protections, and opportunities for positive change. This can be enabled by partnering with communities and civil society actors in equitable, two‐way partnerships. Community partnerships have the potential to help build trust, engage those reluctant to take part in interventions, ensure programmes meet local needs, and support long‐term rehabilitation.

State agencies and national institutions have important roles to play in coordinating interventions, providing funding and quality assurance, and maintaining a focus on public safety. However, navigating local and regional differences and resource disparities can be challenging. Employing knowledgeable practitioners who understand their local setting and can utilise existing multi‐agency processes to adapt programmes to the local context and the individual client's needs can help navigate this challenge. Having input from local stakeholders means programmes are better able to align with community structures and needs, and nurture trust in ways that can help mitigate any negative historical experiences with government agencies.

The importance of *multidisciplinary working* was emphasised throughout the research. Interventions working with at‐risk and radicalised young people need diverse types of knowledge and expertise that are best provided through effective multi‐agency and/or multidisciplinary teams. This should include professionals with specialist knowledge of radicalisation, practical experience from social or youth work, and expertise in mental health, neurodiversity, and trauma. As well as providing knowledge and expertise, multi‐agency working has the potential to improve efficiency by mobilising resources from different contexts and avoiding duplication. Multidisciplinary working is best enabled when practitioners have comprehensive support and training; a clear understanding of their individual and joint roles, responsibilities and objectives; effective information sharing processes, and mutual trust. Concerns over police involvement and its potential to deter youth and families can be addressed by transparency about police involvement, clear information‐sharing protocols, and defined criteria for contacting law enforcement.

Effective *systems and structures* are crucial for supporting work with at‐risk or radicalised youth. This includes everything from the process of programme design, for example, through the development of a clearly defined theory of change to support implementation and evaluation, through to support for practitioners and the provision of quality assurance and professional standards. Training and development are vital. Specialist practitioners require role‐specific training and ongoing professional development opportunities, while non‐specialists need baseline knowledge. Knowledge sharing activities can support this effort, as can forums to address practitioner anxiety about the risks associated with this work. Institutional support, quality assurance, and supervision are important, as are informal and formal support, such as case conferences and mentorship. Quality assurance mechanisms, like independent case reviews and process supervisors, can help improve and assure delivery.

Adequate resourcing is vital. Without it, time constraints due to short‐term funding and high caseloads limit practitioner engagement and can lead to staffing and recruitment challenges, as well as bringing the potential for burnout. Ensuring programmes are appropriately resourced can help address challenges associated with low salaries and lack of permanency, which can impact retention and the development of best practice. Professionalisation and professional standards will further help the field mature. Organisations delivering interventions will benefit from diversifying funding sources, both to mitigate the risks of funding instability and to address reputational concerns about government influence, which can hinder engagement with communities and clients.

*Family engagement* is an important aspect of work with at‐risk or radicalised youth and should be a priority from the start of intervention planning. Families can provide insight and information to practitioners; help interpret progress; reinforce the positive effect of interventions; and help maintain engagement and motivation. However, families also need support. This can be direct support in the form of family counselling, or through family therapy, which can help address issues like estrangement, or through less formal types of support available through community networks. Notwithstanding these benefits, families can represent a risk when they distrust interventions or hold extremist views. In these cases, interventions may look to the extended family networks, wider community members, or find ways to provide sustained out‐of‐home support, such as in school or community settings.

Finally, *relational factors* are important for enabling interventions. This includes nurturing opportunities to develop trusting, equitable relationships with clients to support positive change. Interventions are supported when trust is built from the outset with the client and with families, both to support positive outcomes and to gain consent and help identify any issues as soon as they emerge. Practitioner commitment and responsiveness to client requests help facilitate the intervention process. However, to be sustainable, practitioners need ongoing support to mitigate the pressure and sensitivity of counter‐radicalisation work.

### 6.2 Implications for Research

The review highlights the need for more research using more robust methodologies to better understand the effectiveness of CVE interventions in general, and in particular, those designed to work with youth cohorts. Given the potential risks to young people from involvement with the criminal justice system, including stigma, securitisation and criminalisation, it is important to develop a stronger evidence base to inform policy and practice, and to understand how interventions can best support young people.

As well as stronger evaluations, research should focus on analysing the mechanisms that influence positive change. Drawing on realist evaluation methods, which focus on understanding what works, for whom, under what circumstances, can help with this, as such approaches are better able to identify relationships between contexts, mechanisms, and outcomes that underpin effective interventions (Gielen 2020). The review highlighted a range of implementation factors and moderators, spanning structural and contextual features, identification and referral processes, and client engagement strategies. These provide valuable insights for future research and can feed into realist evaluation designs that can help better understand the mechanisms that shape positive outcomes.

The review's conclusions regarding the importance of relational processes and client‐centred, socio‐ecological approaches highlight the importance of qualitative research that can interpret the dynamics of practitioner‐client relationships; the role of the wider environment, including the family setting; and the experiences of young people participating in interventions. Qualitative methods can illuminate the ways in which trust, empathy, and cultural sensitivity can contribute to positive outcomes, and can complement quantitative studies that focus on intervention outcomes. Longitudinal studies will also be important to help understand the long‐term effects of interventions and identify the factors that sustain positive change over time.

There is also a need for comparative research to understand the impact of different contexts, types of interventions, and delivery agents on outcomes. National and local structures, political contexts, and socio‐cultural factors have the potential to shape intervention processes and outcomes. Comparative studies across diverse settings can make visible how these contextual factors interact with intervention mechanisms, implementation processes, and outcomes, and help interpret whether and which aspects of interventions are transferable across contexts. Empirical research comparing the opportunities and constraints associated with working with youth inside and outside of the criminal justice system would also help to address a key evidence gap identified above.

A second kind of comparative research should examine the impact of interventions on clients with different kinds of characteristics. This includes research on the role gender, mental health, neurodiversity, and different age cohorts have on how young people respond to interventions. For example, studies comparing interventions for neurodivergent youth versus neurotypical youth can identify ways of adapting programmes effectively. Similarly, examining differences across age groups can shed light on developmentally appropriate strategies, whilst also addressing a key evidence gap. This comparative approach will enhance our understanding of both ‘what works’ and also ‘how’ interventions work (Lewis and Marsden 2024) and facilitate the development of more targeted, tailored and effective interventions.

Finally, comparing the implementation factors and moderators identified in this study with reviews of research on adult populations (e.g., Lewis, Marsden, Cherney, et al. 2024) will help identify more general factors that support effective case management at the structural or systems level, and which elements need to be adapted for youth cohorts. Together, this study agenda will strengthen the evidence underpinning policy and practice in an area that is growing in importance and help better understand and explain how and why young people can be more effectively diverted away from violent extremism and terrorism.

## Author Contributions

Content: James Lewis, Sarah Marsden, James Hewitt, Chloe Squires, Anna Stefaniak. Systematic review methods: James Lewis, Sarah Marsden, Anna Stefaniak. Statistical analysis: Anna Stefaniak. Information retrieval: James Lewis, Sarah Marsden, James Hewitt.

## Conflicts of Interest

The authors declare no conflicts of interest.

## Sources of Support

This review is funded by a Campbell Collaboration grant awarded to Sarah Marsden and James Lewis through the Campbell 5RD CVE programme.

## Supporting information

Review_Appendices_Resubmission.

## Endnotes

1 See World Health Organisation (WHO). n.d. Adolescent health. Retrieved from https://www.who.int/health-topics/adolescent-health.

2 See https://www.un.org/en/global-issues/youth.

3
https://www.dhs.gov/sites/default/files/2023-07/23_0724_opa_strategic-intelligence-assessment-data-domestic-terrorism.pdf.

4 See https://www.state.gov/foreign-terrorist-organizations/.

5
https://www.covidence.org/.

6 Available at https://www.ephpp.ca/quality-assessment-tool-for-quantitative-studies/.

7 Available at https://casp-uk.net/casp-tools-checklists.

8 In addition, the report by UNDP (2022) included evaluations of two other interventions, but as these evaluations were separate studies to the one included in the review, they are not included in this count.

9 It is also worth noting that the searches identified a number of records reporting on case management interventions that did not meet our inclusion criteria.

10 While some programmes are police‐led, the police do not play an active role in supporting or working with individuals, as per the definition of non‐criminal justice intervention used in this review.

11 Although evidence of their effectiveness remains limited, the assumed logic of these programmes is therefore increasingly understood as being sound: that individualised processes of radicalisation are best prevented and countered through an individualised, client‐focused approach (Lewis, Marsden, Cherney, et al. 2024).

12 Youth‐specific case management frameworks or programmes have been developed in some countries and context (e.g., Barracosa and March 2022; Ellis and Dalaine 2022) but no studies reporting on such programmes were eligible for inclusion. However, the study by Ellis et al. (2022) is particularly notable as many of the lessons identified by the authors (who were involved in developing the programme in question) clearly overlapped with the themes identified in this review.

13 The use of mentoring in this context is well‐established, and previous literature reviews have examined the implementation and effectiveness of mentoring programmes (e.g., DuBois and Alem 2017; Winterbotham 2020). However, many of the studies examining mentoring interventions did not report on findings specific to youth and so could not be included in the review.

14 Indeed, the STRIVE II programme team note in a ‘lessons learned report’ that the design of the mentorship component was informed by a similar mentoring intervention delivered as part of the earlier STRIVE Horn of Africa programme (see Brett and Kahlmeyer 2017) but was ‘more rigorous and included a greater focus on case management for the individual “at‐risk” youth’ (RUSI 2020, 3).

15 The boundary between primary and secondary prevention was particularly difficult to determine within this category owing to the prevalence of ‘targeted primary’ education programmes (Hassan et al. 2021a). These programmes more targeted than standard primary prevention programmes (i.e., those that are more universal) in that they focus on specific sub‐groups of the population, but not those who are specifically at risk.

16 The design and delivery of educational interventions for at‐risk youth is increasingly reflected in the literature, but a number of studies reporting on such interventions were excluded from the review as they did not meet our methodological inclusion criteria. See, for example, Savage, S. and Fearon., P. A, 2021. “Increasing Cognitive Complexity and Meta‐Awareness Among At‐Risk Youth in Bosnia‐Herzegovina in Order to Reduce Risk of Extremism and Interethnic Tension.” *Peace and Conflict: Journal of Peace Psychology* 27(2): 225; Broadbent, R. 2013. “Using Grass Roots Community Programs as an Anti‐Extremism Strategy.” *Australian Journal of Adult Learning* 53(2): 187–210.

17 As the review shows, dialogue and debate are a key feature of many programmes working with at risk youth. A number of studies that did not meet our inclusion criteria examined similar dialogical approaches. See, for example, Hussain, A., Pilkington, H., Simcock, K., Nicholas, J., Vickers, H., and Rogerson, L. 2019. Talking Our Way Out of Conflict: Critical Reflections on ‘Mediated Dialogue’ as a Tool for Secondary Level CVE. Report prepared for the UK Commission for Countering Extremism; Meringolo, P. 2020. *Preventing Violent Radicalisation in Europe. Multidisciplinary Perspectives*. Springer.

18 The potential impact of families and peers on preventing radicalisation is now reflected in the literature. See, for example, Ellefsen, R., and Sandberg, S. 2024. “Everyday Prevention of Radicalization: The Impacts of Family, Peer, and Police Intervention.” *Studies in Conflict & Terrorism* 47(10): 1342–1365.

19 Programmes that provided this type of support were only eligible for inclusion when they also worked to prevent radicalisation. For a broader review of such programmes, see Haugstvedt (2022).

20 It is also worth noting that the study by Kolbe (2019) was published in an open‐access journal that, as of August 2025, does not meet two criteria that are commonly used to validate open‐access journals, and which are often included in checklists for assessing whether a journal is potentially predatory: it is not indexed in the Directory of Open Access Journals; and the publisher is not listed as a member of the Committee on Publication Ethics (COPE) on the COPE website (see Laine and Winker 2017). In this context, it is also worth noting that, as of August 2025, this journal's publisher is listed in databases of potentially predatory publishers created by other researchers including https://beallslist.net and https://www.predatoryjournals.org. It is important to stress that none of the above is definitive evidence of predatory practices, and that such databases are open to challenge (see da Silva and Tsigaris 2018). It is also important to note that this journal/publisher meets a number of the other validatory criteria identified within such checklists, and are indexed in the ISSN International Centre's Directory of Open Access scholary Resources (ROAD) ‐ which means that the journal and publisher meet specific inclusion/validation criteria relating to publication practices used by that particular directory. We do not, therefore, offer any judgement as to whether this journal is predatory, and stress that we identified no specific concerns relating to the study itself. However, following consultation with the editors, we agreed that it was important to reference these points given ongoing discussion in the literature around the implications of including studies from potentially predatory publishers in systematic reviews (Munn et al. 2021; Rice et al. 2021).

21 These were not the only studies to present a theory of change but were the only studies to examine programme delivery against this theory of change in sufficient detail to be discussed in this section. Most notably, studies by Gielen (2020) and by Weine et al. (2018) developed a theory of change through their research, but did not specifically examine whether and how it was implemented.

22 To be eligible for mentoring, young people had to meet at least one *primary* criteria (Have a close peer or relative who has been recruited into a [violent extremist] group or is engaged in [violent extremist] activities; Be associated with violent criminals or gang members; Hold radical or extremist views and tendencies; Be affiliated with holders of extremist views and tendencies) and at least two *secondary* criteria (Being a former convict; Having a dysfunctional family background; Being a new convert to a religion; Being idle; Being a school dropout; Suddenly becoming socially withdrawn) (Fisher et al. 2020, 23).

23 Risks associated with ‘active profiling’ are noted in several studies pointing to the stigmatising effect this can have on those communities who are profiled (e.g., Badurdeen and Ndenyele 2024).

24 The validity and utility of using formers in this space continues to be debated in the literature, and to date, no definitive conclusion has been reached on this point. See, for example, Noar, A., DeMichele, M. T., McCann, W. S., Baker, D. R., Fisher, D. G., and Abel, M. N. 2023. *Review of Prevention Programming Undertaken by Allies Abroad to Identify Promising Practices: Study Findings Report*. U.S. Department of Homeland Security (DHS) Science and Technology Directorate (S&T).

25 However, these claims may not be believed, even when they are true (Rousseau et al. 2024).

26 There is a large body of literature which has examined whether and how different actors identify and report radicalisation, including a number of studies identified through the literature searches that examined whether and how youth workers and other youth‐focused practitioners specifically perform this function. The vast majority of this work did not meet our inclusion criteria as it did not examine the implementation and effectiveness of any intervention work that would be performed after these individuals were identified.

27 Channel panels are multi‐agency panels that receive and assess referrals received from different sources to determine whether they are eligible to be adopted into the UK government's programme.

28 Although, it is important to note that this study specifically focused on clinical support offered to the children of radicalised parents, or parents suspected of being radicalised, which could potentially have contributed to parents being reticent about their child's participation.

29 It is worth stating that an evidence review conducted by Cherney et al. (2021) found no evidence to suggest that mandating CVE interventions was an effective approach. However, it did identify similar issues relating to implicit or inferred coercion and obtaining consent. See Cherney, A., De Rooy, K., and Eggins, E. 2021. “Mandatory Participation in Programs to Counter Violent Extremism: A Review of Evidence for and Against.” *Journal for Deradicalization* 27: 1–33.

30 See note 28.

31 A notable weakness in the evidence base was the limited amount of empirical evidence that examined whether and how youth‐focused CVE work differs from work with older cohorts, or specific features of youth cohorts that warrant consideration.

32 Although several studies examined work delivered in the context of national strategies or policies and involved some level of national government involvement, the activities delivered often had a local focus.

33 It is worth noting that a pre‐post analysis of outcome data reported that mentees (gender unspecified) who had a female mentor were significantly more likely to ‘identify the risks of joining [violent extremist] groups’ than those who did not (Sahgal and Kimaiyo 2020), although there were no significant differences on other relevant outcomes. A supplemental report pointed to some of the opportunities and challenges relating to working across different genders. For example, ‘cross‐gender matching’ of mentors and mentees had been seen as useful in some cases as ‘males and females became more knowledgeable about the issues of those of the opposite gender’. The fact that the programme employed male and female mentors also meant that mentees could ‘contact a mentor of the same gender when they had gender‐related issues and questions which the other mentors were unable to answer’ (RUSI 2020, 7). However, some male and female participants ‘did not feel comfortable sharing in the presence of the opposite gender’ during group sessions, which led to some meetings becoming ‘gender‐segregated’ (RUSI 2020, 37).

34 Similar practices were also noted in several studies that were excluded from the review, most notably Thornton et al. (2019). The school setting was also seen as potentially helping to overcome some of the challenges around Channel being a voluntary programme based on students having an expectation that they ‘have to comply with demands made of them on school grounds’, even if the authors note that there was ‘no suggestion was made that children were forced to take part’. See Thornton, A., and Bouhana, N. 2019. “Preventing Radicalization in the UK: Expanding the Knowledge‐Base on the Channel Programme.” *Policing: A Journal of Policy and Practice* 13(3): 331–344.
