# Supplementary material for: Non‐Criminal Justice Interventions for Countering Cognitive and Behavioural Radicalisation Amongst Children and Adolescents: A Systematic Review of Effectiveness and Implementation: A Systematic Review
Source: Campbell Syst Rev. 2025 Dec 28;21(4):e70079. doi: 10.1002/cl2.70079 (PMC12745043; doi:10.1002/cl2.70079)
Supplement: Supplementary file 1 — Review_Appendices_Resubmission. [file CL2-21-e70079-s001.docx]

**Appendix I: Search Strategies**

--------------------------------------------------------------------------------

Database: APA PsycInfo <1806 to July Week 5 2024>

Search 7 August 2024

Yield: 1,934

--------------------------------------------------------------------------------

1 (neonatal birth 1 mo or infancy 2 23 mo or preschool age 2 5 yrs or school age 6 12 yrs or adolescence 13 17 yrs or young adulthood 18 29 yrs).ag. (1314203)

2 (adolesc* or boy* or child* or daughter or daughters or girl* or juvenile* or minor or minors or preadolesc* or prepubescen* or puberty or pubescen* or son or sons or teen* or "young adult*" or young* or "young people" or youth*).mp. (1545016)

3 (college* or "elementary school*" or "high school*" or "junior high" or "middle school*" or "primary school*" or pupil* or "secondary school*" or "school-age*" or "school age*" or student* or universit*).mp. (962056)

4 or/1-3 (2531362)

5 extremism/ (917)

6 exp terrorism/ (9219)

7 radical movements/ (448)

8 (extreme-left or extreme-right or "extreme violen*" or extremism or extremist or extremists).mp. (2752)

9 terroris*.mp. (12712)

10 (anarch* or bioterror* or far-left or far-right or incel or incels or indoctrinat* or islamis* or jihadi* or militant* or militia*).mp. (4249)

11 (nationalis* or neo-nazi* or radical* or salafi* or "violent ideolog*" or "white supremacis*").mp. (30454)

12 or/5-11 (46175)

13 risk management/ (6442)

14 school based intervention/ (24092)

15 interven*.mp. (549522)

16 ((alternative* or approach* or "at risk" or campaign* or communit* or dialog* or diversion* or divert*) adj10 (counter* or deradical* or de-radical* or desist* or disengag* or exit* or prevent* or recidivis* or reduc* or re-enter* or reentry or re-entry or rehab* or reintegrat* or re-integrat*)).mp. (71373)

17 ((engage* or engaging or factor* or guidance or implement* or initiative* or interact* or intervene*) adj10 (counter* or deradical* or de-radical* or desist* or disengag* or exit* or prevent* or recidivis* or reduc* or re-enter* or reentry or re-entry or rehab* or reintegrat* or re-integrat*)).mp. (88181)

18 ((method* or model* or program* or policy or policies or practice* or project* or scheme* or strateg* or treat*) adj10 (counter* or deradical* or de-radical* or desist* or disengag* or exit* or prevent* or recidivis* or reduc* or re-enter* or reentry or re-entry or rehab* or reintegrat* or re-integrat* or "risk manage*")).mp. (273365)

19 ((alternative* or counter* or mitigat* or prevent* or reduc* or rehab* or reintegrat* or re-integrat* or "risk manage*" or stop*) adj5 (extremis* or radicali* or terrori*)).mp. (1417)

20 or/13-19 (806330)

21 4 and 12 and 20 (2193)

22 animal.po. (448507)

23 exp animals/ or animal models/ or exp animal research/ or exp primates/ (398197)

24 or/22-23 (464964)

25 human.po. (4778186)

26 24 not 25 (398953)

27 ((afrik* or alban* or arab* or bulgar* or catalan or chinese or croat* or cze* or danish or dut* or estonian or fars* or finn* or french or georgian or ger* or gre* or hebr* or hindi or hungarian or ital* or japan* or kor* or latv* or lith* or malay* or nonenglish or norweg* or polish or portug* or roman* or rus* or serb* or slovak or sloven* or spa* or swed* or turk* or ukr* or urdu) not english).lg. (341890)

28 21 not (26 or 27) (2090)

29 limit 28 to yr="2000 -Current" (1934)

--------------------------------------------------------------------------------

Database: Ovid MEDLINE(R) ALL <1946 to August 06, 2024>

Search 7 August 2024

Yield: 2,977

--------------------------------------------------------------------------------

1 adolescent/ or exp child/ (3457220)

2 (adolesc* or boy* or child* or daughter or daughters or girl* or juvenile* or minor or minors or preadolesc* or prepubescen* or puberty or pubescen* or son or sons or teen* or "young adult*" or young* or "young people" or youth*).mp. (5405245)

3 (college* or "elementary school*" or "high school*" or "junior high" or "middle school*" or "primary school*" or pupil* or "secondary school*" or "school-age*" or "school age*" or student* or universit*).mp. (1061738)

4 or/1-3 (6104289)

5 exp terrorism/ (13977)

6 (extreme-left or extreme-right or "extreme violen*" or extremism or extremist or extremists).mp. (1036)

7 terroris*.tw,ot,kf,kw. (7717)

8 (anarch* or bioterror* or far-left or far-right or incel or incels or indoctrinat* or islamis* or jihadi* or militant* or militia*).tw,ot,kf,kw. (5909)

9 (nationalis* or neo-nazi* or radical* or salafi* or "violent ideolog*" or "white supremacis*").tw,ot,kf,kw. (335227)

10 or/5-9 (357024)

11 risk management/ (19754)

12 interven*.tw,ot,kf,kw. (1516979)

13 ((alternative* or approach* or "at risk" or campaign* or communit* or dialog* or diversion* or divert*) adj10 (counter* or deradical* or de-radical* or desist* or disengag* or exit* or prevent* or recidivis* or reduc* or re-enter* or reentry or re-entry or rehab* or reintegrat* or re-integrat*)).tw,kf,kw. (231602)

14 ((engage* or engaging or factor* or guidance or implement* or initiative* or interact* or intervene*) adj10 (counter* or deradical* or de-radical* or desist* or disengag* or exit* or prevent* or recidivis* or reduc* or re-enter* or reentry or re-entry or rehab* or reintegrat* or re-integrat*)).tw,kf,kw. (354981)

15 ((method* or model* or program* or policy or policies or practice* or project* or scheme* or strateg* or treat*) adj10 (counter* or deradical* or de-radical* or desist* or disengag* or exit* or prevent* or recidivis* or reduc* or re-enter* or reentry or re-entry or rehab* or reintegrat* or re-integrat* or "risk manage*")).tw,kf,kw. (1367738)

16 ((alternative* or counter* or mitigat* or prevent* or reduc* or rehab* or reintegrat* or re-integrat* or "risk manage*" or stop*) adj5 (extremis* or radicali* or terrori*)).tw,kf,kw. (646)

17 or/11-16 (3023422)

18 4 and 10 and 17 (4151)

19 exp animals/ not humans/ (5246267)

20 18 not 19 (3952)

21 limit 20 to english language and limit 10 to yr="2000 -Current" (2977)

--------------------------------------------------------------------------------

Database: JBI EBP Database <Current to July 31, 2024>

Search Date: 7 August 2024

Yield: 114

--------------------------------------------------------------------------------

1 (adolesc* or boy* or child* or daughter or daughters or girl* or juvenile* or minor or minors or preadolesc* or prepubescen* or puberty or pubescen* or son or sons or teen* or "young adult*" or young* or "young people" or youth*).mp. (3619)

2 (college* or "elementary school*" or "high school*" or "junior high" or "middle school*" or "primary school*" or pupil* or "secondary school*" or "school-age*" or "school age*" or student* or universit*).mp. (3010)

3 or/1-2 (4662)

4 (extreme-left or extreme-right or "extreme violen*" or extremism or extremist or extremists).mp. (0)

5 terroris*.mp. (16)

6 (anarch* or bioterror* or far-left or far-right or incel or incels or indoctrinat* or islamis* or jihadi* or militant* or militia*).mp. (11)

7 (nationalis* or neo-nazi* or radical* or salafi* or "violent ideolog*" or "white supremacis*").mp. (133)

8 or/4-7 (159)

9 interven*.mp. (5229)

10 ((alternative* or approach* or "at risk" or campaign* or communit* or dialog* or diversion* or divert*) adj10 (counter* or deradical* or de-radical* or desist* or disengag* or exit* or prevent* or recidivis* or reduc* or re-enter* or reentry or re-entry or rehab* or reintegrat* or re-integrat*)).mp. (1018)

11 ((engage* or engaging or factor* or guidance or implement* or initiative* or interact* or intervene*) adj10 (counter* or deradical* or de-radical* or desist* or disengag* or exit* or prevent* or recidivis* or reduc* or re-enter* or reentry or re-entry or rehab* or reintegrat* or re-integrat*)).mp. (1199)

12 ((method* or model* or program* or policy or policies or practice* or project* or scheme* or strateg* or treat*) adj10 (counter* or deradical* or de-radical* or desist* or disengag* or exit* or prevent* or recidivis* or reduc* or re-enter* or reentry or re-entry or rehab* or reintegrat* or re-integrat* or "risk manage*")).mp. (3218)

13 ((alternative* or counter* or mitigat* or prevent* or reduc* or rehab* or reintegrat* or re-integrat* or "risk manage*" or stop*) adj5 (extremis* or radicali* or terrori*)).mp. (0)

14 or/9-13 (5955)

15 3 and 8 and 14 (114)

--------------------------------------------------------------------------------

Database: Ovid APA PsycExtra <1908 to July 08, 2024>

Search Date: 9 August 2024

Yield: 137

--------------------------------------------------------------------------------

1 (neonatal birth 1 mo or infancy 2 23 mo or preschool age 2 5 yrs or school age 6 12 yrs or adolescence 13 17 yrs or young adulthood 18 29 yrs).ag. 37866

2 (adolesc* or boy* or child* or girl* or juvenile* or minor or minors or preadolesc* or prepubescen* or puberty or pubescen* or teen* or "young adult*" or young* or "young people" or youth*).mp. 65785

3 (college* or "elementary school*" or "high school*" or "junior high" or "middle school*" or "primary school*" or pupil* or "secondary school*" or "school-age*" or "school age*" or student* or universit*).mp. 67220

4 1 or 2 or 3 124188

5 extremism/ 28

6 exp terrorism/ 1532

7 radical movements/ 22

8 (extreme-left or extreme-right or "extreme violen*" or extremism or extremist or extremists).mp. 145

9 terroris*.mp. 1903

10 (anarch* or bioterror* or far-left or far-right or incel or incels or indoctrinat* or islamis* or jihadi* or militant* or militia*).mp. 279

11 (nationalis* or neo-nazi* or radicali* or radical or radical* or salafi* or "violent ideolog*" or "white supremacis*").mp. 1077

12 (radical* adj3 (belief* or group* or ideolog* or left* or movement* or organisation* or organization* or right* or theolog*)).mp. 48

13 5 or 6 or 7 or 8 or 9 or 10 or 11 or 12 3163

14 risk management/ 440

15 school based intervention/ 1763

16 interven*.mp. 20789

17 ((alternative* or approach* or "at risk" or campaign* or communit* or dialog* or divert* or diversion?) adj10 (counter* or deradical* or de-radical* or desist* or disengag* or exit* or prevent* or recidivis* or reduc* or re-enter* or reentry or re-entry or rehab* or reintegrat* or re-integrat*)).mp. 4719

18 ((engage* or engaging or factor* or guidance or implement* or initiative* or interact*) adj10 (counter* or deradical* or de-radical* or desist* or disengag* or exit* or prevent* or recidivis* or reduc* or re-enter* or reentry or re-entry or rehab* or reintegrat* or re-integrat*)).mp. 4052

19 ((method* or model* or program* or policy or policies or practice* or project* or scheme* or strateg* or treat*) adj10 (counter* or deradical* or de-radical* or desist* or disengag* or exit* or prevent* or recidivis* or reduc* or re-enter* or reentry or re-entry or rehab* or reintegrat* or re-integrat* or "risk manage*")).mp. 14200

20 ((alternative* or counter* or mitigat* or prevent* or reduc* or rehab* or reintegrat* or re-integrat* or "risk manage*" or stop*) adj5 (extremis* or radicali* or terrori*)).mp. 204

21 14 or 15 or 16 or 17 or 18 or 19 or 20 35046

22 4 and 13 and 21 155

23 animal.po. 7392

24 exp animals/ or animal models/ or exp animal research/ or exp "Primates (Nonhuman)"/ 5992

25 23 or 24 7881

26 human.po. 298973

27 25 not 26 6195

28 ((afrik* or alban* or arab* or bulgar* or catalan or chinese or croat* or cze* or danish or dut* or estonian or fars* or finn* or french or georgian or ger* or gre* or hebr* or hindi or hungarian or ital* or japan* or kor* or latv* or lith* or malay* or nonenglish or norweg* or polish or portug* or roman* or rus* or serb* or slovak or sloven* or spa* or swed* or turk* or ukr* or urdu) not english).lg. 63

29 22 not (27 or 28) 155

30 limit 29 to yr="2000 -Current" 137

--------------------------------------------------------------------------------

Database: ProQuest Applied Social Services Index & Abstracts <1987 - Present>

Search Date: 7 August 2024

Yield: 387

--------------------------------------------------------------------------------

noft((adolesc* OR boy* OR child* OR daughter* OR girl* OR juvenile* OR minor OR minors OR preadolesc* OR prepubescen* OR puberty OR pubescen* OR son OR sons OR teen* OR "young adult*" OR young* OR "young people" OR youth* OR "college-age*" OR "elementary school*" OR "high school*" OR "junior high" OR "middle school*" OR "postsecondary school*" OR "post-secondary school*" OR "primary school*" OR pupil* OR "secondary school*" OR "school-age*" OR "school age*" OR student* OR "university-age*")) AND noft((anarch* OR bioterror* OR "extreme-left" OR "extreme-right" OR "extreme violen*" OR extremism OR extremist OR extremists OR "far-left" OR "far-right" OR incel OR incels OR indoctrinat* OR islamis* OR jihadi* OR militant* OR militia* OR nationalis* OR neo-nazi* OR radical* OR salafi* OR terroris* OR "violent ideolog*" OR "white supremacis*")) AND (noft(interven*) OR noft(((alternative* OR approach* OR "at risk" OR campaign* OR communit* OR dialog* OR diversion* OR divert*) NEAR/10 (counter* OR deradical* OR de-radical* OR desist* OR disengag* OR exit* OR prevent* OR recidivis* OR reduc* OR re-enter* OR reentry OR re-entry OR rehab* OR reintegrat* OR re-integrat*))) OR noft(((engage* OR engaging OR factor* OR guidance OR implement* OR initiative* OR interact* OR interven*) NEAR/10 (counter* OR deradical* OR de-radical* OR desist* OR disengag* OR exit* OR prevent* OR recidivis* OR reduc* OR re-enter* OR reentry OR re-entry OR rehab* OR reintegrat* OR re-integrat*))) OR noft(((method* OR model* OR program* OR policy OR policies OR practice* OR project* OR scheme* OR strateg* OR treat*) NEAR/10 (counter* OR deradical* OR de-radical* OR desist* OR disengag* OR exit* OR prevent* OR recidivis* OR reduc* OR re-enter* OR reentry OR re-entry OR rehab* OR reintegrat* OR re-integrat* OR "risk manage*"))) OR noft(((alternative* OR counter* OR mitigat* OR prevent* OR reduc* OR rehab* OR reintegrat* OR re-integrat* OR "risk manage*" OR stop*) NEAR/5 (extremis* OR radical* OR terrori*)))) AND pd(20000101-20240807)

--------------------------------------------------------------------------------

Database: ProQuest Dissertations & Theses Global <1861 - Present>

Search Date: 7 August 2024

Yield: 1,517

--------------------------------------------------------------------------------

noft((adolesc* OR boy* OR child* OR daughter* OR girl* OR juvenile* OR minor OR minors OR preadolesc* OR prepubescen* OR puberty OR pubescen* OR son OR sons OR teen* OR "young adult*" OR young* OR "young people" OR youth* OR "college-age*" OR "elementary school*" OR "high school*" OR "junior high" OR "middle school*" OR "postsecondary school*" OR "post-secondary school*" OR "primary school*" OR pupil* OR "secondary school*" OR "school-age*" OR "school age*" OR student* OR "university-age*")) AND noft((anarch* OR bioterror* OR "extreme-left" OR "extreme-right" OR "extreme violen*" OR extremism OR extremist OR extremists OR "far-left" OR "far-right" OR incel OR incels OR indoctrinat* OR islamis* OR jihadi* OR militant* OR militia* OR nationalis* OR neo-nazi* OR radical* OR salafi* OR terroris* OR "violent ideolog*" OR "white supremacis*")) AND (noft(interven*) OR noft(((alternative* OR approach* OR "at risk" OR campaign* OR communit* OR dialog* OR diversion* OR divert*) NEAR/10 (counter* OR deradical* OR de-radical* OR desist* OR disengag* OR exit* OR prevent* OR recidivis* OR reduc* OR re-enter* OR reentry OR re-entry OR rehab* OR reintegrat* OR re-integrat*))) OR noft(((engage* OR engaging OR factor* OR guidance OR implement* OR initiative* OR interact* OR interven*) NEAR/10 (counter* OR deradical* OR de-radical* OR desist* OR disengag* OR exit* OR prevent* OR recidivis* OR reduc* OR re-enter* OR reentry OR re-entry OR rehab* OR reintegrat* OR re-integrat*))) OR noft(((method* OR model* OR program* OR policy OR policies OR practice* OR project* OR scheme* OR strateg* OR treat*) NEAR/10 (counter* OR deradical* OR de-radical* OR desist* OR disengag* OR exit* OR prevent* OR recidivis* OR reduc* OR re-enter* OR reentry OR re-entry OR rehab* OR reintegrat* OR re-integrat* OR "risk manage*"))) OR noft(((alternative* OR counter* OR mitigat* OR prevent* OR reduc* OR rehab* OR reintegrat* OR re-integrat* OR "risk manage*" OR stop*) NEAR/5 (extremis* OR radical* OR terrori*)))) AND pd(20000101-20240807)

--------------------------------------------------------------------------------

Database: ProQuest Education Resources Information Center (ERIC) <1907 - Present>

Search Date: 14 August 2024

Yield: 536

--------------------------------------------------------------------------------

noft((adolesc* OR boy* OR child* OR daughter* OR girl* OR juvenile* OR minor OR minors OR preadolesc* OR prepubescen* OR puberty OR pubescen* OR son OR sons OR teen* OR "young adult*" OR young* OR "young people" OR youth* OR "college-age*" OR "elementary school*" OR "high school*" OR "junior high" OR "middle school*" OR "postsecondary school*" OR "post-secondary school*" OR "primary school*" OR pupil* OR "secondary school*" OR "school-age*" OR "school age*" OR student* OR "university-age*")) AND noft((anarch* OR bioterror* OR "extreme-left" OR "extreme-right" OR "extreme violen*" OR extremism OR extremist OR extremists OR "far-left" OR "far-right" OR incel OR incels OR indoctrinat* OR islamis* OR jihadi* OR militant* OR militia* OR nationalis* OR neo-nazi* OR radical* OR salafi* OR terroris* OR "violent ideolog*" OR "white supremacis*")) AND (noft(interven*) OR noft(((alternative* OR approach* OR "at risk" OR campaign* OR communit* OR dialog* OR diversion* OR divert*) NEAR/10 (counter* OR deradical* OR de-radical* OR desist* OR disengag* OR exit* OR prevent* OR recidivis* OR reduc* OR re-enter* OR reentry OR re-entry OR rehab* OR reintegrat* OR re-integrat*))) OR noft(((engage* OR engaging OR factor* OR guidance OR implement* OR initiative* OR interact* OR interven*) NEAR/10 (counter* OR deradical* OR de-radical* OR desist* OR disengag* OR exit* OR prevent* OR recidivis* OR reduc* OR re-enter* OR reentry OR re-entry OR rehab* OR reintegrat* OR re-integrat*))) OR noft(((method* OR model* OR program* OR policy OR policies OR practice* OR project* OR scheme* OR strateg* OR treat*) NEAR/10 (counter* OR deradical* OR de-radical* OR desist* OR disengag* OR exit* OR prevent* OR recidivis* OR reduc* OR re-enter* OR reentry OR re-entry OR rehab* OR reintegrat* OR re-integrat* OR "risk manage*"))) OR noft(((alternative* OR counter* OR mitigat* OR prevent* OR reduc* OR rehab* OR reintegrat* OR re-integrat* OR "risk manage*" OR stop*) NEAR/5 (extremis* OR radical* OR terrori*)))) AND pd(20000101-20240814)

--------------------------------------------------------------------------------

Database: ProQuest International Bibliography of the Social Sciences (IBSS) <1951 - Present>

Search Date: 14 August 2024

Yield: 1,474

--------------------------------------------------------------------------------

noft((adolesc* OR boy* OR child* OR daughter* OR girl* OR juvenile* OR minor OR minors OR preadolesc* OR prepubescen* OR puberty OR pubescen* OR son OR sons OR teen* OR ("young adult" OR "young adulthood" OR "young adults") OR young* OR "young people" OR youth* OR "college-age*" OR ("elementary school" OR "elementary schoolchildren" OR "elementary schoolhome" OR "elementary schooling" OR "elementary schoolk" OR "elementary schools" OR "elementary schoolteacher") OR ("high school" OR "high schooler" OR "high schoolers" OR "high schoolin" OR "high schooling" OR "high schools" OR "high schoolthe") OR "junior high" OR ("middle school" OR "middle schooler" OR "middle schoolers" OR "middle schooling" OR "middle schools") OR ("postsecondary school" OR "postsecondary schools") OR "post-secondary school*" OR ("primary school" OR "primary schooling" OR "primary schools" OR "primary schoolteacher") OR pupil* OR ("secondary school" OR "secondary schooling" OR "secondary schools") OR "school-age*" OR ("school age" OR "school aged" OR "school ages") OR student* OR "university-age*")) AND noft((anarch* OR bioterror* OR "extreme-left" OR "extreme-right" OR ("extreme violence" OR "extreme violent") OR extremism OR extremist OR extremists OR "far-left" OR "far-right" OR incel OR incels OR indoctrinat* OR islamis* OR jihadi* OR militant* OR militia* OR nationalis* OR neo-nazi* OR radical* OR salafi* OR terroris* OR "violent ideolog*" OR ("white supremacism" OR "white supremacist" OR "white supremacists"))) AND (noft(interven*) OR noft(((alternative* OR approach* OR "at risk" OR campaign* OR communit* OR dialog* OR diversion* OR divert*) NEAR/10 (counter* OR deradical* OR de-radical* OR desist* OR disengag* OR exit* OR prevent* OR recidivis* OR reduc* OR re-enter* OR reentry OR re-entry OR rehab* OR reintegrat* OR re-integrat*))) OR noft(((engage* OR engaging OR factor* OR guidance OR implement* OR initiative* OR interact* OR interven*) NEAR/10 (counter* OR deradical* OR de-radical* OR desist* OR disengag* OR exit* OR prevent* OR recidivis* OR reduc* OR re-enter* OR reentry OR re-entry OR rehab* OR reintegrat* OR re-integrat*))) OR noft(((method* OR model* OR program* OR policy OR policies OR practice* OR project* OR scheme* OR strateg* OR treat*) NEAR/10 (counter* OR deradical* OR de-radical* OR desist* OR disengag* OR exit* OR prevent* OR recidivis* OR reduc* OR re-enter* OR reentry OR re-entry OR rehab* OR reintegrat* OR re-integrat* OR ("risk management" OR "risk manager" OR "risk managers")))) OR noft(((alternative* OR counter* OR mitigat* OR prevent* OR reduc* OR rehab* OR reintegrat* OR re-integrat* OR ("risk management" OR "risk manager" OR "risk managers") OR stop*) NEAR/5 (extremis* OR radical* OR terrori*)))) AND pd(20000101-20240814)

--------------------------------------------------------------------------------

Database: ProQuest Social Services Abstracts <1979 - Present>

Search Date: 14 August 2024

Yield: 269

--------------------------------------------------------------------------------

noft((adolesc* OR boy* OR child* OR daughter* OR girl* OR juvenile* OR minor OR minors OR preadolesc* OR prepubescen* OR puberty OR pubescen* OR son OR sons OR teen* OR "young adult*" OR young* OR "young people" OR youth* OR "college-age*" OR "elementary school*" OR "high school*" OR "junior high" OR "middle school*" OR "postsecondary school*" OR "post-secondary school*" OR "primary school*" OR pupil* OR "secondary school*" OR "school-age*" OR "school age*" OR student* OR "university-age*")) AND noft((anarch* OR bioterror* OR "extreme-left" OR "extreme-right" OR "extreme violen*" OR extremism OR extremist OR extremists OR "far-left" OR "far-right" OR incel OR incels OR indoctrinat* OR islamis* OR jihadi* OR militant* OR militia* OR nationalis* OR neo-nazi* OR radical* OR salafi* OR terroris* OR "violent ideolog*" OR "white supremacis*")) AND (noft(interven*) OR noft(((alternative* OR approach* OR "at risk" OR campaign* OR communit* OR dialog* OR diversion* OR divert*) NEAR/10 (counter* OR deradical* OR de-radical* OR desist* OR disengag* OR exit* OR prevent* OR recidivis* OR reduc* OR re-enter* OR reentry OR re-entry OR rehab* OR reintegrat* OR re-integrat*))) OR noft(((engage* OR engaging OR factor* OR guidance OR implement* OR initiative* OR interact* OR interven*) NEAR/10 (counter* OR deradical* OR de-radical* OR desist* OR disengag* OR exit* OR prevent* OR recidivis* OR reduc* OR re-enter* OR reentry OR re-entry OR rehab* OR reintegrat* OR re-integrat*))) OR noft(((method* OR model* OR program* OR policy OR policies OR practice* OR project* OR scheme* OR strateg* OR treat*) NEAR/10 (counter* OR deradical* OR de-radical* OR desist* OR disengag* OR exit* OR prevent* OR recidivis* OR reduc* OR re-enter* OR reentry OR re-entry OR rehab* OR reintegrat* OR re-integrat* OR "risk manage*"))) OR noft(((alternative* OR counter* OR mitigat* OR prevent* OR reduc* OR rehab* OR reintegrat* OR re-integrat* OR "risk manage*" OR stop*) NEAR/5 (extremis* OR radical* OR terrori*)))) AND pd(20000101-20240814)

--------------------------------------------------------------------------------

Database: ProQuest Sociological Abstracts <1952 - Present>

Search Date: 14 August 2024

Yield: 1,109

--------------------------------------------------------------------------------

noft((adolesc* OR boy* OR child* OR daughter* OR girl* OR juvenile* OR minor OR minors OR preadolesc* OR prepubescen* OR puberty OR pubescen* OR son OR sons OR teen* OR "young adult*" OR young* OR "young people" OR youth* OR "college-age*" OR "elementary school*" OR "high school*" OR "junior high" OR "middle school*" OR "postsecondary school*" OR "post-secondary school*" OR "primary school*" OR pupil* OR "secondary school*" OR "school-age*" OR "school age*" OR student* OR "university-age*")) AND noft((anarch* OR bioterror* OR "extreme-left" OR "extreme-right" OR "extreme violen*" OR extremism OR extremist OR extremists OR "far-left" OR "far-right" OR incel OR incels OR indoctrinat* OR islamis* OR jihadi* OR militant* OR militia* OR nationalis* OR neo-nazi* OR radical* OR salafi* OR terroris* OR "violent ideolog*" OR "white supremacis*")) AND (noft(interven*) OR noft(((alternative* OR approach* OR "at risk" OR campaign* OR communit* OR dialog* OR diversion* OR divert*) NEAR/10 (counter* OR deradical* OR de-radical* OR desist* OR disengag* OR exit* OR prevent* OR recidivis* OR reduc* OR re-enter* OR reentry OR re-entry OR rehab* OR reintegrat* OR re-integrat*))) OR noft(((engage* OR engaging OR factor* OR guidance OR implement* OR initiative* OR interact* OR interven*) NEAR/10 (counter* OR deradical* OR de-radical* OR desist* OR disengag* OR exit* OR prevent* OR recidivis* OR reduc* OR re-enter* OR reentry OR re-entry OR rehab* OR reintegrat* OR re-integrat*))) OR noft(((method* OR model* OR program* OR policy OR policies OR practice* OR project* OR scheme* OR strateg* OR treat*) NEAR/10 (counter* OR deradical* OR de-radical* OR desist* OR disengag* OR exit* OR prevent* OR recidivis* OR reduc* OR re-enter* OR reentry OR re-entry OR rehab* OR reintegrat* OR re-integrat* OR "risk manage*"))) OR noft(((alternative* OR counter* OR mitigat* OR prevent* OR reduc* OR rehab* OR reintegrat* OR re-integrat* OR "risk manage*" OR stop*) NEAR/5 (extremis* OR radical* OR terrori*)))) AND pd(20000101-20240814)

--------------------------------------------------------------------------------

Databases: Clarivate Web of Science (SSCI, CPCI-SSH, ESCI) 2000-01-01 to 2024-08-14

SSCI <1900 - Present> CPCI-SSH <1990 - Present> ESCI <2005 - Present>

Search Date: 14 August 2024

Yield: 2,610 (SSCI: 1,435; CPCI-SSH: 130; ESCI: 1,061)

--------------------------------------------------------------------------------

9 #1 AND #2 AND #8 2,610

8 #3 OR #4 OR #5 OR #6 OR #7 1,391,465

7 TS=interven* 890,923

6 TS=((alternative* OR approach* OR "at risk" OR campaign* OR communit* OR dialog* OR diversion* OR divert*) NEAR/8 (counter* OR deradical* OR de-radical* OR desist* OR disengag* OR exit* OR prevent* OR recidivis* OR reduc* OR re-enter* OR reentry OR re-entry OR rehab* OR reintegrat* OR re-integrat*)) 116,287

5 TS=((engage* OR engaging OR factor* OR guidance OR implement* OR initiative* OR interact*) NEAR/8 (counter* OR deradical* OR de-radical* OR desist* OR disengag* OR exit* OR prevent* OR recidivis* OR reduc* OR re-enter* OR reentry OR re-entry OR rehab* OR reintegrat* OR re-integrat*)) 142,056

4 TS=((method* OR model* OR program* OR policy OR policies OR practice* OR project* OR scheme* OR strateg* OR treat*) NEAR/8 (counter* OR deradical* OR de-radical* OR desist* OR disengag* OR exit* OR prevent* OR recidivis* OR reduc* OR re-enter* OR reentry OR re-entry OR rehab* OR reintegrat* OR re-integrat* OR "risk manage*")) 486,693

3 TS=((alternative* OR counter* OR mitigat* OR prevent* OR reduc* OR rehab* OR reintegrat* OR re-integrat* OR "risk manage*" OR stop*) NEAR/5 (extremis* OR radicali* OR terrori*)) 5,531

2 TS=(anarch* OR bioterror* OR "extreme-left" OR "extreme-right" OR "extreme violen*" OR extremism OR extremist OR extremists OR "far-left" OR "far-right" OR incel OR incels OR indoctrinat* OR islamis* OR jihadi* OR militant* OR militia* OR nationalis* OR neo-nazi* OR radical* OR salafi* OR terroris* OR "violent ideolog*" OR "white supremacis*") 196,423

1 TS=(adolesc* OR boy* OR child* OR daughter* OR girl* OR juvenile* OR minor OR minors OR preadolesc* OR prepubescen* OR puberty OR pubescen* OR teen* OR "young adult*" OR "young people" OR young* OR youth* OR "college-age*" OR "elementary school*" OR "high school*" OR "junior high" OR "middle school*" OR "primary school*" OR pupil* OR "secondary school*" OR "school-age*" OR "school age*" OR son OR sons OR student* OR "university-age*") 2,653,363

--------------------------------------------------------------------------------

Database: Clarivate Web of Science Book Citation Index (WOS.BHCI) 2000-01-01 to 2024-08-09

<2005 - Present>

Search Date: 9 August 2024

Yield: 58

--------------------------------------------------------------------------------

1 "TS=((adolesc* OR boy* OR child* OR girl* OR juvenile* OR minor OR minors OR neonat* OR preadolesc* OR prepubescen* OR puberty OR pubescen* OR teen* OR ""young adult*"" OR ""young people"" OR young* OR youth* OR ""college-age*"" OR ""elementary school*"" OR ""high school*"" OR ""junior high"" OR ""middle school*"" OR ""primary school*"" OR pupil* OR ""secondary school*"" OR ""school-age*"" OR ""school age*"" OR student* OR ""university-age*"")) 75685

2 "TS=(anarch* OR bioterror* OR ""extreme-left"" OR ""extreme-right"" OR ""extreme violen*"" OR extremism OR extremist OR extremists OR ""far-left"" OR ""far-right"" OR incel OR incels OR indoctrinat* OR islamis* OR jihadi* OR militant* OR militia* OR nationalis* OR neo-nazi* OR ""radical* belief*"" OR ""radical* group*"" OR ""radical* ideolog*"" OR ""radical* left*"" OR ""radical* movement*"" OR ""radical* organisation*"" OR ""radical* organization*"" OR ""radical* right*"" OR ""radical* theolog*"" OR radicali* OR radical* OR salafi* OR terroris* OR ""violent ideolog*"" OR ""white supremacis*"") 17621

3 "TS=((alternative* OR counter* OR mitigat* OR prevent* OR reduc* OR rehab* OR reintegrat* OR re-integrat* OR ""risk manage*"" OR stop*) NEAR/5 (extremis* OR radicali* OR terrori*)) 787

4 "TS=((method* OR model* OR program* OR policy OR policies OR practice* OR project* OR scheme* OR strateg* OR treat*) NEAR/8 (counter* OR deradical* OR de-radical* OR desist* OR disengag* OR exit* OR prevent* OR recidivis* OR reduc* OR re-enter* OR reentry OR re-entry OR rehab* OR reintegrat* OR re-integrat* OR ""risk manage*"")) 4600

5 "TS=((engage* OR engaging OR factor* OR guidance OR implement* OR initiative* OR interact*) NEAR/8 (counter* OR deradical* OR de-radical* OR desist* OR disengag* OR exit* OR prevent* OR recidivis* OR reduc* OR re-enter* OR reentry OR re-entry OR rehab* OR reintegrat* OR re-integrat*)) 1080

6 "TS=((alternative* OR approach* OR ""at risk"" OR campaign* OR communit* OR dialog* OR diversion* OR divert*) NEAR/8 (counter* OR deradical* OR de-radical* OR desist* OR disengag* OR exit* OR prevent* OR recidivis* OR reduc* OR re-enter* OR reentry OR re-entry OR rehab* OR reintegrat* OR re-integrat*)) 1490

7 "TS=interve* 11791

8 "#3 OR #4 OR #5 OR #6 OR #7 17822

9 "#8 AND #1 AND #2 58

--------------------------------------------------------------------------------

Database/Platform: EBSCO Criminal Justic Abstracts <1910 - Present>

Search Date: 15 August 2024

Yield: 696

--------------------------------------------------------------------------------

S10 S9 Limiters - Publication Date: 20000101-20240831 696

S9 S1 AND S2 AND S8 Expanders - Apply related words; Apply equivalent subjects 754

S8 S3 OR S4 OR S5 OR S6 OR S7 89,119

S7 ( TI ((alternative* OR counter* OR mitigat* OR prevent* OR reduc* OR rehab* OR reintegrat* OR re-integrat* OR "risk manage*" OR stop*) N3 (extremis* OR radical* OR terrori*)) ) OR ( AB ((alternative* OR counter* OR mitigat* OR prevent* OR reduc* OR rehab* OR reintegrat* OR re-integrat* OR "risk manage*" OR stop*) N4 (extremis* OR radical* OR terrori*)) ) OR ( DE (extremis*) ) 2,684

S6 ( TI ((method* OR model* OR program* OR policy OR policies OR practice* OR project* OR scheme* OR strateg* OR treat*) N3 (counter* OR deradical* OR de-radical* OR desist* OR disengag* OR exit* OR prevent* OR recidivis* OR reduc* OR re-enter* OR reentry OR re-entry OR rehab* OR reintegrat* OR re-integrat* OR "risk manage*")) ) OR ( AB ((method* OR model* OR program* OR policy OR policies OR practice* OR project* OR scheme* OR strateg* OR treat*) N4 (counter* OR deradical* OR de-radical* OR desist* OR disengag* OR exit* OR prevent* OR recidivis* OR reduc* OR re-enter* OR reentry OR re-entry OR rehab* OR reintegrat* OR re-integrat* OR "risk manage*")) ) OR ( DE (radical*) ) 32,168

S5 ( TI ((engage* OR engaging OR factor* OR guidance OR implement* OR initiative* OR interact* OR interven*) N3 (counter* OR deradical* OR de-radical* OR desist* OR disengag* OR exit* OR prevent* OR recidivis* OR reduc* OR re-enter* OR reentry OR re-entry OR rehab* OR reintegrat* OR re-integrat*)) ) OR ( AB ((engage* OR engaging OR factor* OR guidance OR implement* OR initiative* OR interact* OR interven*) N4 (counter* OR deradical* OR de-radical* OR desist* OR disengag* OR exit* OR prevent* OR recidivis* OR reduc* OR re-enter* OR reentry OR re-entry OR rehab* OR reintegrat* OR re-integrat*)) ) OR ( DE (reintegrat* OR re-integrat*) ) 17,172

S4 ( TI ((alternative* OR approach* OR "at risk" OR campaign* OR communit* OR dialog* OR diversion* OR divert*) N3 (counter* OR deradical* OR de-radical* OR desist* OR disengag* OR exit* OR prevent* OR recidivis* OR reduc* OR re-enter* OR reentry OR re-entry OR rehab* OR reintegrat* OR re-integrat*)) ) OR ( AB ((alternative* OR approach* OR "at risk" OR campaign* OR communit* OR dialog* OR diversion* OR divert*) N4 (counter* OR deradical* OR de-radical* OR desist* OR disengag* OR exit* OR prevent* OR recidivis* OR reduc* OR re-enter* OR reentry OR re-entry OR rehab* OR reintegrat* OR re-integrat*)) ) OR ( DE (terrorism) ) 21,632

S3 TI interven* OR AB interven* OR DE intervention* 48,735

S2 ( TI ((anarch* OR bioterror* OR "extreme-left" OR "extreme-right" OR "extreme violen*" OR extremism OR extremist OR extremists OR "far-left" OR "far-right" OR incel OR incels OR indoctrinat* OR islamis* OR jihadi* OR militant* OR militia* OR nationalis* OR neo-nazi* OR radical* OR salafi* OR terroris* OR "violent ideolog*" OR "white supremacis*")) ) OR ( AB ((anarch* OR bioterror* OR "extreme-left" OR "extreme-right" OR "extreme violen*" OR extremism OR extremist OR extremists OR "far-left" OR "far-right" OR incel OR incels OR indoctrinat* OR islamis* OR jihadi* OR militant* OR militia* OR nationalis* OR neo-nazi* OR radical* OR salafi* OR terroris* OR "violent ideolog*" OR "white supremacis*")) ) OR ( DE ((anarch* OR bioterror* OR "extreme-left" OR "extreme-right" OR "extreme violen*" OR extremism OR extremist OR extremists OR "far-left" OR "far-right" OR incel OR incels OR indoctrinat* OR islamis* OR jihadi* OR militant* OR militia* OR nationalis* OR neo-nazi* OR radical* OR salafi* OR terroris* OR "violent ideolog*" OR "white supremacis*")) ) 20,417

S1 ( TI ((adolesc* OR child* OR daughter* OR juvenile* OR preadolesc* OR prepubescen* OR son OR sons OR teen* OR "young adult*" OR "young people" OR young* OR youth* OR "college-age*" OR "elementary school*" OR "high school*" OR "junior high" OR "middle school*" OR "primary school*" OR pupil* OR "secondary school*" OR "school-age*" OR "school age*" OR student* OR "university-age*")) ) OR ( AB ((adolesc* OR child* OR daughter* OR juvenile* OR preadolesc* OR prepubescen* OR son OR sons OR teen* OR "young adult*" OR "young people" OR young* OR youth* OR "college-age*" OR "elementary school*" OR "high school*" OR "junior high" OR "middle school*" OR "primary school*" OR pupil* OR "secondary school*" OR "school-age*" OR "school age*" OR student* OR "university-age*")) ) OR ( DE (adolesc* OR child* OR "young adult*") ) 167,070

--------------------------------------------------------------------------------

Database/Platform: EBSCO Cumulative Index to Nursing and Allied Health Literature (CINAHL)

<1937 - Present>

Search Date: 15 August 2024

Yield: 1,001

--------------------------------------------------------------------------------

S13 S3 AND S6 AND S12 Limiters - Publication Date: 20000101-20240831 1,001

S12 S7 OR S8 OR S9 OR S10 OR S11 958,895

S11 ( TI ((alternative* OR counter* OR mitigat* OR prevent* OR reduc* OR rehab* OR reintegrat* OR re-integrat* OR "risk manage*" OR stop*) N8 (extremis* OR radical* OR terrori*)) ) OR ( AB ((alternative* OR counter* OR mitigat* OR prevent* OR reduc* OR rehab* OR reintegrat* OR re-integrat* OR "risk manage*" OR stop*) N8 (extremis* OR radical* OR terrori*)) ) 2,493

S10 ( TI ((method* OR model* OR program* OR policy OR policies OR practice* OR project* OR scheme* OR strateg* OR treat*) N8 (counter* OR deradical* OR de-radical* OR desist* OR disengag* OR exit* OR prevent* OR recidivis* OR reduc* OR re-enter* OR reentry OR re-entry OR rehab* OR reintegrat* OR re-integrat* OR "risk manage*")) ) OR ( AB ((method* OR model* OR program* OR policy OR policies OR practice* OR project* OR scheme* OR strateg* OR treat*) N8 (counter* OR deradical* OR de-radical* OR desist* OR disengag* OR exit* OR prevent* OR recidivis* OR reduc* OR re-enter* OR reentry OR re-entry OR rehab* OR reintegrat* OR re-integrat* OR "risk manage*")) ) 325,248

S9 ( TI ((engage* OR engaging OR factor* OR guidance OR implement* OR initiative* OR interact* OR interven*) N8 (counter* OR deradical* OR de-radical* OR desist* OR disengag* OR exit* OR prevent* OR recidivis* OR reduc* OR re-enter* OR reentry OR re-entry OR rehab* OR reintegrat* OR re-integrat*)) ) OR ( AB ((engage* OR engaging OR factor* OR guidance OR implement* OR initiative* OR interact* OR interven*) N8 (counter* OR deradical* OR de-radical* OR desist* OR disengag* OR exit* OR prevent* OR recidivis* OR reduc* OR re-enter* OR reentry OR re-entry OR rehab* OR reintegrat* OR re-integrat*)) ) 169,096

S8 ( TI ((alternative* OR approach* OR "at risk" OR campaign* OR communit* OR dialog* OR diversion* OR divert*) N8 (counter* OR deradical* OR de-radical* OR desist* OR disengag* OR exit* OR prevent* OR recidivis* OR reduc* OR re-enter* OR reentry OR re-entry OR rehab* OR reintegrat* OR re-integrat*)) ) OR ( AB ((alternative* OR approach* OR "at risk" OR campaign* OR communit* OR dialog* OR diversion* OR divert*) N8 (counter* OR deradical* OR de-radical* OR desist* OR disengag* OR exit* OR prevent* OR recidivis* OR reduc* OR re-enter* OR reentry OR re-entry OR rehab* OR reintegrat* OR re-integrat*)) )

171,532

S7 TI interven* OR AB interven* OR MH intervention* 595,968

S6 S4 OR S5 38,708

S5 ( TI ((anarch* OR bioterror* OR "extreme-left" OR "extreme-right" OR "extreme violen*" OR extremism OR extremist OR extremists OR "far-left" OR "far-right" OR incel OR incels OR indoctrinat* OR islamis* OR jihadi* OR militant* OR militia* OR nationalis* OR neo-nazi* OR radical* OR salafi* OR terroris* OR "violent ideolog*" OR "white supremacis*")) ) OR ( AB ((anarch* OR bioterror* OR "extreme-left" OR "extreme-right" OR "extreme violen*" OR extremism OR extremist OR extremists OR "far-left" OR "far-right" OR incel OR incels OR indoctrinat* OR islamis* OR jihadi* OR militant* OR militia* OR nationalis* OR neo-nazi* OR radical* OR salafi* OR terroris* OR "violent ideolog*" OR "white supremacis*")) ) 34,382

S4 (MH "Terrorism+") 7,250

S3 S1 OR S2 1,871,685

S2 ( TI ((adolesc* OR boy OR boys OR child* OR daughter* OR girl OR girls OR juvenile* OR preadolesc* OR prepubescen* OR son OR sons OR teen* OR "young adult*" OR "young people" OR young* OR youth* OR college* OR "elementary school*" OR "high school*" OR "junior high" OR "middle school*" OR "primary school*" OR pupil* OR "secondary school*" OR "school-age*" OR "school age*" OR student* OR universit*)) ) OR ( AB ((adolesc* OR boy OR boys OR child* OR daughter* OR girl OR girls OR juvenile* OR preadolesc* OR prepubescen* OR son OR sons OR teen* OR "young adult*" OR "young people" OR young* OR youth* OR college* OR "elementary school*" OR "high school*" OR "junior high" OR "middle school*" OR "primary school*" OR pupil* OR "secondary school*" OR "school-age*" OR "school age*" OR student* OR universit*)) ) 1,264,935

S1 MH ("Child+" OR "Adolescence+" OR "Young Adult") 1,256,042

--------------------------------------------------------------------------------

Database/Platform: EBSCO International Political Science Abstracts <1951 - Present>

Search Date: 15 August 2024

Yield: 39

--------------------------------------------------------------------------------

S5 S4 Limiters - Publication Date: 20000101-20240831 39

S4 S1 AND S2 AND S3 39

S3 TX (interven* OR counter* OR deradical* OR de-radical* OR desist* OR disengag* OR exit* OR prevent* OR recidivis* OR reduc* OR re-enter* OR reentry OR re-entry OR rehab* OR reintegrat* OR re-integrat*) 5,240

S2 TX (anarch* OR bioterror* OR "extreme-left" OR "extreme-right" OR "extreme violen*" OR extremism OR extremist OR extremists OR "far-left" OR "far-right" OR incel OR incels OR indoctrinat* OR islamis* OR jihadi* OR militant* OR militia* OR nationalis* OR neo-nazi* OR radical* OR salafi* OR terroris* OR "violent ideolog*" OR "white supremacis*") 3,957

S1 TX (adolesc* OR boy OR boys OR child* OR daughter* OR girl OR girls OR juvenile* OR preadolesc* OR prepubescen* OR son OR sons OR teen* OR "young adult*" OR "young people" OR young* OR youth* OR college* OR "elementary school*" OR "high school*" OR "junior high" OR "middle school*" OR "primary school*" OR pupil* OR "secondary school*" OR “school-age*” OR “school age*” OR student* OR universit*) 3,197

--------------------------------------------------------------------------------

Database: Epistemonikos <1900 - Present>

Search Date: 15 August 2024

Yield: 1,159

--------------------------------------------------------------------------------

(title:((adolesc* OR boy* OR child* OR daughter* OR girl* OR juvenil* OR preadolesc* OR prepubesc* OR pubert* OR pubescen* OR teen* OR "young adult*" OR "young people" OR young* OR youth* OR college* OR "elementary school*" OR "high school*" OR "junior high*" OR "middle school*" OR "primary school*" OR pupil* OR "secondary school*" OR "school-aged" OR "school age*" OR student* OR universit*)) OR abstract:((adolesc* OR boy* OR child* OR daughter* OR girl* OR juvenil* OR preadolesc* OR prepubesc* OR pubert* OR pubescen* OR teen* OR "young adult*" OR "young people" OR young* OR youth* OR college* OR "elementary school*" OR "high school*" OR "junior high*" OR "middle school*" OR "primary school*" OR pupil* OR "secondary school*" OR "school-aged" OR "school age*" OR student* OR universit*))) AND (title:((anarch* OR bioterror* OR "extreme-left" OR "extreme-right" OR "extreme violen*" OR extremism OR extremist OR extremists OR "far-left" OR "far-right" OR incel OR incels OR indoctrinat* OR islamis* OR jihadi* OR militant* OR militia* OR nationalis* OR neo-nazi* OR radical* OR salafi* OR terroris* OR "violent ideolog*" OR "white supremacis*")) OR abstract:((anarch* OR bioterror* OR "extreme-left" OR "extreme-right" OR "extreme violen*" OR extremism OR extremist OR extremists OR "far-left" OR "far-right" OR incel OR incels OR indoctrinat* OR islamis* OR jihadi* OR militant* OR militia* OR nationalis* OR neo-nazi* OR radical* OR salafi* OR terroris* OR "violent ideolog*" OR "white supremacis*"))) AND (title:((interven* OR counter* OR deradical* OR de-radical* OR desist* OR disengag* OR exit* OR prevent* OR recidivis* OR reduc* OR re-enter* OR reentry OR re-entry OR rehab* OR reintegrat* OR re-integrat*)) OR abstract:((interven* OR counter* OR deradical* OR de-radical* OR desist* OR disengag* OR exit* OR prevent* OR recidivis* OR reduc* OR re-enter* OR reentry OR re-entry OR rehab* OR reintegrat* OR re-integrat*)))

--------------------------------------------------------------------------------

Database: Elsevier Scopus <1788 - Present>

Search Date: 7 August 2024

Yield: 3,390

--------------------------------------------------------------------------------

((TITLE-ABS-KEY((alternative* OR approach* OR "at risk" OR campaign* OR communit* OR dialog* OR diversion* OR divert* ) W/3 (counter* OR deradical* OR de-radical* OR desist* OR disengag* OR exit* OR prevent* OR recidivis* OR reduc* OR re-enter* OR reentry OR re-entry OR rehab* OR reintegrat* OR re-integrat*)) OR TITLE-ABS-KEY((engage* OR engaging OR factor* OR guidance OR implement* OR initiative* OR interact* OR interven* ) W/3 (counter* OR deradical* OR de-radical* OR desist* OR disengag* OR exit* OR prevent* OR recidivis* OR reduc* OR re-enter* OR reentry OR re-entry OR rehab* OR reintegrat* OR re-integrat*)) OR TITLE-ABS-KEY((method* OR model* OR program* OR policy OR policies OR practice* OR project* OR scheme* OR strateg* OR treat* ) W/3 (counter* OR deradical* OR de-radical* OR desist* OR disengag* OR exit* OR prevent* OR recidivis* OR reduc* OR re-enter* OR reentry OR re-entry OR rehab* OR reintegrat* OR re-integrat* OR "risk manage*")) OR TITLE-ABS-KEY((alternative* OR counter* OR mitigat* OR prevent* OR reduc* OR rehab* OR reintegrat* OR re-integrat* OR "risk manage*" OR stop* ) W/3 (extremis* OR radical* OR terrori*)))) AND (INDEXTERMS(terrorism) OR TITLE-ABS-KEY(anarch* OR bioterror* OR "extreme-left" OR "extreme-right" OR "extreme violen*" OR extremism OR extremist OR extremists OR "far-left" OR "far-right" OR incel OR incels OR indoctrinat* OR islamis* OR jihadi* OR militant* OR militia* OR nationalis* OR "neo-nazi*" OR radical* OR salafi* OR terroris* OR "violent ideolog*" OR "white supremacis*")) AND ((INDEXTERMS(adolescent) OR INDEXTERMS(child)) OR (TITLE-ABS-KEY(adolesc* OR boy* OR child* OR daughter* OR girl* OR juvenile* OR minor OR minors OR preadolesc* OR prepubescen* OR puberty OR pubescen* OR son OR sons OR teen* OR "young adult*" OR "young people" OR young* OR youth* )) OR (TITLE-ABS-KEY("college-age*" OR "elementary school*" OR "high school*" OR "junior high" OR "middle school*" OR "primary school*" OR pupil* OR "secondary school*" OR "school-age*" OR "school age*" OR "university-age*"))) AND PUBYEAR > 1999 AND PUBYEAR < 2025

--------------------------------------------------------------------------------

Australian Criminology Database (CINCH) <1968 - Present>

Search Date: 9 August 2024

Yield: 54

--------------------------------------------------------------------------------

[All Fields and Full Text:(adolesc* OR All Fields and Full Text:babies OR All Fields and Full Text:baby OR All Fields and Full Text:boy OR All Fields and Full Text:boys OR All Fields and Full Text:child* OR All Fields and Full Text:girl OR All Fields and Full Text:girls OR All Fields and Full Text:infanc* OR All Fields and Full Text:infant* OR All Fields and Full Text:juvenil* OR All Fields and Full Text:minor OR All Fields and Full Text:minors OR All Fields and Full Text:neonatal* OR All Fields and Full Text:preadolesc* OR All Fields and Full Text:pre-adult OR All Fields and Full Text:pre-adults OR All Fields and Full Text:pre-school OR All Fields and Full Text:pre-schooler OR All Fields and Full Text:pre-schools OR All Fields and Full Text:pre-schools OR All Fields and Full Text:pre-schoolers OR All Fields and Full Text:preschool* OR All Fields and Full Text:prepubesc* OR All Fields and Full Text:preteen* OR All Fields and Full Text:pubescen* OR All Fields and Full Text:pupil OR All Fields and Full Text:pupils OR All Fields and Full Text:school-age OR All Fields and Full Text:school-aged OR All Fields and Full Text:"school age" OR All Fields and Full Text:"school aged" OR All Fields and Full Text:student* OR All Fields and Full Text:teen* OR All Fields and Full Text:toddler* OR All Fields and Full Text:underage* OR All Fields and Full Text:youngs* OR All Fields and Full Text:youth*)] AND [All Fields and Full Text:(anarch* OR All Fields and Full Text:bioterror* OR All Fields and Full Text:"extreme-left" OR All Fields and Full Text:"extreme-right" OR All Fields and Full Text:"extreme violen*" OR All Fields and Full Text:extremism OR All Fields and Full Text:extremist OR All Fields and Full Text:extremists OR All Fields and Full Text:"far-left" OR All Fields and Full Text:"far-right" OR All Fields and Full Text:incel OR All Fields and Full Text:incels OR All Fields and Full Text:indoctrinat* OR All Fields and Full Text:islamis* OR All Fields and Full Text:jihadi* OR All Fields and Full Text:militant* OR All Fields and Full Text:militia* OR All Fields and Full Text:nationalis* OR All Fields and Full Text:neo-nazi OR All Fields and Full Text:neo-nazis OR All Fields and Full Text:radical* OR All Fields and Full Text:salafi* OR All Fields and Full Text:terroris* OR All Fields and Full Text:"violent ideology" OR All Fields and Full Text:"violent ideologies" OR All Fields and Full Text:"white supremacist" OR All Fields and Full Text:"white supremacy" OR All Fields and Full Text:"white supremacists")] AND [All Fields and Full Text:(interven* OR All Fields and Full Text:counter* OR All Fields and Full Text:deradical* OR All Fields and Full Text:de-radicalize OR All Fields and Full Text:de-radicalise OR All Fields and Full Text:de-radicalized OR All Fields and Full Text:de-radicalised OR All Fields and Full Text:desist* OR All Fields and Full Text:disengag* OR All Fields and Full Text:exit* OR All Fields and Full Text:prevent* OR All Fields and Full Text:recidivis* OR All Fields and Full Text:reduc* OR All Fields and Full Text:re-enter OR All Fields and Full Text:re-enters OR All Fields and Full Text:re-entry OR All Fields and Full Text:re-entered OR All Fields and Full Text:re-entering OR All Fields and Full Text:reentry OR All Fields and Full Text:rehab* OR All Fields and Full Text:reintegrat* OR All Fields and Full Text:re-integrate OR All Fields and Full Text:re-integrated OR All Fields and Full Text:re-integration OR All Fields and Full Text:re-integrates OR All Fields and Full Text:re-integrating)]

--------------------------------------------------------------------------------

**Appendix II: Screening Tools**

Title/ Abstract

| **Topic** | **Inclusion criteria Research Question 1 (Effectiveness)** | **Inclusion criteria Research Questions 2 & 3 (Implementation)** | **Exclusion criteria** |
| --- | --- | --- | --- |
| Duplicate | N/A | | Duplicate title, secondary title, author, year to be excluded. |
| Topic | Study focuses on efforts to prevent and counter cognitive and/or behavioural forms of radicalisation | | Study does focus on efforts to counter cognitive and/or behavioural forms of radicalisation |
| Year of Publication | 2000 onwards | | Published before 2000 |
| Language | English | | Language other than English |

Full Text

| **Topic** | **Inclusion criteria Research Question 1 (Effectiveness)** | | **Inclusion criteria Research Questions 2 & 3 (Implementation)** | **Exclusion criteria** |
| --- | --- | --- | --- | --- |
| Duplicate | N/A | | | Duplicate record published in another format (e.g., journal article & chapter in PhD thesis) |
| Research design | Quantitative study using an experimental or stronger quasi-experimental research design to report on primary or secondary research data.  Eligible designs include:  • Randomised controlled trials  • Cross‐over designs  • Propensity or statistically matched control group designs (with or without baseline)  • Unmatched control group designs without baseline where control group has face validity  • Unmatched control group designs with pre-post intervention measures allowing for difference‐in‐difference analysis.  • Short interrupted time‐series designs with control group (less than 25 pre-and post-intervention observations)  • Long interrupted time-series designs with or without a control group (over 25 pre-and post-intervention observations) | | Quantitative study using an experimental, quasi-experimental or non-experimental research design to report on primary or secondary data.  OR  Qualitative study using any research design to report on primary or secondary data. | Study does not meet inclusion criteria Q1-3. |
| Population (I) | Intervention works with children and adolescents aged 0-19 (or with members of their familial or social network).  Secondary or tertiary intervention working with at-risk (secondary) or radicalised (tertiary) children and adolescents. | | | Intervention does not work with children and adolescents (or familial or social networks)  Primary intervention working with broader populations of children and adolescents |
| Population (II) | Reports on data specifically relating to working with children and adolescents aged 0-19 (i.e., reports on an intervention that only works with this age group, or presents data drawn from sub-set of clients who are aged 19 or under) | At least 50 per cent of client sample is aged 19 years or under | | Data is not specific to work with children or adolescents. |
| Intervention | Intervention operates outside of the criminal justice system:  1. Work with individuals before they formally come into contact with the criminal justice system.  2. Provided as an alternative to arrest, charge, or imprisonment to individuals who are/ have previously been subject to a criminal investigation or arrest.  3. Work with individuals who have previously been or are subject to a criminal investigation, arrest, prosecution, and/ or sentence but which operate independently from criminal justice agencies, or the criminal justice system. | | | Intervention delivered in and through the criminal justice system (e.g., in correctional or probation contexts) |
| Comparator | No restrictions | | | N/A |
| Problem | Interventions explicitly focus on countering cognitive and/or behavioural radicalisation. | | | Not explicitly focused on tackling cognitive or behavioural radicalisation |
| Outcomes | Study reports on primary or secondary outcomes relevant to countering cognitive or behavioural radicalisation.  *Primary outcomes*  Attitudes (e.g., reduction in support or sympathy for a violent cause).  Intentions (e.g., reducing an individual's willingness to engage in violent action).  Behaviours (e.g., disengagement or desistance from violent extremism).  *Secondary outcomes*  Dynamic risk factors linked to cognitive or behavioural radicalisation (e.g., socio-demographic; attitudinal; psychological/ personality; experiential'; criminogenic; personal; push; pull) | | Study examines how an intervention is being implemented.  And/or  Study reports on implementation factors (facilitators, barriers) and moderators relevant to implementation. | No discussion of either element. |

**Appendix III. Quality Assessment Tools**

|  | | | | |
| --- | --- | --- | --- | --- |
| **Risk of Bias: Non-Randomised Studies: ROBINS-I^^[[1]](#footnote-1)^^** | | | | |
| **ROBINS-I (I)**  **Bias due to Confounding** | | 1.1. Is there potential for confounding of the effect of intervention in this study? | Yes  Probably Yes  Probably No  No  *If No/ Probably No, risk of bias is low for this domain and no further signalling questions are required..* | |
|  |  | *Only if Yes/ Probably Yes at 1.1.*  1.2. Was the analysis based on splitting participants’ follow up time according to intervention received? | N/A  Yes  Probably Yes  Probably No  No  No Information | |
|  |  | *Only if Yes/ Probably Yes at 1.2*  1.3. Were intervention discontinuations or switches likely to be related to factors that are prognostic for the outcome? | N/A  Yes  Probably Yes  Probably No  No  No Information | |
|  |  | *Only if Yes/ Probably Yes at 1.1.*  1.4. Did the authors use an appropriate analysis method that controlled for all the important confounding areas? | N/A  Yes  Probably Yes  Probably No  No  No Information | |
|  |  | *Only if Yes/ Probably Yes at 1.4.*  1.5. Were confounding areas that were controlled for measured validly and reliably by the variables available in this study? | N/A  Yes  Probably Yes  Probably No  No  No Information | |
|  |  | *Only if Yes/ Probably Yes at 1.1.*  1.6. Did the authors control for any post-intervention variables? | N/A  Yes  Probably Yes  Probably No  No  No Information | |
|  |  | *Only if Yes/ Probably Yes at 1.3*  1.7. Did the authors use an appropriate analysis method that adjusted for all the important confounding areas and for time varying confounding? | N/A  Yes  Probably Yes  Probably No  No  No Information | |
|  |  | *If Yes/ Probably Yes at 1.7*  1.8. Were confounding areas that were adjusted for measured validly and reliably by the variables available in this study? | N/A  Yes  Probably Yes  Probably No  No  No Information | |
|  |  | Risk of Bias for Domain | Low  Moderate  Serious  Critical  No Information | |
| **ROBINS-I (II)**  **2. Bias in selection of participants into the study** | | 2.1. Was selection of participants into the study (or into the analysis) based on participant characteristics observed after the start of intervention? | Yes  Probably Yes  Probably No (Go to 2.4)  No (Go to 2.4)  No Information | |
|  |  | *Only if Yes/ Probably Yes at 2.1*  2.2. Were the post-intervention variables that influenced selection likely to be associated with intervention? | N/A  Yes  Probably Yes  Probably No  No  No Information | |
|  |  | *If Yes/ Probably Yes at 2.2*  2.3. Were the post-intervention variables that influenced selection likely to be influenced by the outcome or a cause of the outcome? | N/A  Yes  Probably Yes  Probably No  No  No Information | |
|  |  | 2.4. Do start of follow-up and start of intervention coincide for most participants? | Yes  Probably Yes  Probably No  No  No Information | |
|  |  | *If Yes /Probably Yes to 2.2 & 2.3, or*  *If No/ Probably No to 2.4*  2.5. Were adjustment techniques used that are likely to correct for the presence of selection biases? | N/A  Yes  Probably Yes  Probably No  No  No Information | |
|  |  | Risk of Bias Judgement for Domain | Low  Moderate  Serious  Critical  No Information | |
| **ROBINS-I (III)**  **3. Bias in classification of interventions** | | 3.1. Were intervention groups clearly defined? | Yes  Probably Yes  Probably No  No  No Information | |
|  |  | 3.2. Was the information used to define intervention groups recorded at the start of the intervention? | Yes  Probably Yes  Probably No  No  No Information | |
|  |  | 3.3. Could classification of intervention status have been affected by knowledge of the outcome or risk of the outcome? | Yes  Probably Yes  Probably No  No  No Information | |
|  |  | Risk of Bias for Domain | Low  Moderate  Serious  Critical  No Information | |
| **ROBINS-I (IV)**  **4. Bias as a result of departures from intended interventions** | | 4.1. Were there deviations from the intended intervention beyond what would be expected in usual practice? | Yes  Probably Yes  Probably No  No  No Information | |
|  |  | *If Yes/ Probably Yes to 4.1.*  4.2. Were these deviations from intended intervention unbalanced between groups and likely to have affected the outcome? | N/A  Yes  Probably Yes  Probably No  No  No Information | |
|  |  | 4.3. Were important cointerventions balanced across intervention groups? | Yes  Probably Yes  Probably No  No  No Information | |
|  |  | 4.4. Was the intervention implemented successfully for most participants? | Yes  Probably Yes  Probably No  No  No Information | |
|  |  | 4.5. Did study participants adhere to the assigned intervention regimen? | Yes  Probably Yes  Probably No  No  No Information | |
|  |  | *If No/ Probably No to 4.3, 4.4 or 4.5.*  4.6. Was an appropriate analysis used to estimate the effect of starting and adhering to the intervention? | N/A  Yes  Probably Yes  Probably No  No  No Information | |
|  |  | Risk of Bias Judgement for Domain | Low  Moderate  Serious  Critical  No Information | |
| **ROBINS-I (V)**  **5. Bias as a result of missing data** | | 5.1. Were outcome data available for all, or nearly all, participants? | Yes  Probably Yes  Probably No  No  No Information | |
|  |  | 5.2. Were participants excluded due to missing data on intervention status? | Yes  Probably Yes  Probably No  No  No Information | |
|  |  | 5.3. Were participants excluded due to missing data on other variables needed for the analysis? | Yes  Probably Yes  Probably No  No  No Information | |
|  |  | *If No/ Probably No to 5.1 or*  *If Yes/ Probably Yes to 5.2 or 5.3*  5.4. Are the proportion of participants and reasons for missing data similar across interventions? | N/A  Yes  Probably Yes  Probably No  No  No Information | |
|  |  | *If No/ Probably No to 5.1 or*  *If Yes/ Probably Yes to 5.2 or 5.3*  5.5. Is there evidence that results were robust to the presence of missing data? | N/A  Yes  Probably Yes  Probably No  No  No Information | |
|  |  | Risk of Bias Judgement for Domain | Low  Moderate  Serious  Critical  No Information | |
| **ROBINS-I (VI)**  **6. Bias in measurement of outcomes** | | 6.1. Could the outcome measure have been influenced by knowledge of the intervention received? | Yes  Probably Yes  Probably No  No  No Information | |
|  |  | 6.2. Were outcome assessors aware of the intervention received by study participants? | Yes  Probably Yes  Probably No  No  No Information | |
|  |  | 6.3. Were the methods of outcome assessment comparable across intervention groups? | Yes  Probably Yes  Probably No  No  No Information | |
|  |  | 6.4. Were any systematic errors in measurement of the outcome related to intervention received? | Yes  Probably Yes  Probably No  No  No Information | |
|  |  | Risk of Bias Judgement for Domain | Low  Moderate  Serious  Critical  No Information | |
| **ROBINS-I (VII)**  **7. Bias in selection of the reported result** | | 7.1. Is the reported effect estimate likely to be selected, on the basis of the results, from multiple outcome measurements within the outcome domain? | Yes  Probably Yes  Probably No  No  No Information | |
|  |  | 7.2. Is the reported effect estimate likely to be selected, on the basis of the results, from multiple analyses of the intervention–outcome relationship? | Yes  Probably Yes  Probably No  No  No Information | |
|  |  | 7.3. Is the reported effect estimate likely to be selected, on the basis of the results, from different subgroups? | Yes  Probably Yes  Probably No  No  No Information | |
|  |  | Risk of Bias Judgement for Domain | Low  Moderate  Serious  Critical  No Information | |
| **Overall Risk of Bias** | | Overall risk of bias | Low  Moderate  Serious  Critical | |
| **Risk of Bias: Randomised Studies: RoB 2^^[[2]](#footnote-2)^^** | | | | |
| **ROB 2 (I)**  **Bias arising from the randomisation process** | 1.1 Was the allocation sequence random? | | | Yes  Probably Yes  Probably No  No  No Information |
|  | 1.2 Was the allocation sequence concealed until participants were enrolled and assigned to interventions? | | | Yes  Probably Yes  Probably No  No  No Information |
|  | 1.3 Did baseline differences between intervention groups suggest a problem with the randomisation process? | | | Yes  Probably Yes  Probably No  No  No Information |
|  | Risk of Bias for Domain | | | High  Low  Some Concerns |
| **ROB 2 (II)**  **Bias due to deviations from intended interventions** | 2.1 Were participants aware of their assigned intervention during the trial? | | | Yes  Probably Yes  Probably No  No  No Information |
|  | 2.2 Were carers and people delivering the interventions aware of participants’ assigned intervention during the trial? | | | Yes  Probably Yes  Probably No  No  No Information |
|  | If Yes/ Probably Yes /No Information to 2.1 or 2.2  2.3. Were there deviations from the intended intervention that arose because of the trial context? | | | N/A  Yes  Probably Yes  Probably No  No  No Information |
|  | If Yes/ Probably Yes to 2.3  2.4. Were these deviations likely to have affected the outcome? | | | N/A  Yes  Probably Yes  Probably No  No  No Information |
|  | If Yes/ Probably Yes/ No Information to 2.4.  2.5. Were these deviations from intended intervention balanced between groups? | | | N/A  Yes  Probably Yes  Probably No  No  No Information |
|  | 2.6 Was an appropriate analysis used to estimate the effect of assignment to intervention? | | | Yes  Probably Yes  Probably No  No  No Information |
|  | If No /Probably No/ No Information to 2.6.  2.7 Was there potential for a substantial impact (on the result) of the failure to analyse participants in the group to which they were randomised? | | | N/A  Yes  Probably Yes  Probably No  No  No Information |
|  | Risk of Bias for Domain | | | High  Low  Some Concerns |
| **RoB 2 (III)**  **Bias due to missing outcome data** | 3.1 Were data for this outcome available for all, or nearly all, participants randomised? | | | Yes  Probably Yes  Probably No  No  No Information |
|  | If No /Probably No /No Information to 3.1.  3.2 Is there evidence that the result was not biased by missing outcome data? | | | N/A  Yes  Probably Yes  Probably No  No |
|  | If No /Probably No to 3.2.  3.3 Could missingness in the outcome depend on its true value? | | | N/A  Yes  Probably Yes  Probably No  No  No Information |
|  | If Yes/ Probably Yes /No Information to 3.3.  3.4 Is it likely that missingness in the outcome depended on its true value? | | | N/A  Yes  Probably Yes  Probably No  No  No Information |
|  | Risk of Bias for Domain | | | High  Low  Some Concerns |
| **RoB 2 (IV)**  **Bias in measurement of outcome** | 4.1 Was the method of measuring the outcome inappropriate? | | | Yes  Probably Yes  Probably No  No  No Information |
|  | 4.2 Could measurement or ascertainment of the outcome have differed between intervention groups? | | | Yes  Probably Yes  Probably No  No  No Information |
|  | If No /Probably No / No Information to 4.1 & 4.2.  4.3 Were outcome assessors aware of the intervention received by study participants? | | | Yes  Probably Yes  Probably No  No  No Information |
|  | If Yes /Probably Yes/ No Information to 4.3.  4.4 Could assessment of the outcome have been influenced by knowledge of intervention received? | | | N/A  Yes  Probably Yes  Probably No  No  No Information |
|  | If Yes /Probably Yes/ No Information to 4.4.  4.5 Is it likely that assessment of the outcome was influenced by knowledge of intervention received? | | | N/A  Yes  Probably Yes  Probably No  No  No Information |
|  | Risk of Bias for Domain | | | High  Low  Some Concerns |
| **RoB 2 (V)**  **Bias in selection of the reported result** | 5.1 Were the data that produced this result analysed in accordance with a prespecified analysis plan that was finalised before unblinded outcome data were available for analysis? | | | Yes  Probably Yes  Probably No  No  No Information |
|  | 5.2 . Is the numerical result being assessed likely to have been selected, on the basis of the results, from: multiple eligible outcome measurements (eg, scales, definitions, time points) within the outcome domain? | | | Yes  Probably Yes  Probably No  No  No Information |
|  | 5.3 . Is the numerical result being assessed likely to have been selected, on the basis of the results, from: multiple eligible analyses of the data? | | | Yes  Probably Yes  Probably No  No  No Information |
|  | Risk of Bias Judgement for Domain | | | Low  Moderate  Serious  Critical  No Information |
| **Overall Risk of Bias** | Risk of Bias | | | High  Low  Some Concerns |

| **Risk of Bias: EPHPP Quality Assessment Tool^^[[3]](#footnote-3)^^** | | |
| --- | --- | --- |
| **A Selection Bias** | Are the individuals selected to participate in the study likely to be representative of the target population? | 1 Very likely  2 Somewhat likely  3 Not likely  4 Can’t tell |
|  | What percentage of selected individuals agreed to participate? | 1 80 - 100%  2 60 – 79%  3 less than 60%  4 Not applicable  5 Can’t tell |
|  | Overall rating for section | 1 Strong  2 Moderate  3 Weak |
| **B Study Design** | Indicate the study design | 1 Randomized controlled trial  2 Controlled clinical trial  3 Cohort analytic (two group pre + post)  4 Case-control  5 Cohort (one group pre + post (before & after))  6 Interrupted time series  7 Other (specify) |
|  | Was the study described as randomized? | 1 No (Go to component C)  2 Yes |
|  | If Yes  Was the method of randomization described? | 1 No  2 Yes |
|  | If Yes  Was the method appropriate? | 1 No  2 Yes |
|  | Overall rating for section | 1 Strong  2 Moderate  3 Weak |
| **C CONFOUNDERS** | Were there important differences between groups prior to the intervention?  Examples of confounders:  1 Race; 2 Sex; 3 Marital status/family; 4 Age;  5 SES (income or class); 6 Education; 7 Health status; 8 Pre-intervention score on outcome measure. | 1 Yes  2 No  3 Can’t tell |
|  | If Yes.  Indicate the percentage of relevant confounders that were controlled (either in the design (e.g. stratification, matching) or analysis)? | 1 80 – 100% (most)  2 60 – 79% (some)  3 Less than 60% (few or none)  4 Can’t Tell |
|  | Overall rating for section | 1 Strong  2 Moderate  3 Weak |
| **D BLINDING** | Was (were) the outcome assessor(s) aware of the intervention or exposure status of participants? | 1 Yes  2 No  3 Can’t tell |
|  | Were the study participants aware of the research question? | 1 Yes  2 No  3 Can’t tell |
|  | Overall rating for section | 1 Strong  2 Moderate  3 Weak |
| **E DATA COLLECTION METHODS** | Were data collection tools shown to be valid? | 1 Yes  2 No  3 Can’t tell |
|  | Were data collection tools shown to be reliable? | 1 Yes  2 No  3 Can’t tell |
|  | Overall rating for section | 1 Strong  2 Moderate  3 Weak |
| **F WITHDRAWALS AND DROP- OUTS** | Were withdrawals and drop-outs reported in terms of numbers and/or reasons per group? | 1 Yes  2 No  3 Can’t tell  4 Not Applicable (i.e. one time surveys or interviews) |
|  | Indicate the percentage of participants completing the study. (If the percentage differs by groups, record the lowest). | 1 80 -100%  2 60-79%  3 less than 60%  4 Can’t tell  5 Not Applicable (i.e. Retrospective case-control) |
|  | Overall rating for section | 1 Strong  2 Moderate  3 Weak  Not applicable |
| **G INTERVENTION INTEGRITY** | What percentage of participants received the allocated intervention or exposure of interest | 1 80 -100%  2 60-79%  3 less than 60%  4 Can’t tell |
|  | Was the consistency of the intervention measured? | 1 Yes  2 No  3 Can’t tell |
|  | Is it likely that the subjects received an unintended intervention (contamination or co-intervention) that may the results? | 1 Yes  2 No  3 Can’t tell |
| **H ANALYSES** | Indicate the unit of allocation (single code) | Community  Organization/institution  Practice/office  Individual |
|  | Indicate the unit of analysis (single code) | Community  Organization/institution  Practice/office  Individual |
|  | Are the statistical methods appropriate for the study design? | 1 Yes  2 No  3 Can’t tell |
|  | Is the analysis performed by intervention allocation status (i.e. intention to treat) rather than the actual intervention received? | 1 Yes  2 No  3 Can’t tell |
| **GLOBAL RATING** | Overall quality | 1 STRONG (No WEAK ratings)  2 MODERATE (One WEAK rating)  3 WEAK (Two or more WEAK ratings) |

| **Assessment of Qualitative Research CASP Checklist^^[[4]](#footnote-4)^^** | | |
| --- | --- | --- |
| Question | Answer Options | Consider |
| Was there a clear statement of the aims of the research? | Yes  No  Can’t Tell | - What was the goal of the research; - Why it was thought important; - Its relevance. |
| Is a qualitative methodology appropriate? | Yes  No  Can’t Tell | - If the research seeks to interpret or illuminate the actions and/or subjective experiences of research participants; - Is qualitative research the right methodology for addressing the research goal. |
| Was the research design appropriate to address the aims of the research? | Yes  No  Can’t Tell | - If the researcher has justified the research design (e.g. have they discussed how they decided which method to use) |
| Was the recruitment strategy appropriate to the aims of the research? | Yes  No  Can’t Tell | - If the researcher has explained how the participants were selected; - If they explained why the participants they selected were the most appropriate to provide access to the type of knowledge sought by the study; - If there are any discussions around recruitment (e.g. why some people chose not to take part) |
| Was the data collected in a way that addressed the research issue? | Yes  No  Can’t Tell | - If the setting for the data collection was justified; - If it is clear how data were collected (e.g. focus group, semi-structured interview etc.); - If the researcher has justified the methods chosen; - If the researcher has made the methods explicit (e.g. for interview method, is there an indication of how interviews are conducted, or did they use a topic guide); - If methods were modified during the study. If so, has the researcher explained how and why; - If the form of data is clear (e.g. tape recordings, video material, notes etc.); - If the researcher has discussed saturation of data |
| Has the relationship between researcher and participants been adequately considered? | Yes  No  Can’t Tell | - If the researcher critically examined their own role, potential bias and influence during (a) formulation of the research questions (b) data collection, including sample recruitment and choice of location; - How the researcher responded to events during the study and whether they considered the implications of any changes in the research design. |
| Have ethical issues been taken into consideration? | Yes  No  Can’t Tell | - If there are sufficient details of how the research was explained to participants for the reader to assess whether ethical standards were maintained; - If the researcher has discussed issues raised by the study (e.g. issues around informed consent or confidentiality or how they have handled the effects of the study on the participants during and after the study); - If approval has been sought from the ethics committee. |
| Was the data analysis sufficiently rigorous? | Yes  No  Can’t Tell | - If there is an in-depth description of the analysis process; - If thematic analysis is used. If so, is it clear how the categories/themes were derived from the data; - Whether the researcher explains how the data presented were selected from the original sample to demonstrate the analysis process; - If sufficient data are presented to support the findings; - To what extent contradictory data are taken into account; - Whether the researcher critically examined their own role, potential bias and influence during analysis and selection of data for presentation. |
| Is there a clear statement of findings? | Yes  No  Can’t Tell | - If the findings are explicit; - If there is adequate discussion of the evidence both for and against the researcher’s arguments; - If the researcher has discussed the credibility of their findings (e.g. triangulation, respondent validation, more than one analyst); - If the findings are discussed in relation to the original research question |
| How valuable is the research? | Free text. | - If the researcher discusses the contribution the study makes to existing knowledge. - If they identify new areas where research is necessary - If the researchers have discussed whether or how the findings can be transferred to other populations or considered other ways the research may be used.   Note: For review purposes, we converted free text for this question into a yes/ no answer to the question of whether the research was valuable based on this criteria. |

**Appendix IV. Data Extraction Tool**

| **Category** | **Field** | | **Type** |
| --- | --- | --- | --- |
| **Record**  **Information** | Author | | Free text |
|  | Year | | Free text |
|  | Title | | Free text |
|  | Bibliographic information | | Free text |
|  | Record type | | Pre-defined codes (single code)   - Journal article - Book - Book chapter - Research institution report - Government/ public agency report - Dissertation/ thesis - Other (specify) |
|  | Publication status | | Pre-defined codes (single code)   - Published - Unpublished |
|  | Overview of study | | Free text |
|  | Funding source | | Free text |
|  | Any declaration of interest | | Free text |
| **Research Design** | Type of research | | Pre-defined codes (single code)   - Quantitative - Qualitative - Mixed methods |
|  | Quantitative design  (if relevant) | | Pre-defined codes (single code)   - Experimental (specify) - Strong quasi-experimental (specify) - Weaker quasi experimental (specify) - Other (specify) |
|  | Comparator condition  (if relevant) | | Pre-defined codes (single code)   - Treatment as usual - Alternative treatment (specify) - No treatment - Waitlist |
|  | Type of comparison  (if relevant) | | Specify (e.g., empty vs. active; comparison between two interventions; comparison between versions of same intervention, etc.) |
|  | Randomisation  (if relevant) | | Pre-defined codes (single code)   - Randomised - Quasi-randomised - Non-randomised - Other (specify) |
|  | Qualitative design  (if relevant) | | Free text |
| **Data Collection** | Quantitative data  (if relevant) | | Free text - specify:  - Primary or secondary data  - Type/ form of data  - Data collection tools |
|  | Qualitative data  (if relevant) | | Free text - specify  - Primary or secondary data  - Type/ form of data  - Data collection tools |
|  | Data source/ sample | | Pre-defined codes (multi-code if needed)   - Practitioners (specify) - Other stakeholders (specify) - Children and/or adolescents - Family/ social networks (specify) - Other (specify) |
|  | Sample size | | Free text - specify sample size for each data source and data type listed above. |
| **Data Analysis** | Sample description | | Free text - sample as described in study |
|  | Approach to data analysis | | Free text - analysis as described in study |
|  | Treatment of data relating to children and adolescents. | | Pre-defined codes (single code)  - All data relates to work with 0-19s  - Subgroup analysis of work with 0-19s  - Data relating to youth broadly defined |
|  | Research question eligibility | | Pre-defined codes (multi-code if needed)   - Q1. Effectiveness - Q2. Implemented as intended - Q3. Implementation factors /moderators |
| **Intervention Details^^[[5]](#footnote-5)^^** | Intervention name | | Free text |
|  | Intervention description | | Free text |
|  | Country of delivery | | Free text |
|  | Age of clients (note, specify age of index client in event that programme works with families or peers only i.e. the person who is at risk or radicalised) | | Pre-defined codes (multi-code if needed)  - 0-9 years old  - 10-14 years old  - 15-19 years old  - 20-24 years old  - Over 25 years old |
|  | Other relevant client demographic information (e.g., types of ideology, etc.) | | Free text |
|  | Prevention | | Pre-defined codes (multi-code if needed)   - Secondary prevention   - Tertiary prevention |
|  | Contact with CJS | | Pre-defined codes (single code)  -No contact with CJS (i.e. pre-crime)  - Diversion from CJS  - Pre-arrest (specify)  - Pre-prosecution (specify)  - Pre-sentencing (specify) |
|  | Direct or Indirect Prevention | | Pre-defined codes (single code))  - Works directly with youth  - Works indirectly through families/ peers  - Combines direct and indirect work |
|  | Intervention length | | Free text |
| **Intervention Context** | Context | | Free text - brief description of delivery context (e.g., community, clinical, educational etc.) and any implementation factors and moderators as defined above. |
|  | Delivery agents | | Free text - description of delivery agents |
|  | Criminal justice agencies | | Pre-defined codes (single code)  - Criminal justice agencies involved (specify)  - Criminal justice agencies not involved. |
| **Data Analysis - Research Question 1 (Effectiveness)** | | | |
| **Progress and Outcome Measures**  **(Study Level)** | Is effectiveness assessed using eligible research design? | | Pre-defined codes (single code)  -Yes  -No |
|  | How is effectiveness assessed? | | Free text |
|  | Primary outcomes | | List of all primary outcomes in study. |
|  | Secondary outcomes | | List of all secondary outcomes in study. |
|  | Author's overall conclusions on intervention effectiveness | | Free text - summary of conclusions. |
|  | Coder’s overall assessment of intervention effectiveness | | Pre-defined codes   - Effective (specify) - Promising (specify) - Mixed results (specify) - Ineffective (specify)   - Unclear (specify) |
| **Outcome Data^^[[6]](#footnote-6)^^**  **(Separate Row for Each Primary/ Secondary outcome)** | Study Details | | Full reference for study |
|  | Outcome | | Free text - measure and description. |
|  | Outcome type | | Pre-defined codes (single code)   - Primary outcome - Secondary outcome |
|  | Primary outcome type  (if relevant) | | Pre-defined codes (single code)  - Attitudes  - Intentions  - Behaviours |
|  | Secondary outcome type  (if relevant) | | Pre-defined codes (single code)  - Socio-demographic/ background  - Attitudinal  - Psychological/ Personality  - Experiential  - Criminogenic |
|  | Measurement tool | | Free text – name/ type of tool used to capture primary/ secondary outcome. |
|  | Source of data used in original analysis. | | Pre-defined codes   - Self-report - Observation - Official source (specify) - Interview - Other (specify) |
|  | Psychometric properties of the measurement tool (e.g., reliability, validity, etc.)? | | Free text – description of properties. |
|  | Type of respondent/ data source. | | Free text – description of the type of respondent from which data was collected (e.g. client, practitioner etc.) |
|  | Time-points at which measurement taken. | | Free text - time-points at which measure collected (e.g. pre/ post intervention) |
|  | Short or delayed effect | | Immediate measurement of outcome  Delayed measurement of outcome |
|  | Was data collected in same way for treatment and comparison condition? | | Pre-defined codes (single code)   - Yes - No (specify) - Unclear |
|  | Are there any raw differences (i.e. significant or non-significant) differences between control and treatment group? | | Pre-defined codes (single code)   - Yes – results favour treatment - Yes – results favour comparison - No - Unclear |
|  | Direction of outcome change | | Pre-defined codes (single code)   - Positive - Negative - Mixed (specify) - Unclear |
|  | Statistically significant differences for outcome | | Pre-defined codes (single code)   - Yes - No - Not tested - Unclear |
|  | Study author(s)’ conclusions | | Free text – outline conclusions drawn in original study about this outcome. |
|  | Coder’s conclusions | | Free text |
| **Effect Size^^[[7]](#footnote-7)^^**  **(Separate Row for Each Effect Size)** | Page number | | Free text – page number on which effect size reported. |
|  | Type of effect captured | | Pre-defined codes (single code)   - Post-intervention only - Pre-intervention & post-intervention   - Follow-up after initial post-intervention measurement. |
|  | Timeframe captured | | Free text for all options.   - Minimum time - Maximum time - Mean time - Fixed (single code) |
|  | How effect size is captured | | Pre-defined codes (single code)   - Reported in document - Calculated by research team |
|  | *If reported in document*  Effect size | | Free text – reported effect size. |
|  | *If calculated by researchers*  Data and calculations used to calculate effect size | | Free text – data used, calculations and final effect size calculated. |
| **Data Analysis - Research Question 2 & 3 (Implementation)** | | | |
| **Q2 – Process of Implementation** | | Is the process of implementation assessed? | Pre-defined codes (single code)  -Yes  -No |
|  |  | How is process of implementation assessed? | Free text |
|  |  | Source of data used in original analysis. | Pre-defined codes   - Self-report - Observation - Official source (specify) - Interview - Other (specify) |
|  |  | Type of respondent/ data source. | Free text – description of the type of respondent from which data was collected (e.g. client, practitioner etc.) |
|  |  | Implemented in way expected? | Free text – description of extent to which implemented in way expected |
|  |  | Evidence | Free text – positive research findings (page number) |
|  |  | Evidence opposed to specific intervention, tool, or approach | Free text – negative research findings (page number) |
|  |  | Study author(s)’ conclusions | Free text – outline conclusions drawn in original study about process. |
|  |  | Coder’s conclusions | Free text –coder conclusions about process of implementation. |
| **Q3 – Implementation Factors/ Moderators** | | Implementation factors/ moderators. | Free text – describe factors/ moderators discussed. |
|  |  | Source of data used in original analysis. | Pre-defined codes   - Self-report - Observation - Official source - Interview   - Other (specify) |
|  |  | Type of respondent. | Free text – description of the type of respondent from which data was collected (e.g. client, practitioner etc.) |
|  |  | Evidence of implementation factors/ moderators having a positive effect on process.  (Separate row for each factor or moderator) | Free text – positive findings |
|  |  | Evidence of implementation factors/ moderators having a negative effect on process.  (Separate row for each factor or moderator) | Free text – negative findings |
|  |  | Study author(s)’ conclusions | Free text – outline conclusions drawn in original study about factor(s) |
|  |  | Coder’s conclusions | Free text –coder conclusions |

**Appendix V: Studies Excluded Using Refined Criteria**

| **Study** | **Exclusion Criteria** |
| --- | --- |
| Algristian, H., Choiriya, D. D., Abdillah, D. S., Ulya, A., Sodali, H. A., & Muhammad, A. R. (2019). Why Does de-Radicalization Seem a Utopia? Evaluation on "Chlidren of The Country" Program. Journal of Public Health in Africa, 10(S1), 148-151. | Lack of clear boundary between primary and secondary prevention |
| Axiom Monitoring and Evaluation (2015). Final Report SEED II: Exploring the Agricultural Initiatives Influence on Stability in Somalia. | Lack of clear boundary between primary and secondary prevention |
| Azam, Z., & Fatima, S. B. (2017). Mishal: a case study of a deradicalization and emancipation program in Swat Valley, Pakistan. Journal for Deradicalization, (11), 1-29. | Intervention was not considered to operate outside of the criminal justice system based on context. |
| Basse, Y. O. (2018). Final Evaluation Kallewa Manio: An Integrated Approach to Counter Violent Extremism in Diffa. Search for Common Ground | Lack of clear boundary between primary and secondary prevention |
| Boyle, P., Bouasla, E., & Abderebbi, M. (2016). Mid-Term Evaluation Favorable Opportunities to Reinforce Self-Advancement for Today's Youth. USAID. | Lack of clear boundary between primary and secondary prevention |
| Cockayne, J., O’Neil, S., Felbab-Brown, V., Chowdhury Fink, N., & Oswald, B. (2015). UN DDR in an Era of Violent Extremism: Is It Fit for Purpose? United Nations University. | Intervention was not considered to operate outside of the criminal justice system based on context. |
| Cook, J. (2023). Distinguishing Children From ISIS-Affiliated Families in Iraq and Their Unique Barriers for Rehabilitation and Reintegration. Perspectives on Terrorism, 17(3), 42-69. | Intervention was not considered to operate outside of the criminal justice system based on context. |
| EFCA (2018). The Contribution of Positive Youth Development in Tajikistan to Effective Peacebuilding and to Countering or Preventing Violent Extremism: Successes, Limitations, and Recommendations. | Lack of clear boundary between primary and secondary prevention |
| Finkel, S. E., Belasco, C. A., Gineste, C., Neureiter, M., & McCauley, J. (2018). Peace Through Development II Burkina Faso, Chad and Niger Impact Evaluation Endline Report. USAID. | Lack of clear boundary between primary and secondary prevention |
| Hiariej, E., Rachmawati, A. D., Taek, A. M., Kurniasari, M., Alvian, R. A. (2017). Final Evaluation Reducing the Recruitment and Recidivism of Violent Extremists in Indonesia. Search for Common Ground. | Lack of clear boundary between primary and secondary prevention |
| International Management Systems (2017). Innovative Approaches to CVE Programming: Insights and Lessons from the USAID OTI Lebanon Community Resilience Initiative. | Lack of clear boundary between primary and secondary prevention |
| Ipp, O., Prado, A., & Fourati, Y. A. (2014). Tunisia Transition Initiative (TTI) Final Evaluation Report. USAID. | Lack of clear boundary between primary and secondary prevention |
| Khalil, J. & Zeuthen, M. (2014). Qualitative Study on Countering Violent Extremism (CVE) Programming Under the Kenya Transition Initiative (KTI). USAID. | Lack of clear boundary between primary and secondary prevention |
| Khalil, J., Brown, R., Chant, C., Olowo, P., & Wood, N. (2019). Deradicalisation and disengagement in Somalia: Evidence from a rehabilitation programme for former members of Al-Shabaab. RUSI. | Intervention was not considered to operate outside of the criminal justice system based on context. |
| Khan, I. (2024). The Resurgence of Militant Groups in Pakistan's Borderlands: Analyzing Shortcomings in Rehabilitation and Reintegration Strategies. Journal for Deradicalization, (38), 1-27. | Intervention was not considered to operate outside of the criminal justice system based on context. |
| Kollmorgen, J. C., Ogada, M., Korir, S., & Dena, E. (2019). Strengthening Community Resilience Against Extremism (SCORE). USAID | Lack of clear boundary between primary and secondary prevention |
| Kurtz, J., Tesfaye, B., & Wolfe, R.J. (2018). Can economic interventions reduce violence? Impacts of vocational training and cash transfers on youth support for political violence in Afghanistan. Washington, DC: Mercy Corps. | Lack of clear boundary between primary and secondary prevention |
| Lamhaidi, N. (2017). Women's Caravan for Peace Final Evaluation. Search for Common Ground | Lack of clear boundary between primary and secondary prevention |
| Levy, D., Jamankulov, K., & Sartbay, T. (2019). Project Evaluation #JashStan: Youth as Agents of Peace and Stability in Kyrgyzstan. Search for Common Ground | Lack of clear boundary between primary and secondary prevention |
| Mbugua, P. K., & Ayoub, N. T. (2023). Religious Peacebuilding’s Response to Violent Extremism in Informal Settlements in Egypt. In S. M. Kilonzo, E. Chitando, & J. Tarusarira (Eds.). The Palgrave Handbook of Religion, Peacebuilding, and Development in Africa (pp. 455-473). Cham: Springer International Publishing. | Lack of clear boundary between primary and secondary prevention |
| Meinema, E. (2020). ‘Idle minds’ and ‘empty stomachs’: Youth, violence and religious diversity in coastal Kenya. Africa, 90(5), 890-913. | Lack of clear boundary between primary and secondary prevention |
| Mercy Corps (2015). Does youth employment build stability? Evidence from an impact evaluation of vocational training in Afghanistan. Washington, DC: Mercy Corps. | Lack of clear boundary between primary and secondary prevention |
| Monzani, B., Sarota, A., Venturi, B. (2018). Evaluation Report Inuka! Community-Led Security Approaches to Violent Extremism in Coastal Kenya. Search for Common Ground and Agency for Peacebuilding. | Lack of clear boundary between primary and secondary prevention |
| Murtaza, N., Sohail, A., Perveen Shaikh, R., Ahmed, S., Anver, S., Ahmad, A., Asghar, M., Ashraf, J., & Yar Khan, U. (2018). Punjab Youth Workforce Development Project Midterm Evaluation. USAID | Lack of clear boundary between primary and secondary prevention |
| Odjidja, E. N., Ayaluri, S. S., & Fiodorov, D. (2024). Do Livelihood Interventions Contribute to the Prevention of Violent Extremism? A Realist Evaluation. The RUSI Journal, 169(3), 52-66. | Lack of clear boundary between primary and secondary prevention |
| Peracha, F., Savage, S., Khan, R., Ayub, A., & Zahra, A. (2022). Promoting cognitive complexity among violent extremist youth in Northern Pakistan. Journal of Strategic Security, 15(1), 14-53. | Intervention was not considered to operate outside of the criminal justice system based on context. |
| Peracha, F., Khan, R. R., & Savage, S. (2016). Sabaoon: Educational methods successfully countering and preventing violent extremism. In S. Zeiger & A. Aly (Eds.). Expanding research on countering violent extremism (pp. 85-104). Hedayah. | Intervention was not considered to operate outside of the criminal justice system based on context. |
| Sarota, A. (2017). Baseline Evaluation of: Katika Usalama Tunategemeana and Pamoja! Strengthening Community Resilience in Tanzania. Search for Common Ground | Lack of clear boundary between primary and secondary prevention |
| Schumicky-Logan, L. (2017). Addressing violent extremism with a different approach: The empirical case of at-risk and vulnerable youth in Somalia. Journal of Peacebuilding & Development, 12(2), 66-79. | Lack of clear boundary between primary and secondary prevention |
| Swedberg, J. (2011). Mid-Term Evaluation of USAID's Counter-Extremism Programming in Africa. USAID. | Lack of clear boundary between primary and secondary prevention |
| Swedberg, J. & Reisman, L. (2013). Mid-term evaluation of three countering violent extremism project. USAID. | Lack of clear boundary between primary and secondary prevention |
| Tines, J., Haq Siddiqui, Noman ul, Akhtar, N., Sadiq, M., Tanveer, T., & Iqbal Zaidi, S. Z. (2017). Karachi Youth Workforce Development Project: Midterm Evaluation Report. USAID. | Lack of clear boundary between primary and secondary prevention |
| Ugwueze, M. I., Ngwu, E. C., & Onuoha, F. C. (2022). Operation safe corridor programme and reintegration of Ex-Boko Haram fighters in Nigeria. Journal of Asian and African Studies, 57(6), 1229-1248. | Intervention was not considered to operate outside of the criminal justice system based on context |
| Tropp, L. R., Bilali, R., & Flickinger, S. (2019). Healing Our Communities: Promoting Social Cohesion in Rwanda. USAID. | Definition of youth |
| USAID (2020). Demobilization, Disassociation, Reintegration, and Reconciliation (DDRR) in Northeast Nigeria: Public report. | Intervention was not considered to operate outside of the criminal justice system based on context. |
| USAID & Education Development Center (2019). USAID's Mindanao Youth for Development (MYDev) Program FY17 Impact Evaluation Report & FY18\|19. USAID. | Lack of clear boundary between primary and secondary prevention |

**Appendix VI: Studies Awaiting Classification**

Al-Badayneh, D. M., & Alhasan, K. (2016). Religious behavior and radicalization among Arab Youth: Implications for Terrorism Recruitment and De-radicalization. In S. Ekici, H. Akdoğan, & E. Ragab (Eds.). In Countering Terrorist Recruitment in the Context of Armed Counter-Terrorism Operations (pp. 130-145). IOS press.

Amaka, C. E. (2020). Causes of violent youth radicalization: Nigeria's persisting security challenge. Internet Journal of Restorative Justice

Angel, H. (2008). Islamism, radicalisation and young people. Safer Communities, 7(2), 12-18.

Awan, I. (2012). Muslim communities, conflict and terrorism: a study of Alum Rock. Safer Communities, 11(4), 195-204.

Awan, I., & Blakemore, B. (2016). Extremism, Counter-Terrorism and Policing. London: Routledge

Ayhan, U. (2015). Foreign Terrorist Fighters (FTF): Break & End of Violent Extremism, Radicalism (BEVER). Turkish Journal of Police Studies/Polis Bilimleri Dergisi, 17(1).

Campbell, J., Duffy, J., & Simpson, M. (2016). Political conflict and social work education. In I. Taylor, M. Bogo, M. Lefevre & B. Teater (Eds.). Routledge International Handbook of Social Work Education (pp. 275-285). Routledge

Chermak, S. M., Freilich, J. D., & Caspi, D. (2009). Policymakers and law enforcement must consider the unintended consequences of their proposed responses to extremist and terrorist groups. Contemporary issues in criminal justice policy: Policy proposals from the American Society of Criminology Conference.

Cheung, C. K. (2023). Deradicalization in response to social experiences in youth in Hong Kong. International Journal of Sociology and Social Policy, 43(7/8), 643-660.

Choudhury, T. (2009). Stepping Out: Supporting Exit Strategies from Violence and Extremism. Institute for Strategic Dialogue.

Coester, M. (2008). Prevention of Hate Crimes and Right-wing Extremism in the State of Lower Saxony and Germany. Conference Papers - American Society of Criminology

Coppock, V., Guru, S., & Stanley, T. (2018). On becoming ‘radicalised’: Pre-emptive surveillance and intervention to save the young Muslim in the UK. In M. Grasso & J. Bessant (Eds.). Governing Youth Youth Politics in the Age of Surveillance (pp. 108-122). Routledge.

Davis, P. K., & Jenkins, B. M. (2012). Toward an analytic basis for influence strategy in counterterrorism. In A. Wenger & A. Wilner (Eds.). Deterring Terrorism: Theory and Practice (pp. 67-94). Stanford Security Studies.

Duzcu, M. (2016). The tragedy of youth in conflict and post-conflict settings: Preventing youth participation in violence and assessing their role in Turkey's Southeast (2009-2015). PhD Thesis, Coventry University.

Eatwell, R. (2012). Responses to the extreme right in Britain. In R. Eatwell & M. Goodwin (Eds.). The New Extremism in 21st Century Britain (pp. 211-230). Routledge.

El-Said, H. (2015). New Approaches to Countering Terrorism: Designing and Evaluating Counter Radicalization and De-Radicalization Programs. Springer.

Eriksson, A. (2008). Challenging cultures of violence through community restorative justice in Northern Ireland. In H. Ventura Miller (Ed.). Restorative Justice: From Theory to Practice (pp. 231-260). Emerald Group Publishing Limited.

Euer, K., Krols, Y., Van Bouchaute, K., Groenen, A., & Paoli, L. (2014). Strengthening Resilience against Violent Radicalization (STRESAVIORA), Part III: Conclusions and Recommendations.

Eurocities (2016). City Responses on Preventing Radicalisation and Violent Extremism: Social Inclusion as a Tool?

Faccini, L., & Allely, C. S. (2017). Rare instances of individuals with autism supporting or engaging in terrorism. Journal of Intellectual Disabilities and Offending Behaviour, 8(2), 70-82.

Feddes, A., Nickolson, L., Mann, L., & Doosje, B. (2020). Psychological Perspectives on Radicalization. Routledge.

Folami, O. (2016). Strengthening DDR through reparations: An exploration of gender blindness in the Niger Delta post-amnesty reintegration programme. PhD Thesis, Ulster University.

Frankel, S. (2017). Negotiating childhoods. London: Palgrave Macmillan.

Fraser-Rahim, M & Khalid, M. H. (2021). Deradicalization and rehabilitation: A case study of the Quilliam Foundation's decade-long approach in the UK, the United States, and worldwide. In K. Bhui & D. Bhugra (Eds.). Terrorism, Violent Radicalisation and Mental Health (pp. 169–202). Oxford University Press.

Gajimuradova, G., Dobrokhleb, V., Bezverbniy, V., & Khramova, M. (2017). Social and Demographic Factors of Religious-Political Extremism in Central Asia and the Caucasus: How it Emerged and How to Oppose it. Central Asia & the Caucasus (14046091), 18(3).

Giannone, D., & Wilson, R. A. (2003). The cat eyes program: Enlisting community members in the fight against terrorism. Police Chief, 70(3), 37-39.

Gluck, Z. (2019). Recolonizing Security: An Anthropology of the War on Terror in Kenya. PhD Thesis, The City University of New York.

Gunaratna, R., & Hussin, S. (2019). Introduction. In R. Gunaratna. & S. Hussin (Eds). Terrorist Deradicalisation in Global Contexts: Success, Failure and Continuity. Routledge.

Gunaratna, R., Jerard, J., & Nasir, S. M. (2013). Countering Extremism: Building Social Resilience through Community Engagement (Vol. 1). World Scientific.

Hart, N. A. (2021). Reimagining Prevent Duty in schools: How teachers can support students in their liberation from racialised narratives of radicalisation. Equality, Diversity and Inclusion: An International Journal, 40(2), 180-198.

International Alert (2019). Evaluation Summary: Final Evaluation of the Social Cohesion Programme in Northeast Nigeria.

Jacob, J. U. U. (2021). Countermeasures to extremist propaganda: A strategy for countering absolutist religious beliefs in northeast Nigeria. In G, D. Rawnsley, Y. Ma, & K. Pothong (Eds.). Research Handbook on Political Propaganda (pp. 270-284). Edward Elgar Publishing.

Kader, A. H. (2016). Fighting terrorism: Preventing the radicalisation of youth in a secular and globalised world. In Z. A. Rasheed & N. Saat (Eds.). Majulah!: 50 years Of Malay/Muslim Community in Singapore (pp. 256-257). World Scientific.

Kartha, T. (2019). Rehabilitation and reintegration: India's policy approach in Jammu and Kashmir. In R. Gunaratna. & S. Hussin (Eds). Terrorist Deradicalisation in Global Contexts: Success, Failure and Continuity. Routledge.

Kondrakova, E. D. (2018). The programme of Pyatigorsk State University on counter extremism activities on the territory of the educational organization and student dormitories. International Journal of Engineering & Technology, 7(2), 56-61.

Krafchik, M., Ryszkowska, Y., Way, C. G., & Dudley, W. M. (2011). Evaluation of Young and Safe Project: London Borough of Lambeth. Inspira Consulting.

Lambert, R. (2010). The London Partnerships: An Insider's Analysis of Legitimacy and Effectiveness. PhD Thesis: University of Exeter.

Lemkey, L. (2019). Radicalisation of young people: The use of the internet and addressing radicalisation through education. PhD Thesis, University of Birmingham.

Lundie, D. (2018). Multi-agency Prevent work: A whole-curriculum approach. British Journal of School Nursing, 13(2), 92-93.

McDonald, B., & Mir, Y. (2011). Al‐Qaida‐influenced violent extremism, UK government prevention policy and community engagement. Journal of aggression, conflict and peace research, 3(1), 32-44.

McRae, D. (2009). DDR and Localized Violent Conflict: Evaluating Combatant Reintegration Programs in Poso, Indonesia. Indonesian Social Development Paper, 14.

Meringolo, P., Cecchini, C., & Donati, C. (2022). Migrants as suspects? A participatory consensus conference to promote well-being and inclusion. Journal of Prevention & Intervention in the Community, 50(3), 224-239.

Milov, I. E., Yavorskii, M. A., & Khasnutdinov, R. R. (2022). Countering Extremism among Young People with the Help of New Information Technologies. Economic and Legal Foundations of Innovative Development in the Digital Age.

Mirahmadi, H., & Farooq, M. (2011). A community based approach to countering radicalization: A partnership for America. WORDE: World Organization for Resource Development and Education.

Nicolls, M. & Hassan, A. (2014). Evaluation Report Mid-Term Performance Evaluation of the USAID Somali Youth Leaders Initiative.

Nitsch, H., & Irani, D. (2017). Prevention, anti-radicalisation and the role of social media: A view from Germany. In M. Conway, L. Jarvis, O. Lehane, S. Macdonald, & L. Nouri (Eds). Terrorists' Use of the Internet (pp. 257-265). IOS Press.

Okoye, I. E. (2016). The Effectiveness of Governments' Responses to Terrorism: A Comparative Study of Nigeria and United States, 1980-2013. Doctoral dissertation: Texas Southern University.

Oser, F., Riegel, C., & Steinmann, S. (2011). Peace and morality: Two children of the same parents. In G. Salomon & E. Cairns (Eds.). Handbook on peace education (pp. pp. 155-170). Psychology Press.

Owonikoko, S. B. (2020). Amnesty for Boko Haram members-lessons from the amnesty programme for militants in the Niger Delta region. Africa Insight, 49(4), 39-54.

Parent, R. B., & Ellis III, J. O. (2016). Countering Radicalization in the Community and in Prison Environments. Law Enforcement Executive Forum (Vol. 16, No. 3).

Ranstorp, M. (2010). Understanding Violent Radicalisation: Terrorist and Jihadist Movements in Europe. Routledge.

Rubin, L., Gunaratna, R., & Jerard, J. (2011). Terrorist Rehabilitation and Counter-Radicalisation: New Approaches to Counter-Terrorism. Routledge.

Silke, A. (Ed.). (2011). The Psychology of Counter-Terrorism. London: Routledge.

Silvestru, D., Rainer, K., & Springnagel, M. (2018). The AWID prevention approach: The generation of a holistic good practice model for prevention of radicalization in youth work. In Proceedings of the 13th International Conference on Availability, Reliability and Security (pp. 1-5).

Soares, N., Allely, C. S., Straub, F., & Penner, M. (2022). Autism spectrum disorder, extremism, and the role of developmental-behavioral pediatric clinicians. Journal of Developmental & Behavioral Pediatrics, 43(8), 480-488.

Spalek, B., & Lambert, R. (2012). Policing within a counter-terrorism context post-7/7: The importance of partnership, dialogue and support when engaging with Muslim communities. In R. Eatwell, & M. J. Goodwin (Eds.) The New Extremism in 21st Century Britain (pp. 103-122). Routledge.

Spalek, B., McDonald, L. Z. & El-Awa, S. (2010). Police-Community Engagement and Partnership Work within a Counter-Terrorism Context. Prison Service Journal

Sraieb-Koepp, N. (2017). The role of media in public advocacy and countering violent extremism. In R. Andersen & P. L. de Silva (Eds.). Routledge Companion to Media and Humanitarian Action (pp. 261-267). Routledge.

Stanek, R. (2013). Countering Violent Extremism: A Community Partnership Approach. Police Chief

Thompson, S. K.; Leroux, E.; Mirenzi, S.; Hurd, D.; Giffin, L. & Amos, J. (2020). Design and Performance: Developing Canadian Partnerships for Countering Violent Extremism (Final Report). Public Safety Canada.

Van Brunt, B., & Taylor, C. (2020). Understanding and Treating Incels: Case Studies, Guidance, and Treatment of Violence Risk in the Involuntary Celibate Community (1st ed.). Routledge

Victoroff, J. E., & Kruglanski, A. W. (2009). Psychology of Terrorism: Classic and Contemporary Insights. Psychology Press.

Weine, S. (2012). Keeping the Family in Community Resilience. International Society for Traumatic Stress Studies (ISTSS) 28th Annual Meeting: Innovations to Expand Services and Tailor Traumatic Stress Treatments, November 1-3, 2012, Los Angeles, CA [Abstracts]

Weine, S. (2016). Resilience and countering violent extremism. In U. Kumar (Ed.). The Routledge International Handbook of Psychosocial Resilience (pp. 189-201). Routledge.

Yildiz, S., & Göktepe, F. (2011). The Role of Social Projects in Preventing Radicalization in Terrorist Organization. In I. Bal, S. Ozeren, & M. A. Sozer (Eds.). Multi-Faceted Approach to Radicalization in Terrorist Organizations (pp. 77-95). IOS Press.

Young, H., Rooze, M., Russell, J., Ebner, J., & Schulten, N. (2016). Evidence-based Policy Advice. TERRA.

**Appendix VI: Studies Excluded at Full Text**

**Exclusion: Research Design**

Abbas, T. (2019). Implementing ‘Prevent’ in countering violent extremism in the UK: A left-realist critique. Critical Social Policy, 39(3), 396-412

Abbas, T. (2021). The scope and limits of combatting violent extremism in the United Kingdom. Revista CIDOB d'Afers Internacionals(128), 155-174

Abbas, T., & Awan, I. (2015). Limits of UK counterterrorism policy and its implications for Islamophobia and far right extremism. International Journal for Crime, Justice and Social Democracy, 4(3), 16-29

Abdel-Fattah, R. (2020). Countering violent extremism, governmentality and Australian Muslim youth as ‘becoming terrorist’. Journal of Sociology, 56(3), 372-387.

Acil Allen Consulting (2019). NSW COUNTERING VIOLENT EXTREMISM PROGRAM EVALUATION. Acil Allen Consulting.

Ademi, M., & Vula, V. (2023). THE ROLE OF CIVIL SOCIETY FOR PREVENTION AND COMBAT OF VIOLENT EXTREMISM AND RADICALIZATION LEADING TO TERRORISM-WAR. Access to Justice in Eastern Europe, 6(3), 192-203.

Afzal, W., & Hagan, A. (2017). Using virtual reality to counter extremism. Proceedings of the Association for Information Science and Technology, 54(1), 612-613.

Agerschou, T. (2014). Preventing radicalization and discrimination in Aarhus. Journal for Deradicalization (1), 5-22.

Ajodo-Adebanjoko, A. (2022). The Role of Youths in Countering Violent Extremism in Northeast Nigeria. Conflict Trends, 2022(1), 46-54.

Akbarzadeh, S. (2013). Investing in Mentoring and Educational Initiatives: The Limits of De-Radicalisation Programmes in Australia. Journal of Muslim Minority Affairs, 33(4), 451-463.

Aked, H., Younis, T., & Heath-Kelly, C. (2021). Racism, mental health and pre-crime policing: The ethics of Vulnerability Support Hubs.

Ali, R., Šibljakovic, D., Lippe, F., Neuburg, U., & Neuburg, F. (2020). ‘YOU’RE AGAINST DAWLA, BUT YOU’RE LISTENING TO THEIR NASHEEDS?’APPROPRIATING JIHADI AUDIOVISUALITIES IN THE ONLINE STREETWORK PROJECT JAMAL AL-KHATIB–MY PATH! In C. Günther & S. Pfeifer (Eds.), JIHADI AUDIOVISUALITYAND ITS ENTANGLEMENTS (pp. 222-246). Edinburgh University Press

Allais, P. (2022). Countering Radicalization and Terrorism in Indonesia. In A.J. Masys (Ed.). Handbook of Security Science (pp. 1085-1107). Springer.

Aly, A. (2012). Countering violent extremism Social harmony, community resilience and the potential of counter-narratives in the Australian context. Conference on Prevent and Counter-Radicalisation in 2012 - Challenges and Ways Forward.

Aly, A., Balbi, A.-M., & Jacques, C. (2015). Rethinking countering violent extremism: Implementing the role of civil society. Journal of Policing, Intelligence and Counter Terrorism, 10(1), 3-13.

Anand, A., & Mantovani, G. (2020). Homegrown terrorism: An analysis of its effects on PESTLE factors. Transdisciplinary Perspectives on Risk Management and Cyber Intelligence, 21-46.

Andersen, L. E., & Moe, L. W. (2015). Responding to radicalization: Exporting the dilemmas. In N. Hvidt & H. Mouritzen (Eds.). Danish Foreign Policy Yearbook 2015 (pp. 71-101). Danish Institute for International Studies

Andrews, S. (2020). Prevent Tragedies: A case study in female-targeted strategic communications in the United Kingdom’s Prevent counterterrorism policy. Journal for Deradicalization (24), 1-39.

APA (2017). Why Do Some With Radical Views Become Terrorists Yet Others Don't? APA.

Arbeit, M. R., Burnham, S. L. F., de Four, D., & Cronk, H. (2020). Youth practitioners can counter fascism: What we know and what we need. Journal of Youth Development, 15(5), 37-67.

Ashour, O. (2010). Online De-Radicalization? Countering Violent Extremist Narratives Message, Messenger and Media Strategy. Perspectives on Terrorism, 4(6), 15-19.

Atnashev, V. R. (2016). Impact of Islamophobia and Human Rights: The Radicalization of Muslim Communities. Public Administration, Governance and Globalization, 17, 91-105.

Augestad Knudsen, R. (2020). Measuring radicalisation: risk assessment conceptualisations and practice in England and Wales. Behavioral Sciences of Terrorism and Political Aggression, 12(1), 37-54.

Augestad Knudsen, R. (2021). Between vulnerability and risk? Mental health in UK counter-terrorism. Behavioral Sciences of Terrorism and Political Aggression, 13(1), 43-61.

Awan, A. (2016). Negative Youth Engagement: Participation in Radicalism and Extremism. In United Nations Annual World Youth Report United Nations.

Awan, I. (2012). I am a Muslim not an extremist’: How the Prevent Strategy has constructed a “suspect” community. Politics & Policy, 40(6), 1158-1185.

Ayres, J. M. (2018). Counter-Radicalization: An Analysis on Violent Extremist Ideologies. National American University.

Bács, G. Z. (2022). National Security and Higher Education - Challenges, Risks and Threats. Nemzetbiztonsagi Szemle = National Security Review, 10(3), 91-99.

Baffa, R., Vest, N., Chan, W., & Fanlo, A. (2019). Defining and Understanding the Next Generation of Salafi-Jihadis. RAND.

Bakker, E., & de Graaf, B. (2011). Preventing Lone Wolf Terrorism some CT Approaches Addressed. Perspectives on Terrorism, 5(5/6), 43-50.

Banholzer, L. (2014). When do disarmament, demobilisation and reintegration programmes succeed? German Development Institute.

Barclay, J. (2011). Strategy to Reach, Empower, and Educate Teenagers (STREET): A Case Study in Government- Community Partnership and Direct Intervention to Counter Violent Extremism. Global Center on Cooperative Security.

Barna, C. (2015). The Road to Jihad in Syria-Using SOCMINT to Counter the Radicalization of Muslim Youth in Romania. In M. Lombardi, E. Ragab, & V Chin (Eds.). Countering Radicalisation and Violent Extremism Among Youth to Prevent Terrorism (pp. 190-197). IOS Press.

Barracosa, S., & March, J. (2021). Dealing With Radicalised Youth Offenders: The Development and Implementation of a Youth-Specific Framework. Frontiers in Psychiatry, 12, 773545.

Barton, G. (2019). Australia isn’t taking the national security threat from far-right extremism seriously enough. The Conversation (3 Oct).

Basit, A. (2015). Countering Violent Extremism: Evaluating Pakistan's Counter-Radicalization and De-radicalization Initiatives. Ipri Journal, 15(2), 44-69.

Bastug, M. F., & Evlek, U. K. (2016). Individual disengagement and deradicalization pilot program in Turkey: Methods and outcomes. Journal for Deradicalization (8), 25-45.

Becker, J. (2017). Why the U.S. Military Should Support Domestic CVE. Perspectives on Terrorism, 11(3), 71-76.

Beelmann, A. (2021). Concept of and approaches toward a developmental prevention of radicalization: Promising strategies to keep young people away from political, religious, and other forms of extremism. Monatsschrift Fur Kriminologie Und Strafrechtsreform, 104(3), 298-309.

Beelmann, A., Wermke, M., & Dingfelder, J. (2022). Muslim Youth in Germany. In L. Robinson & M. R. Gardee (Eds.). Radicalisation, Extremism and Social Work Practice: Minority Muslim Youth in the West (pp. 134-157). Routledge.

Bellis, M. A., & Hardcastle, K. (2019). Preventing violent extremism in the UK: Public health solutions. Public Health Wales.

Ben-Cheikh, I., Rousseau, C., Hassan, G., Brami, M., Hernandez, S., & Rivest, M. H. (2018). Intervention in context of radicalization leading to violence: A multidisciplinary clinical approach. Sante Mentale au Quebec, 43(1), 85-99.

Berger, J. M. (2016). Making CVE work. International Centre for Counter-Terrorism (ICCT).

Bergin, A. (2017). Local government and Australian counter-terrorism strategy. Journal of Policing, Intelligence and Counter Terrorism, 12(1), 74-77.

Bertram, L. (2015). How could a terrorist be de-radicalised? Journal for Deradicalization, 5, 120-149.

Beutel, A., & Weinberger, P. (2016). Public-private partnerships to counter violent extremism: Field principles for action. Final Report to the US Department of State.

Bhui, K. S. & Jenkins, R. (2019). Violent Radicalisation: Relational Roots and Preventive Implications. In R. Williams, V. Kemp, S. A. Haslam, C. Haslam, K. S. Bhui, S. Bailey & D. Maughan (2019). Social Scaffolding: Applying the Lessons of Contemporary Social Science to Health and Healthcare (pp. 183-195). Cambridge University Press.

Bhui, K. S., Hicks, M. H., Lashley, M., & Jones, E. (2012). A public health approach to understanding and preventing violent radicalization. BMC Medicine, 10, 16.

Bhulai, R., & Fink, N. C. (2016). Strengthening regional cooperation to prevent and counter violent extremism in South Asia: What role for civil society? Global Center on Cooperative Security.

Bigo, D., Bonelli, L., Guittet, E.-P., & Ragazzi, F. (2014). Preventing and countering youth radicalisation in the EU.

Bilal Nasir, M. (2019). Mad kids, good city: Counterterrorism, mental health, and the resilient Muslim subject. Anthropological Quarterly, 92(3), 817-844.

Bilazarian, T. (2020). Countering Violent Extremist Narratives Online: Lessons from Offline Countering Violent Extremism. Policy and Internet, 12(1), 46-65.

Birt, Y. (2009). Promoting virulent envy? Reconsidering the UK's terrorist prevention strategy. The RUSI Journal, 154(4), 52-58.

Bjorgo, T. (2002). Exit Neo-Nazism Reducing Recruitment and Promoting Disengagement from Racist Groups. NUPI

Bjørgo, T. (2016). Counter-terrorism as crime prevention: A holistic approach. Behavioral Sciences of Terrorism and Political Aggression, 8(1), 25-44.

Bjorgo, T. & Carlsson, Y. (2005). Early Intervention with Violent and Racist Youth Groups. NUPI

Blaydes, L., & Rubin, L. (2008). Ideological reorientation and counterterrorism: Confronting militant Islam in Egypt. Terrorism and Political Violence, 20(4), 461-479.

Botha, S. (2021). The women and girls associated with Boko Haram: How has the Nigerian government responded? South African Journal of International Affairs, 28(2), 263-284.

Boucek, C. (2008). Extremist re-education and rehabilitation in Saudi Arabia. In T. Bjørgo & J. Horgan (Eds.). Leaving Terrorism Behind (pp. 212-223). Routledge.

Boukalas, C. (2019). The Prevent paradox: Destroying liberalism in order to protect it. Crime Law and Social Change, 72(4), 467-482.

Bovenkerk, F. (2011). On leaving criminal organizations. Crime, Law and Social Change, 55(4), 261-276.

Brett, J., Eriksen, K. B., & Sorensen, A. K. R. (2015). Lessons learned from Danish and other international efforts on Countering Violent Extremism (CVE) in development contexts. Ministry of Foreign Affairs of Denmark.

Briggs, R. (2010). Community engagement for counterterrorism: Lessons from the United Kingdom. International Affairs, 86(4), 971-981.

Broadbent, R. (2013). Using Grass Roots Community Programs as an Anti-Extremism Strategy. Australian Journal of Adult Learning, 53(2), 187-210.

Bronsard, G., A, C., & Vermeulen, F. (2022). Editorial: Radicalization Among Adolescents. Frontiers Psychiatry, 13.

Brooks, N., Honnavalli, V., & Jacobson-Lang, B. (2022). Children of ISIS: Considerations regarding trauma, treatment and risk. Psychiatry, Psychology and Law, 29(1), 107-133.

Brown, K. E. (2023). Gender, governance, and countering violent extremism (CVE) in the UK. International Journal of Law, Crime and Justice, 72, 100371.

Buana, D. R. (2020). Teenagers and Terrorism in Indonesia; How To Understanding and Preventing. Journal of Critical Reviews, 7(19), 2960-2964.

Bux, S. (2007). Muslim Youths, Islam and Violent Radicalisation: Addressing Some Myths. Police Journal, 80(3), 267-278.

Canadian Association of Chiefs of Police (2008). Building community resilience to violent ideologies. CACP Prevention of Radicalization Study Group

Cantle, T., & Thomas, P. (2014). Taking the Think Project Forward-The Need for Preventative Anti-Extremism Educational Work.

Carmi, E., & Gianfransesco, A. (2017). Serious case review: Siblings W and X. Identifying the strengths and gaps in multi-agency responses to vulnerable adolescents at risk of exploitation through radicalisation. Brighton & Hove Local Safeguarding Children Board. Brighton: Brighton and Hove Local Safeguarding Children's Board (LSCB).

Carnegie, P. J. (2015). Countering the (Re-) Production of Militancy in Indonesia between Coercion and Persuasion. Perspectives on Terrorism, 9(5), 15-26.

Cherney, A. (2016). Designing and implementing programmes to tackle radicalization and violent extremism: lessons from criminology. Dynamics of Asymmetric Conflict, 9(1-3), 82-94.

Cherney, A. (2017). Community engagement to tackle terrorism and violent extremism : challenges, tensions and pitfalls. Policing and Society, 27(7), 750-763.

Cherney, A., De Rooy, K., & Eggins, E. (2021). Mandatory participation in programs to counter violent extremism: A review of evidence for and against. Journal for Deradicalization (27), 1-33.

Chin, V. (2014). Collateral Damage of Counter-terrorism Measures and the Inevitable Consequence of the Social Exclusion and Marginalization of Vulnerable Groups. Conference on Countering Violent Extremism among Youth to Prevent Terrorism.

Chowdhury Fink, N. (2015). Thinking Outside the Box: Exploring the Critical Roles of Sports, Arts, and Culture in Preventing Violent Extremism. Hedayah and Global Center on Cooperative Security.

Christensen, T. W. (2019). Lessons learned from P/CVE youth mentorship. RUSI.

Clark, M. (2019). Motivational interviewing for deradicalization: Increasing the readiness to change. Journal for Deradicalization, 20(47-74).

Clarke, C. P. (2020). Conducting P/CVE Assessment in Conflict Environments: Key Considerations.

Clubb, G. (2016). The role of former combatants in preventing youth involvement in terrorism in Northern Ireland: A framework for assessing former Islamic State combatants. Studies in Conflict & Terrorism, 39(9), 842-861.

Clubb, G., & Tapley, M. (2018). Conceptualising De-Radicalisation and Former Combatant Reintegration in Nigeria. Third World Quarterly, 39(11).

Coester, M. (2010). Commentary: Right-Wing Extremism and Bias Crime in Germany. Journal of Ethnicity in Criminal Justice, 8(1), 49-69.

Cohen, J. D. (2016). The next generation of government CVE strategies at home: Expanding opportunities for intervention. Annals of the American Academy of Political and Social Science, 668(1), 118-128.

College of Policing (2023). Multi-agency interventions to address radicalisation. College of Policing.

Connor, R. O., Betancourt, T. S., & Enelamah, N. V. (2021). Safeguarding the Lives of Children Affected by Boko Haram: Application of the SAFE Model of Child Protection to a Rights-Based Situation Analysis. Health & Human Rights: An International Journal, 23(1), 27-41.

Cooley, A., & Cooley, S. (2020). Child's Play: Cooperative Gaming as a Tool of Deradicalization. Journal for Deradicalization (23), 96-133.

Coppock, V., & McGovern, M. (2014). 'Dangerous Minds'? Deconstructing counter-terrorism discourse, radicalisation and the 'psychological vulnerability' of Muslim children and young people in Britain. Children and Society, 28(3), 242-256.

Creative Associates International. (2018). Project Overview: Tunisia ETTYSAL.

Crenshaw, M. (2010). Introduction. In M. Crenshaw (Ed.). The Consequences of Counterterrorism (pp. 1–30). Russell Sage Foundation.

Crone, M. (2018). Preventing violent extremism in Lebanon: experience from a Danish-Lebanese partnership. DIIS.

Dalgaard-Nielsen, A. (2016). Countering Violent Extremism with Governance Networks. Perspectives on Terrorism, 10(6), 135-139.

Dalgaard-Nielsen, A., & Ilum, J. (2020). Promoting disengagement from violent extremism in Scandinavia: What, who, how? In Hansen, S. J. & Lid, S. (Eds.). Routledge Handbook of Deradicalisation and Disengagement (pp. 242-255). Routledge.

Dandurand, Y. (2014). Social Inclusion Programmes for Youth and the Prevention of Violent Extremism. Conference on Countering Violent Extremism among Youth to Prevent Terrorism.

Dandurand, Y. (2015). Social Inclusion Programmes for Youth and the Prevention of Violent Extremism. In M. Lombardi, E. Ragab & V. Chin (Eds.). Countering Radicalisation and Violent Extremism Among Youth to Prevent Terrorism (pp. 23-36). IOS Press

Daugherty, C. E. (2019). Deradicalization and disengagement: Exit programs in Norway and Sweden and addressing neo-Nazi extremism. Journal for Deradicalization (21), 219-260.

Davies, G., Neudecker, C., Ouellet, M., Bouchard, M., & Ducol, B. (2016). Toward a framework understanding of online programs for countering violent extremism. Journal for Deradicalization (6), 51-86.

Davies, L. (2014). Interrupting extremism by creating educative turbulence. Curriculum Inquiry, 44(4), 450-468.

Davies, L. (2018). Review of educational initiatives in counter-extremism internationally: What works? Segerstedt Institute, University of Gothenburg.

de Koning, M. (2023). Risking Muslims: Counter- Radicalisation Policies and Responses of Dutch Muslims to the Racialisation of Danger. In A. Kaya, A. Benevento, & M. Koca (Eds.). Nativist and Islamist Radicalism Anger and Anxiety (pp. 161-180). Routledge

De Waele, M. (2019). Belgium: Preventing Radicalisation on a Local Level and Working for an Inclusive Society. In S. Jayakumar (Ed.). Terrorism, Radicalisation and Countering Violent Extremism: Practical Considerations and Concerns (pp. 69-78). Palgrave.

Demant, F., Wagenaar, W., & Donselaar, J. (2009). Racism & Extremism Monitor: Deradicalisation in practice. Ann Frank Stichting.

Demolli, H., & Qerimi, I. (2021). Kosovo's Legislation and Other Mechanisms on Counterterrorism. Studia Europejskie-Studies in European Affairs, 25(1), 169-190.

Derluyn, I., Vindevogel, S., & De Haene, L. (2013). Toward a relational understanding of the reintegration and rehabilitation processes of former child soldiers. Journal of Aggression, Maltreatment and Trauma, 22(8), 869-886.

Dresser, P. (2021). What about hope? A critical analysis of pre-empting childhood radicalisation. Critical Studies on Terrorism, 14(2), 201-224.

DuBois, D. L., & Alem, F. (2017). Mentoring and domestic radicalization. National Mentoring Resource Center Research Review.

Dusseaux-Edom, R., & Boudoukha, A. H. (2022). Muslim Youth in France: About Discrimination, Radicalisation and Terrorist Attacks. In L. Robinson & M. R. Gardee (Eds.). Radicalisation, Extremism and Social Work Practice: Minority Muslim Youth in the West (pp. 70-91). Routledge.

Duvall, J. A. (2012). An analysis of modern state-level terrorist deradicalization campaigns. Master's Thesis, Naval Postgraduate School

Dzhekova, R. Mancheva, M., Stoynova, N., & Anagnostou, D. (2017). Monitoring Radicalisation: A Framework for Risk Indicators. Center for the Study of Democracy.

Ebiede, T. M. (2018). Assessing the Effectiveness of Employment Programmes for Ex-Combatants: A Case Study of Nigeria's Post Amnesty Programme (PAP). IDS Bulletin-Institute of Development Studies, 49(5), 105-121.

Egloff, G. (2015). Violence prevention and beyond - Theses on the spirit of terrorism. Egyptian Journal of Forensic Sciences, 5(3), 75-81

Eisenman, D. P., & Flavahan, L. (2017). Canaries in the coal mine: Interpersonal violence, gang violence, and violent extremism through a public health prevention lens. International Review of Psychiatry, 29(4), 341-349.

Ekici, S. (2014). Countering Violent Extremism Among Youth: The Turkish Case. Conference on Countering Violent Extremism among Youth to Prevent Terrorism.

El-Amraoui, A. F., & Ducol, B. (2019). Family-oriented P/CVE programs: Overview, challenges and future directions. Journal for Deradicalization, (20), 190-231.

El-Said, H. (2012). De-radicalising Islamists: Programmes and their impact in Muslim majority states. ICSR.

Ellis, B. H., Miller, A. B., Schouten, R., Agalab, N. Y., & Abdi, S. M. (2022). The Challenge and Promise of a Multidisciplinary Team Response to the Problem of Violent Radicalization. Terrorism and Political Violence, 34(7), 1321-1338.

Ellis, B., & Abdi, S. (2017). Building community resilience to violent extremism through genuine partnerships. American Psychologist, 72(3), 289-300.

Ennaji, M. (2016). Recruitment of foreign male and female fighters to Jihad: Morocco’s multifaceted counter-terror strategy. International Review of Sociology, 26(3), 546-557.

Ensign, M. M. (2016). "We are Obsessed with Peace": A Story of Peace Building in Northeastern Nigeria. Contemporary French and Francophone Studies, 20(2), 168-175

Ermakov, P. N., & Brizhak, Z. I. (2010). Higher education and the system of counteraction to ideology of terrorism. Rossiyskiy Psikhologicheskiy Zhurnal, 7(5-6), 57-64.

Evans, C. (2020). Countering violent extremism, safeguarding and the law: A practitioner’s perspective on protecting young and vulnerable people from exploitation. Sociology of Crime Law and Deviance, 25, 131-151.

Federal Ministry for Family Affairs, Senior Citizens, Women and Youth (2018). German Federal Government Report on the Work and Effectiveness of the Federal Government Programmes to Prevent Extremism.

Fedotova, O. D. (2010). A sources of a problem of pedagogical prevention of terrorism: The approach of the West German researcher. Rossiyskiy Psikhologicheskiy Zhurnal, 7(5-6), 104-108.

Finn Church Aid (2021). REACH OUT 3 FINAL REPORT: Organisations, Authorities and Religious Communities Cooperate in Support of Families.

Flood, M. (2023). Engaging men online: Using online media for violence prevention with men and boys. In K. Boyle & S. Berridge (Eds.) The Routledge Companion to Gender, Media and Violence (pp. 491-500). Routledge.

Ford, K. (2017). Developing a Peace Perspective on Counter-Extremist Education. Peace Review, 29(2), 144.

Freear, M., & Andrew, G. (2020). Preventive Communication: Emerging Lessons from Participative Approaches to Countering Violent Extremism in Kenya. The RUSI Journal, 165(1), 90-106.

Freizer, S. (2019). Return of women from ISIS controlled territory: the challenge for Turkey. Turkish Policy Quarterly, 18(2), 30.

Garbarino, J., Governale, A., Henry, P., & Nesi, D. (2015). Children and Terrorism. Social Policy Report. Volume 29, Number 2.

Gavrilovici, O., Dronic, A., & Remaschi, L. (2020). Innovative methods for the interventions in preventing violent radicalisation. In P. Meringolo (Eds.). Preventing Violent Radicalisation in Europe: Multidisciplinary Perspectives (pp. 59-84). Springer

Gereluk, D. (2023). A Whole-School Approach to Address Youth Radicalization. Educational Theory, 73(3), 434-451.

Gielen, A. J. (2018). Exit programmes for female jihadists: A proposal for conducting realistic evaluation of the Dutch approach. International Sociology, 33(4), 454-472.

Gielen, A.-J. (2020). Deradicalisation and disengagement in the Benelux. In S. J. Hansen & S. Lid (Eds.). Routledge Handbook of Deradicalisation and Disengagement (pp. 211-223). Routledge.

Gizyatova, L. A. (2019). School - Based Extremism and Drug Abuse Prevention in the UK. 5th International Forum on Teacher Education (IFTE), 963-968.

Glaser, M., Greuel, F., Herding, M., Hohnstein, S., & Langner, J. (2017). Young and Radical: Political Violence during Adolescence. Deutsches Jugendinstitut.

Gorbunova, J. (2016). Modern information and educational environment: new possibilities of designing and implementing measures to counter extremist sentiments among the students. International Conference on Education Environment for the Information Age (EEIA), 29.

Götsch, K. (2017). Austria and the threats from Islamist radicalisation and terrorist involvement: An overview of governmental and non-governmental initiatives and policies. Journal for Deradicalization (12), 169-191.

Greenberg, K. J. (2016). Counter-radicalization via the Internet. Annals of the American Academy of Political and Social Science, 668(1), 165-179.

Griffith-Dickson, G., Dickson, A., & Robert, I. (2014). Counter-extremism and De-radicalisation in the UK: A Contemporary Overview. Journal for Deradicalization (1), 26-37.

Halafoff, A., & Wright-Neville, D. (2009). A missing peace? The role of religious actors in countering terrorism. Studies in Conflict & Terrorism, 32(11), 921-932.

Hardy, K. (2019). Countering right-wing extremism: lessons from Germany and Norway. Journal of Policing, Intelligence and Counter Terrorism, 14(3), 262-279.

Hassan, M. H. (2007). Singapore’s Muslim Community -Based Initiatives against JI. Perspectives on Terrorism, 1(5), 3-8.

Haugstvedt, H., & Kruse, E. L. (2022). Gazing inwards: A Discussion of the Potential of Norwegian Social Workers to Counteract the Negative Looping Effect of Radicalisation Labels. Journal for Deradicalization, 31, 73-94.

Heath-Kelly, C. (2012). Reinventing prevention or exposing the gap? False positives in UK terrorism governance and the quest for pre-emption. Critical Studies on Terrorism, 5(1), 69-87.

Heath-Kelly, C. (2017). The geography of pre-criminal space: epidemiological imaginations of radicalisation risk in the UK Prevent Strategy, 2007–2017. Critical Studies on Terrorism, 10(2), 297-319.

Hellmuth, D. (2015). Countering jihadi terrorists and radicals the French way. Studies in Conflict & Terrorism, 38(12), 979-997.

Hemmingsen, A.-S. (2015). An introduction to the Danish approach to countering and preventing extremism and radicalization. DIIS.

Heng, Y. K. (2022). Total Defence: Civil Society in Singapore and the Struggle Against Global Terrorism. In K. Um & C. Takenaka (Eds.). Globalization and Civil Society in East Asian Space (pp. 187-207). Routledge.

Hervé, A. (2023). Preventing violent extremism in cross-border villages in the Lake Chad Basin countries: A lesson in cross-border cooperation. In D. Houehounha & E. Moukala (Eds.) Managing Transnational UNESCO World Heritage Sites in Africa (pp. 115-120). Springer.

Herzog-Evans, M. (2018). A comparison of two structured professional judgment tools for violent extremism and their relevance in the French context. European Journal of Probation, 10(1), 3-27.

Heydemann, S. (2014). Countering violent extremism as a field of practice. USIP.

Holdaway, L. (2017). Violent extremism: Four tips for effective programming. International Alert.

Holmer, G. (2013). Countering Violent Extremism: A Peacebuilding Perspective. USIP.

Horgan, J. (2008). Deradicalization or Disengagement? A Process in Need of Clarity and a Counterterrorism Initiative in Need of Evaluation. Perspectives on Terrorism, 2(4), 3-8.

Horgan, J., Lorig, C., Borum, R., Allely, C. S., & Herrenkohl, T. I. (2024). Understanding and preventing violent extremism in school settings. Journal of School Psychology, 106.

Human Security Collective (2018). Community-Based Preventive and Remedial Measures to Prevent Violent Extremism: A Human Security Approach to Help Transform Conflicts, Improve Social Cohesion and Improve Local Security. International Annuals of Criminology, 56, 198-219.

Huq, A. Z. (2020). Community-led counterterrorism. In B. Spalek & D. Weeks (Eds.). Communities and Counterterrorism (pp. 52-67). Routledge.

Hussein, S. (2021). Preventing Violent Extremism the Lebanese Way A Critical Analysis of the Lebanese PVE Strategy. Perspectives on Terrorism, 15(5), 72-84.

Idris, I. (2019). Preventing/countering violent extremism programming on men, women, boys and girls. Institute of Development Studies.

Iganski, P. (2012). Hate crime: taking stock: programmes for offenders of hate. Northern Ireland Association for the Care and Resettlement of Offenders (7.27).

Ilyas, M. (2021). De-Radicalisation and Humanitarianism in Indonesia. Social Sciences-Basel, 10(3), 17.

Jaipal, R. & Modico, J. (2020). Contribution To The Report By The UN Special Rapporteur On Racism And Discrimination by The American Psychological Association At The UN.

Jayakumar, S. (2021). Research Note: Singapore’s “Deradicalisation” Model: Revolution – or Evolutions? Journal for Deradicalization (26), 227-246.

Johansen, M.-L. (2018). Navigating the politics of anxiety: Moral outrage, responsiveness, and state accountability in Denmark. Conflict and Society, 4(1), 9-22.

Jones, J. J. (2010). Countering Islamic radicalization and al Shabaab recruitment within the ethnic Somali population of the United States: An argument for applying best practices for stemming youth gang recruitment and initiation. Masters' Thesis, Naval Postgraduate School.

Jugl, I. (2022). Breaking up the Bubble: Improving critical thinking skills and tolerance of ambiguity in deradicalization mentoring. Journal for Deradicalization (30), 45-80.

Juntunen, M. (2021). When a Family Member becomes Radicalised: Prevention of Violent Extremism and Family Support Activities in Six Western European Countries. Finn Church Aid & EU Internal Security Fund.

Kakhuta-banda, F. B. (2018). Countering Terrorism Through Youth Deradicalisation in Africa Case Study of Kenya. PhD Thesis, University of Nairobi

Käsehage, N. (2017). De-Radicalising Militant Salafists. Perspectives on Terrorism, 11(1), 77-79.

Kaya, Z. (2016). Letting go: De-radicalization in Egypt. Journal for Deradicalization (6), 87-104.

Khalil, J., & Zeuthen, M. (2014). A case study of counter violent extremism (CVE) programming: Lessons from OTI’s Kenya transition initiative. Stability, 3(1).

Khan, M.G. (2013). Youth work, community cohesion, preventing violent extremism and Islamophobia: a symbiotic relationship. In Young Muslims, Pedagogy and Islam. Policy Press.

Klima, N., Pauwels, L., & Hardyns, W. (2021). Evaluating multi-agency working in extremism prevention. 26th German Prevention Congress 2021/14th Annual International Forum (AIF).

Koehler, D. (2013). Family Counselling as Prevention and Intervention Tool Against ‘Foreign Fighters’. The German ‘Hayat’ Program. Journal Exit-Deutschland, 3, 182-204.

Koehler, D. (2015). De-radicalization and disengagement programs as counter-terrorism and prevention tools. Insights from field experiences regarding German right-wing extremism and jihadism. In M. Lombardi., E. Ragab, & V. Chin (Eds.). Countering Radicalisation and Violent Extremism Among Youth to Prevent Terrorism (pp. 120-150). IOS Press.

Koehler, D. (2016). Understanding deradicalization: Methods, tools and programs for countering violent extremism. Routledge.

Koehler, D. (2017). Preventing violent radicalisation: Programme design and evaluation. In D. Muro (Ed.). Resilient cities: countering violent extremism at local level (pp. 91-98). CIDOB.

Koehler, D. (2017). Structural quality standards for work to intervene with and counter violent extremism. German Institute on Radicalization and De-Radicalization Studies.

Koehler, D. (2019). Are there ‘best practices’ in deradicalisation? Experiences from frontline intervention and comparative research. In S. Jayakumar (Ed.) Terrorism, Radicalisation & Countering Violent Extremism (pp. 59-68), Palgrave.

Koehler, D., Buchheit, F., & Sari-Turan, A. (2018). Applying quality standards in countering violent extremism and deradicalization. The case of Baden-Württemberg. In A. Armborst, E. Marks, C. Trautmann, & S. Ullrich (Eds.) Countering violent extremism: Building an evidence-base for the prevention of radicalization and violent extremism (pp. 75-90). Pro BUSINESS.

Konze, A. (2016). Deradicalisation of Foreign Fighters. 11th Biennial Conference on Criminal Justice and Security in Central and Eastern Europe - Safety, Security, and Social Control in Local Communities, 277-283.

Korn, J. (2016). European CVE Strategies from a Practitioner’s Perspective. Annals of the American Academy of Political and Social Science, 668(1), 180-197.

Kovack, L. N. (2017). ACT as Potential for De-Radicalizing, Disengaging, and Reintegrating U.S. Youth. Peace Review, 29(2), 160-169.

Kruber, S., Wahid, Y., Vergani, M., & Barton, G. (2022). Conclusion: The Way Forward. In G. Barton, M. Vergani, & Y. Wahid (Eds.) Countering Violent and Hateful Extremism in Indonesia. New Security Challenges (pp. 295-308). Palgrave Macmillan.

Kuwali, D. (2022). Countering violent extremism in Africa. In D. Kuwali (Ed.). The Palgrave Handbook of Sustainable Peace and Security in Africa (pp. 197-216). Palgrave Macmillan.

Lankford, A., & Gillespie, K. (2011). Rehabilitating terrorists through counter-indoctrination: Lessons learned from the Saudi Arabian program. International Criminal Justice Review, 21(2), 118-133.

Larsson, G., & Mattsson, C. (2022). Social Work as a Tool for Countering Violent Extremism in Sweden. In L. Robinson & M. R. Gardee (Eds.). Radicalisation, Extremism and Social Work Practice: Minority Muslim Youth in the West (pp. 217-232). Routledge.

Lauland, A., Moroney, J. D. P., Rivers, J. G., Bellasio, J., & Cameron, K. (2019). Countering Violent Extremism in Australia and Abroad: A Framework for Characterising CVE Programs in Australia, the United States, and Europe. RAND Corporation.

Lavarias, R. B. (2015). Winning the Hearts and Minds: Improving U.S. Counter-Radicalization Efforts Through a Study of The United Kingdom’s PREVENT programme. Homeland Security Affairs.

Lavenne-Collot, N., Dissaux, N., Campelo, N., Villalon, C., Bronsard, G., Botbol, M., & Cohen, D. (2022). Sympathy-empathy and the radicalization of young people. Children, 9(12), 1889.

Lesniewicz, A. L. S. (2009). Alternatives to punishment: Counterterrorism strategies in Algeria. University of Maryland, College Park.

Limbada, Z., & Davies, L. (2016). Addressing the Foreign Terrorist Fighter Phenomenon from a Human Rights Perspective. International Community Law Review, 18(5), 483-493.

Lindekilde, L. (2014). Refocusing Danish counter-radicalisation efforts: An analysis of the (problematic) logic and practice of individual de-radicalisation interventions. In C. Baker-Beall, C. Heath-Kelly & L. Jarvis (Eds.). Counter-Radicalisation: Critical Perspectives (pp. 223-241). Routledge.

Lodenius, A.-L. (2010). To leave a destructive life full of hate: The Story of Exit in Sweden. Exit Fryshuset.

Lohninger, E. (2024). EVALUATION BRIEF-STRIVE Juvenile: Preventing and Responding to Violence Against Children by Terrorist and Violent Extremist Groups.

Lombardi, M., Ragab, E., & Chin, V. (Eds.). (2014). Countering radicalisation and violent extremism among youth to prevent terrorism (Vol. 118). IOS Press.

Lowe, D. (2023). Hate Crime in Northern Ireland: The Need for Legislation and a Bespoke Version of the Prevent Strategy. Terrorism and Political Violence.

Lowry, K. (2018). Responding to the challenges of violent extremism/terrorism cases for United States Probation and Pretrial Services. Journal for Deradicalization (17), 28-88.

MacVicar, I. C. (2020). What About the Camp Followers – and their Children? Journal for Deradicalization (22), 319-378.

Masyhar, A., Maskur, M. A., Prasetyowati, S. R., Prihama, A. E., Priyono, R., & Alif, A. (2022). Digital transformation of youth movement for counter radicalism. AIP Conference Proceedings, 2573.

McDonald, L. (2011). Securing Identities, Resisting Terror: Muslim Youth Work in the UK and Its Implications for Security. Religion, State and Society, 39(2–3), 177-189.

McDonald, L. Z. (2012). Engaging young people within a counter-terrorism context. Counter-Terrorism: Community-Based Approaches to Preventing Terror Crime, 119-136.

McKenzie, A., & O’Brien, D. (2024). The Evolution of Estimated Time of Arrival: The City of Toronto’s CVE Program. Journal for Deradicalization (39), 94-128.

McLean, L. H., & Fraser, E. (2009). Youth Exclusion, Violence, Conflict and Fragile States. Social Development Direct.

Meyers, L. (2007). Little girls lost. Monitor on Psychology, 38(2), 34.

Mikhael, D., & Norman, J. (2018). Refugee youth, unemployment and extremism: countering the myth. Forced Migration Review (57), 57-58.

Miller-Idriss, C., & Pilkington, H. (2017). In Search of the Missing Link: Gender, Education and the Radical Right. Gender and Education, 29(2), 133-146.

Mirahmadi, H., Ziad, W., Farooq, M., & Lamb, R. D. (2015). Empowering Pakistan's Civil Society to Counter Global Violent Extremism. In: Center for Middle East Policy at Brookings.

Mitchell, S. (2016). Deradicalization: using triggers for the development of a US program. Journal for Deradicalization (9), 101-125.

Mourad, M. (2018). Tailoring Violent Extremism Prevention: A Targeted Intervention Method Homeland Security Affairs.

Mucha, W. (2017). Polarization, stigmatization, radicalization. Counterterrorism and homeland security in France and Germany. Journal for Deradicalization (10), 230-254.

Mullins, S. (2010). Rehabilitation of Islamist terrorists: Lessons from criminology. Dynamics of Asymmetric Conflict, 3(3), 162-193.

Myers, E., & Hume, E. (2018). PEACEBUILDING APPROACHES TO PREVENTING AND COUNTERING VIOLENT EXTREMISM: Assessing the evidence for key theories of change.

Naado, H. O. (2011). Countering violent extremism among Kenyan Muslim youth (Policy Brief).

Nalani, A., & Yoshikawa, H. (2023). White Christian Nationalism and Youth Development in the USA. Society, 60(4), 551-565.

Nemr, C., & Savage, S. (2019). Integrative complexity interventions to prevent and counter violent extremism. Global Center on Cooperative Security.

Neumann, P. R. (2013). Options and Strategies for Countering Online Radicalization in the United States. Studies in Conflict and Terrorism, 36(6), 431-459.

Novelli, M. (2017). Education and Countering Violent Extremism: Western Logics from South to North? Compare: A Journal of Comparative and International Education, 47(6), 835-851.

O'Donnell, A. (2018). Contagious Ideas: Vulnerability, Epistemic Injustice and Counter-Terrorism in Education. Educational Philosophy and Theory, 50(10), 981-997.

O’Halloran, P. (2017). The challenges of evaluating attitudinal change: A case study of the effectiveness of international countering violent extremism (CVE).

Onapajo, H. (2020). Children in Boko Haram Conflict: The Neglected Facet of a Decade of Terror in Nigeria. African Security, 13(2), 195-211

Osman, R. b. A. H., & Mekki, A. (2017). The Tiger and the Terrorist: How Malaysian NGOs deal with Terrorism. Intellectual Discourse, 25(2), 297-319.

Ostwaldt, J. (2018). Closing the “Critical Disconnect “. The establishment of regional prevention networks at the interface of prevention and deradicalisation work using the example of the Federal State Democracy Centre Baden-Wuerttemberg. Journal for Deradicalization (14), 218-248.

Özerdem, A., & Podder, S. (2011). Disarming Youth Combatants: Mitigating Youth Radicalization and Violent Extremism. Journal of Strategic Security, 4(4), 63-80.

Parent, R. B., & Ellis, J. O., III. (2011). Countering radicalization of diaspora communities in Canada (Working Paper Series No. 11-12).

Pašagić, A. (2019). Between child soldiers and terrorists: Reintegrating child members of the Islamic State. Journal for Deradicalization (20), 109-155.

Patel, F., & Koushik, M. (2017). Countering Violent Extremism. In: Brennan Center for Justice.

Pathe, M. T., Haworth, D. J., Goodwin, T.-A., Holman, A. G., Amos, S. J., Winterbourne, P., & Day, L. (2018). Establishing a joint agency response to the threat of lone-actor grievance-fuelled violence. Journal of Forensic Psychiatry & Psychology, 29(1), 37-52.

Patón-Romero, J. D., & Lovås, I. V. (2022). A Proposal to Develop Software to Prevent Young People to Acquire Extremist Beliefs. European Journal of Sustainable Development, 11(4), 158-168.

Patouris, E., & Toscano, T. (2020). Editorial. Internet Journal of Restorative Justice (IJRJ), 7-8.

Patricia Andrews, F., & Boyd-MacMillan, E. M. (2016). Complexity Under Stress: Integrative Approaches to Overdetermined Vulnerabilities. Journal of Strategic Security, 9(4), 11-31.

Pauha, T., & Ritola, V. (2022). Muslim Youth in Finland. In L. Robinson & M. R. Gardee (Eds.). Radicalisation, Extremism and Social Work Practice: Minority Muslim Youth in the West (pp. 196-216). Routledge.

Pllana, M. H. (2021). Strategic communication and prevention of violent extremism through education: The case of Kosovo. Journal of Educational and Social Research, 11(2), 106-114.

Police Executive Research Forum. (2017). Building Interdisciplinary Partnerships to Prevent Violent Extremism.

Powers, S. T. (2015). Expanding the Paradigm: Countering Violent Extremism in Britain and the Need for a Youth Centric Community Based Approach. Journal of Terrorism Research, 6(1), 19-26.

Proctor, K., & Mazurana, D. (2018). The role of gender in mobilizing and countering fundamentalist violent extremist organizations. Routledge Handbook of Gender and Security, 227-238.

Qureshi, A. (2015). PREVENT: Creating “radicals” to strengthen anti-Muslim narratives. Critical Studies on Terrorism, 8(1), 181-191.

Ragazzi, F. (2017). Countering terrorism and radicalisation: Securitising social policy? Critical Social Policy, 37(2), 163-179.

RAN (2021). National hubs supporting local actors in P/CVE – practitioners’ insights.

Ranstorp, M., Gustafsson, L., Hyllengren, P., & Ahlin, F. (2016). Preventing and Countering Violent Extremism: An Initial Rapid Evidence Assessment and Analysis Plan Examining Local Authority Action Plans and Programming Elements.

Richardson, C., Berlouis, K. M., & Cameron, P. A. (2017). Radicalisation of young adults in the Balkan States: counter-measures, healthcare provision, and community involvement. Journal for Deradicalization (11), 87-111.

Richardson, C., Cameron, P. A., & Berlouis, K. M. (2017). The role of sport in deradicalisation and crime diversion. Journal for Deradicalization (13), 29-48.

Ridwan, W. (2022). The Dynamics of Islamic Mass Organisations in Preventing Violent Extremism. In G. Barton, M. Vergani, & Y. Wahid (Eds.). Countering Violent and Hateful Extremism in Indonesia: Islam, Gender and Civil Society. New Security Challenges (pp. 215-231). Springer.

Rieker, P., Glaser, M., & Schuster, S. (2006). Prevention of Right-Wing Extremism, Xenophobia and Racism in European Perspective. Außenstelle Halle.

Robins, A. (2020). Peacetech Technology Education in Post-Conflict Youth Peacebuilding Programs. Education Research and Perspectives, 47, 1-24.

Robinson, L., & Gardee, M. R. (2022). Muslim Youth in the United Kingdom. In L. Robisnson & M. R. Gardee (Eds.).Radicalisation, Extremism and Social Work Practice: Minority Muslim Youth in the West (pp. 21-47). Routledge.

Rogan, J. (2016). Young People’s Participation in Peacebuilding: A practice note. Inter-Agency Network on Youth Development Working Group on Youth and Peacebuilding.

Rousseau, C., Aggarwal, N. K., & Kirmayer, L. J. (2021). Radicalization to Violence: A View from Cultural Psychiatry. Transcultural Psychiatry, 58(5), 603-615.

Rousseau, C., Frounfelker, R., Ngov, C., & Crocker, A. (2023). Clinical Services Addressing Violent Extremism: The Quebec Model. International Journal of Forensic Mental Health, 22(3), 222-232.

RUSI (2017). STRIVE: Lessons Learned: STRIVE. RUSI

RUSI. (2020). STRIVE Lessons Learned: STRIVE II Strengthening Resilience to Violence and Extremism. RUSI

Rusyana, A. Y., Budiman, B., Abdullah, W. S., & Witro, D. (2023). Concepts and Strategies for Internalizing Religious Moderation Values among the Millennial Generation in Indonesia. Religious Inquiries, 12(2), 157-176.

Samuel, T. K. (2018). Engaging youths in counter-violent extremism (CVE) initiatives. In M. Khader, N. L., J. Tan, D. D. Cheong, & J. Chin (Eds.). Seng Learning From Violent Extremist Attacks: Behavioural Sciences Insights For Practitioners And Policymakers (pp. 221-235). World Scientific

Sandyarani, U. (2022). International and Local Actor Collaborations to Prevent Violent Extremism Among Youth in Indonesia: Initiatives and Effectiveness. In G. Barton, M. Vergani, Y. Wahid (Eds.). Countering Violent and Hateful Extremism in Indonesia. New Security Challenges (pp. 125-143), Palgrave Macmillan

Sarma, K. M. (2019). Multi-agency working and preventing violent extremism Paper 2. RAN.

Satterley, S. (2017). On the Trail of Counter Radicalisation: An Examination of Strategies to Prevent the Threat of Islamist Extremism. Master's Thesis, Griffith University.

Scrivens, R., & Perry, B. (2017). Resisting the right: Countering right-wing extremism in Canada. Canadian Journal of Criminology and Criminal Justice, 59(4), 534-558.

Sestoft, D. (2019). Identification and Prevention of Radicalization. Practice and Experiences with a Multidisciplinary Working Model. In D. Marazziti & S, M, Stahl (Eds.). Evil, Terrorism & Psychiatry: Stahl Essential Psychopharmacology Handbooks (pp. 146-154). Cambridge.

Sestoft, D., Hansen, S. M., & Christensen, A. B. (2017). The police, social services, and psychiatry (PSP) cooperation as a platform for dealing with concerns of radicalization. International Review of Psychiatry, 29(4), 350-354.

Sestoft, D., Rasmussen, M. F., Vitus, K., & Kongsrud, L. (2014). The police, social services and psychiatry cooperation in Denmark—A new model of working practice between governmental sectors. A description of the concept, process, practice and experience. International journal of law and psychiatry, 37(4), 370-375.

Shaikh, M., Tiflati, H., Gurski, P., & Amarasingam, A. (2020). Turning the page on extremism: Deradicalization in the North American context. In S. J. Hansen & S. Lid (Ed.). Routledge Handbook of Deradicalisation and Disengagement (pp. 312-325). Routledge.

Shavit, U., & Andresen, S. (2016). Can Western Muslims Be De-radicalized? Middle East Quarterly, 23(4), 1-10.

Sheppard-Luangkhot, T. (2020). Grassroots solutions for radicalized youth and the addictive process of extremism. Internet Journal of Restorative Justice, 2020(Spec Iss 3), 52-62.

Siegel, A., Brickman, S., Goldberg, Z., & Pat-Horenczyk, R. (2019). Preventing future terrorism: Intervening on youth radicalization. In C. W. Hoven, L. V. Amsel, & S. Tyano (Eds.). An international perspective on disasters and children's mental health (pp. 391-418). Springer.

Sitter, N., & Parker, T. (2014). Fighting Fire with Water: NGOs and Counterterrorism Policy Tools. Global Policy, 5(2), 159-168.

Skleparis, D., & Augestad Knudsen, R. (2020). Localising ‘radicalisation’: Risk assessment practices in Greece and the United Kingdom. The British Journal of Politics and International Relations, 22(2), 309-327.

Sounaye, A. (2021). Governing Muslim Subjects in the Sahel: Deradicalisation and a State-Led Islamic Reform in West Africa. In J. E. Dağyeli, C. Ghrawi & U. Freitag (Eds.). Claiming and Making Muslim Worlds: Religion and Society in the Context of the Global (pp. 99-130). De Gruyter.

Spalek, B. (2016). Radicalisation, de-radicalisation and counter-radicalisation in relation to families: Key challenges for research, policy and practice. Security Journal, 29(1), 39-52.

Spalek, B., & Weeks, D. (2020). The role of communities in counterterrorism: Analyzing policy and exploring psychotherapeutic approaches within community settings. In B. Spalek & D. Weeks (Eds.). Communities and Counterterrorism (pp. 5-17). Routledge.

Stanley, T., Guru, S., & Coppock, V. (2017). A risky time for Muslim families: Professionalised counter-radicalisation networks. Journal of Social Work Practice, 31(4), 477-490.

Stanley, T., Guru, S., & Gupta, A. (2018). Working with PREVENT: Social work options for cases of ‘radicalisation risk’. Practice, 30(2), 131-146.

Stewart, C. (2017). Countering violent extremism policy in the United States: are CVE programs in America effectively mitigating the threat of homegrown violent extremism? Homeland Security Affairs.

Straub, F. (2020). The Importance of Community Policing in Preventing Terrorism.

Striegher, J.-L. (2013). The deradicalisation of terrorists. Salus Journal, 1(1), 19-40.

Subedi, D. B. (2017). Early Warning and Response for Preventing Radicalization and Violent Extremism. Peace Review, 29(2), 135.

Summerfield, D. (2016). Mandating doctors to attend counter-terrorism workshops is medically unethical. BJPsych Bulletin, 40(2), 87-88.

Tahir, H. (2022). Radicalisation and Violent Extremism among Norwegian Youth. In L. Robinson and M. R. Gardee (Eds.). Radicalisation, Extremism and Social Work Practice: Minority Muslim Youth in the West (pp. 176-195). Routledge.

Tammikko, T. (2018). The political challenges of community-level PVE practices: The Danish case of Copenhagen vs. Aarhus on dialoguing with extremist milieus. Journal for Deradicalization (16), 103-124.

Taskarina, L., & Veronika, N. W. (2021). Dreaming of the Peaceful Ways: An Evolution of the Indonesian Deradicalisation Program. Technium Soc. Sci. J., 25, 687.

The Global Center on Cooperative Security. (2015). Countering violent extremism and promoting community resilience in the Greater Horn of Africa: An action agenda.

Thomas, P. (2012). Prevent and Community Cohesion in Britain The worst of all possible worlds? Conference on Prevent and Counter-Radicalisation in 2012 - Challenges and Ways Forward, 36-53.

Tireli, Ü. (2022). Muslim Youth in Denmark. In In L. Robinson & M. R. Gardee (Eds.). Radicalisation, Extremism and Social Work Practice: Minority Muslim Youth in the West (pp. 158-175). Routledge.

Trisko Darden, J. (2019). Tackling Terrorists’ Exploitation of Youth. AEI.

UNDP (2021). Annual Report on Prevention of Violent Extremism: Areas of PVE Programming.

UNDP (2021). Applying Behavioural Science to Support the Prevention of Violent Extremism: Experiences and Lessons Learned.

UNDP (2023). Prevention of Violent Extremism: 2022 Annual Report.

USAID (2017). Promising Practices in Engaging Youth in Peace and Security and P/CVE: Summary of Key Interventions and Examples.

Van Brunt, B., Pescara-Kovach, L., & Van Brunt, B. (2023). White supremacist violence: Understanding the resurgence and stopping the spread. Routledge.

Veenkamp, I., Zeiger, S. (2014). Countering Violent Extremism: Program and policy approaches relating to youth through education, families and communities. Conference on Countering Violent Extremism among Youth to Prevent Terrorism, 118, 151-163. Hedayah.

Verdegaal, M., & Haanstra, W. (2017). The role of Youth Work in the Prevention of Radicalisation and Violent Extremism. Radicalisation Awareness Network

Vermeulen, F., van Leyenhorst, M., Roex, I., Schulten, N., & Tuzani, N. (2021). Between Psychopathology and Ideology: Challenges and Practices in Interpreting Young Extremists Experiencing Mental Illness in the Netherlands. Frontiers in Psychiatry, 12, 790161.

Viano, E. C. (2018). Female Migration to ISIS: Conclusions and Recommendations. International Annals of Criminology, 56(1-2), 220-226.

Vidino, L., & Brandon, J. (2012). Countering radicalization in Europe. International Centre for the Study of Radicalisation and Political Violence.

Voogt, S. (2017). Countering far-right recruitment online: CAPE’s practitioner experience. Journal of Policing, Intelligence and Counter Terrorism, 12(1), 34-46.

Walton, O. (2010). Youth, Armed Violence and Job Creation Programmes: A Rapid Mapping Study. Norwegian Peacebuilding Centre and Governance and Social Development Resource Centre.

Weeks, D. (2019). Barking Mosque and Quintessential Insight: Overcoming the Problematic Government/Community Counterterrorism Partnership in the UK. Studies in Conflict & Terrorism, 42(8), 735-754.

Weeks, D. (2021). Lessons learned from UK efforts to deradicalize terror offenders. CTC Sentinel, 14(3), 33-39.

Weilnböck, H. (2012). Anti hate crime and deradicalisation interventions–in prison and community: The results of recent good practice studies in Germany and internationally. Berlin: Violence Prevention Network. In: Berlin: Violence Prevention Network. NIACRO.

Weilnböck, H. (2012). Hate Crime and Radicalisation: the German political experience and the Schellenberg Report. Belfast: NIACRO.

Weilnböck, H., & Kossack, O. (2020). Prevention of group hatred and right-wing extremism in Germany and Central and Eastern European—experiences, lessons learnt and ways forward from the European Fair Skills, Fair* in and CEE Prevent Net projects. International perspectives of crime prevention, 11th edn. Forum Verlag Godesberg GmbH, 159-189.

Weilnböck, H., Baer, S., & Wiechmann, P. (2012). Hate crime prevention and deradicalization in environments vulnerable to extremism: community work with the fair skills approach and the we-among-ourselves group. Zeitschrift des Informations-und Dokumentationzentrums für Antirassismusarbeit in NRW, 3-7.

Weine, S. M., Ellis, B. H., Haddad, R., Miller, A. B., Lowenhaupt, R., & Polutnik, C. (2015). Lessons Learned from Mental Health and Education:Identifying Best Practices for Addressing Violent Extremism. START.

Weine, S. M., Stone, A., Saeed, A., Shanfield, S., Beahrs, J., Gutman, A., & Mihajlovic, A. (2017). Violent extremism, community-based violence prevention, and mental health professionals. The Journal of Nervous and Mental Disease, 205(1), 54-57.

Weine, S., & Braniff, W. (2016). Empowering communities to prevent violent extremism: A report on the August 2014 National Summit. In G. LaFree & J. D. Freilich (Eds.). The Handbook of the Criminology of Terrorism (pp. 449-467). Wiley.

Weine, S., Eisenman, D. P., Jackson, L. T., Kinsler, J., & Polutnik, C. (2017). Utilizing mental health professionals to help prevent the next attacks. International Review of Psychiatry, 29(4), 334-340.

Weine, S., Eisenman, D. P., Kinsler, J., Glik, D. C., & Polutnik, C. (2017). Addressing violent extremism as public health policy and practice. Behavioral Sciences of Terrorism and Political Aggression, 9(3), 208-221.

Weine, S., Horgan, J., Robertson, C., Loue, S., Mohamed, A., & Noor, S. (2009). Community and family approaches to combating the radicalization and recruitment of Somali-American youth and young adults: A psychosocial perspective. Dynamics of Asymmetric Conflict, 2(3), 181-200.

Wichmann, F. (2022). How to use campaigns for exit work An overview of Exit Germany’s target group approach. Journal EXIT-Deutschland.

Williams, M. (2022). Anatomy of process evaluations for P/CVE. Williams, MJ (2022). Anatomy of process evaluations for P/CVE. Journal for Deradicalization, 30, 262-274.

Winterbotham, E. (2024). The Implementation of P/CVE in Conflicts: Lessons from STRIVE Afghanistan.

Wyckoff, R. (2020). Terrorism prevention through community policing. Naval Postgraduate School.

YouthPower. (2017). Promising Practices In Engaging Youth In Peace And Security And P/CVE: Summary of key interventions and examples.

Zahid, F. (2017). Pakistan’s CVE Programme: An Overview of Achievements and Challenges. Counter Terrorist Trends and Analyses, 9(6), 11-16.

Zakuan, U. A. A. (2021). Combating terrorism through community engagement: Experiences from Malaysia and Indonesia. In R. Gunaratna & M. M. Aslam (Eds.). Civil Society Organizations Against Terrorism (pp. 107-127). Routledge.

Zeiger, S. (2016). Expanding Research on Countering Violent Extremism. Hedayah and Edith Cowan University.

Zeiger, S., & Aly, A. (2015). Violent Extremism: Developing an Evidence Base for Policy and Practice. In: Hedayah and Curtin University.

Zeuthen, M. (2020). Disengagement and preventing/countering violent extremism in the Horn of Africa: An analysis of contemporary approaches and discussion of the role disengagement can play in preventing/countering violent extremism. In S. J. Hansen & S. Lid (Eds.). Routledge Handbook of Deradicalisation and Disengagement (pp. 300-311). Routledge.

**Exclusion: Problem**

Ajibola, I. O. (2015). Nigeria's Amnesty Program: The Role of Empowerment in Achieving Peace and Development in Post-Conflict Niger Delta. SAGE Open, 5(3), 11.

Álvarez-Benjumea, A., & Winter, F. (2018). Normative change and culture of hate: An experiment in online environments. European Sociological Review, 34(3), 223-237.

Ashindorbe, K., Afatakpa, F., & Owonikoko, S. B. (2021). Civilian Joint Task Force and Nigeria's Counter-Terrorism Operation: A Critique of the Community-Based Approach to Insecurity. African Security, 14(3), 286-305.

Bangura, I. (2021). SUPPORTING THE SOCIO-ECONOMIC REINTEGRATION OF CHILDREN ASSOCIATED WITH ARMED FORCES AND ARMED GROUPS INCLUDING THE CIVILIAN JOINT TASK FORCE (CJTF) IN NORTHEAST NIGERIA. Search for Common Ground.

Compass, Northern Ireland Association for Mental Health (2012). Challenge To Change: Evaluation Report.

Fiedler, N., Sommer, F., Leuschner, V., & Scheithauer, H. (2019). Student crisis prevention in schools: The NETWorks Against School Shootings Program (NETWASS)-An approach suitable for the prevention of violent extremism? International Journal of Developmental Science, 13(3-4), 109-122.

Hernandez-Cordero, L. J., & Fullilove, M. T. (2008). Constructing peace: helping youth cope in the aftermath of 9/11. American Journal of Preventive Medicine, 34(3 Suppl), S31-35.

Itzhaky, H., & York, A. S. (2005). The role of the social worker in the face of terrorism: Israeli community-based experience. Social Work, 50(2), 141-149.

Jauhiainen, A. (2019). Searching for desistance: A process evaluation of Aggredi, a street violence-focused intervention program. University of Helsinki.

Link, M. R. (2014). An ecological framework for addressing America's youth in armed groups. Adler School of Professional Psychology.

Ng, S., & Nerad, S. (2015). Evaluation of the FOCUS Rexdale pilot project. Delivered to the City of Toronto and Toronto Police Service. In: Vision and Results Inc. and SN Management.

Onyima, J. K. (2017). Sub-Saharan Africa: Societal Reintegration of Ex-Militant Youths. Conflict Studies Quarterly(21), 76-100.

Parkes, C. M. (2014). Responses to terrorism: Can psychosocial approaches break the cycle of violence? Routledge.

Perkoski, E., & Chenoweth, E. (2010). The effectiveness of counterterrorism in Spain: a new approach. Annual Meeting of the International Studies Association, New Orleans, March, 1517.

Sallah, M. (2011). Working with young people in the UK: Considerations of race, religion and globalisation. PhD Thesis, De Montfort University

Shamai, M. (2003). Using social constructionist thinking in training social workers living and working under threat of political violence. Social Work, 48(4), 545-555.

Viñas-Racionero, R., Scalora, M. J., & Ramzah, H. (2022). Blue is a Colour for Help: The Role of Police in De-escalating Threats to a Large University. Policing-a Journal of Policy and Practice, 16(3), 493-507.

Wapmuk, S. (2012). The Amnesty Programme and the Challenges of Ending Youth Militancy in Nigeria’s Niger Delta. Insight on Africa, 4(2), 153-168.

Weine, S., Eisenman, D., Martinez, M., Boyd, L., & Brown, M. (2021). Evaluation of a targeted violence prevention program in Los Angeles County, California. In: University of Illinois at Chicago.

Wessells, M. (2005). Child soldiers, peace education, and postconflict reconstruction for peace. Theory into Practice, 44(4), 363-369.

White, G., Mazerolle, L., Porter, M. D., & Chalk, P. (2014). Modelling the effectiveness of counter-terrorism interventions. Trends and Issues in Crime and Criminal Justice(475), 1-8.

Zerla, P. (2024). Trauma, Violence Prevention, and Reintegration: Learning from Youth Conflict Narratives in the Central African Republic.

**Exclusion: Population**

Aarten, P. G. M., Mulder, E., & Pemberton, A. (2018). The narrative of victimization and deradicalization: An expert view. Studies in Conflict & Terrorism, 41(7), 557-572.

Agastia, I. G. B. D., Perwita, A. A. B., & Subedi, D. B. (2020). Countering violent extremism through state-society partnerships: a case study of de-radicalisation programmes in Indonesia. Journal of Policing, Intelligence and Counter Terrorism, 15(1), 23-43.

Agwu, P. C., Nwangwu, C., & Okoye, U. O. (2022). Post-amnesty peacebuilding and restiveness in the Niger Delta: what do grassroots narratives suggest? Security Journal, 35(3), 754-776.

Ahmed, K., Belanger, P., & Szmania, S. (2018). Community-focused counter-radicalization and counter-terrorism projects: Experiences and lessons learned. Rowman & Littlefield.

Aked, H. (2022). "Mad," bad or Muslim? The UK's Vulnerability Support Hubs and the nexus of mental health, counterterrorism and racism. DP - Mar 2022. Bioethics, 36(3), 290-297.

Al-Kadi, A., & Vale, G. (2020). Local voices against violence: women challenging extremism in Iraq and Syria. Conflict, Security & Development, 20(2), 247-271.

Alonso, R., & Bada, J. D. (2016). What role have former ETA terrorists played in counterterrorism and counterradicalization initiatives in Spain? Studies in Conflict & Terrorism, 39(11), 982-1006.

Ambrozik, C. (2019). Community stakeholder responses to countering violent extremism locally. Studies in Conflict & Terrorism, 42(12), 1044-1068.

Andre, V. (2021). Consequences of Extremist Digital Heritage on the Rehabilitation Process. European Commission

Andrews, S. (2020). Women and 'Prevent': Perceptions, Policy and Encounter. PhD Thesis, University of Lincoln.

Anindya, C. R. (2024). An Indonesian way of P/CVE and interpreting the whole-of-society approach: lessons from civil society organisations. Journal of Policing, Intelligence and Counter Terrorism, 19(3), 390-405.

Baaken, T., Korn, J., Ruf, M., & Walkenhorst, D. (2021). Dissecting Deradicalization: Challenges for Theory and Practice in Germany. International Journal of Conflict and Violence (IJCV), 14, 1-18.

Berlingozzi, L. (2022). O sister, where art thou? Assessing the limits of gender mainstreaming in preventing and countering violent extremism in Mali. Critical Studies on Terrorism, 15(3), 659-680.

Bilazarian, T. (2016). Countering Violent Extremism: Lessons on Early Intervention from the United Kingdom’s Channel Program. The Program on Extremism at George Washington University.

Bintarsari, N. K. (2022). Countering terrorism in Indonesia: a study of policy in counter-terrorism measures of the Indonesia National Counter-terrorism Agency (Badan Nasional Penanggulangan Terorisme/BNPT), PhD Thesis, Rutgers.

Bowei, B. (2020). Evaluating the effects of counterterrorism strategies on insurgency in Nigeria. PhD Thesis: Walden University.

Brady, E. (2021). Assessing Counter-Terrorism Strategies through a Mixed Methods Research Design: The Case of CONTEST in the UK. PhD Thesis, University of St Andrews.

Brown, K. E., & Mohamed, F. N. (2023). Logics of care and control: governing European “returnees” from Iraq and Syria. In A.-K. Rothermel & L.J. Shepherd (Eds.). Gender and the Governance of Terrorism and Violent Extremism (pp. 110-136). Routledge.

Cherney, A., & Belton, E. (2023). The evaluation of case-managed programs targeting individuals at risk of radicalisation. Terrorism and Political Violence, 35(4), 846-865.

Cherney, A., Templar, A., & Koehler, D. (2022). Disguised compliance within CVE programs–how can deception and disguised compliance within CVE programs be understood, recognised and addressed? AVERT.

Christensen, T. W. (2015). A Question of Participation: Disengagement from the Extreme Right. A case study from Sweden. PhD Thesis, Roskilde University.

Christensen, T. W. (2020). Civil Actors' Role in Deradicalisation and Disengagement Initiatives: When Trust is Essential. In Hansen, S. J. & Lid, S. (Eds.). Routledge Handbook of Deradicalisation and Disengagement (pp. 143-155). Routledge.

Costa, V., Liberado, P., Esgalhado, G., Cunha, A. I., & das Neves, P. (2021). One size does not fit all: Exploring the characteristics of exit programmes in Europe. Journal for Deradicalization (28), 1-38.

Davey, J., Birdwell, J., & Skellett, R. (2018). Counter conversations: A model for direct engagement with individuals showing signs of radicalisation online. Institute for Strategic Dialogue.

Davey, J., Tuck, H., & Amarasingam, A. (2019). An imprecise science: Assessing interventions for the prevention, disengagement and de-radicalisation of left and right-wing extremists. Institute for Strategic Dialogue.

Eijkman, Q., & Roodnat, J. (2017). Beware of branding someone a terrorist: Local professionals on person-specific interventions to counter extremism. Journal for Deradicalization (10), 175-202.

Eike, B., von Lautz, Y., Kart, M., & Stein, M. Gender Constructions in the Prevention of and Deradicalization from Islamism in Germany. Journal for Deradicalization (37), 140-173.

El-Said, H., & Harrigan, J. (2013). Deradicalising violent extremists: Counterradicalisation and deradicalisation programmes and their impact in Muslim majority states. Routledge.

Ellefsen, R., Jämte, J., & Sjøen, M. M. (2023). Key dilemmas in the prevention of radicalization and violent extremism. Journal for Deradicalization (34), 115-144.

Ellis, B. H. (2024). Multidisciplinary Threat Assessment and Management Teams in Practice: Common Elements and Operations of Community Based MTAMTs.

Ellis, B. H. (2024). Understanding the Potential for Multidisciplinary Threat Assessment and Management Teams to Prevent Terrorism: Conducting a Formative Evaluation of the MassBay Threat Assessment Team.

Elshimi, M. S. (2017). De-radicalisation in the UK prevent strategy: Security, identity and religion. Routledge.

Frenett, R., & Dow, M. (2015). One to one online interventions: A pilot CVE methodology. Institute for Strategic Dialogue.

Gruber, B. (2023). Governing vulnerability through case management: from crime to radicalisation prevention in the Netherlands. In C. Heath-Kelly & B. Gruber (Eds.). Vulnerability (pp. 125-145). Manchester University Press.

Gruber, B. (2023). Resilience in radicalization prevention: A study about resilience as a norm in primary and secondary prevention in Germany and the Netherlands. PhD Thesis, University of Groningen.

Gruber, B. (2024). Affective discipline – resilience in radicalisation prevention. Critical Studies on Terrorism, 17(3), 786-808.

Haid, H. (2018). Reintegrating ISIS Supporters in Syria: Efforts, Priorities and Challenges. ICSR.

Hardyns, W., Klima, N., & Pauwels, L. (2022). Evaluation and mentoring of the multi-agency approach to violent radicalisation in Belgium, the Netherlands and Germany (Vol. 4). Maklu.

Hardyns, W., Thys, J., Dorme, L., Klima, N., & Pauwels, L. (2021). Multi-agency working to prevent violent radicalisation. Radices, 1(1), 22-40.

Harris-Hogan, S. (2020). How to evaluate a program working with terrorists? Understanding Australia’s countering violent extremism early intervention program. Journal of Policing, Intelligence and Counter Terrorism, 15(2), 97-116.

Haugstvedt, H. (2021). Managing the tension between trust and security: A qualitative study of Norwegian social workers’ experience with preventing radicalisation and violent extremism. PhD Thesis: University of Stavanger.

Hecker, M. (2021). ONCE A JIHADIST, ALWAYS A JIHADIST? A Deradicalization Program Seen from the Inside. IPRI.

Hussain, A., Pilkington, H., Simcock, K., Nicholas, J., Vickers, H., & Rogerson, L. (2021). Talking Our Way Out of Conflict: Critical Reflections on ‘Mediated Dialogue’ as a Tool for Secondary Level CVE.

Ishomuddin, I., Fauzi, I. A., Arifin, S., & Haris, A. (2021). Deradicalization of former terrorists in East Java, Indonesia: the commitment to Islamic religious education. Religación: Revista de Ciencias Sociales y Humanidades, 6(30), 1.

Jackson, B., Rhoades, A., Reimer, J., Lander, N., Costello, K., & Beaghley, S. (2019). Practical Terrorism Prevention: Reexamining U.S. National Approaches to Addressing the Threat of Ideologically Motivated Violence. RAND Corporation.

Jämte, J., & Ellefsen, R. (2020). Countering extremism (s): Differences in local prevention of left-wing, right-wing and Islamist extremism. Journal for Deradicalization (24), 191-231.

Jones, M. (2020). A Template for the Global South? Understanding the Promises and Pitfalls of Preventing/Countering Violent Extremism in Kenya. RUSI.

Klima, N. (2022). Local Multi-Agency Working to Prevent Violent Extremism. RADIS Conference

Koehler, D., & Klosinski, C. (2024). How to Make Sense of it All: A Feasibility Study of Meaning-Centered Interventions in Deradicalization Counseling. Terrorism and Political Violence, 1-16.

Koehler, D., Cherney, A., & Templar, A. (2023). Truth or dare? Exploring the importance of factual accuracy in different deradicalization counseling approaches. Studies in Conflict & Terrorism, 1-21.

Ladbury, S. (2015). Women and Extremism: The Association of Women and Girls with Jihadi Groups and Implications for Programming. Department for International Development.

Lid, S., & Christensen, T. W. (2023). Risk assessment and reintegration of radicalised individuals in the Nordic countries. Norwegian Institute for Urban and Regional Research (NIBR).

Madriaza, P. (2023). Framing local collaboration among frontline workers in the field of preventing violent extremism. Journal for Deradicalization (35), 107-137.

Madriaza, P., Ponsot, A-S., Marion, D. & Monnier, C. (2017). The Prevention of Radicalization Leading to Violence: An International Study of Front-Line Workers and Intervention Issues.

Maiberg, H. (2019). Overview of Methods Supporting the De-radicalisation and Disengagement of Islamic Radicals. Sõjateadlane(13), 149-179.

Miller, E., & Toliver, J. (2016). Promising Practices for Using Community Policing Strategies to Prevent Violent Extremism: How to Create and Implement a Community Outreach Program.

Moonshot. (2020). From passive search to active conversation: An evaluation of the Facebook Redirect Programme: Overview of the pilot programme and recommendations for future deployments.

Moum Hellevik, P., J. Andersen, A., & Engh Førde, K. (2024). Policing mental health? Norwegian police’s work with preventing radicalization into violent extremism. Behavioral Sciences of Terrorism and Political Aggression, 16(2), 248-262.

Mykkanen, T. (2022). Intersubjective body mapping for reintegration: Assessing an art-based methodology to promote reintegration of foreign terrorist fighters. Critical Studies on Terrorism, 15(4), 988-1022.

Ngachra, M. O., & Kimokoti, S. (2023). Effectiveness of Non-Custodial Reintegration Programmes on Welfare of Returnee Terrorist Fighters in Mombasa, Kenya.

Njoku, E. T. (2021). Strategic exclusion: The state and the framing of a service delivery role for civil society organizations in the context of counterterrorism in Nigeria. Studies in Conflict & Terrorism, 44(5), 410-430.

Okonofua, B. A. (2016). The Niger Delta Amnesty Program: The Challenges of Transitioning From Peace Settlements to Long-Term Peace. SAGE Open, 6(2), 16.

Overdulve, C., Duits, N., & Kempes, M.(2022). Evaluating Risk Analysis in Practice: User Validation of the VERA-2R.

Paat, Y.-F., Torres-Hostos, L. R., Garcia Tovar, D., Camacho, E., Zamora, H., Jr., & Myers, N. W. (2023). An integrated ecological approach to countering targeted violence on the U.S.-Mexico border: Insights and lessons learned. Journal of Prevention & Intervention in the Community, 51(4), 375-395.

Papp, S. Z., Örell, R., Meredith, K., Papatheodorou, K., Tadjbakhsh, S., & Brecht H. (2022). The role of civil society organisations in exit work. European Commission.

Persson, E. (2019). A collaborative approach between street-level bureaucrats in the preventive work against violent extremism-a successful way of working: A comparative case study of the preventive work against violent extremism conducted in five Swedish municipalities.

Pettinger, T. (2020). British terrorism preemption: Subjectivity and disjuncture in Channel “de‐radicalization” interventions. The British Journal of Sociology, 71(5), 970-984.

Pettinger, T. (2020). CTS and normativity: the essentials of preemptive counter-terrorism interventions. Critical Studies on Terrorism, 13(1), 118-141.

Piltch-Loeb, R., McBride, M. K., Ekström, A., Hadji-Janev, M., Nielsen, R., Legault, R., Harriman, N. W., & Savoia, E. (2021). The use of a scenario-based nominal group technique to assess P/CVE programs: Development and pilot testing of a toolkit. Journal for Deradicalization (28), 108-140.

Ponsot, A. S., Autixier, C., & Madriaza, P. (2018). Factors facilitating the successful implementation of a prevention of violent radicalization intervention as identified by front-line practitioners. Journal for Deradicalization (16), 1-33.

R.T.I International (2020). Countering Violent Extremism: Diversion Program—Assessment Tools to Support Secondary and Tertiary Intervention for Violent Extremism: Final Report.

R.T.I International (2023). Review of Prevention Programming Undertaken by Allies Abroad to Identify Promising Practices Report.

Raets, S. (2022). Trial and terror: Countering violent extremism and promoting disengagement in Belgium. Journal for Deradicalization (30), 223-261.

Roberts, S. (2018). Detecting radicalisation in communities: The role of multi-agency partnership and the power of local information. Proceedings of the 9th International RAIS Conference on Social Sciences and Humanities, 42-57.

Rosen, S. D. (2010). Pathways to prevention? Evaluating the United Kingdom's approach to counter-radicalization. PhD Thesis, Georgetown University.

Rousseau, C., Johnson-Lafleur, J., Ngov, C., La Rochelle, X., Brouillette-Alarie, S., Gignac, M., Veissiere, S., & Crocker, A. (2024). Risk assessment challenges in a specialized clinic for individuals referred for violent extremism. Journal of Threat Assessment and Management, 11(2), 67-82.

Rousseau, C., Johnson-Lafleur, J., Ngov, C., Miconi, D., Mittermaier, S., Bonnel, A., Savard, C., & Veissière, S. (2023). Social and individual grievances and attraction to extremist ideologies in individuals with autism: Insights from a clinical sample. Research in Autism Spectrum Disorders, 105.

RTI International (2023). Department of Homeland Security (DHS) FY2021 Targeted Violence and Terrorism Prevention (TVTP) Grantee Evaluation Site Profile. Palm Beach County Sheriff’s Office (PBSO).

RTI International (2023). FY2020 Targeted Violence and Terrorism Prevention Grant Evaluations.

Russo, A., & Selenica, E. (2022). Radicalisation, counter-radicalisation and countering violent extremism in the Western Balkans and the South Caucasus: the cases of Kosovo and Georgia. Critical Studies on Terrorism, 15(4), 963-987.

Savage, S., Khan, A., & Liht, J. (2014). Preventing violent extremism in Kenya through value complexity: Assessment of being Kenyan being Muslim. Journal of Strategic Security, 7(3), 1-26.

Schulten, N. (2022). Practitioners’ perspectives on the challenges of dealing with the interaction between mental illness and violent extremism in Countering Violent Extremism (CVE). Behavioral Sciences of Terrorism and Political Aggression, 1-26.

Schuurman, B., & Bakker, E. (2016). Reintegrating jihadist extremists: Evaluating a Dutch initiative. Behavioral Sciences of Terrorism and Political Aggression, 8(1), 66-85.

Sizoo, B., Doosje, B., & van Meijel, B. (2023). Perceptions of radicalisation in mental health care and the security domain: roles, responsibilities, and collaboration. Psychology, Crime & Law, 29(5), 548-565.

Solhjell, R., Sivenbring, J., Kangasniemi, M., Kallio, H., Christensen, T. W., Haugstvedt, H., & Gjelsvik, I. M. (2022). Experiencing trust in multiagency collaboration to prevent violent extremism: A Nordic qualitative study. (32).

Spalek, B. (2013). Terror crime prevention with communities. Bloomsbury Academic.

Spalek, B., & Davies, L. (2012). Mentoring in Relation to Violent Extremism: A Study of Role, Purpose, and Outcomes. Studies in Conflict & Terrorism, 35(5), 354-368.

Spalek, B., Davies, L., & McDonald, L. Z. (2010). Key Evaluation Findings of the West Midlands 1-2-1 Mentoring Project. University of Birmingham.

Stahl, G., Adams, B., & Oberg, G. “If you don’t have rapport, you haven’t got anything really”: Affective intensities, relationality and rapport building in the work of CVE Intervention Practitioners. Dynamics of Asymmetric Conflict, 1-18.

Sukabdi, Z. A. (2015). Terrorism in Indonesia: A Review on Rehabilitation and Deradicalization. Journal of Terrorism Research, 6(2).

Thompson, S. K., & Leroux, E. (2023). Lessons learned from dual site formative evaluations of Countering violent extremism (CVE) programming co-led by Canadian police. Journal of Policing, Intelligence and Counter Terrorism, 18(1), 24-42.

Tropp, L. R., Bilali, R., & Flickinger, S. (2019). Healing Our Communities: Promoting Social Cohesion in Rwanda. USAID, Karuna Center for Peacebuilding, AEGIS Preventing Crimes Against Humanity.

Van der Heide, L., & Schuurman, B. (2018). Reintegrating terrorists in the Netherlands: Evaluating the Dutch approach. Journal for Deradicalization (17), 196-239.

Vittum, K., Ombok, O., Odary, K., & Mmoji, G. (2016). Evaluation Kenya Tuna Uwezo: Final Performance Evaluation USAID/Kenya and East Africa Office of Democracy. USAID

von Lautz, Y., Bösing, E., Kart, M., & Stein, M. (2023). Counseling towards the deradicalization of Islamist extremists: An overview of approaches based on two qualitative interview studies with prevention practitioners in Germany. Journal for Deradicalization (36), 23-63.

von Lautz, Y., Bösing, E., Kart, M., & Stein, M. (2024). Influences of Discrimination and Stigmatization on Secondary and Tertiary Level P/CVE Efforts–Insights from German Practitioners into Countering Islamist Extremism. Journal for Deradicalization (38), 122-164.

Wdzięczak, H. (2022). Gender mainstreaming in Preventing and Countering Violent Extremism. Journal for Deradicalization (33), 70-107.

Weeks, D. (2018). Doing derad: An analysis of the UK system. Studies in Conflict & Terrorism, 41(7), 523-540.

White, J. (2022). Finding the right mix: re-evaluating the road to gender-equality in countering violent extremism programming. Critical Studies on Terrorism, 15(3), 585-609.

**Exclusion: Intervention I - Does not examine intervention**

Abbas, M-S. (2019). ‘I grew a beard and my dad flipped out!’ Co-option of British Muslim parents in countering ‘extremism’ within their families in Bradford and Leeds. Journal of Ethnic and Migration Studies, 45(9), 1458-1476

Abbas, M-S. (2019). Producing 'internal suspect bodies': Divisive effects of UK counter-terrorism measures on Muslim communities in Leeds and Bradford. British Journal of Sociology, 70(1), 261-282

Abbas, T., & Siddique, A. (2012). Perceptions of the processes of radicalisation and de-radicalisation among British South Asian Muslims in a post-industrial city. Social Identities: Journal for the Study of Race, Nation and Culture, 18(1), 119-134

Abbas, T., Awan, I., & Marsden, J. (2023). "Pushed to the Edge": The Consequences of the 'Prevent Duty' in De-Radicalising Pre-Crime Thought among British Muslim University Students. Race, Ethnicity and Education, 26(6), 719-734.

Abdelgwad, M. A. Z. (2023). The Role of Saudi Universities in Encountering Electronic Extremism among University Youth from the Faculty Members' And Students' Points of View. Conhecimento & Diversidade, 15(36), 501-524.

Acik, N., & Pilkington, H. (2018). Youth mobilisations of ‘suspect communities’ UK.

Acik, N., Deakin, J., & Hindle, R. (2018). Safeguarding, Surveillance and Control: School Policy and Practice Responses to the Prevent Duty and the “war on terror” in the UK. In J. Deakin, E. Taylor & A. Kupchik (Eds.). The Palgrave International Handbook of School Discipline, Surveillance, and Social Control (pp. 467-489). Palgrave Macmillan

Afrianty, D. (2012). Islamic education and youth extremism in Indonesia. Journal of Policing, Intelligence and Counter Terrorism, 7(2), 134-146.

Agius, C., Edney-Browne, A., Nicholas, L., & Cook, K. (2022). Anti-feminism, gender and the far-right gap in C/PVE measures. Critical Studies on Terrorism, 15(3), 681-705

Ahdash, F. (2024). The familialization of terrorism and the securitization of the family: gendered narratives of infantilization and demonization. Journal of Law and Society, 51(2), 212-238.

Ahmad, H. (2016). Youth De-radicalization: Best Practices for Canada. Masters' Thesis, Royal Roads University Victoria.

Ahmad, H. H. (2017). Youth de-radicalization: A Canadian framework. Journal for Deradicalization (12), 119-168.

Ahmed, Z. S. (2018). A Critique of the Need and Application of Peace Education in Pakistan. Asian Journal of Peacebuilding, 6(1), 99-112.

Ajah, B. O., Ernest, D. C., & Salami, K. K. (2020). Terrorism in Contemporary Nigerian Society: Conquest of Boko-haram, Myth or Reality. International Journal of Criminal Justice Sciences, 15(2), 312-324.

Ajala, I. (2019). Tunisian terrorist fighters: a grassroots perspective. Behavioral Sciences of Terrorism & Political Aggression, 11(2), 178-190.

Al-Badayneh, D. M., Al-Assasfeh, R. A., & Ekici, S. (2023). Developing A Robust Legal System Through Scale for Youth Extremism Across Arab Cultures. International Journal of Criminal Justice Sciences, 18(1), 29-51.

Al-Badayneh, D. M., Al-Tarawneh, M. E., Lamchichi, A. A., & Ellamey, Y. M. (2024). Religious observance and youth violent extremism among young Arab students: Implications for youth religious de-radicalization. European Journal of Science and Theology, 20(1), 1-17.

Al-hammadin, E., Hartley, J., Abdalla, M., & Jones, C. (2023). Trust-building in countering violent extremism programs: a Muslim youth perspective. Journal of Policing, Intelligence and Counter Terrorism.

Aldrich, D. P., & Mahabir, R. (2023). Countering violent extremism in Trinidad and Tobago: An evaluation. Terrorism and Political Violence, 35(7), 1486-1501.

Alghofaili, F. (2021). A comparative analysis of British and Saudi approaches to counter radicalisation: Engaging the Muslim community as a model. PhD Thesis, University of Birmingham.

Ali, R. B., Moss, M., Barrelle, S. A., & Lentini, K. (2017). Initiatives that Counter Violent Radicalization but are Perceived as Suitable by Targeted Communities Journal of Police and Criminal Psychology. Communities Journal of Police and Criminal Psychology, 32(1), 43-55.

Allely, C., & Faccini, L. (2019). Clinical profile, risk, and critical factors and the application of the "path toward intended violence" model in the case of mass shooter Dylann Roof. Deviant Behavior, 40(6), 672-689.

Allen, C. (2022). The neoliberalisation of formal governmental relations with Britain’s Muslim communities. British Politics, 17(2), 232-249.

Alonso, R., & Delgado, P. (2023). The radicalization of young jihadi convicted of membership of terrorist organization in Spain: Analyzing the pieces of the puzzle. Studies in Conflict & Terrorism, 46(5), 594-617.

Alyousef, Y., & Zanuddin, H. (2018). Saudi Arabian government crisis management and prevention strategies: Has it been effective to curb the presence of radical groups in the social media? International Journal of Engineering and Technology(UAE), 7(2.29 Special Issue 29), 633-638.

Arifi, B. (2019). Drivers to Violent Extremism in South Eastern Europe - the North Macedonian Context. Journal of Penal Law and Criminology-Ceza Hukuku Ve Kriminoloji Dergisi, 7(1), 27-51.

Aslam, T., Rehman, A. U., & Ullah, F. (2023). Determinants of Radicalization and Militancy amongst the Youth in Pakistan. Pakistan Journal of Criminology, 15(1), 156-173.

Awan, I., & Guru, S. (2017). Parents of foreign "terrorist" fighters in Syria-Will they report their young? Ethnic and Racial Studies, 40(1), 24-42.

Ayad, M., & Amarasingam, A. (2019). No Platform for Old Men Barriers to Online Youth Civic Engagement and P-CVE in Europe. Institute for Strategic Dialogue.

Ayima, K. (2019). Counter-Ideology as a Wider Strategy for Defeating the Boko Haram Terrorist Group. PhD Thesis, Walden University.

Baak, M., Stahl, G., Schulz, S., & Adams, B. (2022). 'We Have to Be Really Careful': Policy Intermediaries Preventing Violent Extremism in an Era of Risk. Journal of Education Policy, 37(3), 461-481.

Bailey, G. (2012). Community, politics and extremism: a study of far-right and radical Islamist engagement with wider society. PhD Thesis, Keele University.

Baldino, D., & Lucas, K. (2019). Anti-government rage: understanding, identifying and responding to the sovereign citizen movement in Australia. Journal of Policing, Intelligence and Counter Terrorism, 14(3), 245-261.

Bangura, I., Owusu, A., & Quaye, A. S. (2024). When waiting means trouble: an enquiry into the approach toward prevention against jihadism in Senegal. Frontiers in Political Science, 6.

Bastani, N., & Gazzotti, L. (2022). “Still a bit uncomfortable, to be an arm of the state”: Making sense and subjects of counter-extremism in the UK and Morocco. Environment and Planning C: Politics and Space, 40(2), 520-540.

Baykal, A., Bressan, S., Friedrich, J., Pasquali, G., Rotmann, P., & Wagner, M. (2021). Evaluating P/CVE: Institutional structures in international comparison. Global Public Policy Institute.

Beaghley, S., Helmus, T. C., Matthews, M., Ramchand, R., Stebbins, D., Kadlec, A., & Brown, M. A. (2017). Development and Pilot Test of the RAND Program Evaluation Toolkit for Countering Violent Extremism. RAND Corporation.

Beighton, C., & Revell, L. (2020). Implementing the 'Prevent Duty' n England: The semiotisation of discourse and practice in further education. Discourse-Studies in the Cultural Politics of Education, 41(4), 516-531.

Belov, V., Trofimovich, B. L., Fedorovna, G. N., Vitalievich, Z. K., Alekseevich, R. A., Alekseevich, T. V., Alexandrovna, Y. V., & Alexandrovich, S. Y. (2020). Consistency of personal identity as a resource of pedagogical prevention of adolescents' radicalization. Revista San Gregorio(42), 275-281.

Benjamin, S., Koirikivi, P., Salonen, V., Gearon, L., & Kuusisto, A. (2023). Safeguarding social justice and equality: Exploring Finnish youths' 'Intergroup Mindsets' as a novel approach in the prevention of radicalization and extremism through education. Education Citizenship and Social Justice, 21.

Benjamin, S., Salonen, V., Gearon, L., Koirikiv, P., & Kuusisto, A. (2021). Safe space, dangerous territory: Young people's views on preventing radicalization through education-Perspectives for pre-service teacher education. Education Sciences, 11(5).

Beršnak, J. V., & Prezelj, I. (2021). Recognizing youth radicalization in schools: Slovenian ‘frontline’ school workers in search of a compass. International Sociology, 36(1), 49-70.

Bjorgo, T. (2005). Conflict Processes between Youth Groups in a Norwegian City: Polarisation and Revenge. European Journal of Crime, Criminal Law and Justice, 13(1), 44-74.

Bjornsgaard, K. T. H., & Moeyens, C. (2019). The Many States of Activism: The Global YouthCAN Activism Survey. Institute for Strategic Dialogue.

Bouzar, D. (2018). D3.3 – Stages of the radicalization and deradicalization process. Research and Innovation Action (RIA) Partnership against violent radicalization in cities. Project Number: 740072

Bowie, R. A., & Revell, L. (2018). How Christian Universities Respond to Extremism. Education Sciences, 8(3), 14.

Bragin, M. (2021). The paradox of hope: A psychodynamic approach to understanding the motivations of young people engaged in violent extremism. Journal of Infant, Child & Adolescent Psychotherapy, 20(4), 411-424.

Breidlid, T. (2021). Countering or contributing to radicalisation and violent extremism in Kenya? A critical case study. Critical Studies on Terrorism, 14(2), 225-246.

Bressan, S., Ebbecke, S. & Rahlf, L. (2024). How Do We Know What Works in Preventing Violent Extremism? Evidence and Trends in Evaluation from 14 Countries. GPPI.

Briggs, R., Fieschi, C., & Lownsbrough, H. (2006). Bringing it Home. Community-based approaches to counter-terrorism. Demos.

Bronsard, G., Cohen, D., Diallo, I., Pellerin, H., Varnoux, A., Podlipski, M. A., Gerardin, P., Boyer, L., & Campelo, N. (2022). Adolescents Engaged in Radicalisation and Terrorism: A Dimensional and Categorical Assessment. Frontiers in Psychiatry, 12(774063).

Brown, R. A., Weilant, S., Ramchand, R., Hiatt, L., Rhoades, A. L., Palimaru, A. I., & Helmus, T. C. (2021). Violent extremism in America: Interviews with former extremists and their families on radicalization and deradicalization. RAND Corporation.

Bryan, H. (2017). Developing the political citizen: How teachers are navigating the statutory demands of the Counter-Terrorism and Security Act 205 and the Prevent Duty. Education, Citizenship and Social Justice, 12(3), 213-226.

Bull, M., & Rane, H. (2019). Beyond faith: Social marginalisation and the prevention of radicalisation among young Muslim Australians. Critical Studies on Terrorism, 12(2), 273-297.

Busher, J., & Jerome, L. (2020). The Prevent Duty in Education: Impact, Enactment and Implications. Palgrave.

Busher, J., Choudhury, T., Thomas, P., & Harris, G. (2017). What the Prevent Duty means for schools and colleges in England: An analysis of educationalists' experiences. Aziz Foundation.

Campelo, N., Bouzar, L., Oppetit, A., Pellerin, H., Hefez, S., Bronsard, G., Cohen, D., & Bouzar, D. (2018). Joining the Islamic State from France between 2014 and 2016: An observational follow-up study. Palgrave Communications, 4(1)

Campelo, N., Oppetit, A., Thompson, C., Cohen, D., & Louet, E. (2022). A Clinical and Psychopathological Approach to Radicalization Among Adolescents. Frontiers in Psychiatry, 13, 788154.

Capone, F. (2017). 'Worse' than Child Soldiers? A Critical Analysis of Foreign Children in the Ranks of ISIL. International Criminal Law Review, 17(1), 161-185.

Carroll II, D. W. (2022). Leaving Hate: Social Work and the Journey Out of Far-Right Extremism. Indiana University-Purdue University Indianapolis.

Catherine, B., & Louis, B. (2023). A Path of Radicalization: Complementary of the Group and the Individual Flaw. Journal of Forensic Psychology Research & Practice, 23(2), 178-200.

Chivers, C. (2018). ‘What is the headspace they are in when they are making those referrals?’ Exploring the lifeworlds and experiences of health and social care practitioners undertaking risk work within the Prevent Strategy. Health, Risk & Society, 20(1-2), 81-103.

Choudhury, T. (2020). Campaigning on campus: Student Islamic societies and counterterrorism. In B. Spalek & D. Weeks (Eds.). Communities and Counterterrorism (pp. 18-36). Routledge.

Christodoulou, E. (2020). ‘Boosting resilience’ and ‘safeguarding youngsters at risk’: Critically examining the European Commission’s educational responses to radicalization and violent extremism. London Review of Education, 18(1), 18-34.

Chuang, Y.-L., Chou, T., & D'Orsogna, M. R. (2018). Age-structured social interactions enhance radicalization. The Journal of Mathematical Sociology, 42(3), 128-151.

Clément, P.-A., Madriaza, P., & Morin, D. (2021). Constraints and opportunities in evaluating programs for prevention of violent extremism: how the practitioners see it. UNESCO Chair in Prevention of Radicalisation and Violent Extremism (UNESCO).

Danvers, E. (2023). Prevent/Ing critical thinking? The pedagogical impacts of Prevent in UK higher education. Teaching in Higher Education, 28(6), 1264-1279.

de Divitiis, V. (2011). Considerations over Factors Empowering Radicalization in the European Union. In G. M. Ruggiero (Ed.). Perspectives on Immigration and Terrorism (pp. 51-61). IOS Press.

de Montclos, M.-A. P. (2018). ‘The only good jihadist is a dead jihadist’: Boko Haram and de-radicalization around Lake Chad. Small Wars & Insurgencies, 29(5-6), 863-885.

Decker, S. H., & Pyrooz, D. C. (2020). The imprisonment-extremism nexus: Continuity and change in activism and radicalism intentions in a longitudinal study of prisoner reentry. PLoS ONE, 15(11), e0242910.

DeMichele, M., Simi, P., & Blee, K. (2024). Becoming an Ex-Extremist: Stopping the Hate and Embracing a New Identity. British Journal of Criminology, 16.

Duits, N., Alberda, D. L., & Kempes, M. (2022). Psychopathology of Young Terrorist Offenders, and the Interaction With Ideology and Grievances. Frontiers in Psychiatry, 13, 801751.

Durodie, B. (2016). Securitising Education to Prevent Terrorism or Losing Direction? British Journal of Educational Studies. 64(1), 21-35.

Durrani, H. (2020). Critical Approach to Pakistan’s Counter-terrorism Legislative Framework. McGill University.

E, Varsha & Sahay, M. (2023). Juvenile Terrorism. Medico-Legal Update, 23, 36-40.

Edwards, P. (2016). Closure through resilience: The case of Prevent. Studies in Conflict & Terrorism, 39(4), 292-307.

Edwards, P. (2021). Surveillance, safeguarding and beyond: The Prevent Duty and resilient citizenship. Critical Studies on Terrorism, 14(1), 47-66.

Eklund Wimelius, M., Eriksson, M., Strandh, V., & Ghazinour, M. (2020). "They think of us as part of the problem instead of part of the solution": Swedish civil society and faith based organizations in resilience building and prevention of radicalization and violent Islamist extremism. Journal for Deradicalization (22), 122-154.

Eliseev, S., Vicentiy, I., & Gluchich, V. (2017). Youth political extremism: Methods of early warning. Teorija in Praksa, 54(6), 990-1007.

Farhadi, A. (2022). Countering Violent Extremism in Central Asia and South Asia: Islamophobia and Cyber-Radicalization in the Digital Era. In A. Farhadi, R. P. Sanders. & A. Masys (Eds.). The Great Power Competition Volume 3: Cyberspace: The Fifth Domain (pp. 83-97). Springer.

Fatykhova, R. M., & Mingazova, D. V. (2015). Diagnostics of teenagers’ disposition towards destructive communication as a way of youth extremism prevention. New Educational Review, 42(4), 102-111.

Feddes, A. R. (2015). Socio-psychological factors involved in measures of disengagement and deradicalization and evaluation challenges in Western Europe. Middle East Institute.

Feddes, A. R., Mann, L., & Doosje, B. (2013). Scientific Approach to Formulate Indicators & Responses to Radicalisation. Empirical study. SAFIRE.

Fergusson, J., Ahmed, K. (2017). Canada’s National Counterterrorism Strategy and Challenges of Community Based Intervention in Countering Radicalization. In S. Romaniuk, F. Grice, D. Irrera & S. Webb (Eds.) The Palgrave Handbook of Global Counterterrorism Policy. Palgrave Macmillan, London.

Finn, M., Momani, B., Opatowski, M., & Opondo, M. (2016). Youth evaluations of CVE/PVE programming in Kenya in context. Journal for Deradicalization (7), 164-224.

Franco, J. (2018). Preventing Other ‘Marawis’ in the Southern Philippines. Asia & the Pacific Policy Studies, 5(2), 362-369.

Gavrielides, T. (2020). Violent youth radicalisation in Europe: The youth-led model. Internet Journal of Restorative Justice (IJRJ), 9-32.

Gearson, J., & Rosemont, H. (2015). CONTEST as Strategy: Reassessing Britain's Counterterrorism Approach. Studies in Conflict & Terrorism, 38(12), 1038-1064.

Gerrand, V. (2023). Young people, radicalisation, and resilience. In R. McNeil-Willson. & A. Triandafyllidou (Eds.). Routledge Handbook of Violent Extremism and Resilience (pp. 71-91), Routledge.

Gheorghe, R. M., & Clement, D. (2023). 'It's time to put the copes down and get to work': A qualitative study of incel exit strategies on r/IncelExit. Behavioral Sciences of Terrorism and Political Aggression, 21.

Glazzard, A., & Reed, A. (2018). Global Evaluation of the European Union Engagement on Counter-Terrorism. ICCT.

Greater Manchester Preventing Hateful Extremism and Promoting Social Cohesion Commission (2018). A Shared Future.

Green, H. L. (2018). Vulnerabilities to Engagement in Violent Extremism. PhD Thesis, University of Birmingham

Grimbergen, C., & Fassaert, T. (2022). Occurrence of Psychiatric Disorders, Self-Sufficiency Problems and Adverse Childhood Experiences in a Population Suspected of Violent Extremism. Frontiers in psychiatry Frontiers Research Foundation, 13, 779714.

Grossman, M. (2017). Understanding Youth Resilience to Violent Extremism: A Standardised Research Measure: Final Research Report.

Grossman, M., Hadfield, K., Jefferies, P., Gerrand, V., & Ungar, M. (2022). Youth resilience to violent extremism: Development and validation of the BRAVE measure. Terrorism and Political Violence, 34(3), 468-488.

Guidi, E., & Babetto, C. (2020). First steps: Reaching consensus on understanding violent radicalisation and utilising participatory approaches for prevention. In P. Meringolo (Eds.). Preventing Violent Radicalisation in Europe: Multidisciplinary Perspectives, (43-56). Springer.

Hand, M. (2023). Education, Extremism, and Aversion to Compromise. Educational Theory, 73(3), 341-354.

Haringová, I. (2018). Governmental Approaches Towards Countering Violent Extremism and Radicalisation: A Comparative Analysis of the United Kingdom and the Kingdom of Denmark. Charles University

Harris-Hogan, S., Barrelle, K., & Zammit, A. (2016). What is countering violent extremism? Exploring CVE policy and practice in Australia. Behavioral Sciences of Terrorism and Political Aggression, 8(1), 6-24.

Hart, S. D., Cook, A. N., Pressman, D. E., Strang, S., & Lim, Y. L. (2017). A concurrent evaluation of threat assessment tools for the individual assessment of terrorism. Canadian Network for Research on Terrorism, Security, and Society (TSAS).

Hashom, H. B., & Azmin, A. J. (2019). The ethics of Islamic entrepreneurship in preventing radicalism among Malaysian graduates. M. M. Aslam, & R. Gunaratna (Eds.).Terrorist Rehabilitation and Community Engagement in Malaysia and Southeast Asia (pp. 112-120). Routledge.

Haugstvedt, H., & Sjøen, M. M. (2021). Exploring Youths’ Willingness to Engage with Civil Society and Public Sector Institutions: The Untapped Potential of Religious Communities in Preventing Violent Extremism. Democracy and Security, 17(4), 337-355.

Haynes, C. (2015). Countering extremism: An understanding of the problem, the process and some solutions. Naval Postgraduate School.

Heath-Kelly, C. (2024). Devolution and the prevent strategy in Scotland: Constitutional politics and the path of Scottish P/CVE. Parliamentary Affairs, 77(2), 350-370.

Heath-Kelly, C., & Strausz, E. (2019). The banality of counterterrorism "after, after 9/11"? Perspectives on the Prevent duty from the UK health care sector. Critical Studies on Terrorism, 12(1), 89-109.

Hedayah. (2020). Blueprint of a Rehabilitation and Reintegration Center: Guiding Principles For Rehabilitating and Reintegrating Returning Foreign Terrorist Fighters and their Family Members.

HEFCE (2017). Implementation of the Prevent Duty in the Higher Education Sector in England: 2015-16. Monitoring Outcomes. January 2017/01.

Hendri, N., Qudsy, S., & Elvira, M. (2024). Resistance of Islamist Groups to Government Policies in the Settlement of Religious Radicalism. International Journal of Islamic Thought, 25, 109-121.

Henrich, S. (2023). Extremism in secure forensic settings: The assessment of vulnerabilities for radicalisation. PhD Thesis, University of Central Lancashire.

Higton, J., Patel, R., Hansel, M., Choudhoury, A., Francis, N., & Merrett, D. (2021). Prevent Referrals in Higher Education: Approaches and Practices. Research Report. UK Department for Education.

Higton, J., Patel, R., Mulla, I., Francis, N., Choudhoury, A., Wilkinson, B., Baginsky, M., & van Rij, A. (2018). Prevent and Counter-Extremism in General Further Education Colleges. UK Department for Education.

Hill, R. (2019). Counter-Extremism in British Schools: Ensuring Respect for Parents’ Rights Over Their Children’s Religious Upbringing. British Journal of Educational Studies, 67(1), 115-129.

Holdo, M. (2021). An inclusive and participatory approach to counter-radicalization? Examining the role of Muslim associations in the Swedish policy process. Ethnicities, 21(3), 477-497.

Holland, J., & Higham-James, N. (2024). Enactors of the State: The Everyday Coproduction of Security in the Prevention of Radicalisation. Political Studies, 24.

Holmer, G., Bauman, P., & Aryaeinejad, K. (2018). Measuring up: Evaluating the impact of P/CVE programs. USIP.

Horgan, J., & Braddock, K. (2010). Rehabilitating the terrorists?: Challenges in assessing the effectiveness of de-radicalization programs. Terrorism and Political Violence, 22(2), 267-291.

Huesca, E. F., Jr. (2019). On "Youth, Peace, and Security" in Mindanao, Philippines. Peace Review, 31(1), 57.

IAHV (n.d.). BEYOND VIOLENT EXTREMISM & ARMED CONFLICT: IAHV Training and Programs.

Igboin, B. O. (2022). ‘Small Fires Causing Large Fires’: An Analysis of Boko Haram Terrorism–Insurgency in Nigeria. Religions, 13(6).

Ilyas, M., Ismail, Z., Abdullah, M. A., & Zulfidar, F. (2020). YOUTH EXISTENCE AND RADICALISM IN ACEH, INDONESIA. Jurnal Ilmiah Peuradeun, 8(2), 409-422.

Institute for Strategic Dialogue (2022). Baseline Evaluation Report for PROACT – Community-Based Interventions in Kenya Program.

Irwin, N. (2015). The complexity of responding to home-grown terrorism: radicalisation, de-radicalisation and disengagement. Journal of Policing, Intelligence and Counter Terrorism, 10(2), 166-175.

Isakjee, A. (2013). The securitised identities of young Muslim men in Birmingham. PhD Thesis, University of Birmingham.

Itälunni, J. (2018). Development of the RADAR-iTE instruction card for operational and educational purposes.

Jacobson, M. (2009). Terrorist Drop-outs One Way of Promoting a Counter-Narrative. Perspectives on Terrorism, 3(2), 12-17.

Jacoby, T. A. (2016). How the War Was ‘One’: Countering violent extremism and the social dimensions of counter-terrorism in Canada. Journal for Deradicalization (6), 272-304.

Jakob, J. M. (2016). Terror’s Motor: How Shame and Humiliation Turn the Spiral of Violence. Masters' Thesis, Uppsala University.

James, J. S. & Janmaat, J. G. (2019). Civil Disorder, Domestic Terrorism and Education Policy The Context in England and France. Palgrave Macmillan.

James, N. C. (2020). Implementing the Prevent Duty : Conceptualising Threat Within Greater Manchester's Further Education Sector. PhD Thesis, University of Leeds.

Jarvis, L. & Lister, M. (2015). Anti-Terrorism, Citizenship and Security. Manchester University Press.

Jensen, M. & Simi, P. (2021). Empirical Assessment of Domestic Disengagement and Deradicalization (EAD3).

Jensen, M., James, P., & Yates, E. (2023). Contextualizing Disengagement: How Exit Barriers Shape the Pathways Out of Far-Right Extremism in the United States. Studies in Conflict & Terrorism, 46(3), 249-277.

Jones, C. (2019). Effective Community Engagement: Back to the Basics to Counter Violent Extremism and Other Youth Crimes. In S. Jayakumar (Ed.). Terrorism, Radicalisation and Countering Violent Extremism: Practical Considerations and Concerns (pp. 29-41). Springer.

Jones, C. (2022). Hard to reach or don’t want to reach? Understanding the significance of trust and respectful relationships in “countering violent extremism”. Journal of Ethnicity in Criminal Justice, 20(2), 165-189.

Jorgensen, K. E. (2022). "IS Drew This Dream Picture-Like Floating on a Pink Cloud": Danish Returnees' Entry into and Exit from Salafi-Jihadism through Nurtured and Fractured Fantasies. Societies, 12(4), 21.

Joyce, C. A. (2018). Exploring teachers' beliefs, values and attitudes towards radicalisation, extremism and the implementation of anti-radicalisation strategies. PhD Thesis, University of Sheffield.

Kenney, M. (2018). The Islamic State in Britain: Radicalization and Resilience in an Activist Network. Cambridge University Press.

Khalid, I. (2014). Topology of Extremism: Implication on the Contemporary Politics of Pakistan. South Asian Studies, 29(1), 23-39.

Kimmel, M. (2007). Racism as adolescent male rite of passage: Ex-Nazis in Scandinavia. Journal of Contemporary Ethnography, 36(2), 202-218.

King, S. (2022). Reactive Ethnicity and Religious Emotions: Disentangling the Influences of Religousity and Criminogenic Factors on Islamist Radicalization Processes in the West. PhD Thesis, Friedrich-Alexander-Universitaet Erlangen-Nuernberg.

Kislyakov, P., Sergeev, S., Strelkov, V., Belyakova, N., Romanova, A. (2018). PSYCHOLOGISTS AND TEACHERS CONCEPTION OF DESTRUCTIVE IMPACT OF EXTREMIST ORGANIZATIONS ON MINORS. International Conference on Research Paradigms Transformation in Social Sciences (RPTSS), 50, 584-591.

Koehler, D. (2018). Dialogue about Radicalisation and Equality: De-radicalization Programme Integrity Evaluation Checklist (DPIEC). DARE Project.

Koehler, D., & Fiebig, V. (2019). Knowing What to Do: Academic and Practitioner Understanding of How to Counter Violent Radicalization. Perspectives on Terrorism, 13(3), 44-62.

Lafrarchi, N. (2021). Intra-and interreligious dialogue in Flemish (Belgian) secondary education as a tool to prevent radicalisation. Religions, 12(6).

Lakhani, S. (2012). Preventing violent extremism: Perceptions of policy from grassroots and communities. The Howard Journal of Criminal Justice, 51(2), 190-206.

Lakhani, S. (2020). Social capital and the enactment of Prevent Duty: An empirical case-study of schools and colleges. Critical Studies on Terrorism, 13(4), 660-679.

Lakhani, S., & James, N. (2021). “Prevent duty”: Empirical reflections on the challenges of addressing far-right extremism within secondary schools and colleges in the UK. Critical Studies on Terrorism, 14(1), 67-89.

Langer, P. C., & Ahmad, A. N. (2019). Psychosocial Needs of Former ISIS Child Soldiers in Northern Iraq.

Lášticová, B., Hargašová, L., Andraščiková, S., Dráľ, P., & Findor, A. (2018). Perspectives and limits of tolerant intergroup relations formation in civic education practice in Slovakia. Studia Paedagogica, 23(3), 69-90.

Lefas, M. (2023). BLUE SKY VI: An Independent Analysis of UN Counterterrorism Efforts. Global Center on Cooperative Security.

Lewis, J. (2018). Prevent as an intractable policy controversy: Implications and solutions. Journal for Deradicalization (15), 111-150.

Lewis, J. (2020). Enacting the Prevent Duty in Secondary Schools. In J. Busher & L. Jerome (Eds.). The Prevent Duty in Education: Impact, Enactment and Implications (pp. 117-137). Palgrave Macmilan.

Lindekilde, L. (2012). Neo-liberal Governing of "Radicals": Danish Radicalization Prevention Policies and Potential Iatrogenic Effects. International Journal of Conflict and Violence, 6(1), 109-125.

Ljujic, V., van Prooijen, J. W., & Weerman, F. (2017). Beyond the crime-terror nexus: socio-economic status, violent crimes and terrorism. Journal of Criminological Research, Policy and Practice, 3(3), 158-172.

Lobato, R. M., Garcia-Coll, J., Martin-Criado, J. M., & Moyano, M. (2023). Impact of psychological and structural factors on radicalization processes: A multilevel analysis from the 3N model. Psychology of Violence, 13(6), 479-487.

Lobato, R. M., Ruipérez, J., & Marrero, I. (2022). Strategies for Preventing Radicalisation: Insights from the Practitioner Perspective. Journal of Peacebuilding and Development, 17(2), 173-193.

Lochmann, M., & Guedj, M. (2021). Under what conditions do lay people and health professionals accept a breach of doctor-patient confidentiality regarding a patient with signs of terrorist radicalization? European Review of Applied Psychology / Revue Europeenne de Psychologie Appliquee, 71(5), 1-10.

Lub, V. (2013). Radicalisation and Social Policy: Evaluating the Theories of Change. Evidence and policy, 9(2), 165-183.

Lucini, B. (2017). The Other Side of Resilience to Terrorism: A Portrait of a Resilient-Healthy City. Springer

Lundie, D. C. (2019). Building a terrorist house on sand: A critical incident analysis of interprofessionality and the Prevent duty in schools in England. Journal of Beliefs and Values, 40(3), 321-337.

Lundy, P., Gilmartin, N., McDermott, P., Finegan, R., & Murphy, R. (2021). Dealing with the Legacy of Conflict in Northern Ireland through Engagement and Dialogue. Glencree Journal.

Mastroe, C. (2016). Evaluating CVE: Understanding the recent changes to the United Kingdom’s implementation of Prevent. Perspectives on Terrorism, 10(2), 50-60.

Mattson, C., & Johansson, T. (2018). Becoming, belonging and leaving–Exit processes among young neo-Nazis in Sweden. Journal for Deradicalization (16), 33-69.

Mattsson, C. (2018). Caught between the urgent and the comprehensible: Professionals' understanding of violent extremism. Critical Studies on Terrorism, 11(1), 111-129.

Mattsson, C. (2023). The lock pickers, the gatekeepers, and the non-grievables: A case study of youth workers’ roles in preventing violent extremism. Nordic Social Work Research, 13(2), 306-317.

Mattsson, C. (2023). Unintended but not unanticipated consequences: youth work, organized crime, and concealed radicalization. Journal for Deradicalization (36), 1-22.

Mattsson, C., & Johansson, T. (2019). Leaving Hate Behind–Neo-Nazis, Significant Others and Disengagement. Journal for Deradicalization, 18, 185-216.

Mattsson, C., & Johansson, T. (2020). Talk is silver and silence is gold? Assessing the impact of public disengagement from the extreme right on deradicalization. Journal for Deradicalization (24), 79-112.

Mattsson, C., & Johansson, T. (2020). The hateful other: Neo-Nazis in school and teachers’ strategies for handling racism. British Journal of Sociology of Education, 41(8), 1149-1163.

Mattsson, C., & Säljö, R. (2018). Violent Extremism, National Security and Prevention. Institutional Discourses and their Implications for Schooling. British Journal of Educational Studies, 66(1), 109-125.

Merhej, R. (2024). Prejudice in children and extremism in adults: Intriguing mindset convergences. Social Science Information Sur Les Sciences Sociales, 63(1), 47-67.

Michael, G., & Minkenberg, M. (2007). A continuum for responding to the extreme right: A comparison between the United States and Germany. Studies in Conflict & Terrorism, 30(12), 1109-1123.

Missier, C. A. (2022). Fundamentalism and the search for meaning in digital media among Gen y and Gen Z. Journal for Deradicalization, 33, 255-285.

Moeller, M. J., & Scheithauer, H. (2024). Developmental and biographical issues in radicalization pathways: A comparative case analysis of homegrown German convicts of Islamist terrorism-related offenses. Terrorism and Political Violence, 36(2), 214-233.

Mohamed, M. H. (2021). Dangerous or political? Kenyan youth negotiating political agency in the age of ‘new terrorism’. Media, War and Conflict, 14(3), 303-321.

Mohammadi, M. (2021). The Role of Education in Combating Violent Extremism in Developing Countries with an Emphasis on Afghanistan. Shanlax International Journal of Education, 9(3), 273-287.

Mohammed, R., & Neuner, F. (2022). Putative juvenile terrorists: the relationship between multiple traumatization, mental health, and expectations for reintegration among Islamic State recruited adolescent and young adult fighters. Conflict & Health, 16(1), 58.

Monaghan, J. (2014). Security traps and discourses of radicalization: Examining surveillance practices targeting Muslims in Canada. Surveillance and Society, 12(4), 485-501.

MOPAC. (2019). A Shared Endeavour working in Partnership to Counter Violent Extremism in London: The London Countering Violent Extremism Programme Report 2018-2019.

Morris, A., & Meloy, J. R. (2020). A Preliminary Report of Psychiatric Diagnoses in a Scottish County Sample of Persons of National Security Concern. Journal of Forensic Sciences, 65(5), 1638-1645.

Mubaraq, Z., Arifin, S., Abdullah, I., Jubba, H., & Indiyanto, A. (2022). Return of the Lost Son: Disengagement and social reintegration of former terrorists in Indonesia. Cogent Social Sciences, 8(1).

Mullins, S. (2016). Counter-terrorism in Australia: Practitioner perspectives. Journal of Policing, Intelligence and Counter Terrorism, 11(1), 93-111.

Mykkänena, T. (2020). An Assessment of Returning Foreign Terrorist Fighters’ Commitment to Reintegrate: A Case Study of Kwale County, Kenya. Journal for Deradicalization.

Navarro-Granados, M., Llorent-Bedmar, V., & Palma, V. C. C. D. (2020). The views on terrorism in the name of Islam held by Islamic religion teachers in Spain. Religions, 11(11), 1-17.

Niconchuk, M. (2019). A Dangerous Displacement Crisis: The Psychological Ecology of Extremism After the Fall of ISIS. Terrorism, Radicalisation and Countering Violent Extremism: Practical Considerations and Concerns, 81-99.

Njoku, E. T. (2020). Investigating the intersections between counter-terrorism and NGOs in Nigeria: Development practice in conflict-affected areas. Development in Practice, 30(4), 501-512.

Nwagu, C. Y. (2009). Counter-Terrorism and human rights protection in Uganda: Preventing wrongs without violating rights. Master's thesis, University of Pretoria.

O'Donnell, A. (2016). Securitisation, Counterterrorism and the Silencing of Dissent: The Educational Implications of "Prevent". British Journal of Educational Studies, 64(1), 53-76.

O’Farrell, T., K, & Street, J. (2019). A Threat Inflated? The Countering and Preventing Violent Extremism Agenda in Kyrgyzstan. Saferworld.

O’Toole, T., Meer, N., DeHanas, D. N., Jones, S. H., & Modood, T. (2016). Governing through Prevent? Regulation and contested practice in State–Muslim engagement. Sociology, 50(1), 160-177.

Open Society Justice Initiative. (2016). Eroding Trust: The UK's PREVENT Counter-Extremism Strategy in Health and Education. Open Society Justice Initiative.

Oppetit, A., Campelo, N., Bouzar, L., Pellerin, H., Hefez, S., Bronsard, G., Bouzar, D., & Cohen, D. (2019). Do Radicalized Minors Have Different Social and Psychological Profiles From Radicalized Adults? Frontiers in Psychiatry, 10, 644.

Ortbals, C. D. (2023). Mothers Will Stop at Nothing: The Responses of Mothers of Terrorists. In D.Ü. Arıboğan & H. Khelghat-Doost (Eds.). Constructing Motherhood Identity Against Political Violence: Beyond Crying Mothers (pp. 21-38). Springer.

Parker, D., Pearce, J. M., Lindekilde, L., & Rogers, M. B. (2019). Challenges for effective counterterrorism communication: Practitioner insights and policy implications for preventing radicalization, disrupting attack planning, and mitigating terrorist attacks. Studies in Conflict & Terrorism, 42(3), 264-291.

Patouris, E. (2020). “What we need is not to judge, but to understand”: A Cyprus youth perspective on preventing youth radicalisation. Internet Journal of Restorative Justice, 2020(special issue 3), 33-50.

Pearce, J. M., Lindekilde, L., & Parker, D. (2023). Understanding UK university academic staff attitudes towards recognising and responding to student radicalisation. British Educational Research Journal, 49(6), 1254-1272.

Peatfield, E.-J. (2021). Race and radicalisation: Examining perceptions of counter-radicalisation policy amongst minority groups in Liverpool 8 and 24. PhD Thesis, The University of Liverpool.

Pilkington, H. (2023). Radicalization as and in Process: Tracing Journeys through an “Extreme-Right” Milieu. Studies in Conflict & Terrorism, 1-27.

Pilkington, H., & Hussain, A. (2022). Why wouldn't you consult us? Reflections on preventing radicalisation among actors in radical(ising) milieus. Journal for Deradicalization (30), 1-44.

Pirol, A. (2017). Protecting city from radical ideologies and activities. International Journal of Civic, Political, and Community Studies, 15(3), 9-18.

Poni, M., Kambellari, E., Zeneli, M., & Baci, R. (2021). Role of women in preventing radicalization and violent extremism that leads to terrorism in Albania. Academic Journal of Interdisciplinary Studies, 10(1), 240-247.

Pyszczynski, T., Rothschild, Z., Motyl, M., & Abdollahi, A. (2009). The cycle of righteous destruction: A Terror Management Theory perspective on terrorist and counter-terrorist violence. In W. G. K. Stritzke, S. Lewandowsky, D. Denemark, J. Clare, & F. Morgan (Eds.), Terrorism and torture: An interdisciplinary perspective (pp. 154–178). Cambridge University Press

Razdykova, G., Tucker, N., Ellis, B. H., Orell, R., Birman, D., & Weine, S. (2024). Operation Jusan in Year 4: Understanding and Addressing Present and Future Needs. Centre for Research and Evidence on Security Threats (CREST).

Reding, A., Van Gorp, A., Robertson, K., Walczak, A., Giacomantonio, C., & Hoorens, S. (2014). Handling ethical problems in counterterrorism. An inventory of methods to support ethical decisionmaking. RAND.

Reisman, L., & Payan, G. (2015). Turning away from MS-13 and al-Shabaab: Analyzing youth resilience in Honduras and North East Kenya. Education Development Center.

Reiter, J., Doosje, B., & Feddes, A. R. (2021). Radicalization and deradicalization: A qualitative analysis of parallels in relevant risk factors and trigger factors. Peace and Conflict: Journal of Peace Psychology, 27(2), 268-283.

Riany, Y. E., Haslam, D., Musyafak, N., Farida, J., Ma’arif, S., & Sanders, M. (2019). Understanding the role of parenting in developing radical beliefs: Lessons learned from Indonesia. Security Journal, 32(3), 236-263.

Robson, J., & Hunt, R. (2021). Lecturing within the panoptic scheme of Prevent in an English University. Critical Studies on Terrorism, 14(4), 578-597.

Romaniuk, P. (2015). Assessing violent extremism: The case of Burkina Faso. In Zeiger, S. & Aly, A. (Eds.). Countering violent extremism: Developing an evidence-base for policy and practice (pp. 37–48), Hedayah.

Sabir, R. (2014). Understanding Counter-Terrorism Policy and Practice in the UK since 9/11.

Saltman, E., Kooti, F., & Vockery, K. (2023). New models for deploying counterspeech: Measuring behavioral change and sentiment analysis. Studies in Conflict & Terrorism, 46(9), 1547-1574.

Sas, M., Ponnet, K., Reniers, G., & Hardyns, W. (2020). The Role of Education in the Prevention of Radicalization and Violent Extremism in Developing Countries. Sustainability, 12(6), 12.

Schanzer, D. H., & Eyerman, J. (2016). United States Attorneys’ community outreach and engagement efforts to counter violent extremism: Results from a nationwide survey.

Schanzer, D., & Eyerman, J. (2019). Engaging With Communities to Prevent Violent Extremism A Review of the Obama Administration’s CVE Initiative. Duke University and RTI International.

Schanzer, D., Kurzman, C., & Moosa, E. (2010). Anti-terror lessons of Muslim-Americans.

Schewe, J., & Koehler, D. (2021). When Healing Turns to Activism: Formers and Family Members’ Motivation to Engage in P/CVE. Journal for Deradicalization (28), 141-182.

Schlegel, L. (2022). “At least I tried, God damnit” and “Even a drop in the ocean is still more than nothing”: In their own words, what motivates people to work in P/CVE? Critical Studies on Terrorism, 15(4), 945-962.

Schneider, L. (2021). Counter-Terrorism on Campus : English Universities' Engagement with the Prevent Duty. PhD Thesis, University of Oxford.

Schumicky-Logan, L., & Dos Reis, A. A. (2023). Sensible localisation - local peace committees' role in preventing violent and hateful extremism. Conflict Security & Development, 23(5), 525-544.

Schumpe, B. M., Bélanger, J. J., Moyano, M., & Nisa, C. F. (2020). The Role of Sensation Seeking in Political Violence: An Extension of the Significance Quest Theory. Journal of Personality and Social Psychology, 118(4), 743-761.

Scrivens, R., Venkatesh, V., Bérubé, M., & Gaudette, T. (2022). Combating violent extremism: Voices of former right-wing extremists. Studies in Conflict & Terrorism, 45(8), 661-681.

Search for Common Ground. (2019). #Jashstan2: Youth as Agents of Peace and Stability in Kyrgyzstan: Conflict Assessment and Baseline Report. Search for Common Ground.

Seitakhmetova, N., Aliyarov, E., Zhandossova, S., Tolen, Z., & Nurov, M. (2024). Counteracting Religious Extremism in Youth Environments: World Experience and Kazakhstan. Journal of Social Studies Education Research, 15(3), 257-288.

Shally, A. (2023). WORKING WITH AND FOR YOUTH-Practical ideas to foster youth engagement.

Shortland, N., Alison, L., Rhodes, H., Thompson, L., Nader, E., Christiansen, P., Long, M., & Giles, S. (2022). An empirical assessment of the Prioritization and Intervention for Violent Extremists Tool (PIVET). Journal of Threat Assessment and Management, 9(2), 80-97.

Sikkens, E., Sieckelinck, S., van San, M., & de Winter, M. (2017). Parental reaction towards radicalization in young people. Child & Family Social Work, 22(2), 1044-1053.

Sikkens, E., van San, M., Sieckelinck, S., & de Winter, M. (2017). Parental Influence on Radicalization and De-radicalization according to the Lived Experiences of Former Extremists and their Families. Journal for Deradicalization (12), 192-226.

Sikkens, E., van San, M., Sieckelinck, S., & de Winter, M. (2018). Parents’ Perspectives on Radicalization: A Qualitative Study. Journal of Child & Family Studies, 27(7), 2276-2284

Sivenbring, J., & Andersson Malmros, R. (2019). Mixing logics: Multiagency approaches for countering violent extremism.

Sivenbring, J., & Malmros, R. A. (2023). The good, the bad, and the ugly–Involving civil society organizations in Nordic policy for preventing extremism. Journal of civil society, 19(1), 1-19.

Sjøen, M. M. (2023). The two faces of Janus: educational pathways into and out of violent extremism in Norway. Journal of Peace Education, 20(2), 217-240.

Sjøen, M. M. (2024). Engaging with the Elusiveness of Violent Extremism in Norwegian Schools–The Promise and Potential of Agonistic Listening. British Journal of Educational Studies, 72(3), 321-340.

Sommers, M. (2019). Youth and the Field of Countering Violent Extremism. Promundo.

Sonrexa, J., Kelly, L. M., Barton, G., & Ware, A. (2023). Perspectives on violent extremism from development–humanitarian NGO staff in Southeast Asia. Third World Quarterly, 44(1), 170-189.

Spalek, B. (2011). 'New terrorism' and crime prevention initiatives involving Muslim young people in the UK: Research and policy contexts. Religion, State and Society, 39(2-3), 191-207.

Spalek, B., & McDonald, L. Z. (2010). Terror Crime Prevention: Constructing Muslim Practices and Beliefs as ‘Anti-Social’ and ‘Extreme’ through CONTEST 2. Social Policy and Society, 9(1), 123-132.

Spalek, B., El Awa, S., McDonald, L., & Lambert, R. (2009). Police-Muslim engagement and partnerships for the purposes of counter terrorism: An examination. University of Birmingham.

Stahl, G., Baak, M., Schulz, S., Adams, B., & Peterson, A. (2021). Preventing violent extremism: Resourcing, stakeholder strategies and fostering belonging and connection in Australian schools. British Educational Research Journal, 47(5), 1177-1193.

Taylor, C., Semmelrock, T., & McDermott, A. (2019). The Cost of Defection: The Consequences of Quitting Al-Shabaab. International Journal of Conflict and Violence, 13, 657-657.

Thaqi, X., Shchekotin, E., Kaznacheev, D., Kaznacheeva, N., & Kaznacheeva, N. (2020). Social network big data analysis as a tool for preventing extremism on the Internet in the interests of sustainable development. E3S Web of Conferences, 208.

Thijssen, G., Sijtsema, J., Bogaerts, S., van de Voorde, L., & Masthoff, E. (2023). Radicalization Processes and Transitional Phases in Female and Male Detainees Residing in Dutch Terrorism Wings. Behavioral Sciences, 13(10), 23.

Thomann, E., Maxia, J., & Ege, J. (2023). How street-level dilemmas and politics shape divergence: The accountability regimes framework. Policy Studies Journal, 51(4), 793-816.

Thomas, P. (2011). Youth, multiculturalism and community cohesion. Springer.

Thomas, P. (2012). Responding to the Threat of Violent Extremism: Failing to Prevent. Bloomsbury.

Thomas, P. (2014). Divorced but still co-habiting? Britain's Prevent/community cohesion policy tension. British Politics, 9(4), 472-493.

Thomas, P., Grossman, M., Christmann, K., & Miah, S. (2020). Community reporting on violent extremism by “intimates”: emergent findings from international evidence. Critical Studies on Terrorism, 13(4), 638-659.

Toledo-Dumenes, J. A. (2024). Challenges to the integration of young Muslims in Europe and Spain: strategies and tactics of radical Islamist groups. Universitas-Revista De Ciencias Sociales Y Humanas(40), 67-92.

UNDP. (2019). Frontlines: Young People at the Forefront of Preventing and Responding to Violent Extremism.

Ungar, M. (2016). Building Social Inclusion and Community Engagement of Youth: Pathways to Resilience as Alternatives to Violence. In T. Morris & M. Hadji-Janevouth (Eds.). Countering Terrorism in South Eastern Europe (pp. 103-109). IOS Press.

Upal, A. (2015). Alternative narratives for preventing the radicalization of Muslim youth. Journal for Deradicalization (2), 138-162.

Valleau, A., Rahimov, K., & Cherkasov, A. (2018). The soft dimension of the Shanghai Cooperation Organization's fight against the "Three Evil Forces". Insights on counterterrorism preventive measures and youth education. European Journal of Contemporary Education, 7(4), 858-873.

van de Weert, A. (2022). Justice and risk assessment: the subjectivity of pre-emptive screening of radicalisation processes. Journal of Policing, Intelligence and Counter Terrorism, 17(3), 287-300.

van de Weert, A., & Eijkman, Q. (2021). Reconsidering early detection in countering radicalization by local frontline professionals. Ethics and terrorism, 177-188.

van de Weert, A., & Eijkman, Q. A. M. (2019). Subjectivity in detection of radicalisation and violent extremism: a youth worker's perspective. Behavioral Sciences of Terrorism and Political Aggression, 11(3), 191-214.

van de Weert, A., & Eijkman, Q. A. M. (2020). Early detection of extremism? The local security professional on assessment of potential threats posed by youth. Crime, Law and Social Change, 73(5), 491-507.

van de Weert, A., & Eijkman, Q. A. M. (2021). In Every Artery of Society? How Dutch Community Police Officers Perceive Their Role in Early Detection of Violent Extremism among Youth. Policing (Oxford), 15(2), 1144-1157.

van de Wetering, D., & Hecker, T. (2023). The Interplay of Psychological Stress, Aggression, Identity, and Implicit Knowledge: Findings from a Qualitative Study of Disengagement and Deradicalisation Processes Involving Former Right-Wing Extremists. In A. Kaya, A. Benevento, & M. Koca. (Eds.). Nativist and Islamist Radicalism (pp. 71-94). Routledge.

van San, M., Sieckelinck, S., & de Winter, M. (2013). Ideals adrift: An educational approach to radicalization. Ethics and Education, 8(3), 276-289.

Vaughn, L. (2019). "Doing Risk": Practitioner Interpretations of Risk of Childhood Radicalisation and the Implementation of the HM Government PREVENT Duty. PhD Thesis, The University of Liverpool.

Vaughn, L. (2024). ‘False evidence appearing real’: the evolution of pre-criminal safeguarding for childhood radicalisation in the UK. Critical and Radical Social Work, 1-21.

Vermeulen, F. (2014). Suspect Communities-Targeting Violent Extremism at the Local Level: Policies of Engagement in Amsterdam, Berlin, and London. Terrorism and Political Violence, 26(2), 286-306.

Vermeulen, F., & Bovenkerk, F. (2012). Engaging with violent Islamic extremism: Local policies in Western European cities. The Hague: Eleven International Publishers.

Vermeulen, F., & Visser, K. (2021). Preventing violent extremism in the Netherlands: Overview of its broad approach. Revista CIDOB d'Afers Internacionals(128), 131-153.

Vicente, Á. (2022). How radicalizing agents mobilize minors to jihadism: a qualitative study in Spain. Behavioral sciences of terrorism and political aggression, 14(1), 22-48.

Vicente, Á. (2022). Terrorist participation despite social influences opposing violent extremism: a qualitative study among young jihadists in Spain. Studies in Conflict & Terrorism, 1-24.

Voronkina, L. B., Serdyukova, E. F., Khrebina, S. V., Shapovalova, M. L., & Yundin, R. N. (2017). Research on the Chechen Republic: Youth value orientations as a factor of preventing the propensity for extremism. Man in India, 97(16), 241-251.

Vukičević, J. P., Prpić, M., & Korda, M. (2019). The Police Perception of the Role of School in Preventing Radicalization: The Case of Croatia. Varstvoslovje: Journal of Criminal Justice & Security, 21(2), 184-204.

Walter, F., Leonard, S., Miah, S., & Shaw, J. (2021). Characteristics of autism spectrum disorder and susceptibility to radicalisation among young people: A qualitative study. Journal of Forensic Psychiatry & Psychology, 32(3), 408-429.

Warrington, A. (2018). ‘Sometimes you just have to try something’-A critical analysis of Danish state-led initiatives countering online radicalisation. Journal for Deradicalization (14), 111-152.

Warshel, Y. (2010). How do you convince children that the "army", "terrorists" and the "police" can live together peacefully? A peace communication assessment model. University of California, San Diego.

Weine, S. E. E., & Polutnik, C. (2019). Helpful and harmful practices for addressing alleged transnational crimes in Somali-American communities.

Weine, S., & Ahmed, O. (2012). Building resilience to violent extremism among Somali-Americans in Minneapolis-St. Paul. Final Report to Human Factors/Behavioral Sciences Division, Science and Technology Directorate. US Department of Homeland Security.

Wendelberg, L. (2021). An Ontological Framework to Facilitate Early Detection of 'Radicalization' (OFEDR)-A Three World Perspective. Journal of Imaging, 7(3), 22.

Williams, M. J., Bélanger, J. J., Horgan, J., & Evans, W. P. (2019). Experimental Effects of a Call-Center Disclaimer Regarding Confidentiality on Callers’ Willingness to Make Disclosures Related to Terrorism. Terrorism and Political Violence, 31(6), 1327-1341.

Williams, M. J., Horgan, J. G., Evans, W. P., & Bélanger, J. J. (2020). Expansion and replication of the theory of vicarious help-seeking. Behavioral Sciences of Terrorism and Political Aggression, 12(2), 89-117.

Williams, M., Walsh Taza, R., & Prelis, S. (2016). Working Together to Address Violent Extremism: A Strategy for Youth-Government Partnerships.

Zeuthen, M. (2015). From Policy to Practice: Findings and lessons learned from a research-based pilot countering violent extremism programme in the Horn of Africa. Countering Violent Extremism: Developing an Evidence-Base for Policy and Practice.

**Exclusion: Intervention II - Criminal Justice Response**

Adcox, K. R. (2014). Community-oriented counterterrorism: incorporating national homeland security mandates into the local community policing philosophy. Defence and Peace Economics, 20(4), 287-301.

Adebayo, J. O. (2019). Nigerian Experience with Post-Conflict Peacebuilding: Examining Operation Safe Corridors and the Niger Delta Amnesty Programme. Journal of African Union Studies, 8(3).

Aksu, G. (2014). Winning hearts and minds in counterterrorism through community policing and procedural justice: Evidence from Turkey. American University.

Alsubaie, B. A. (2016). Countering Terrorism in the Kingdom of Saudi Arabia: An Examination of the Prevention, Rehabilitation, and After-Care strategy (PRAC). University of Newhaven.

Anindya, C. R. (2019). The deradicalisation programme for Indonesian deportees: A vacuum in coordination. Journal for Deradicalization (18), 217-243.

Azam, Z., & Fatima, S. B. (2017). Mishal: A case study of a deradicalization and emancipation program in Swat Valley, Pakistan. Journal for Deradicalization (11), 1-29.

Aziz, S. F. (2014). Policing terrorists in the community. Harvard National Security Journal, 5, 147.

Bahadur Lamb, J. (2014). Gendered counter terrorism? The potential impact of police officer perceptions of PREVENT policing. Behavioral Sciences of Terrorism and Political Aggression, 6(3), 183-194.

Barkindo, A., & Bryans, S. (2016). De-radicalising prisoners in Nigeria: Developing a basic prison based de-radicalisation programme. Journal for Deradicalization, 7, 1-25.

Boucek, C. (2008). Counter-Terrorism from Within: Assessing Saudi Arabia’s Religious Rehabilitation and Disengagement Programme. The RUSI Journal, 153(6), 60-65.

Brett, J. (2012). Recent Danish Counterradicalization Initiatives: A Case Study on the Danish Security and Intelligence Service’s Dialogue Forum. Global Center on Cooperative Security.

Bullock, K., & Johnson, P. (2018). Police engagement with Muslim communities: breaking out, breaking in, and breaking through. Policing and Society, 28(8), 879-897.

Chalmers, I. (2017). Countering violent extremism in Indonesia: Bringing back the Jihadists. Asian Studies Review, 41(3), 331-351.

Cherney, A. (2018). Police community engagement and outreach in a counterterrorism context. Journal of Policing, Intelligence and Counter Terrorism, 13(1), 60-79.

Cherney, A. (2020). Evaluating interventions to disengage extremist offenders: A study of the Proactive Integrated Support Model (PRISM). Behavioral Sciences of Terrorism and Political Aggression, 12(1), 17-36.

Cherney, A., & Belton, E. (2020). Assessing intervention outcomes targeting radicalised offenders: Testing the Pro Integration Model of extremist disengagement as an evaluation tool. Dynamics of Asymmetric Conflict, 13(3), 193-211.

Clark, C. (2023). Relationship between detention facility experiences of juvenile delinquents and their exposure to criminalization. PhD Thesis, Walden University

Cockayne, J., O’Neil, S., Felbab-Brown, V., Chowdhury Fink, N., & Oswald, B. (2015). UN DDR in an Era of Violent Extremism: Is It Fit for Purpose? United Nations University.

Cook, J. (2023). Distinguishing Children From ISIS-Affiliated Families in Iraq and Their Unique Barriers for Rehabilitation and Reintegration. Perspectives on Terrorism, 17(3), 42-70.

Davies, H. J., & Plotkin, M. R. (2005). Protecting your community from terrorism: Strategies for local law enforcement.

Davis, L. M., Pollard, M., Ward, K., Wilson, J. M., Varda, D. M., Hansell, L., & Steinberg, P. (2010). Long-term effects of law enforcement's post-9/11 focus on counterterrorism and homeland security. RAND Corporation

de Meerendré, L. K., Varga, R., Brion, F., Crahay, C., Verfaillie, K., Hanard, E., De Kimpe, S., Coline, R., Vanneste, C., & Van Praet, S. (2022). Impact Assessment of Belgian De-„Radicalisation “Policies Upon Social Cohesion and Liberties. Final Report.

Dean, C., Lloyd, M., Keane, C., Powis, B., & Randhawa, K. (2018). Interviewing With Extremist Offenders–A Pilot Study. HMPPS.

Dresser, P. (2015). Understanding Prevent Policing through Dispositif and Reflexive Risk. PhD Thesis, Manchester Metropolitan University

Dresser, P. (2019). “Trust your instincts–act!” PREVENT police officers’ perspectives of counter-radicalisation reporting thresholds. Critical Studies on Terrorism, 12(4), 605-628.

Dunn, K. M., Atie, R., Kennedy, M., Ali, J. A., O’Reilly, J., & Rogerson, L. (2015). Can you use community policing for counter terrorism? Evidence from NSW, Australia. Police Practice and Research, 17(3), 196-211.

Ehiane, S. (2019). Deradicalisation and Disengagement of the Extremist Group in Africa. The Nigerian Experience Journal of African Foreign Affairs, 6(2).

Ellefsen, R., & Sandberg, S. (2022). Everyday Prevention of Radicalization: The Impacts of Family, Peer, and Police Intervention. Studies in Conflict & Terrorism, 24.

Ezzarqui, L. (2010). De-radicalization and rehabilitation program: the case study of Saudi Arabia. PhD Thesis, Georgetown University.

Førde, K. E., & Andersen, A. J. (2021). «Basically, I am here to help you»: Police conversation interventions in the prevention of radicalisation and violent extremism as a pastoral technique of power. Nordisk Tidsskrift for Kriminalvidenskab, 108(2), 311-326

Gale, J. (2012). Is the machinery of local policing delivery seen as fit for purpose by practitioners and community members to anticipate and mitigate the risk of harmful radicalisation at street level? PhD Thesis, University of Exeter.

Guru, S. (2012). Under siege: Families of counter-terrorism. British Journal of Social Work, 42(6), 1151-1173.

Hassan, I. (2022). Reintegrating ex-combatants: An assessment of Operation Safe Corridor. Journal for Deradicalization (33), 150-180.

Heath-Kelly, C. (2024). Multi-agency counter-terrorism in Britain and Norway: Intelligence agencies and the administration of welfare. Security Dialogue, 09670106241234203.

Hirschfield, A., Christmann, K., Wilcox, A., Rogerson, M., & Sharratt, K. (2012). Process Evaluation of Preventing Violent Extremism Programmes for Young People. Youth Justice Board

HMIFRCS. (2018). Counter-terrorism policing An inspection of the police’s contribution to the government’s Prevent programme. HMIFRCS

Innes, M., Roberts, C., & Lowe, T. (2017). A Disruptive Influence? “Prevent-ing” Problems and Countering Violent Extremism Policy in Practice. Law & Society Review, 51(2), 252-281.

Khalil, J., Brown, R., Chant, C., Olowo, P., & Wood, N. (2019). Deradicalisation and disengagement in Somalia: Evidence from a rehabilitation programme for former members of Al-Shabaab. RUSI.

Khan, I. (2024). The Resurgence of Militant Groups in Pakistan's Borderlands: Analyzing Shortcomings in Rehabilitation and Reintegration Strategies. Journal for Deradicalization (38), 1-27.

Kurtenbach, S., Schumilas, L., Kareem, A., Waleciak, J., & Zaman, M. (2021). A cross-cultural comparison of deradicalisation: Results from Germany and Pakistan. Journal for Deradicalization (29), 205-252.

Kutner, S. (2016). The call for component analyses of the Saudi Arabian Risk Reduction Initiative: An examination of religious re-education’s role in the deradicalization and disengagement Process. Jornal for Deradicalization.

Lamb, J. B. (2012). Preventing Violent Extremism; A Policing Case Study of the West Midlands. Policing: A Journal of Policy and Practice, 7(1), 88-95.

Lambert, R. A. (2011). Countering al-Qaeda in London: Police and Muslims in partnership. Hurst.

Lambert, R., & Parsons, T. (2020). Community-based counterterrorism policing: recommendations for practitioners. In B. Spalek & D. Weeks (Eds.). Communities and Counterterrorism (pp. 68-85). Routledge.

Manby, M. (2009). Evaluation of Kirklees Youth Offending Team (PVE) Pilot Parenting Project. Nationwide Children’s Research Centre.

Manby, M. (2009). Kirklees Youth Offending Team Prevent (previously PVE) Programme—Evaluation of Theatre Project. Nationwide Children’s Research Centre.

Manby, M. (2009). Kirklees Youth Offending Team. Prevent (previously PVE) Programme—Evaluation of Film Project. Nationwide Children’s Research Centre.

Manby, M. (2009). Kirklees Youth Offending Team. Prevent (previously PVE) Programme—Evaluation of Diversity Group. Nationwide Children’s Research Centre.

Manby, M. (2010). Evaluation of Kirklees Youth Offending Team Prevent (previously PVE) Project Evaluation of Citizenship Programme. Nationwide Children’s Research Centre.

Manby, M. (2010). Kirklees Youth Offending Team Prevent (previously PVE) Project Evaluation of Pathways into Adulthood Programme. Nationwide Children’s Research Centre.

Marion, N., & Cronin, K. (2009). Law Enforcement Responses to Homeland Security Initiatives: The Case of Ohio. Southwest Journal of Criminal Justice, 6(1).

Maulana, I., Indriana, D., & Goei, G. (2022). The Relevance of High-Risk Prisons to Indonesia’s Preventing Violent Extremism Policy. Perspectives on Terrorism, 16(3), 22-36.

Membrives, M. T. G., & Alonso, R. (2022). Countering Violent Extremism in Spain: Analyzing the Intervention with Young Jihadi Convicted of Membership of a Terrorist Organization. Studies in Conflict & Terrorism, 33.

Meringolo, P. (2020). Preventing Violent Radicalisation in Europe: Multidisciplinary Perspectives. Springer

Meringolo, P., Bosco, N., Cecchini, C., & Guidi, E. (2019). Preventing violent radicalization in Italy: The actions of EU project PROVA. Peace and Conflict: Journal of Peace Psychology, 25(2), 165-169.

Meringolo, P., Guidi, E., Balvin, N., & Christie, D. J. (2020). Promoting Civic Engagement and Social Inclusion Interventions for Minors Involved with Crimes. Children and Peace: From Research to Action,

Meringolo, P., Guidi, E., Cecchini, C., & Donati, C. (2018). Youth violent radicalisation. Preventive strategies and community-based practices in Europe.

Milla, M. N., Hudiyana, J., & Arifin, H. H. (2020). Attitude toward rehabilitation as a key predictor for adopting alternative identities in deradicalization programs: An investigation of terrorist detainees' profiles. Asian Journal of Social Psychology, 23(1), 15-28.

Murphy, G. R., Plotkin, M. R., & Flynn, E. A. (2003). Protecting Your Community from Terrorism. Improving Local-Federal Partnerships.

Murray, M. (2018). Assessing The Importance of CVE Strategies in Ontario.

NSW Ombudsman (2007). Review of Parts 2A and 3 of the Terrorism (Police Powers) Act 2002 : Preventative detention and covert search warrants.

Odubo, E. F. (2014). Educational impact of Nigeria's amnesty program on the Niger Delta ex-militants. PhD Thesis, Walden University.

Onapajo, H., & Ozden, K. (2020). Non-military approach against terrorism in Nigeria: Deradicalization strategies and challenges in countering Boko Haram. Security Journal, 1-17.

Onyango, R. (2018). Process Evaluation of Terrorism Amnesty and Reintegration Program, and Perceptions of the Program Within Kenya Police. PhD Thesis, City University of New York

Orban, F. (2022). Building Trust Alliances to Rehabilitate Terrorists and Radicalized Prisoners? In F. Orban & E. S. Larsen (Eds.). Living alliances, leaving alliances: Interdisciplinary perspectives (pp. 165-187). Waxman

Paripurna, A., Sarwirini, & Subandi, I. (2021). Pathway of School-Age Youth into Violent Extremist Activity and the De-Radicalisation Programme in Indonesia. International Journal of Criminal Justice Sciences, 16(1), 1-16.

Peracha, F., Khan, R. R., & Savage, S. (2016). Sabaoon: Educational methods successfully countering and preventing violent extremism. In S. Zeiger & A. Aly (Eds.). Expanding research on countering violent extremism (pp. 85-104). Hedayah.

Peracha, F., Savage, S., Khan, R., Ayub, A., & Zahra, A. (2022). Promoting Cognitive Complexity Among Violent Extremist Youth in Northern Pakistan. Journal of Strategic Security, 15(1), 14-53.

Peterson, A. (2012). Legitimacy and the Swedish Security Service’s Attempts to Mobilize Muslim Communities. International Journal of Criminology and Sociology, 1, 109-120.

Pickering, S., McCulloch, J., & Wright-Neville, D. (2008). Counter-terrorism policing: Towards social cohesion. Crime, Law and Social Change, 50(1-2), 91-109.

Ramakrishna, K. (2009). 'Counter‐Ideological’ Work in Singapore: A Preliminary Assessment. Journal of Policing, Intelligence and Counter Terrorism, 4(2), 41-51.

Ramirez, D. A., & Quinlan, T. L. (2008). The Greater London Experience: Essential lessons learned in law enforcement-community partnerships and terrorism prevention.

RAN (2023). The Dutch Multi-Agency Approach to Rehabilitation of Radicalised Detainees (MAR).

Rhazzali, M. K., & Schiavinato, V. (2023). Adolescence as a “Radical” Age and Prevention of Violent Radicalisation: A Qualitative Study of Operators of a Juvenile Penal Circuit in Italy. Religions, 14(8).

San, S. (2020). Counter-terrorism policing innovations in Turkey: A case study of Turkish National Police CVE experiment. Policing and Society.

Sozeri, S., Altinyelken, H., & Volman, M. (2022). The role of mosque education in the integration of Turkish-Dutch youth: Perspectives of Muslim parents, imams, mosque teachers and key stakeholders. Ethnic and Racial Studies, 45(16), 122-143.

Spalek, B. (2012). Policing within counter-terrorism. In B. Spalek (Ed.). Counter-Terrorism: Community-Based Approaches to Preventing Terror Crime (pp. 50-73). Palgrave Macmillan.

Sukabdi, Z. A. (2019). Motivation-Ideology-Capability (MIC) Risk Assessment and Treatment Management for Preventing Ideology-Based Terrorism Recidivism in Indonesia. Swinburne University of Technology Melbourne, Australia.

Sukabdi, Z. A., Daffern, M., Ogloff, J. R., & Suwartono, C. (2022). Competencies for rehabilitation professionals working with ideology-based terrorism offenders. Crime, Law and Social Change, 78(2), 189-217.

Sumpter, C. (2021). Lab-in-Field Experiments for the Reintegration of Violent Extremists: The Promise of Prosocial Evaluation.

Sumpter, C., Wardhani, Y. K., & Priyanto, S. (2021). Testing transitions: Extremist prisoners re-entering Indonesian society. Studies in Conflict & Terrorism, 44(6), 473-494.

Syafiq, M. (2019). Deradicalisation and Disengagement from Terrorism and Threat to Identity: An Analysis of Former Jihadist Prisoners’ Accounts. Psychology and Developing Societies, 31(2), 227-251.

Ugwueze, M. I., Ngwu, E. C., & Onuoha, F. C. (2022). Operation Safe Corridor Programme and Reintegration of Ex-Boko Haram Fighters in Nigeria. Journal of Asian and African Studies, 57(6), 1229-1248.

UNODC. (2023). STRIVE Juvenile: Preventing and Responding to Violence against Children by Terrorist and Violent Extremist Groups (part of GLOZ43).

USAID. (2021). Demobilization, Disassociation, Reintegration, and Reconciliation (DDRR) in Northeast Nigeria: Public report.

Vanchoski, & et al. (2020). Enhancing the Understanding of Foreign Terrorist Fighters (FTF): Challenges for Rehabilitation, Resocialization and Reintegration of Returnees in the Republic of North Macedonia.

Veldhuis, T. (2015). Captivated by fear: An evaluation of terrorism detention policy.

Watchlist on Children and Armed Conflict (2020). Countering Terrorism and Violent Extremism: The erosion of children’s rights in armed conflict.

Webster, S., Kerr, J., & Tompkins, C. (2017). A Process Evaluation of the Structured Risk Guidance for Extremist Offenders. Ministry of Justice Analytical Series.

Weggemans, D., & De Graaf, B. (2017). Reintegrating Jihadist Extremist Detainees: Helping Extremist Offenders Back into Society. Routledge.

Weine, S. P. C., & Younis, A. (2015). The Role of Community Policing in Countering Violent Extremism.

Weine, S., & Ahmed Younis, C. P. (2017). Community Policing to Counter Violent Extremism: A Process Evaluation in Los Angeles. Final Report Office of University Programs, Science and Technology Directorate, U.S. Department of Homeland Security. College Park, MD: START.

**Exclusion: Intervention III - Out of Scope**

Abbas, T., Obe, W., Ahmed, Z., Kabal, R., Lyne, A., Dadabhai, S., & Jiang, S. (2008). Preventing violent extremism: An independent evaluation of the Birmingham Pathfinder. Waterhouse Consulting Group

Abdellatif, M. S. (2022). A Training Programme on Constructive Thinking Skills for Reducing Psychological Defeatism and Attitude towards Intellectual Extremism of University Students. Cypriot Journal of Educational Sciences, 17(4), 1032-1050.

Abdulsalam, Y. K., Theeboom, M., & Coalter, F. (2023). Football-based prevention of radicalisation: A Theory of Change for football-based prevention of Radicalisation. Journal for Deradicalization (35), 138-173.

Abu-Nimer, M., & Nasser, I. (2017). Building peace education in the Islamic educational context. International Review of Education, 63(2), 153-167.

Agency for Peacebuilding (2022). SUMMARY REPORT: “Leading the Way to Peace” Project Evaluation.

Ahmed, M. (2021). The Use of Counter Narratives as a Prevention and Countering Violent Extremism (P/CVE) Communications-Based Measure: A Study of Muslim-American Undergraduate Students in Universities and Colleges in California. PhD Thesis, Old Dominion University.

Akbar, I. (2019). Strategy of Cirebon City to Prevent Radicalism: An ethnographic study of the non-formal education system. International Journal of Innovation, Creativity and Change, 8(4), 248-260.

Akdag, M., Alasag, A., Gürlesin, Ö., & Avest, I. T. (2019). A light-hearted way for young people to develop a religious life orientation. Studies in Interreligious Dialogue, 9(1), 103-123.

Al Masri, M., & Slavona, I. (2018). More Resilient, Still Vulnerable: Taking Stock of Prevention of Violent Extremism Programming with Youth in Tripoli, Lebanon. International Alert.

Al-Maqosi, Y. A., Al-Bataineh, M. T., & Al-Kilani, A. M. (2019). The Effectiveness of an Educational Program for Developing Tolerance Values and Resistance to Intellectual Extremism at Secondary Level in Jordan. Journal of Educational and Psychological Studies, 13(4), 628-642.

Al-Saeed Mohamed, Z. A., & Habib, A. M. O. (2024). THE ROLE OF ISLAMIC STUDIES COURSES IN PREVENTING EXTREMISM AND TERRORISM AMONG FEMALE STUDENTS AT PRINCE SATTAM BIN ABDULAZIZ UNIVERSITY. Synesis, 16(1), 167-187.

Alabdulhadi, M. M. J., & Alkandari, K. M. (2024). Practices of Islamic education teachers in promoting moderation (wasatiyyah) values among high school students in Kuwait: challenges and obstacles. Cogent Education, 11(1).

Alajmi, M. M. (2021). The role of the college of education at Kuwait University in countering violent extremism among students. Journal of Social Studies Education Research, 12(3), 204-224.

Aldrich, D. P. (2014). First steps towards hearts and minds? USAID’s countering violent extremism policies in Africa. Terrorism and Political Violence, 26(3), 523-546.

Algristian, H., Choiriya, D. D., Abdillah, D. S., Ulya, A., Sodali, H. A., & Muhammad, A. R. (2019). Why does de-radicalization seem a utopia? Evaluation on “children of the country” program. Journal of Public Health in Africa, 10(S1), 148-151.

Ali, N., Afwadzi, B., Abdullah, I., & Mukmin, M. I. (2021). Interreligious Literacy Learning as a Counter-Radicalization Method: A New Trend among Institutions of Islamic Higher Education in Indonesia. Islam & Christian Muslim Relations, 32(4), 383-405.

Ali, R., Özvatan, Ö., & Walter, L. (2023). The narrative foundations of radical and deradicalizing online discursive spaces: A comparison of the cases of Generation Islam and Jamal al-Khatib in Germany. Religions, 14(2), 167.

Ali, Y., Ahmad, A., Shah, Z. A. A., & Awan, M. A. (2020). Preventing radicalism in educational institutions: Pakistan a case in point. Journal of Public Affairs, 20(3), 8.

Aly, A., Taylor, E., & Karnovsky, S. (2014). Moral disengagement and building resilience to violent extremism: An education intervention. Studies in Conflict & Terrorism, 37(4), 369-385.

Aly, A., Weimann-Saks, D., & Weimann, G. (2014). Making ‘Noise’ Online An Analysis of the Say No to Terror Online Campaign. Perspectives on Terrorism, 8(5), 33-47.

Amanullah, Z., & Harrasy, A. (2017). Between Two Extremes, Responding to Islamist and tribalist messaging online in Kenya during the 2017 elections. Institute for Strategic Dialogue.

Amegboh, J. E., & Rothbart, D. (2021). Peacebuilding from the Bottom: Local Youth Non- Governmental Organizations Strategies to Prevent Terrorism and Violent Extremism in Mali. PhD Thesis, George Mason University.

Amin, A., Kurniawan, D. A., Chen, D., Wirayuda, R. P., & Alimni. (2022). Servation of Bengkulu Local Wisdom: The Application of Syarafal Anam in Preventing Student Radicalism. International Journal of Instruction, 15(3), 931-948.

Amin, A., Rizal, S., & Wulandari, A. (2022). Islamic “Syarafal Anam” Culture: Can This Prevent Students’ Radicalism? Eurasian Journal of Educational Research, 2022(97), 182-201.

Amit, S., Barua, L., & Kafy, A. A. (2021). Countering violent extremism using social media and preventing implementable strategies for Bangladesh. Heliyon, 7(5), e07121.

Amit, S., Kafy, A. A., & Barua, L. (2022). Mapping successful countering violent extremism strategies for implementation in Bangladesh. In M. Boskovic, G. Misev., & N. Putnik (Eds.). Fighting for Empowerment in an Age of Violence (pp. 55-86). IGI Global.

Amjad, N., & Wood, A. M. (2009). Identifying and changing the normative beliefs about aggression which lead young Muslim adults to join extremist anti-Semitic groups in Pakistan. Aggressive Behavior, 35(6), 514-519.

Anggraeni, D. (2022). An Initiative of Women in Preventing and Countering Violence Extremism: The Mothers for Change Program. In G. Barton, M. Vergani, & Y. Wahid (Eds.). Countering Violent and Hateful Extremism in Indonesia. New Security Challenges (pp. 85-100). Palgrave Macmillan.

Anis, E. Z. (2016). Countering Terrorist Narratives: Winning the Hearts and Minds of Indonesian Millennials. 1st International Conference on South East Asia Studies (ICSEAS), 189-210.

Antonysamy, A. P. (2017). Peace Building Among the Sri Lankan Refugees Tamil Nadu, India: A Critical Study of Educational Engagements of Jesuit Refugee Service (JRS). Master's thesis, Fordham University.

Aprin, F., Manske, S., Chounta, I. A., & Hoppe, H. U. (2021). Is This Fake or Credible? A Virtual Learning Companion Supporting the Judgment of Young Learners Facing Social Media Content. Lecture Notes in Computer Science (including subseries Lecture Notes in Artificial Intelligence and Lecture Notes in Bioinformatics), 13103 LNCS, 52-60.

Asif, U. (2018). “Sports for Development and Peace” and Violent Extremism: The Role of Sports for Development and Peace Programs to Prevent Violent Extremism Among Youth in Pakistan. Seoul National University.

Asif, U., & Charway, D. (2024). Analysing the role of sport for development and peace programmes in high-risk communities: Violent Extremism among the Youth of Pakistan. In B. Graeff, S. Šafaříková & L. Cherurbai Sambili-Gicheha (Eds.). Routledge Handbook of the Global South in Sport for Development and Peace (pp. 231-245). Routledge.

Asimovic, N., Ditlmann, R. K., & Samii, C. (2024). Estimating the effect of intergroup contact over years: evidence from a youth program in Israel. Political Science Research and Methods, 12(3), 475-493.

Audit Commission. (2008). Preventing Violent Extremism: Learning and Development Exercise. Report to the Home Office and Communities and Local Government.

Axiom Monitoring & Evaluation. (2015). Final Report SEED II: Exploring the Agricultural Initiatives Influence on Stability in Somalia. Department for International Development.

Babiyants, K. (2012). Reflexive game as a means to prevent extremism among youth. International Journal of Psychology, 47, 402-403.

Babiyants, K. A. & Manuylova, O. V. (2020). Reflexive Game in the Work with Students from Post-conflict Regions. 6th International Forum on Teacher Education (IFTE), 171-182.

Bala, A. (2017). Bottom-up approach to countering violent extremism in Tunisia: Evaluation Report. Search for Common Ground Tunisia.

Barzegar, A., Powers, S., & El Karhili, N. (2016). Civic approaches to confronting violent extremism. Institute for Strategic Dialogue.

Basmeh, & Zeitooneh. (2018). Exploring Resilience, Violent Extremist Thinking, and the Impact of Peace Education on Syrian Youth in Shatila. International Alert.

Basse, Y. O. (2018). Final Evaluation Kallewa Manio: An Integrated Approach to Counter Violent Extremism in Diffa. Search for Common Ground.

Bean, S., Hill, P., Sany, J., & Riveles, S. (2011). USAID/West Africa Peace through Development (PDEV): Program Assessment Report. USAID.

Beaujouan, J., & Rasheed, A. (2022). Investigating the role of religious institutions in the prevention of violent extremism in Nineveh province, Iraq. Journal for Deradicalization (32), 76-109.

Beider, H., & Briggs, R. (2010). Promoting community cohesion and preventing violent extremism in higher and further education.

Bélanger, J. J., Nisa, C. F., Schumpe, B. M., Gurmu, T., Williams, M. J., & Putra, I. E. (2020). Do counter-narratives reduce support for ISIS? Yes, but not for their target audience. Frontiers in Psychology, 11, 1059.

Bessingpas, M. T. (2009). Reforming primary education in Pakistan in the interest of US national security. PhD Thesis, Georgetown University.

Beza, T. (2016). CRITICAL CHOICES-Assessing the Effects of Education and Civic Engagement on Somali Youths’ Propensity Towards Violence. Mercy Corps.

Bhulai, R. (2017). GOING LOCAL: Supporting Community-Based Initiatives to Prevent and Counter Violent Extremism in South and Central Asia. Global Center on Cooperative Security.

Bivens, F. et al. (2021). PEACE, MY HEART: Amplifying Youth-Led Peacebuilding in South Asia.

Blair, G., Littman, R., Nugent, E. R., Wolfe, R., Bukar, M., Crisman, B., Etim, A., Hazlett, C., & Kim, J. (2021). Trusted authorities can change minds and shift norms during conflict. Proceedings of the National Academy of Sciences of the United States of America, 118(42), 19.

Bodine-Baron, E., Marrone, J. V., Helmus, T. C., & Schlang, D. (2020). Countering Violent Extremism in Indonesia: Using an Online Panel Survey to Assess a Social Media Counter-Messaging Campaign. RAND Corporation.

Bokhari, F., & Zahid Shahab, A. (2020). Challenges and opportunities for peace educators: Lessons from a youth‐led effort in Pakistan. Conflict Resolution Quarterly, 38(1-2), 73-86.

Bonnell, J. (2010). Teaching approaches that help to build resilience to extremism among young people. UK Department for Education.

Bourgeois-Guerin, E., Miconi, D., Rousseau-Rizzi, A., & Rousseau, C. (2021). Evaluation of a training program on the prevention of violent radicalization for health and education professionals. Transcultural Psychiatry, 58(5), 712-728.

Boyd-MacMillan, E. M. (2016). Increasing Cognitive Complexity and Collaboration Across Communities: Being Muslim Being Scottish. Journal of Strategic Security, 9(4), 79-110.

Boyd-MacMillan, E. M., Campbell, C., & Furey, A. (2016). An IC Intervention for Post-Conflict Northern Ireland Secondary Schools. Journal of Strategic Security, 9(4), 111-124.

Boyd-MacMillan, E. M., Patricia Andrews, F., Ptolomey, A. M., & Mathieson, L. J. (2016). I SEE! Scotland: Tackling Sectarianism and Promoting Community Psychosocial Health. Journal of Strategic Security, 9(4), 53-78.

Boyle, P., Bouasla, E., & Abderebbi, M. (2016). Mid-Term Evaluation Favorable Opportunities to Reinforce Self-Advancement for Today’s Youth. USAID.

Chatellier, S. (2012). Pakistani Women Moderating Extremism A Coalition-Building Case Study. The Institute for Inclusive Security.

Christiaens, E., Hardyns, W., & Pauwels, L. (2018). Evaluating the BOUNCEUp tool: Research findings and policy implications. Federal Public Service Home Affairs.

Christiaens, E., Hardyns, W., Pauwels, L., & Klima, N. (2018). Preventing crime and violent extremism by strengthening youth resilience: Implementation of the BOUNCE resilience tools in 10 European cities. Freedom from Fear, 2018(14), 162-173.

Colibaba, A., Gheorghiu, I., Colibaba, C., Danaila, L., & Ursa, O. (2019). SMILE AND THE WORLD WILL SMILE AT YOU: THE SMILE PROJECT. In ICERI2019 Proceedings (pp. 2140-2146). IATED.

D'Lima, P. (2019). Promoting tolerance to personal uncertainty : An exploratory study of a preventative universal intervention. PhD Thesis: Cardiff University.

Dafnos, A. (2014). Narratives as a means of countering the radical right; Looking into the Trojan T-shirt project. Journal Exit-Deutschland, 3, 156-188.

Dashtgard, P., Bates, L., Pressman, E., Russell, W., Gebbia-Richards, D., Hughes, B. & Miller-Idriss, C. (2021). EMPOWERED TO INTERVENE AN IMPACT REPORT ON THE SPLC/PERIL GUIDE TO YOUTH RADICALIZATION. SPLC & PERIL.

De Silva, S. (2017). Role of Education in the Prevention of Violent Extremism. World Bank.

Dechesne, M., & Ahajjaj, J. (2021). Discover your inner strength: A positive psychological approach to bolster resilience and address radicalization. Frontiers in Psychology, 12.

Dhungana, S. K., Ismanbaeva, R., & Aisakhunova, A. (2016). Reducing Violent Religious Extremism and Preventing Conflict in Kyrgyzstan and Central Asia 2013-2016. Search for Common Ground.

Dietrich, K. (2018). The Way Foward, Assessing the Impact of the “White Dove” CVE Radio Project in Northern Nigeria. Equal Access International.

Dolphin, D., & Pirlogea, I. (2023). Teachers in Romanian Secondary Schools: Challenges and Responses in Preventing Polarization and Extremism. Italian Journal of Sociology of Education, 15(3), 149-174.

Domergue, C. M., & Julien. (2021). Providing support to children and youth vulnerable to or affected by radicalization leading to violent extremism (RLVE). Hedayah.

Domergue, J. (2022). Supporting Families in Countering Violent Extremism in Nigeria: Evaluation Report 2019-2022. Hedayah.

Doney, J., & Wegerif, R. (2017). Measuring Open-Mindedness: An evaluation of the impact of our school dialogue programme on students’ open mindedness and attitudes to others. Tony Blair Institute for Global Change.

Duzcu, M. (2020). TURKEY: Youth empowerment through governmental initiatives. Conflict Studies Quarterly(30), 3-30.

Ebers, A., & Thomsen, S. L. (2022). Evaluating an interactive film on the prevention of political radicalization. Journal for Deradicalization (30), 169-222.

Effendi, R., Sukmayadi, V., & Unde, A. A. (2022). Social media as a medium for preventing radicalization (A case study of an Indonesian youth community's counter-radicalization initiatives on Instagram). Plaridel, 19(2).

Ekpon T. (2017). The role of young people in preventing violent extremism in the Lake Chad Basin. Civil Society Platform for Peacebuilding and State-building.

Elliott, K. E. (2022). What do teachers have to do with it?: Finnish teachers on preventing violent extremism. Drexel University.

Erdemandi, M. E. S., & Michael, J. W. (2024). Assessing the Effectiveness of Programs To Prevent and Counter Violent Extremism. NIJ Journal, 285.

Eurasia Foundation of Central Asia. (n.d). The Contribution of Positive Youth Development in Tajikistan to Effective Peacebuilding and to Countering or Preventing Violent Extremism: Successes, Limitations, and Recommendations.

Falkheimer, J. (2022). Strategies to Counter Extremism and Radicalisation in Swedish Schools – Managing Salafi Jihadists Attempts to Influence Students. Scandinavian Journal of Public Administration, 26(1), 67-86.

Faridi, F., & Umiarso, U. (2024). Deradicalization in Islamic Education in Indonesia: A Phenomenological Study. Eurasian Journal of Educational Research, (109), 59-73.

Fatgehipon, A. H., & Bin-Tahir, S. Z. (2019). Building students state defending awareness in preventing the radicalism. International Journal of Scientific and Technology Research, 8(10), 3536-3539.

Feddes, A. R., Huijzer, A., van Ooijen, I., & Doosje, B. (2019). Fortress of Democracy: Engaging youngsters in democracy results in more support for the political system. Peace and Conflict: Journal of Peace Psychology, 25(2), 158.

Feddes, A. R., Mann, L., & Doosje, B. (2015). Increasing self-esteem and empathy to prevent violent radicalization: A longitudinal quantitative evaluation of a resilience training focused on adolescents with a dual identity. Journal of Applied Social Psychology, 45(7), 400-411.

Finkel, S. E., Belasco, C. A., Gineste, C., Neureiter, M., & McCauley, J. (2018). Peace Through Development II Burkina Faso, Chad and Niger Impact Evaluation Endline Report. USAID.

Finkel, S. E., Belasco, C. A., Neureiter, M., McCauley, J., Hoepers, B., & Corrigan, C. C. (2017). USAID/WEST Africa (USAID/WA) Evaluation & Analytical Services (EAS) Project for the Regional Peace and Governance Programs, Midline Report for Impact Evaluation of the Peace Through Development Phase II (PDEV II) Project in Chad, Niger, and Burkina Faso. USAID.

Finkel, S. E., Rojo-Mendoza, R. T., Schwartz, C. L., Belasco, C. A., & Kreft, A. (2015). Evaluation & Analytical Services (EAS) Project for The Regional Peace and Governance Programs Impact Evaluation of Peace through Development II (P-DEV II) Radio Programming in Chad and Niger Final Report. USAID.

Gansewig, A., & Walsh, M. (2021). Broadcast Your Past: Analysis of a German Former Right-Wing Extremist’s YouTube Channel for Preventing and Countering Violent Extremism and Crime. Journal for Deradicalization (29), 129-176.

Garadian, E. A. (2018). Youth Camp for Preventing Violent-Extremism: Fostering Youth Dialogue, Encountering Diversity. Studia Islamika, 25(2), 423.

Garaigordobil, M. (2012). Evaluation of a program to prevent political violence in the Basque conflict: Effects on the capacity of empathy, anger management and the definition of peace. Gaceta Sanitaria, 26(3), 211-216.

Gavrilovici, O., & Dronic, A. (2020). Increasing professionals' knowledge and skills to deal with violent radicalisation. In P. Meringolo (Eds.). Preventing Violent Radicalisation in Europe. Multidisciplinary Perspectives (pp. 109-133). Springer.

Gereluk, D., & Titus, C.-A. (2018). How Schools Can Reduce Youth Radicalization. Solsko Polje, 29(5/6), 33-50,155.

Ghosh, R. (2018). The potential of the ERC program for combating violent extremism among youth. Religion and Education, 45(3), 370-386.

Ghosh, R., Chan, W. Y. A., Manuel, A., & Dilimulati, M. (2017). Can education counter violent religious extremism? Canadian Foreign Policy Journal, 23(2), 117-133.

Greiner, K. (2010). Applying Local Solutions to Local Problems: Radio Listeners as Agents of Change. USAID.

Grossman, A. N., Nomikos, W. G., & Siddiqui, N. A. (2023). Can Appeals for Peace Promote Tolerance and Mitigate Support for Extremism? Evidence from an Experiment with Adolescents in Burkina Faso. Journal of Experimental Political Science, 10(1), 124-136.

Gurlesin, O., Akdag, M., Alasag, A., & Ter Avest, I. (2020). Playful religion: An innovative approach to prevent radicalisation of Muslim youth in Europe. Religions, 11(2).

Hamid, A. (2020). RADICALISM PREVENTION THROUGH ISLAMIC RELIGIOUS EDUCATION LEARNING AT ELEMENTARY SCHOOL. Jurnal Pendidikan Islam, 6(1), 109-126.

Hankir, A., Carrick, F. R., & Zaman, R. (2017). Part I: Muslims, social inclusion and the West. Exploring challenges faced by stigmatized groups. Psychiatria Danubina, 29(Suppl 3), 164-172.

Harris-Hogan, S., Barrelle, K., & Smith, D. (2019). The role of schools and education in countering violent extremism (CVE): applying lessons from Western countries to Australian CVE policy. Oxford Review of Education, 45(6), 731-748.

Helmus, T. C., York, E., & Chalk, P. (2013). Promoting Online Voices for Countering Violent Extremism. RAND Corporation.

Hiariej, E., Rachmawati, A. D., Taek, A. M., Kurniasari, M., & Alvian, R. A. (2017). Final Evaluation Reducing the Recruitment and Recidivism of Violent Extremists in Indonesia. Search for Common Ground.

Hirschi, C., & Widmer, T. (2012). Approaches and challenges in evaluating measures taken against right-wing extremism. Evaluation and Program Planning, 35(1), 171-179.

Horgan, J. G., Williams, M. J., Evans, W. P., & Belanger, J. J. (2017). Assessment Report: Current Capabilities of 2‐1‐1 Call Centers and Local Service Providers.

Hulse, T. J. J., & Moeyens, C. (2022). Shared Endeavour Fund Call One Evaluation Report.

Hunter, R. (2016). Difficult Terrain and Unreported Successes: Young People and Community-Based Restorative Justice in Northern Ireland. Irish Probation Journal, 13, 175-193.

Iacopini, G., Stock, L., & Junge, K. (2011). Evaluation of Tower Hamlets Prevent Projects. The Tavistock Institute.

Ibragimov, I. D., Neif, N. M., Nikolaeva, Y. V., Demina, S. V., Fedorchuk, Y. M., Morozov, A. V., & Selezneva, N. A. (2018). Strategy And Tactics Of Students Readiness Formation To Counter Cyber Extremist Activities. Modern Journal of Language Teaching Methods, 8(5), 149-165.

Ibrahim, A. (2010). Tackling Muslim Radicalization: Lessons from Scotland. Institute for Social Policy and Understanding.

Ilisko, D. (2016). Building Bridges over Troubled Waters or Learning "to Live Together" Sustainably. Conference on Religious Education between Radicalism and Tolerance, 187-201.

Institute for Community Cohesion (2007) Young People and Extremism: Some Reflections from Our Local Studies.

International Alert (2018). Making PVE Programmes Work. International Alert.

Ipp, O., Prado, A., & Fourati, Y. A. (2014). Tunisia Transition Initiative (TTI) Final Evaluation Report. USAID

Ipsos MORI (2021). Evaluation of the Building a Stronger Britain Together (BSBT) programme. Home Office.

Ipsos MORI. (2019). Building a Stronger Britain Together (BSBT) Progress Report 2019 Interim evaluation findings.

Iqbal, K., Zafar, S. K., & Mehmood, Z. (2019). Critical evaluation of Pakistan’s counter-narrative efforts. Journal of Policing, Intelligence and Counter Terrorism, 14(2), 147-163.

Ishaku, B., Aksit, S., & Maza, K. D. (2021). The role of faith-based organizations in counter-radicalization in Nigeria: The case of Boko Haram. Religions, 12(11), 1003.

Izzi, V. (2013). Just Keeping Them Busy? Youth Employment Projects as a Peacebuilding Tool’. International Development Planning Review, 35(2), 103-117.

Jailobaev, T., Jailobaeva, K., Baialieva, G., Asilbekova, G., & Eshmuratova, Z. (2022). FINAL EVALUATION REPORT FOR THE “PREVENTION OF VIOLENT EXTREMISM IN CENTRAL ASIAN COUNTRIES THROUGH STRENGTHENING SOCIAL COHESION AMONG LABOUR MIGRANTS, RETURNEES, AND THEIR FAMILIES” PROJECT.

Jailobaeva, K. (2020). Research on the Role of Educational Institutions in Building Resilience of Adolescents to Radicalisation and Violent Extremism in the Kyrgyz Republic. Hedayah.

Jerome, L., & Elwick, A. (2016). Evaluation report on the ACT Building Resilience Project Full Report. Middlesex University and Association for Citizenship Teaching (ACT).

Johansson, S. (2013). Innovative Methods and Models of Collaboration in the Field of Pedagogical Prevention of Xenophobia, Anti-Semitism and Right-wing Extremism: Chances and Perspectives for a Better Cooperation between Formal and Non-formal Education in Germany German Youth Institute. Socialinė Teorija, Empirija, Politika Ir Praktika, 7, 119-132.

Johns, A., Grossman, M., & McDonald, K. (2014). “More than a game”: The impact of sport-based youth mentoring schemes on developing resilience toward violent extremism. Social Inclusion, 2(2), 57-70.

Johnson-Lafleur, J., Zoldan, Y., Frounfelker, R. L., & Rousseau, C. (2023). Collective case formulation in situations of violent radicalization: A critical perspective in training. Transcultural Psychiatry, 60(2), 302-312.

Josefsson, T., Nilsson, M., & Borell, K. (2017). Muslims Opposing Violent Radicalism and Extremism: Strategies of Swedish Sufi Communities. Journal of Muslim Minority Affairs, 37(2), 183-195.

Khalil, J., & Ipp, O. (2016). USAID/OTI PDQIII Task Order #10, Activity #3 Mali Transition Initiative: Final Evaluation. USAID.

Khalil, J., & Zeuthen, M. (2014). Qualitative Study on Countering Violent Extremism (CVE) Programming Under the Kenya Transition Initiative (KTI). USAID.

Khan, S. (2019). Challenging Hateful Extremism: Commission for Countering Extremism report on extremism in England and Wales. Commission for Countering Extremism.

Kimaiyo, T., Suleiman, K., & Zeuthen, M. (2023). Lessons Learned from Student-led Initiatives to Prevent Violent Extremism in Kenyan Universities. RUSI.

Kirana, D. (2018). Enhancing Religious Education: An Attempt to Counter Violent Extremism in Indonesia. Studia Islamika, 25(1), 199.

Klapp, M. (2018). Salam-Online: Preventive Measures against extreme online messages among Muslims in Germany. Insights into a pilot project at the Center for Islamic Theology, Münster. Lodz Papers in Pragmatics, 14(1), 181-201.

Koehler, D., & Ehrt, T. (2018). Parents’ Associations, Support Group Interventions and Countering Violent Extremism: An Important Step Forward in Combating Violent Radicalization. International Annals of Criminology, 56(1-2), 178-197.

Kollmorgen, J. C., & Barry, C. (2017). Evaluation Report Ex-Post Performance Evaluation of USAID/RDMA Sapan Program. USAID.

Kollmorgen, J. C., Ogada, M., Korir, S., & Dena, E. (2019). Strengthening Community Resilience Against Extremism (SCORE).

Kurtz, J., Tesfaye, B., & Wolfe, R. (2018). Can Economic Interventions Reduce Violence? Impacts of Vocational Training and Cash Transfers on Youth Support for Political Violence in Afghanistan. Mercy Corps.

Kurtz, J., Wolfe, R., & Tesfaye, B. (2016). Does Youth Employment Build Stability? Evidence from an Impact Evaluation of Vocational Training in Afghanistan. In S. Zeiger & A. Aly (Eds.). Expanding Research on Countering Violent Extremism. Hedayah.

Lahmann, H. (2021). “Afghanistan Is Darkness and Light”: Creative Arts for Youth and Peace in Afghanistan. PhD Thesis, New York University.

Lamhaidi, N. (2017). Women’s Caravan for Peace Final Evaluation. Search for Common Ground.

Langer, A., Kaufhold, M.-A., Runft, E. M., Reuter, C., Grinko, M., & Pipek, V. (2019). Counter Narratives in Social Media: An Empirical Study on Combat and Prevention of Terrorism. ISCRAM.

Lawale, S. (2020). Preventing and Countering Violent Extremism Through Education: A Vital Element for the Peace Process in the Gulf. Asian Journal of Middle Eastern and Islamic Studies, 14(1), 98-120.

Lee, B. J. (2020). Informal countermessaging: The potential and perils of informal online countermessaging. In M. Conway & S. Macdonald (Eds.). Islamic State’s Online Activity and Responses (pp. 161-177). Routledge.

Letsch, L. (2018). Countering violent extremism in Tunisia–between dependency and self-reliance. Journal for Deradicalization (17), 163-195.

Levy, D., Jamankulov, K., & Sartbay, T. (2019). Project Evaluation #JashStan: Youth as Agents of Peace and Stability in Kyrgyzstan.

Liht, J., & Savage, S. (2013). Preventing Violent Extremism through Value Complexity: Being Muslim Being British. Journal of Strategic Security, 6(4), 44-66.

Lippe, F., Walter, R., & Hofinger, V. (2022). Evaluating an online-game intervention to prevent violent extremism. Journal for Deradicalization (32), 1-34.

Lund, D. E. (2006). Social justice activism in the heartland of hate: Countering extremism in Alberta. Alberta Journal of Educational Research, 52(2).

Macaluso, A. (2016). From Countering to Preventing Radicalization Through Education: Limits and Opportunities. The Hague Institute for Global Justice.

Macnair, L., & Frank, R. (2017). Voices Against Extremism: A case study of a community-based CVE counter-narrative campaign. Journal for Deradicalization, 10, 147-168.

Makbuloh, D. (2019). The <i>Asah Potential Fitrah</i> Method in Encountering Radicalism in Lampung. Pertanika Journal of Social Science and Humanities, 27(2), 937-947.

Management Systems International (2017). Innovative Approaches to CVE Programming: Insights and Lessons From the USAID OTI Lebanon Community Resilience Initiative.

Mansour, S. (2017). The Morocco Transforming Violent Extremism Media Training Program Final External Evaluation. Search for Common Ground.

Mbugua, P. K., & Ayoub, N. T. (2023). Religious Peacebuilding’s Response to Violent Extremism in Informal Settlements in Egypt. The Palgrave Handbook of Religion, Peacebuilding, and Development in Africa, 455-473.

McGinn, G. (2021). The Adaptation of the Education Profession in Identifying Radicalisation in Schools: A Review of the Evidence and Observations from a Cohort of Educators and Prevent Practitioners. Masters' Thesis, Canterbury Christ Church University.

Meinema, E. (2020). 'Idle minds' and 'empty stomachs': Youth, violence and religious diversity in coastal Kenya. Africa, 90(5), 890-913.

Memarzia, M. & Star, K.(2011). Choices and Voices - A Serious Game for Preventing Violent Extremism. In B. Akhgar & S. Yates (Eds.). Intelligence Management: Knowledge Driven Frameworks for Combating Terrorism and Organized Crime (pp. 133-142). Springer.

Menendez-Ferreira, R., Torregrosa, J., Panizo-Lledot, A., Gonzalez-Pardo, A., & Camacho, D. (2020). Improving Youngsters' Resilience through Video Game-Based Interventions. Vietnam Journal of Computer Science, 7(3), 263-279.

Mercy Corps (2012). Support for Kosovo Young Leaders (SKYL): End of Program Evaluation. Mercy Corps.

Mercy Corps (2013). Examining the Links Between Youth Economic Opportunity, Civic Engagement, and Conflict: Evidence from Mercy Corps’ Somali Youth Leaders Initiative. Mercy Corps

Mercy Corps (2015). USAID Somalia, Somali Youth Leaders Initiative (SYLI) Quarterly Progress Report Quarter III, FY 2015. USAID.

Mercy Corps. (2015). Does youth employment build stability? Evidence from an impact evaluation of vocational training in Afghanistan. Mercy Corps.

Mercy Corps. (2016). USAID Somalia, Somali Youth Leaders Initiative (SYLI) Quarterly Progress Report, Quarter II, FY 2016. USAID.

Merkel, K. & Hulse., T. (2022). MULTI-STAKEHOLDER MODELS FOR LOCAL PREVENTION: Technical Evaluation and Learnings from Kumanovo, North Macedonia.

Miller, H., Tayeb, R., & Welland, L. (2020). Preventing Violent Extremism Through Mental Health Promotion: An Evaluation of a Public Health Approach. REACHOUT Australia.

Millett, K., & Ahmad, F. (2021). Echoes of Terror(ism): The Mutability and Contradictions of Countering Violent Extremism in Québec. Canadian Social Studies, 52(2), 52-67.

Mirahmadi, H. (2016). Building resilience against violent extremism: A community-based approach. The ANNALS of the American Academy of Political and Social Science, 668(1), 129-144.

Moffett, K., & Sgro, T. (2016). School-based CVE strategies. Annals of the American Academy of Political and Social Science, 668(1), 145-164.

Monaci, S. (2020). Social Media Campaigns Against Violent Extremism: A New Approach to Evaluating Video Storytelling. International Journal of Communication, 14(1), 980-1003.

Monzani, B., Sarota, A., & Venturi, B. (2018). Evaluation Report Inuka! Community-Led Security Approaches to Violent Extremism in Coastal Kenya. Search for Common Ground and Agency for Peacebuilding.

Moyano, M., Lobato, R. M., Blaya-Burgo, M., Arnal, N., Cuadrado, E., Mateu, D., Ramírez-García, A., de Murga, M., & Trujillo, H. M. (2022). Preventing violent extremism in youth through sports: An intervention from the 3N model. Psychology of Sport and Exercise, 63.

Muncy, D., David, R., & Saleh, B. (2015). Training of Leaders on Religious and National Co-Existence (TOLERANCE) Project Mid-term Evaluation Report. USAID.

Murtaza, N., Sohail, A., Perveen Shaikh, R., Ahmed, S., Anver, S., Ahmad, A., Asghar, M., Ashraf, J., & Yar Khan, U. (2018). Punjab Youth Workforce Development Project Midterm Evaluation. USAID

Nafi’a, I., Gumiandari, S., Hakim, M. A., Safii, S., & Rokhmadi, R. (2022). Mitigating radicalism amongst Islamic college students in Indonesia through religious nationalism. HTS Teologiese Studies / Theological Studies, 78(4).

Nash, C., Nesterova, Y., Primrose, K., Chan, W. Y. A., Rios, R. A., Flores, M. J. V., & Zowmi, A. (2018). #Youth Waging Peace. Action Guidelines for the Prevention of Violent Extremism: Reflection is Action, Conversation is Action, Compassion is Action. In.

Nasih, A. M., Darwis, & Hamid, A. (2023). Countering Islamic Radicalism among Indonesian University Students: An Investigation on Social Media Using LINE Official Account. Journal of Al-Tamaddun, 18(1), 179-192.

National Institute of Justice (2017). An Evaluation of a Community-Based Program to Counter Violent Extremism Leads to New Measures of Effectiveness That Could Aid Future Evaluations. National Institute of Justice

Nešković, T. (2019). Preliminary Findings: Evaluation of a Pilot Project on “Countering Violent Extremism in the Regions of Maghreb and Sahel. UN Interregional Crime and Justice Research Institute.

Ngaruko, D. D. (2021). Final Evaluation Report: KWA PAMOJA TUDUMISHE AMANI PROJECT - WORKING TOGETHER TO REDUCE VIOLENT EXTREMISM THREATS IN PWANI AND MTWARA REGIONS IN TANZANIA.

Njoku, E. T. (2021). State-oriented service-delivery partnership with civil society organizations in the context of counter-terrorism in Nigeria. Development Policy Review, 39(5), 757-772.

Nordbruch, G. (2016). The Role of Education in Preventing Radicalisation. RAN Centre of Excellence.

Octavia, L., & Wahyuni, E. (2014). Final Evaluation Report For the Project: Countering & Preventing Radicalization in Indonesian Pesantren. Search for Common Ground.

Odjidja, E. N., Ayaluri, S. S., & Fiodorov, D. (2024). Do Livelihood Interventions Contribute to the Prevention of Violent Extremism? The RUSI Journal, 169(3), 52-66.

OPM. (2010). Research into Best Practice in Preventing Violent Extremism and Understanding the Causes of Violent Extremism.

Osae Ihidero, D. (2020). An Appraisal of ‘Facebook’s Global Peer-to-Peer Campaign on Challenging Violent Extremism’ in Nigeria: The SOAR Peer-to-Peer Project. Journal of English Literature and Cultural Studies, 1(1), 41-48.

Parker, D., & Lindekilde, L. (2020). Preventing extremism with extremists: A double-edged sword? An analysis of the impact of using former extremists in Danish schools. Education Sciences, 10(4), 1-19.

Parker, L., Boyer, I., & Gatewood, C. (2018). Young Digital Leaders Impact Report. Institute for Strategic Dialogue.

Payne, M. A. (2017). Toujours Le Choix: The Role of Entertainment Education in Radicalization Prevention. The American University of Paris.

Pipe, R., Egal, J., Malla, L., Billow, Z., & Abdi, A. (2016). Somalia Program Support Services Final Performance Evaluation of the Transition Initiatives for Stabilization Project. USAID and International Business & Technical Consultants, Inc.

Rahlf, L. (2024). Participatory Theatre for Preventing Violent Extremism Through Education-Reflections on Prospects and Preconditions. Journal for Deradicalization (39), 77-93.

Rahman, S. F. A., & Gaber, G. F. (2024). A PROGRAM BASED ON RATIONAL-EMOTIONAL GUIDANCE IN DEVELOPING DIGITAL CITIZENSHIP AND REDUCING EXTREMIST THOUGHTS AMONG UNIVERSITY FEMALE STUDENTS. Revista Gestao & Tecnologia-Journal of Management and Technology, 24(2), 7-30.

Ravizza, D. M. (2021). Countering the trajectories towards new forms of violence in youth: Enhancing protective factors through sports. In M. Lang (2021) Routledge Handbook of Athlete Welfare (pp. 263-276). Routledge.

Reeves, J., & Sheriyar, A. (2015). Addressing Radicalisation into the Classroom - A New Approach to Teacher and Pupil. Learning Journal of Education and Training, 2(2), 20-39.

Reynolds, L., & Scott, R. (2016). Digital Citizens: Countering Extremism Online. Demos.

Rhoades, A. L., Helmus, T. C., Marrone, J. V., Smith, V. M., & Bodine-Baron, E. (2020). Promoting Peace as the Antidote to Violent Extremism: Evaluation of a Philippines-Based Tech Camp and Peace Promotion Fellowship. RAND Corporation.

Rizal, Educ, Univ Tadulako Palu, M. C., Univ Tadulako, P. I., & Univ Tadulako Palu, F. T. T. (2017). Importance of Application of Dialogical Learning Approach Efforts to Prevent Radicalism. 1st Indonesian Communication Forum of Teacher Training and Education Faculty Leaders International Conference on Education (ICE), 174, 387-391.

Robinson, D., Reeve, K., Platts-Fowler, D., Green, S., Walshaw, A., Batty, E., Bashir, N., Pearce, S., & Eadson, W. (2013). An Evaluation of Getting on Together: The Community Cohesion Strategy for Wales.

Romanova, T. V. (2020). Pedagogical Prevention Technology of Extremism Manifestations in Youths Based on Social Competencies. 6th International Forum on Teacher Education (IFTE), 2081-2099.

Rulison, K., Weaver, G., Milroy, J., Beamon, E., Kelly, S., Ameeni, A., Juma, A., Abualgasim, F., Husain, J., & Wyrick, D. (2024). Using the Preparation Phase of the Multiphase Optimization Strategy to Design an Antiextremism Program in Bahrain: Formative and Pilot Research. JMIR Formative Research, 8, e58322.

Sabic-El-Rayess, A., Joshi, V., & Hruschka, T. (2023). Building resilience to hate in classrooms: Innovation in practice and pedagogy to prevent extremism and violence in U.S. schools. Journal of Prevention & Intervention in the Community, 51(4), 313-331.

Saleh, N. F., Roozenbeek, J. O. N., Makki, F. A., McClanahan, W. P., & Van Der Linden, S. (2024). Active inoculation boosts attitudinal resistance against extremist persuasion techniques: A novel approach towards the prevention of violent extremism. Behavioural Public Policy, 8(3), 548-571.

Saltman, E. M., Dow, M., & Bjornsgaard, K. (2016). Youth innovation labs: A model for preventing and countering violent extremism. Institute for Strategic Dialogue, 1-44.

Samuel, T. K. (2022). Education in Preventing and Countering Violent Extremism (P/CVE) Among the Youth. In A.J. Masys (Ed.). Handbook of Security Science (pp. 1039-1062), Springer.

Sandford, L. (2017). Exploring the capabilities of Prevent in addressing radicalisation in cyberspace within Higher Education. PhD Thesis, University of Gloucestershire

Sarota, A. (2017). Baseline Evaluation of: Katika Usalama Tunategemeana and Pamoja! Strengthening Community Resilience in Tanzania. Search for Common Ground.

Sarwar, M., Ul Hassan, M., & Pervaiz, M. (2023). Effectiveness Of Narrative Therapy In Promoting Students' Resilience To Violent Extremism. Journal of Namibian Studies, suppl. Special Issue 1, 34, 1.

Savage, S., Oliver, E., Gordon, E., & Tutton, L. (2021). Addressing social polarization through critical thinking: Theoretical application in the "Living Well With Difference" course in secondary schools in England. Journal of Social and Political Psychology, 9(2), 490-505.

Savoia, E. (2020). Evaluation of the Peer to Peer (P2P) Challenging Extremism Initiative.

Savoia, E., McBride, M., Stern, J., Su, M., Harriman, N., Aziz, A., & Legault, R. (2020). Assessing the Impact of the Boston CVE Pilot Program: A Developmental Evaluation Approach. Homeland Security Affairs, 16.

Savoia, E., Su, M., Harriman, N., & Testa, M. A. (2019). Evaluation of a school campaign to reduce hatred. Journal for Deradicalization (21), 43-83.

Schorn, F., Moubayed, L., & Auten, S. (2010). Review of the Office of Middle East Programs Youth Initiatives. USAID and Aguirre Division of JBS International, Inc.

Schulten, N., Vermeulen, F. F., & Doosje, B. (2020). Preventing polarization: An empirical evaluation of a dialogue training. Cogent Social Sciences, 6(1).

Schumicky-Logan, L. (2017). Addressing violent extremism with a different approach: The empirical case of at-risk and vulnerable youth in Somalia. Journal of Peacebuilding and Development, 12(2), 66-79.

SecDev Foundation. (2016). Extreme Dialogue: Social Media Target Audience Analysis and Impact Assessments in Support of Countering Violent Extremism. An abridged summary report of findings and lessons learned SecDev Foundation and The Institute for Strategic Dialogue.

Sheikh, S. S. S., & King, E. (2012). Evaluation of the Muslim Council of Wales’ Prevent work (Research Summary No. 23).

Sheikh, S., Sarwar, S., & King, E. (2012). Evaluation of the Muslim Council of Wales' Prevent Work.

Sjøen, M. M. (2019). When counterterrorism enters the curriculum: How the global war on terror risks impairing good education. Journal for Deradicalization (20), 156-189.

Sklad, M., Irrmischer, M., Park, E., Versteegt, I., Wignand, J. (2021). Perspective Taking Skills and Conflict Resolution. In: Social and Civic Competencies Against Radicalization in Schools. Palgrave Macmillan.

Smith, P. (2008). Prevention of Violent Extremism Project Report. Criminal Justice, 1-62.

Spalek, B. (2013). Counter-terrorism: police and community engagement in Britain: Community-focused or community-targeted? In R. Gunaratna, J. Jerard & S. M. Nasir (Eds.) Countering Extremism: Building Social Resilience through Community Engagement (pp. 57–71). Imperial College Press.

Speckhard, A., Ellenberg, M., & Ahmed, M. (2020). Jihad is our Way: Testing a Counter Narrative Video in Two Somali American Focus Groups. Bildhaan: An International Journal of Somali Studies, 20(1), 8.

Speckhard, A., Ellenberg, M., Shaghati, H., & Izadi, N. (2020). Hypertargeting Facebook profiles vulnerable to ISIS recruitment with" Breaking the ISIS brand counter narrative video clips" in multiple Facebook campaigns. Journal of Human Security, 16(1), 16-29.

SPLC & PERIL (2023). Building Networks & Addressing Harm: A Community Guide to Online Youth Radicalization Impact Study.

Stahl, G., Baak, M., Schulz, S., & Peterson, A. (2023). A cautionary tale: CVE policy actors managing risk in Australian schools. Current Sociology, 71(5), 905-923.

Stanley, T. (2018). Side by Side (the Prevent Duty Online Modules for Learners across the Further Education and Training Sector) by the Education & Training Foundation, London, 2017.

Sumbulah, U. (2017). De-radicalisation of Indonesian Students: A Case Study of UIN Malang. Pertanika Journal of Social Science and Humanities, 25, 155-164.

Supratno, H., Subandiyah, H., Raharjo, R. P., Univ Negeri Surabaya, S. I., & Univ Negeri, S. (2018). Character Education in Islamic Boarding School as a Medium to Prevent Student Radicalism. 2nd Social Sciences, Humanities and Education Conference - Establishing Identities through Language, Culture, and Education, 222, 405-410.

Svennevig, H., Jerome, L., & Elwick, A. (2021). Countering Violent Extremism in Education: A Human Rights Analysis. Human Rights Education Review, 4(1), 91-110.

Swedberg, J. (2011). Mid-Term Evaluation of USAID’s Counter-Extremism Programming in Africa. USAID.

Swedberg, J., & Reisman, L. (2013). Mid-Term Evaluation of Three Countering Violent Extremism projects. USAID

Taylor, B., Mills, M., Elwick, A., Pillinger, C., Gronland, G., Hayward, J., Hextall, I., & Panjwani, F. (2021). Addressing Extremism through the Classroom: A Research Report from the Centre for Teachers & Teaching Research.

Taylor, E., Taylor, P. C., Karnovsky, S., Aly, A., & Taylor, N. (2017). "Beyond Bali": A transformative education approach for developing community resilience to violent extremism. Asia Pacific Journal of Education, 37(2), 193-204.

Tesfaye, B., McDougal, T., Maclin, B., & Blum, A. (2018). If Youth Are Given the Chance: Effects of Education and Civic Engagement on Somali Youth Support of Political Violence. Mercy Corps.

Thomas, P. (2008). Kirklees ‘Preventing Violent Extremism’ Pathfinder: Issues and Learning from the First Year.

Tiflati, H. (2016). Western Islamic schools as institutions for preventing behavioral radicalization: The case of Quebec. Journal for Deradicalization, 6(1), 180-205.

Tines, J., Siddiqui, H., ul, N., Akhtar, N., Sadiq, M., Tanveer, T., & Iqbal Zaidi, S. Z. (2017). Karachi Youth Workforce Development Project: Midterm Evaluation Report. USAID.

Ullah, Z. (2023). Final Evaluation Report: Youth for Humanity.

USAID (2019). USAID’s Mindanao Youth for Development (MYDev) Program FY17 Impact Evaluation Report & FY18|19. USAID.

Vicente, P. C., & Vilela, I. (2021). Preventing violent Islamic radicalization: experimental evidence on anti-social behavior. Working Paper of the Helen Kellogg Institute for International Studies, 2021(445).

Vicente, P. C., & Vilela, I. (2022). Preventing Islamic radicalization: Experimental evidence on anti-social behavior. Journal of Comparative Economics, 50(2), 474-485.

Wachs, S., Krause, N., Wright, M. F., & Gámez-Guadix, M. (2023). Effects of the prevention program “HateLess. Together against hatred” on adolescents’ empathy, self-efficacy, and countering hate speech. Journal of Youth and Adolescence, 52(6), 1115-1128.

Walsh, M., & Gansewig, A. (2019). A former right-wing extremist in school-based prevention work: Research findings from Germany. Journal for Deradicalization (21), 1-42.

Whittaker, J., & Elsayed, L. (2019). Linkages as a lens: An exploration of strategic communications in P/CVE. Journal for Deradicalization, 20, 1-46.

Wildan, M. (2022). Countering Violent Extremism in Indonesia: The Role of Former Terrorists and Civil Society Organisations. In G. Barton, M. Vergani, Y. Wahid (Eds.) Countering Violent and Hateful Extremism in Indonesia. New Security Challenges (pp. 195-214). Springer.

Williams, M. J., Horgan, J. G., & Evans, W. P. (2016). Evaluation of a multi-faceted, US community-based, Muslim-led CVE program.

Wilner, A., & Rigato, B. (2017). The 60 days of PVE campaign: Lessons on organizing an online, peer-to-peer, counter-radicalization program. Journal for Deradicalization (12), 227-268.

Wilson, N. L., & Krentel, J. (2018). Lessons from Strengthening Capacity in Countering Violent Extremism. In: United States Institute of Peace.

Winston, J., & Strand, S. (2013). Tapestry and the aesthetics of theatre in education as dialogic encounter and civil exchange. Research in Drama Education, 18(1), 62-78.

Winter, C., & Fürst, J. (2017). Challenging Hate: Counter-speech Practices in Europe. In: The International Centre for the Study of Radicalisation and Political Violence.

Wong, K., Walton, G., & Bailey, G. (2021). Using information science to enhance educational preventing violent extremism programs. Journal of the Association for Information Science and Technology, 72(3), 362-376.

Zeiger, S., Mattei, C., & Nettleton, L. (2019). Preventing Violent Extremism Through Education in Uganda. Hedayah.

**Exclusion: Outcomes - No Eligible Data Relevant to Objectives**

Agbiboa, D. E. (2015). Youth As Tactical Agents of Peacebuilding and Development in the Sahel. Journal of Peacebuilding and Development, 10(3), 30-45.

Challgren, J., Kenyon, T., Kervick, L., Scudder, S., Walters, M., Whitehead, K., & Flynn, C. R. (2016). Countering violent extremism: Applying the public health model. Georgetown University.

Desmarais, C., & Rousseau, C. (2024). Self-representation and future perception of youth followed by a specialized intervention team in violent radicalization. International Journal of Applied Psychoanalytic Studies, 21(1).

Ghazi, M. (2024). Women, Minors and Families Affiliated with Terrorist Groups: Reintegration and Peacebuilding in Morocco. In N. Käsehage (Eds.). Keeping Peace in Troubled Times: Perspectives from Different Disciplines on War and Peace (pp. 209-229). Springer Nature.

Gustafson, S. (2020). Moving toward the enemy: A case for missiological engagement in counter/deradicalization. Journal for Deradicalization (25), 117-157.

Harahap, H. I., & Irmayani, M. T. (2017). The Phenomenon of De-radicalization in Al Hidayah Boarding School, North Sumatra Province, Indonesia. 3rd International Conference on Social and Political Sciences (ICSPS), 129, 5-8.

Harahap, H. I., Irmayani, T., & Lubis, F. H. (2019). The Rationality of De-radicalization Efforts for the Children of Terrorists at Al-Hidayah Islamic Boarding School. International Journal of Islamic Thought, 16, 38-50.

Helmus, T. C., & Klein, K. (2018). Assessing Outcomes of Online Campaigns Countering Violent Extremism: A Case Study of the Redirect Method. RAND Corporation.

Hulse, T. & Williams, M. J. (2024). Shared Endeavour Fund: Call Three Evaluation Report. ISD.

Kudlacek, D., Phelps, M., Fleischer, S., Treskow, L., Ehimen, E., Hemert, D., Hueting, T., Kraus, B., Labuda, K., Llinares, F. M., Lortal, G., Marsh, B., Ortuño, R. B., Purcell, S., Pastuovic, M. H., Rooze, M., Castro Toledo, F. J., Wagner, D., & Görgen, T. (2017). Prevention of radicalization in selected European countries: A comprehensive report of the state of the art in counterradicalisation. Kriminologisches Forschungsinstitut Niedersachsen.

Kudlacek, D., Phelps, M., Fleischer, S., Treskow, L., Grätsch, E. I., Ehimen, E., Hemert, D. A. v., Hueting, T., Kraus, B., & Labuda, K. (2020). Prevention of Radicalisation in Selected European Countries: A comprehensive report of the state of the art in counter-radicalisation.

Lindekilde, L. (2012). Value for money? Problems of impact assessment of counter-radicalisarion policies on end target groups: The case of Denmark. European Journal on Criminal Policy and Research, 18(4), 385-402.

Lohlker, R. (2019). Emerging Alternatives and Participatory Action Research: Report on the Video Project “Jamal al-Khatib.”. K. Limacher, A. Mattes, & C. Novak (Eds.). Prayer, Pop and Politics: Researching Religious Youth in Migration Society (pp. 145-160). V&R Unipress.

Mesok, E. (2022). Beyond instrumentalisation: gender and agency in the prevention of extreme violence in Kenya. Critical Studies on Terrorism, 15(3), 610-631.

Moonshot. (2021). Canada Redirect – Final Report.

Moonshot. (2023). Countering Radicalization to Violence in Ontario and Quebec: Canada’s First Online-Offline Interventions Model.

Moonshot. (2023). SCREEN Hate: National findings report.

Muhammad, A., & Hiariej, E. (2021). Deradicalization program in Indonesia radicalizing the radicals. Cogent Social Sciences, 7(1), 1905219.

Nilsson Lundmark, E., & Nilsson, I. (2014). A SUMMARY OF THE PRICE OF INTOLERANCE A Socioeconomic Analysis of the White Supremacy Movement and the Kungälv Model.

R.T.I International (2021). OFFICE FOR TARGETED VIOLENCE AND TERRORISM PREVENTION (OTVTP) FY2016 GRANT EVALUATIONS.

Rabasa, A., Pettyjohn, S. L., Ghez, J., & Boucek, C. (2010). Deradicalizing Islamist extremists. RAND Corporation.

Savage, S., & Fearon, P. A. (2021). Increasing cognitive complexity and meta-awareness among at-risk youth in Bosnia-Herzegovina in order to reduce risk of extremism and interethnic tension. Peace and Conflict: Journal of Peace Psychology, 27(2), 225-239.

Savoia, E., Testa, M. A., Stern, J., Lin, L., Konate, S., & Klein, N. (2016). Evaluation of the Greater Boston Countering Violent Extremism (CVE) Pilot Program. Harvard T.H. Chan School of Public Health.

Sivenbring, J. (2019). Signs of concern about Islamic and right-wing extremism on a helpline against radicalization. Journal for Deradicalization (18), 108-145.

Sumbulah, U. (2019). Preventing radicalism by family and civil society organizations in Indonesia. Pertanika Journal of Social Sciences and Humanities, 27(1), 391-403.

Sumpter, C. (2017). Countering violent extremism in Indonesia: priorities, practice and the role of civil society. Journal for Deradicalization.

van Heelsum, A., & Vermeulen, F. (2018). Cities' Policies: The Work of European Cities to Counter Muslim Radicalisation. Journal of International Migration and Integration, 19(1), 161-179.

Weisburd, D., Wolfowicz, M., Hasisi, B., Paulucci, M., & Andrighetto, G. What is the best approach for preventing recruitment to terrorism?: Findings from ABM experiments in social and situational prevention. Criminology and Public Policy, 21(2).

Yayla, A. S. (2020). Preventing Terrorist Recruitment through Early Intervention by Involving Families. Journal for Deradicalization (23), 134-188.

**Exclusion: Isolating Relevant Findings**

Aiello, E., Puigvert, L., & Schubert, T. (2018). Preventing violent radicalization of youth through dialogic evidence-based policies. International Sociology, 33(4), 435-453.

Ambrozik, C. (2018). Countering Violent Extremism Locally. PhD Thesis, Cornell University

Badurdeen, F. A., & Goldsmith, P. (2018). Initiatives and perceptions to counter violent extremism in the coastal region of Kenya. Journal for Deradicalization.

Blanchfield, D. (2021). A Contemporary Insight into Islamist Extremist Radicalisation and an Exploration of the Effectiveness of Prevention and Reformative Strategies. Masters Thesis, Canterbury Christ Church University.

Hulse, M. J. W., & Tim. (2023). SHARED ENDEAVOUR FUND: Call Two - Evaluation Report. Institute for Strategic Dialogue.

Kundnani, A. (2009). Spooked! How not to prevent violent extremism. Institute of Race Relations.

Lehane, O. (2019). Dealing with Frustration: A Grounded Theory Study of CVE Practitioners. International Journal of Conflict and Violence (IJCV), 12(0), a641.

Lehane, O. (2019). Mining the Personal to Carve a Space of One's Own: A Grounded Theory Study of Grassroots Countering Violent Extremism Practitioners. PhD Thesis, Dublin City University.

Puigvert, L., Aiello, E., Oliver, E., & Ramis-Salas, M. (2020). Grassroots community actors leading the way in the prevention of youth violent radicalization. PLoS ONE, 15(10), e0239897.

Salyk-Virk, M. J. (2020). Building community resilience? Community perspectives of the countering violent extremism pilot program in Minneapolis/St. Paul. Studies in Conflict & Terrorism, 43(11), 1011-1042.

Wise, P., Roberts, S., Formosa, J., & Chan, A. (2018). Evaluation of the COMPACT program. New South Wales: Urbis.

**Exclusion: Ineligible Document**

Bjorgo, T. (2002). Racist Groups: Reducing Recruitment and Promoting Disengagement. Journal fur Konflikt- und Gewaltforschung, 4(1), 5-31.

Bouzar, D. (2017). A novel motivation-based conceptual framework for disengagement and de-radicalization programs. Sociol Anthropol, 5(8), 600-614.

Bouzar, D., & Bénézech, M. (2019). Protection factors and risk factors for disengaging from jihadist violent extremism: Studies on the variables “of becoming” of 450 Jihadists. Journal de Medecine Legale Droit Medical, 62(3), 3-25.

Bouzar, D., & Laurent, G. (2019). The importance of interdisciplinarity to deal with the complexity of the radicalization of a young person. Annales Medico-Psychologiques, 177(7), 663-674.

BrÅ. (2001). Exit: A follow-up and evaluation of the organisation for people wishing to leave racist and nazi groups. BrÅ.

Brylov, D. (2022). MARSEILLAISE VERSUS JIHADISM, OR THE FRENCH EXPERIENCE IN COMBATING ISLAMIST RADICALIZATION. World of the Orient, 115(2), 95-109.

Campelo, N., Oppetit, A., Hefez, S., Thompson, C., & Cohen, D. (2018). Feedback after 2 years of specialized consultations to prevent radicalization in a child and adolescent psychiatry department. Neuropsychiatrie de l'Enfance et de l'Adolescence, 66(5), 286-293.

CEE Prevent Net (2020). 10+ 1 Good Practices in Preventing Intolerance, Discrimination, and Group Hatred in Central and Eastern Europe. CEE Prevent Net.

Colliver, C., & Davey, J. (2017). Cross-spectrum counter violent extremism: Prevention and intervention models. In S. Hohnstein & M. Herding (Eds.). Digitale Medien und politisch-weltanschaulicher Extremismus im Jugendalter (pp. 164 - 189). Deutsches Jugendinstitut.

de Galembert, C. (2016). Le « radical », une nouvelle figure de dangerosité carcérale aux contours flous / The "radical", a new figure of prison dangerousness with fuzzy contours. Critique Internationale, 3(72), 53.

Díaz, G., & Rodríguez, P. (2015). The European Union and Islamist Terrorism. Revista UNISCI(39), 175-188.

Donnet, C. (2020). Reports for the "risk of radicalization" in schools in France, a new tool for the regulation of Islam. Deviance Et Societe, 44(3), 420-452.

Euer, K., Van Vossole, A., Groenen, A., & Van Bouchaute, K. (2015). A strategy for the prevention of the violent radicalisation of the young. Politiques sociales, 3-4.

Fernández Abad, C. (2022). LA PREVENCIÓN DE LA RADICALIZACIÓN EN EL ÁMBITO EDUCATIVO: UN ANÁLISIS CRÍTICO SOBRE LA PRETENSIÓN DE INVOLUCRAR AL PROFESORADO EN LA LUCHA CONTRA EL TERRORISMO. Estudios Penales y Criminológicos, 42, 1-34.

Ferzetti, F., & Lannutti, V. (2021). La prevenzione delle forme di ostilità e di radicalismo. Politiche migratorie e percorsi di inclusione giovanile in Europa e in Italia negli ultimi vent'anni. Rivista Trimestrale di Scienza dell'Amministrazione(4), 1.

García, M. D., Quijada, R., & Mora, R. (2023). The risk of radicalization among young immigrants. Guidelines for its prevention through social intervention. Cuadernos De Trabajo Social, 36(2), 231-240.

GCTF (2016). The Role of Families in Preventing and Countering Violent Extremism: Strategic Recommendations and Programming Options.

Glowacz, F. (2020). Desistance narratives of radicalized youths involved with terrorist groups. Criminologie, 53(1), 281-304.

Glowacz, F., & Collard, I. (2019). Le théâtre comme média de prévention de la radicalisation: évaluation du projet « nadia ». Revue Internationale de Criminologie et de Police Technique et Scientifique, 72(3), 308-321.

Grigoryeva, M. V., Sharov, A. A., & Zagranichny, A. I. (2022). Radicalizing youth social activism and finding support groups through digital and traditional means. Perspektivy Nauki i Obrazovania, 59(5), 446-461.

Helin, D., Glowacz, F., & Kumlu, S. (2015). What is the correct psycho-educational action to take against violent radicalisation among young people? Politiques sociales, 3-4.

Izluchenko, T. V. (2019). Features of the prevention of extremism in higher education institutions. Perspektivy Nauki i Obrazovania, 39(3), 73-87.

Kleeberg-Niepage, A., Medimond, & Univ Flensburg, D. P. F. G. (2013). Is young people's political extremism a developmental problem? 16th European Conference on Developmental Psychology, 101-103.

Kofi Annan Foundation (2020). Countering Violent Extremism: A Guide for Young People by Young People. Kofi Annan Foundation.

Manuel Tamayo, S., Fernández, I. B., & Rut Bermejo, C. (2021). La radicalización violenta de los jóvenes un reto en la construcción de sociedades seguras: una propuesta de tipología y escala de jóvenes vulnerables a procesos de radicalización violenta. Cuadernos de Gobierno y Administración Pública, 8(2), 119-130.

Medeiros, S. P. P. (2018). A INFLUÊNCIA DA DIMENSÃO RELIGIOSA (ISLÂMICA) NAS ESTRATÉGIAS DE SAÍDA DE JOVENS RADICALIZADOS. Universidade Católica Portuguesa.

Meere, F., & Lensink, L. (2015). Youth Counselling Against Radicalisation: Guidelines for Front-Line Workers. Verwey Jonker Instituut.

Métraux, J. C. (2019). “Radicalizations” question youth and family professionals. Cahiers Critiques de Therapie Familiale et de Pratiques de Reseaux, 63(2), 67-84.

Nagumo, E., Teles, L. F., & Silva, L. D. (2022). EDUCATION AND MISINFORMATION: MEDIA LITERACY, SCIENCE AND DIALOGUE. Etd Educacao Tematica Digital, 24, 220-237.

Nash, C. (2017). Youth Led Guide on Prevention of Violent Extremism Through Education. UNESCO and Mahatma Gandhi Institute of Education for Peace and Sustainable Development.

Nash, C., Nesterova, Y., Primrose, K., Chan, W. Y. A., Rios, R. A., Velásquez Flores, M. J., & Zowmi, A. (2017). Youth led guide on prevention of violent extremism through education.

Nash, C., Nesterova, Y., Primrose, K., Chan, W. Y. A., Rios, R. A., Velásquez Flores, M. J., & Zowmi, A. (2018). Action: guidelines for prevention of violent extremism.

NI'MAH, S. A. (2022). Peran Kiai Di Kabupaten Purbalingga Dalam Menangkal Radikalisme Pada Kegiatan Belajar Agama Islam Secara Online. PhD Thesis, UIN Prof. KH Saifuddin Zuhri.

Pazukhina, S. V. (2023). Psychological Foundations of Psychological and Pedagogical Prevention of Extremism and Protest Behavior among Young People. Psychology and Law, 13(2), 206-223.

Prinzjakowitsch, W. (2018). The role of non-formal education in P/CVE. RAN Issue Paper. Annex to RAN's Manifesto for Education. RAN Europe

Radicalisation Awareness Network (2015). Preventing radicalisation to terrorism and violent extremism: Educating young people

Rau, T., Heimgartner, A., Fegert, J. M., & Allroggen, M. (2020). Do radicalized persons have access to psychotherapeutic support?: Selected results of guided interviews. Psychotherapeut, 65(4), 297-303.

Rofik, M. N. (2021). Implementasi Program Moderasi Beragama Di Kementerian Agama Kabupaten Banyumas Pada Lingkungan Sekolah. (29309410), 149.

Rossi, F. (2021). The parabola of women and minors in the “global caliphate”: Vulnerability, illusions and traumas on the path from joining to rejecting totalitarian “jihad”. Rassegna Italiana di Criminologia, 15(2), 130-136.

Ryan, C. (2023). L'antiterrorisme et Les Droits de L'enfant Au Canada: Recalculer L'équation du Principe Fondamental de Proportionnalité de La Détermination de La Peine: Une Approche Centrée Sur Les Droits de L'enfant. Universite de Moncton.

Serdyuk, N. V., Grishchenko, L. L., & Stolyarenko, A. M. (2018). Psychological and pedagogical aspects of the prevention of extremism in youth. Psychology and Law, 8(3), 167-178.

SEZGİN, F., & GÜNEŞ, İ. D. (2013). TÜRKİYE'DE ORTAÖĞRETİM DERS KİTAPLARINDA TERÖR KONUSUNUN İŞLENİŞİ. Turkish Journal of Police Studies / Polis Bilimleri Dergisi, 15(4), 109-134.

Spiess, E., Kaminski, S., & Dukat, U. (2006). Sensitisation vis-a-vis xenophobia and racism - Results of a scientific monitoring of the XENOS project "Concept Workshop against Right Extremism". Gruppendynamik Und Organisationsberatung, 37(4), 347-362.

Swedish Agency for Youth and Civil Society (2018). Crack the code!—A guide on how public stakeholders and civil society can work together to prevent violent extremism.

Tolvaišis, L. L., & Jovanovic, P. (2022). Legislative and Practical Aspects of Countering Extremism in Sports: the Experience of Serbia. Teoriya i Praktika Fizicheskoy Kultury, 2022(2), 71-73.

Velički, D., & Perković, M. (2010). Strategies for combating and preventing right-wing extremism in the federal Republic of Germany. Drustvena Istrazivanja, 19(6), 1163-1181.

Vertone, L. (2021). RADIP-JF—Radicalization intervention program in juvenile forensics. Forensische Psychiatrie, Psychologie, Kriminologie, 15(2), 119-126.

Winarni, L. N. (2020). THE EXISTENCE OF PANCASILA IN FACING THREATS AGAINST DIVERSITY. Jurnal IUS Kajian Hukum dan Keadilan, 8(1), 89-96.

Woltman, P., & Gssime, Y. (2018). Ex Post Paper: Empowering Young People to Successfully Participate in PCVE. RAN Centre of Excellence.

World Organisation for Resource Development & Education (WORDE). The Montgomery County Model.

Zuschke, A. Extremismus im Klassenzimmer-Theoretische Chancen und Möglichkeiten des Politikunterrichtes.(Extremism in the Class Room. Theoretical Chances and Perspectives for Teaching Politics). Journal for Deradicalization (3), 135-148.

**Exclusion: Duplicate**

Bonnell, J., Copestake, P., Kerr, D., Passy, R., Reed, C., Salter, R., Sarwar, S., & Sheikh, S. (2011). Teaching Approaches that Help Build Resilience to Extremism Among Young People: Research Brief. UK Department for Education.

Christensen, T. W. (2015). How extremist experiences become valuable knowledge in EXIT programmes. Journal for Deradicalization (3), 92-134.

Christensen, T. W. (2015). When good intentions are not enough-A successful mentor-mentee relation requires a deliberated practice. Psyke & Logos, 36(1), 242-265.

Gielen, A.-J. (2015). Supporting families of foreign fighters. A realistic approach for measuring the effectiveness. Journal for Deradicalization (2), 21-48.

Gielen, A.-J., & Dijkman, A. (2019). Evidence-based informed policy design for support groups for families of foreign fighters: Ex ante application of realistic evaluation and review. Journal for Deradicalization (20), 232-271.

Haugstvedt, H. (2019). Trusting the mistrusted: Norwegian social workers’ strategies in preventing radicalization and violent extremism. Journal for Deradicalization, (19), 149-184.

Haugstvedt, H. (2022). The role of social support for social workers engaged in preventing radicalization and violent extremism. Nordic Social Work Research, 12(1), 166-179.

Haugstvedt, H., & Gunnarsdottir, H. M. (2023). Managing role expectations and emotions in encounters with extremism: Norwegian social workers’ experiences. Qualitative Social Work, 22(1), 67-85.

Haugstvedt, H., & Tuastad, S. E. (2023). "It Gets a Bit Messy": Norwegian Social Workers' Perspectives on Collaboration with Police and Security Service on Cases of Radicalisation and Violent Extremism. Terrorism and Political Violence, 35(3), 677-693.

Jackson, B. A., Rhoades, A. L., Reimer, J. R., Lander, N., Costello, K., & Beaghley, S. (2019). Building an Effective and Practical National Approach to Terrorism Prevention. RAND Corporation

Skiple, A. (2018). Youth delinquency or everyday racism? Front-line professionals’ perspectives on preventing racism and intolerance in Sweden. Journal for Deradicalization (14), 52-78.

Skiple, A. (2020). The Importance of Significant Others in Preventing Extremism: The Philosophy and Practice of the Swedish Tolerance Project. Young, 28(4), 422-438.

1. Based on Sterne, J. A. C., Higgins, J. P. T., Elbers, R. G. , Reeves, B. C. and the development group for ROBINS- I. Risk of Bias in Non-randomized Studies of Interventions (ROBINS-I): detailed guidance, updated 12 October 2016. Available from http://www.riskofbias.info [accessed 12^th^ April 2022]. [↑](#footnote-ref-1)
2. Based on Sterne, J. A. C., Savović, J., Page, M. J., Elbers, R. G., Blencowe, N. S., Boutron, I., Cates, C. J., Cheng, H-Y., Corbett, M. S., Eldridge, S. M., Hernán, M. A., Hopewell, S., Hróbjartsson, A., Junqueira, D. R., Jüni, P., Kirkham, J. J., Lasserson, T., Li, T., McAleenan, A., Reeves, B. C., Shepperd, S., Shrier, I., Stewart, L. A., Tilling, K., White, I. R., Whiting, P. F., & Higgins, J. P. T. (2019) RoB 2: a revised tool for assessing risk of bias in randomised trials. BMJ, 366, l4898 [↑](#footnote-ref-2)
3. https://www.ephpp.ca/PDF/Quality%20Assessment%20Tool_2010_2.pdf [↑](#footnote-ref-3)
4. Available at https://casp-uk.net/casp-tools-checklists/. The original tool also includes a question relating to how the value of the research that was not used to assess studies. [↑](#footnote-ref-4)
5. Separate row for each intervention examined in individual study. [↑](#footnote-ref-5)
6. Based on coding tool first developed by Mazerolle et al. (2020) as used in Lewis et al. (2023) [↑](#footnote-ref-6)
7. Based on coding tool first developed by Mazerolle et al. (2020) as used in Lewis et al. (2023). [↑](#footnote-ref-7)
